# Construction of bovine whole-genome radiation hybrid and linkage maps using high-throughput genotyping

**DOI:** 10.1111/j.1365-2052.2006.01564.x

**Published:** 2007-04-01

**Authors:** S D McKay, R D Schnabel, B M Murdoch, J Aerts, C A Gill, C Gao, C Li, L K Matukumalli, P Stothard, Z Wang, C P Van Tassell, J L Williams, J F Taylor, S S Moore

**Affiliations:** * Department of Agricultural, Food and Nutritional Science, University of Alberta Edmonton, AB, Canada; † Division of Animal Sciences, University of Missouri Columbia, MO, USA; ‡ Division of Genetics and Genomics, Roslin Institute (Edinburgh), Midlothian Scotland, EH25 9PS UK; § Department of Animal Science, Texas A&M University College Station, TX, USA; ** Bovine Functional Genomics Laboratory, U.S. Department of Agriculture, Agricultural Research Service Beltsville, MD, USA; †† Bioinformatics and Computational Biology, George Mason University Manassas, VA, USA

**Keywords:** bovine, illumina, map, single nucleotide polymorphism

## Abstract

High-density whole-genome maps are essential for ordering genes or markers and aid in the assembly of genome sequence. To increase the density of markers on the bovine radiation hybrid map, and hence contribute to the assembly of the bovine genome sequence, an Illumina® BeadStation was used to simultaneously type large numbers of markers on the Roslin-Cambridge 3000 rad bovine–hamster whole-genome radiation hybrid panel (WGRH_3000_). In five multiplex reactions, 6738 sequence tagged site (STS) markers were successfully typed on the WGRH_3000_ panel DNA. These STSs harboured SNPs that were developed as a result of the bovine genome sequencing initiative. Typically, the most time consuming and expensive part of creating high-density radiation hybrid (RH) maps is genotyping the markers on the RH panel with conventional approaches. Using the method described in this article, we have developed a high-density whole-genome RH map with 4690 loci and a linkage map with 2701 loci, with direct comparison to the bovine whole-genome sequence assembly (Btau_2.0) in a fraction of the time it would have taken with conventional typing and genotyping methods.

## Introduction

Radiation hybrid (RH) mapping is a powerful tool that can be utilized for the production of in-depth comparative maps of single chromosomes and whole genomes. The construction of an RH map relies upon scoring the presence or absence of markers in a hybrid cell panel constructed by fusing irradiated donor cells with recipient rodent cells. Conventional methods for typing markers on RH panels rely on individual or low-complexity multiplex PCR assays for all typed markers on DNA derived from each of the cell lines in the panel, followed by agarose gel electrophoresis to detect the presence or absence of the marker in individual RH cell lines. The proportion of the DNA fragments from the donor genome that harbor any particular marker will vary between the cell lines and so signal intensity varies among the positive cells. Signal differences as well as PCR artifacts and differentiation of rodent vs. target species amplification products usually necessitates running assays in duplicate and on occasion in triplicate to confirm results.

Radiation hybrid mapping in cattle was established in the late 1990s with the creation of the 5000-rad panel (Womack *et al.* 1997). Since then, bovine RH panels of various resolution have been created and utilized, including 3000- (Williams *et al.* 2002), 7000- (Itoh *et al.* 2005) and 12 000- (Rexroad *et al.* 2000) rad panels. In this study, we used the Illumina® BeadStation 500G to type a large number of markers which were distributed throughout the bovine genome on the Roslin-Cambridge 3000-rad bovine–hamster whole-genome radiation hybrid panel. The accuracy of typing was confirmed by building the markers into radiation hybrid maps together with 1125 markers that had been conventionally typed on the RH panel. With this high-throughput approach, 4690 loci were rapidly mapped. In addition, paternally related registered Angus A1 sires were genotyped with 2701 of the 4690 loci. The resulting human–cattle comparative maps have direct comparison to the bovine whole genome sequence assembly (Btau_2.0).

## Materials and methods

### Marker selection

Information for sequences containing SNPs was obtained from public databases (http://www.ncbi.nlm.nih.gov/projects/SNP/, ftp://ftp.hgsc.bcm.tmc.edu/pub/data/Btaurus/snp). Oligonucleotides were designed, synthesized and assembled into oligo pooled assays (OPA) by Illumina Inc.

### Oligo pooled assays

GoldenGate SNP assays were designed at Illumina using a proprietary design tool that selects oligonucleotides with balanced melting temperatures at each locus and compares these oligonucleotide sequences to an internal database to ensure sequence uniqueness. Assay designs were based on build Btau 2.0 of the cattle genome sequence. Sequences of up to 200 bases in length, with at least 50 bases on each side of the SNP of interest, were used as input for the design program. Ambiguities or additional SNPs were indicated as ‘N’ or IUPAC codes for mixed bases. The portion of the oligos that bound to the genomic sequence was all between 20 and 23 bases long, with a pair of oligos designed for each locus. Therefore, the footprint of each assay on the genomic DNA was usually 45–55 bases long, including a gap of 1–20 bases between the two oligos. Of the 10 631 candidate SNPs submitted for scoring, 6738 were included as part of the final assay pool.

### Typing of the RH panel

**Figure 1:**
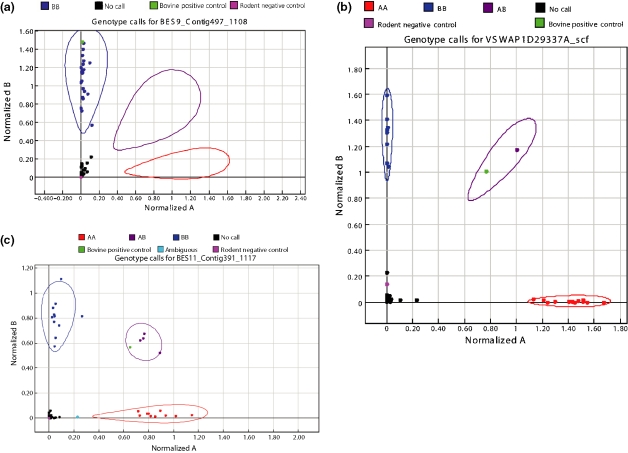
A Cartesian plot depicting radiation hybrid typing generated by the Illumina® BeadStation. (a) Clones located under the sweeping arc from 0.30 on the *y*-axis to 0.30 on the *x*-axis were identified as negatives and labelled no calls (U). Once selected, the negative cluster was excluded and the calling algorithm was rerun. Clones containing BB genotypes were scored as present. Clones containing U genotypes were scored as absent. (b) Clones located under the sweeping arc from 0.40 on the *y*-axis to 0.40 on the *x*-axis were identified as negatives and labelled no calls (U). Once selected, the negative cluster was excluded and the calling algorithm was rerun. Clones containing A, H and B genotypes were scored as present. Clones containing U genotypes were scored as absent. (c) Clones located under the sweeping arc from 0.10 on the *y*-axis to 0.10 on the *x*-axis were identified as negatives and labelled no calls (U). Once selected, the negative cluster was excluded and the calling algorithm was rerun. One clone was labelled as ambiguous and marked as ‘2’. It is located at approximately 0 on the *y*-axis and just to the right of 0.2 on the *x*-axis. Clones containing A, H and B genotypes were scored as present. Clones containing U genotypes were scored as absent. Clones marked as ‘2’ were scored as ambiguous.

DNA from the 94 cell lines of the Roslin-Cambridge 3000-rad bovine–hamster whole genome radiation hybrid (WGRH_3000_) panel plus hamster and bovine control DNA samples (Williams *et al.* 2002) were typed following the manufacturer's protocol for the Illumina® BeadStation 500G (Oliphant *et al.* 2002; http://www.illumina.com). Typing was carried out in five multiplex reactions which included all 6738 loci in the final assay pool. Illumina®gencall software was used to manually score the presence or absence of loci across the RH panel clones. The donor bull used to construct the RH panel was either homozygous or heterozygous for each of the markers. For the homozygous markers, positive and negative clones formed two distinct clusters (Fig. 1a), allowing simple scoring of positives and negatives. Clones within the negative control cluster were marked using the ‘exclude selected sample’ option of the gencall software, so these clones were identified as ‘No Calls’, which produced ‘U genotypes.’ For these markers, RH vectors were generated for these loci by scoring the clones as present (1) or the U genotypes as absent (0). In contrast, the heterozygous markers produced four clusters (Fig. 1b): clones harbouring the A allele, clones with the B allele, clones with both alleles (H) and clones with no alleles (U). For heterozygous markers, RH vectors were generated by combining the A, B and H genotypes and scoring these cells as present (1) and scoring the U types as absent (0).

In conventional RH mapping studies, it is reasonably common to find markers that amplify clone DNA with low intensity and/or the amplification is difficult to replicate. These low-intensity positives can be attributed to mis-priming, to low target copy number within the clone, or to a mutation within the primer site in the animal's DNA used to construct the panel. These clones are usually scored as ambiguous (2) for the marker. These ambiguities were not eliminated with the high-throughput approach described here. As seen in Fig. 1c, hybrids were sometimes labelled as ambiguous. Vectors for these outliers were manually changed to ‘2’.

Of the 6738 loci that were typed on the RH panel, 4690 were assigned to chromosomes based on two-point analysis with 1125 markers previously assigned in an earlier version of the WGRH_3000_ map (Williams *et al.* 2002) using RH map (Lange *et al.* 1995). A minimum LOD score of 4.5 was used as evidence of linkage. Whole chromosome maps were constructed using the default algorithm of carthagene (Schiex & Gaspin 1997) as described (Williams *et al.* 2002). We typed 1423 SNPs that were physically separated by approximately 1–2 kb (Table S1); for these loci, only one locus of the pair was typed on the panel whereas the map position of the other locus was based upon their order in the Btau_2.0 assembly. Homologous synteny blocks (HSB) and inversions were defined according to the criteria of Murphy *et al.* (2005) and the accepted threshold for BLASTN hits was *E* < 0.00001.

### Genotypes for the linkage map

Genotypes for 80 paternally related registered Angus AI sires were determined for 3072 of the 6738 SNPs typed on the RH panel using the Illumina® BeadStation. These animals were a subset of a larger 14 generation pedigree composed of 1697 registered Angus sires (Morsci *et al.* 2006). Linkage analysis was performed using crimap (Green *et al.* 1990).

### Map construction

Homologous bovine sequence coordinates were obtained by BLASTN (Altschul *et al.* 1990) analysis of the 500-bp sequences harbouring each target SNP against the bovine sequence assembly (Btau_2.0). Orthologous human sequence coordinates were derived from University of California Santa Cruz (UCSC) alignments of bovine scaffolds (Btau_1.0) to the human assembly (Build 35). Only SNP markers with un-ambiguous match positions in the human and bovine genomes were retained. Approximately 40% of these SNPs were not assigned to a bovine chromosome in the Btau_2.0 assembly. The best alignment to Build 35 of the human assembly and the human–bovine comparative map of Itoh *et al.* (2005) were used to order these loci relative to the SNPs contained within the bovine sequence assembly.

A framework map was constructed using loci for which the best BLAST hit to the bovine assembly agreed with its map location predicted in the RH analysis. The next group of loci integrated into the map were those that had the best human BLAST hit mapped to an orthologous bovine location and in agreement with the RH map position. Any locus within a scaffold which could not be assigned by these processes but another locus in the same scaffold had previously been assigned was then integrated into the map. Following that, any locus from the linkage map that was significantly linked (LOD > 3.0) to an SNP already included in the map was integrated into the map only if an available RH location, bovine BLAST hit or human BLAST hit localized the SNP to the same chromosomal region. Finally we utilized the FLIPS5 option of crimap to identify ordering errors for each chromosome. This approach was primarily used to detect small inversions of loci groups which were not identified in the bovine–human comparative map or to correct erroneous inversions suggested by markers that were incorrectly ordered in the comparative map (Itoh *et al.* 2005).

## Results

Of the 6738 loci included in the Illumina oligo pooled assays, 5815 (86.3%) were successfully typed and 4690 of the 5815 (80.7%) were assigned to the RH map. These rates exceeded the 62.3% success rate in our laboratory using conventional RH typing methods (data not shown). Of the 4690 markers whose RH map locations were determined, 4334 were SNPs identified as a result of the bovine whole-genome sequencing initiative. Furthermore, 2701 of these markers were included in a high-density linkage map and used to estimate recombination distance among loci and to confirm locus order produced in the RH map (Table S2). At least two independent measures of support were needed for integrating loci into the linkage map, which resulted in 2701 of the 3072 markers (87.9%) being included in the map. Only 1801 (66.7%) of these loci were assigned chromosomal coordinates in Btau_2.0 (Table 1). Disagreement in marker order between the linkage map and Btau_2.0 were found on chromosomes 3, 7, 10, 15, 18, 27 and 29 and inversions on chromosomes 13 and 19. Comparisons between the linkage map and Btau_2.0 identified 133 of 2701 (4.9%) markers with chromosomal assignment discordancies (labelled in red on the Btau_2.0 maps in Figure S1). While Btau_2.0 chromosome 11 had 14 discordant markers, Btau_2.0 chromosomes 4, 10, 16 and 28 had no discordant markers. Human sequence orthologs which were supported by either RH map or Btau_2.0 positions were identified for 1495 (55.3%) of the 2701 loci on the linkage map (Table 2). These coordinates indicate 185 blocks of bovine–human synteny and 28 putative major inversions (Table 2 and Table S3).

**Table 1:** Summary statistics when comparing Btau_2.0 and the linkage map.

Btau_2.0	Btau_2.0 length (Mb)	No.BLASTN hits	Avg. marker spacing (Mb)	No. dicordant markers on Btau_2.0
1	97.2	71	1.37	6
2	83.1	89	0.93	11
3	81.5	121	0.67	10
4	66.4	67	0.99	0
5	75.4	107	0.70	5
6	65.6	67	0.98	6
7	67.8	74	0.92	4
8	60.4	53	1.14	3
9	62.6	64	0.98	11
10	66.4	67	0.99	0
11	86.5	89	0.97	14
12	46.8	44	1.06	1
13	61.2	65	0.94	3
14	46.2	42	1.10	4
15	53.5	74	0.72	1
16	56.5	69	0.82	0
17	44	41	1.07	2
18	55.5	58	0.96	3
19	54.6	53	1.03	9
20	40.5	44	0.92	1
21	46.3	51	0.91	6
22	45.7	41	1.11	2
23	41.5	75	0.55	12
24	44.7	47	0.95	2
25	39.6	62	0.64	9
26	34.9	47	0.74	3
27	24.6	25	0.98	2
28	32.6	49	0.67	0
29	43.6	45	0.97	3
Total	1625.2	1801	0.90	133

**Table 2:** Summary statistics of human–cattle comparative maps.

HSA	HSA length (Mbp)	No. BLASTN hits	Average marker spacing (Mb)	No. human synteny blocks	No. putative inversions
1	240.2	171	1.40	20	6
2	222.2	140	1.59	15	3
3	192.7	115	1.68	17	4
4	174.4	90	1.94	15	2
5	172	98	1.76	12	3
6	162	85	1.91	6	2
7	155.1	73	2.12	8	1
8	129.1	51	2.53	6	3
9	126.9	67	1.89	10	1
10	128.8	100	1.29	10	1
11	132.6	99	1.34	11	0
12	129	82	1.57	13	0
13	89.7	40	2.24	8	0
14	81.5	40	2.04	6	0
15	72.1	43	1.68	6	0
16	81.9	47	1.74	5	0
17	63.5	30	2.12	4	0
18	66.2	40	1.66	5	1
19	51.2	19	2.69	2	0
20	60.1	31	1.94	4	1
21	31.3	19	1.65	1	0
22	23	15	1.53	1	0
Total	2585.5	1495	1.73	185	28

The overall length of the autosomal RH map was 80086.8 cR_3000_ (Table S1) with an average intermarker distance of 13.77 cR_3000_ between markers. Chromosome 5 was the most densely mapped chromosome, with 366 markers and a total length of 3798.5 cR_3000_ compared to 177 markers that were mapped by linkage with a total length of 133.9 cM. Conversely, BTA27 was the most sparsely mapped chromosome, with 92 markers on the RH map and a total chromosomal length of 1298.5 cR_3000_ compared to 40 markers on the linkage map with a total chromosomal length of 51.6 cM.

There were 1125 common markers between the RH map in this study and the USDA-MARC linkage map. The RH and linkage maps described here were in general agreement to the USDA-MARC linkage map (Figure S1). However, there were inversions of marker orders on BTA2, 9, 10, 19, 26 and 27 between the RH and MARC maps. Major order disagreements were found on BTA19, where a substantial marker gap at the proximal end of the chromosome prevented alignment of the RH and linkage maps.

## Discussion

An updated WGRH_3000_ map was developed in this study, including 4690 new SNP loci and 1125 previously typed markers. Of these, 4334 SNPs were identified in the bovine genome sequencing project (ftp://ftp.hgsc.bcm.tmc.edu/pub/data/Btaurus/snp/Btau20040927/bovine-snp.txt). Linkage maps containing 2701 loci were constructed for all autosomes, and human–cattle comparative maps were built. We report cR_3000_ locations for 4690 markers, cM location for 2701 markers and Mb positions for 1801 of the 4690 loci. This approach for high-throughput RH mapping has allowed us to rapidly assemble comprehensive maps of the bovine genome.

Generally, linkage maps are conducive to the high-level ordering of loci along a chromosome with a resolution that is generally determined by the number of informative meioses. On the other hand, RH maps are often superior for fine-mapping closely linked loci (Mellersh *et al.* 2000). Alignment of the high-density linkage map in this study with the Btau_2.0 assembly confirmed the overall quality of Btau_2.0 but indicated there were 133 incorrectly assigned loci and localized inversions in scaffold ordering within the assembly. Because the marker density of the maps presented here was higher than that used to assign and order the sequence scaffolds in the Btau_2.0 genome assembly, we were able to position additional scaffolds. These data have been contributed to the bovine composite map (W. A. Snelling *et al.* personal communication) and will be used for the next assembly of the bovine genome sequence.

The use of high-density SNP assays such as the Illumina GoldenGate or Infinium platforms harbouring as many as 64 000 loci per assay will allow typing of a whole-genome RH panel in as little as one day. Thus, the generation of *de novo* RH maps from short sequences can now be accomplished rapidly and cost effectively. This strategy appears to be particularly useful for the development of high-content maps necessary for the assembly of whole-genome shotgun sequences. On the other hand, for those genomes where a sequence assembly is not likely in the near future, the approach described here may provide an effective means of generating comprehensive comparative maps. High-throughput RH mapping may contribute towards low-cost genome sequencing by coupling the methodology described here with 454 sequencing technology (Margulies *et al.* 2005).

## Supplementary Material

The following supplementary material is available for this article online from http://www.blackwell-synergy.com/doi/full/10.1111/j.1365-2052.2007.01564.x

Table S1 Summary statistics of University of Alberta radiation hybrid maps.

Table S2 Map locations, Btau_2.0 and orthologous human information on markers that were both radiation hybrid and linkage mapped.

Table S3 Radiation hybrid and human map coordinates for all human synteny blocks.

Figure S1 Human-cattle comparative maps and bovine genome assembly comparative maps for each chromosome. Markers whose names appear in red on the bovine genome assembly version 2 (Btau_2.0) maps are markers whose chromosomal assignment is incorrect on Btau_2.0. Comparative maps of the USDA-MARC linkage map (MARC), the University of Alberta radiation hybrid map (UofA) and the University of Missouri-Columbia (UMC) linkage map for each chromosome. Please note: Blackwell Publishing is not responsible for the content or functionality of any supplementary materials supplied by the authors.
