# Supplementary material for: Construction of bovine whole-genome radiation hybrid and linkage maps using high-throughput genotyping
Source: Anim Genet. 2007 Apr 1;38(2):120–5. doi: 10.1111/j.1365-2052.2006.01564.x (PMC2063635; doi:10.1111/j.1365-2052.2006.01564.x)
Supplement: Figure S1 — Human-cattle comparative maps and bovine genome assembly comparative maps for each chromosome. Markers whose names appear in red on the bovine genome assembly version 2 (Btau_2.0) maps are markers whose chromosomal assignment is incorrect on Btau_2.0. Comparative maps of the USDA-MARC linkage map (MARC), the University of Alberta radiation hybrid map (UofA) and the University of Missouri-Columbia (UMC) linkage map for each chromosome. [file age0038-0120_fig_S1.pdf]

BTA1

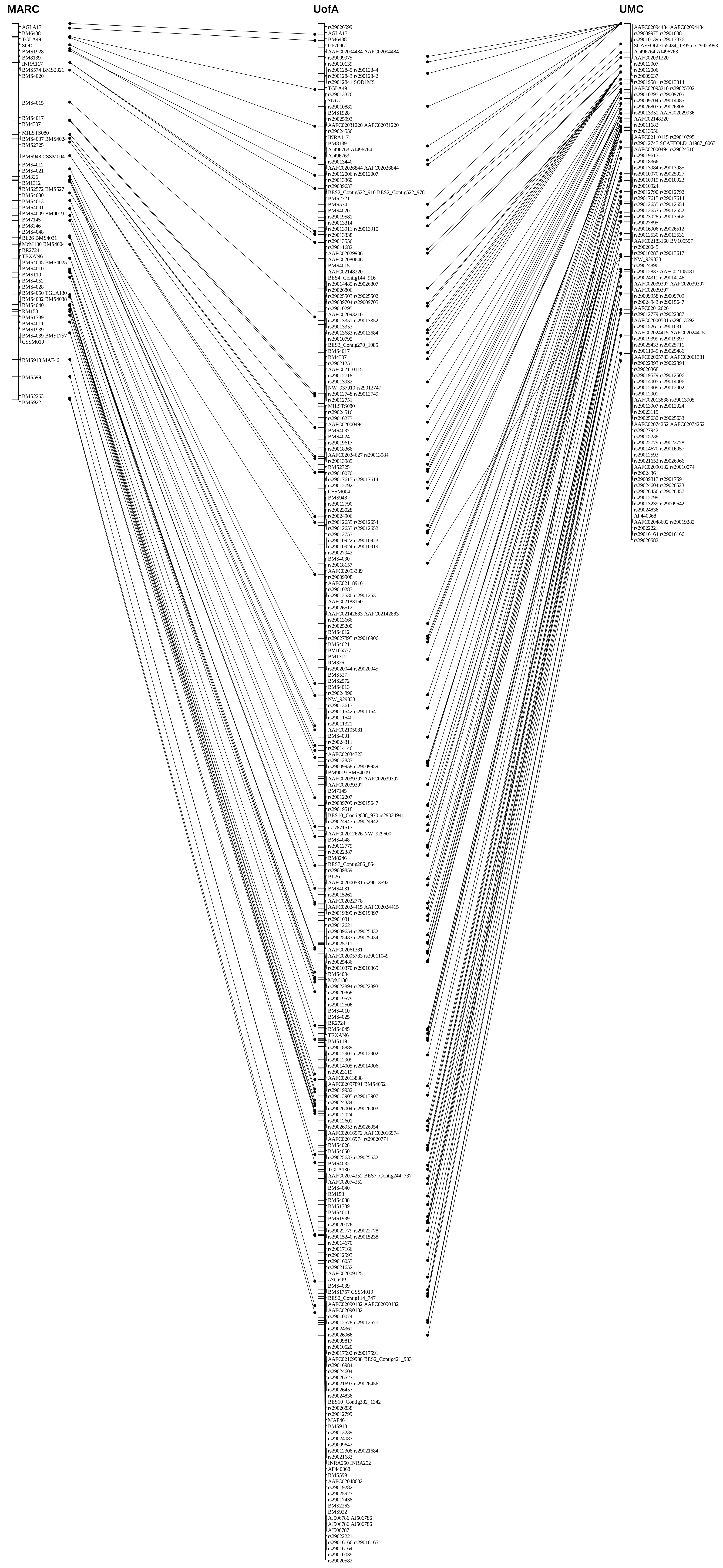

BTA1

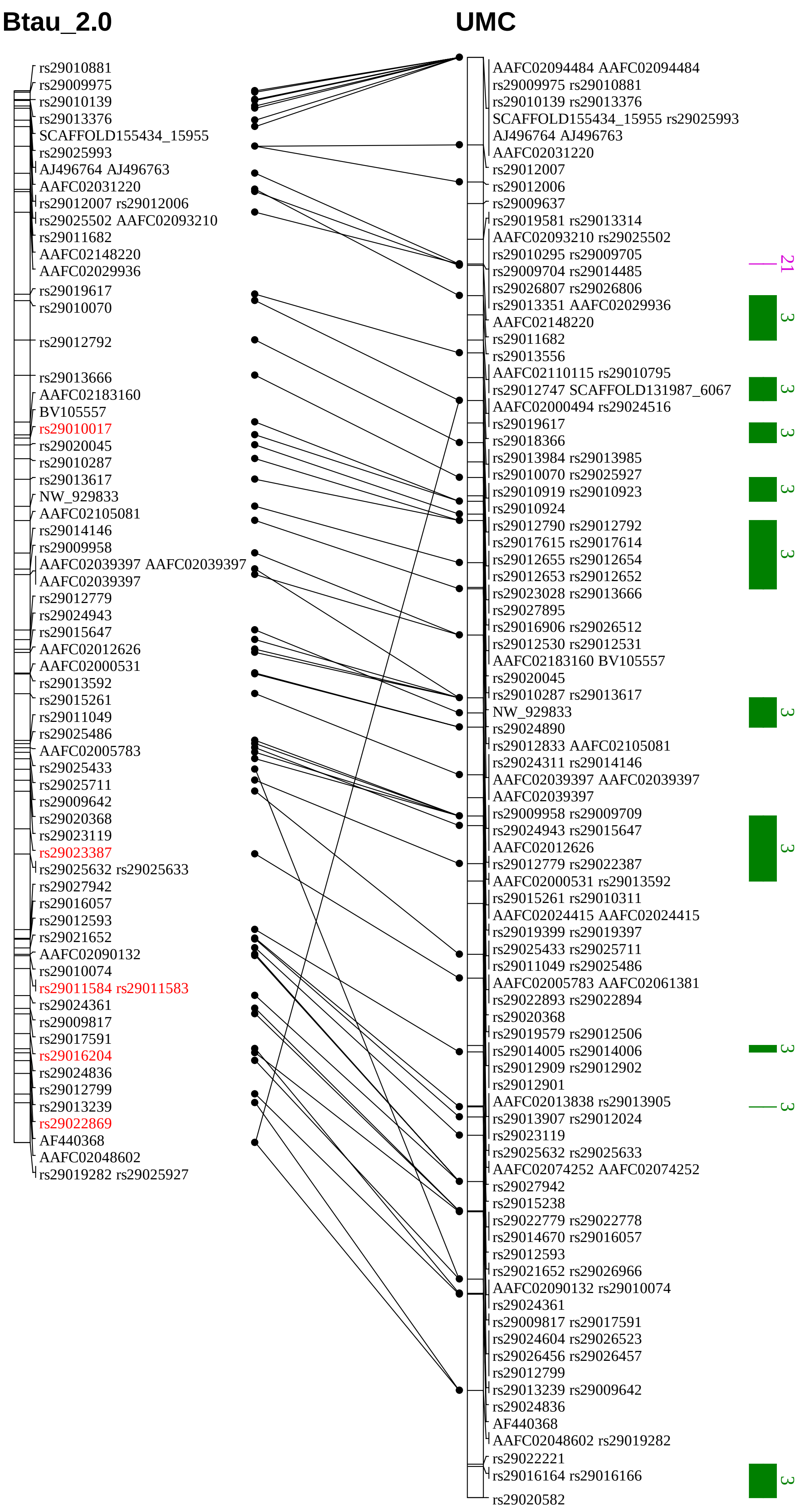
Btau_2.0
rs29010881
rs29009975
rs29010139
rs29013376
SCAFFOLD155434_15955
rs29025993
AJ496764 AJ496763
AAFC02031220
rs29012007 rs29012006
rs29025502 AAFC02093210
rs29011682
AAFC02148220
AAFC02029936
rs29019617
rs29010070
rs29012792
rs29013666
AAFC02183160
BV105557
rs29010017
rs29020045
rs29010287
rs29013617
NW_929833
AAFC02105081
rs29014146
rs29009958
AAFC02039397 AAFC02039397
AAFC02039397
rs29012779
rs29024943
rs29015647
AAFC02012626
AAFC02000531
rs29013592
rs29015261
rs29011049
rs29025486
AAFC02005783
rs29025433
rs29025711
rs29009642
rs29020368
rs29023119
rs29023387
rs29025632 rs29025633
rs29027942
rs29016057
rs29012593
rs29021652
AAFC02090132
rs29010074
rs29011584 rs29011583
rs29024361
rs29009817
rs29017591
rs29016204
rs29024836
rs29012799
rs29013239
rs29022869
AF440368
AAFC02048602
rs29019282 rs29025927
UMC
AAFC02094484 AAFC02094484
rs29009975 rs29010881
rs29010139 rs29013376
SCAFFOLD155434_15955 rs29025993
AJ496764 AJ496763
AAFC02031220
rs29012007
rs29012006
rs29009637
rs29019581 rs29013314
AAFC02093210 rs29025502
rs29010295 rs29009705
rs29009704 rs29014485
rs29026807 rs29026806
rs29013351 AAFC02029936
AAFC02148220
rs29011682
rs29013556
AAFC02110115 rs29010795
rs29012747 SCAFFOLD131987_6067
AAFC02000494 rs29024516
rs29019617
rs29018366
rs29013984 rs29013985
rs29010070 rs29025927
rs29010919 rs29010923
rs29010924
rs29012790 rs29012792
rs29017615 rs29017614
rs29012655 rs29012654
rs29012653 rs29012652
rs29023028 rs29013666
rs29027895
rs29016906 rs29026512
rs29012530 rs29012531
AAFC02183160 BV105557
rs29020045
rs29010287 rs29013617
NW_929833
rs29024890
rs29012833 AAFC02105081
rs29024311 rs29014146
AAFC02039397 AAFC02039397
AAFC02039397
rs29009958 rs29009709
rs29024943 rs29015647
AAFC02012626
rs29012779 rs29022387
AAFC02000531 rs29013592
rs29015261 rs29010311
AAFC02024415 AAFC02024415
rs29019399 rs29019397
rs29025433 rs29025711
rs29011049 rs29025486
AAFC02005783 AAFC02061381
rs29022893 rs29022894
rs29020368
rs29019579 rs29012506
rs29014005 rs29014006
rs29012909 rs29012902
rs29012901
AAFC02013838 rs29013905
rs29013907 rs29012024
rs29023119
rs29025632 rs29025633
AAFC02074252 AAFC02074252
rs29027942
rs29015238
rs29022779 rs29022778
rs29014670 rs29016057
rs29012593
rs29021652 rs29026966
AAFC02090132 rs29010074
rs29024361
rs29009817 rs29017591
rs29024604 rs29026523
rs29026456 rs29026457
rs29012799
rs29013239 rs29009642
rs29024836
AF440368
AAFC02048602 rs29019282
rs29022221
rs29016164 rs29016166
rs29020582
21
3
3
3
3
3
3
3
3
3
3

BTA2

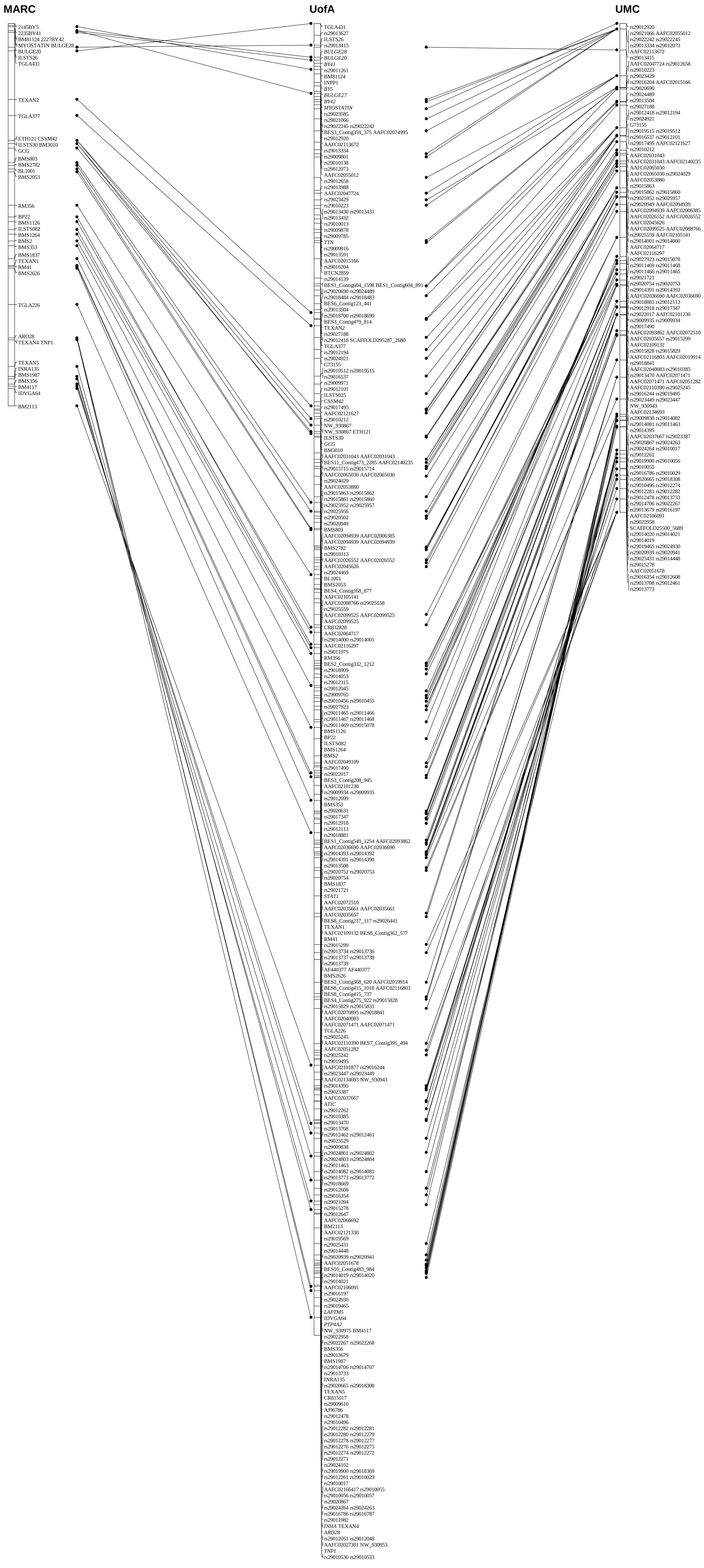
MARC
UofA
UMC
2145BY5
2235BY41
BM81124 2227BY42
MYOSTATIN BULGE28
BULGE20
ILSTS26
TGLA431
TEXAN2
TGLA377
ETH121 CSSM42
ILSTS30 BM3010
GCG
BMS803
BMS2782
BL1001
BMS2053
RM356
BP22
BMS1126
ILSTS082
BMS1264
BMS2
BMS353
BMS1837
TEXAN1
RM41
BMS2626
TGLA226
ARO28
TEXAN4 TNP1
TEXAN5
INRA135
BMS1987
BMS356
BM4117
IDVGA64
BM2113

BTA2

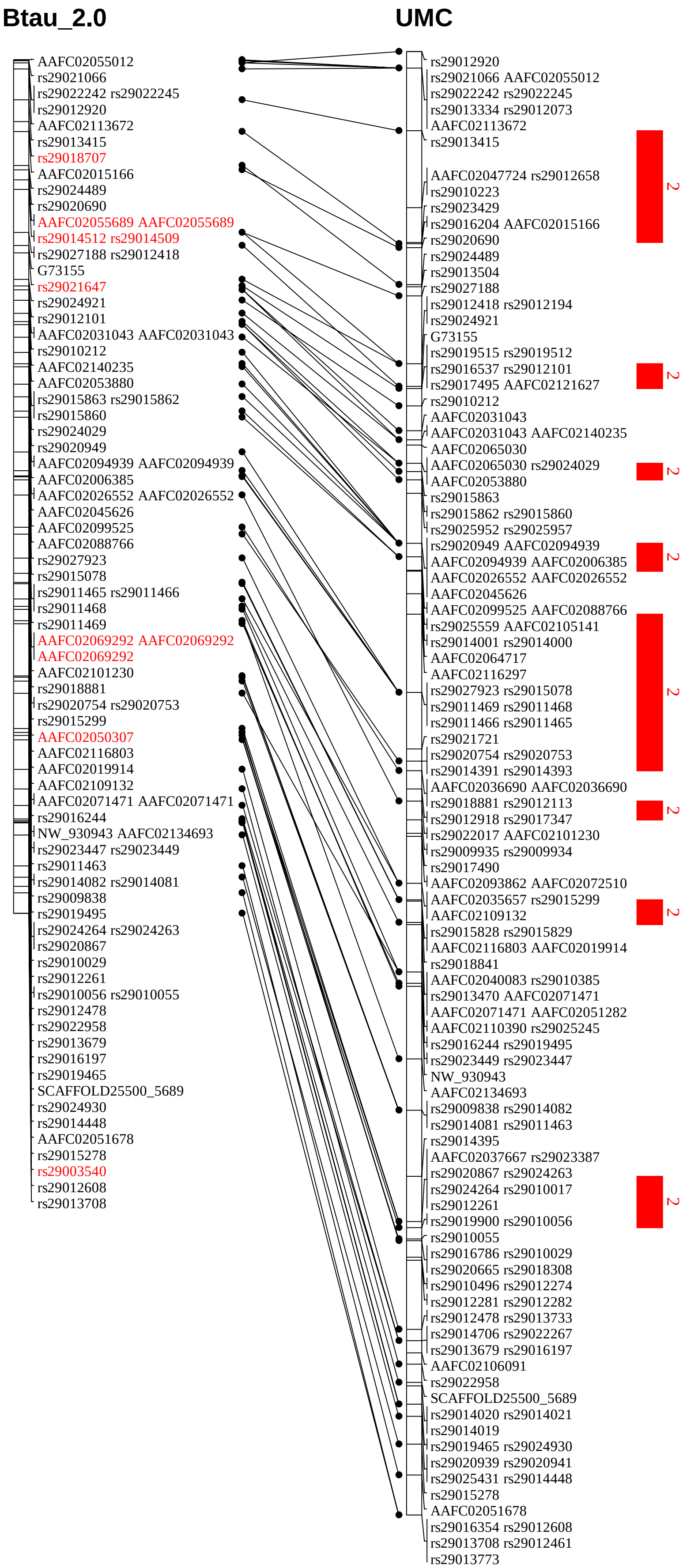
Btau_2.0
UMC
AAFC02055012
rs29021066
rs29022242 rs29022245
rs29012920
AAFC02113672
rs29013415
rs29018707
AAFC02015166
rs29024489
rs29020690
AAFC02055689 AAFC02055689
rs29014512 rs29014509
rs29027188 rs29012418
G73155
rs29021647
rs29024921
rs29012101
AAFC02031043 AAFC02031043
rs29010212
AAFC02140235
AAFC02053880
rs29015863 rs29015862
rs29015860
rs29024029
rs29020949
AAFC02094939 AAFC02094939
AAFC02006385
AAFC02026552 AAFC02026552
AAFC02045626
AAFC02099525
AAFC02088766
rs29027923
rs29015078
rs29011465 rs29011466
rs29011468
rs29011469
AAFC02069292 AAFC02069292
AAFC02069292
AAFC02101230
rs29018881
rs29020754 rs29020753
rs29015299
AAFC02050307
AAFC02116803
AAFC02019914
AAFC02109132
AAFC02071471 AAFC02071471
rs29016244
NW_930943 AAFC02134693
rs29023447 rs29023449
rs29011463
rs29014082 rs29014081
rs29009838
rs29019495
rs29024264 rs29024263
rs29020867
rs29010029
rs29012261
rs29010056 rs29010055
rs29012478
rs29022958
rs29013679
rs29016197
rs29019465
SCAFFOLD25500_5689
rs29024930
rs29014448
AAFC02051678
rs29015278
rs29003540
rs29012608
rs29013708
rs29012920
rs29021066 AAFC02055012
rs29022242 rs29022245
rs29013334 rs29012073
AAFC02113672
rs29013415
AAFC02047724 rs29012658
rs29010223
rs29023429
rs29016204 AAFC02015166
rs29020690
rs29024489
rs29013504
rs29027188
rs29012418 rs29012194
rs29024921
G73155
rs29019515 rs29019512
rs29016537 rs29012101
rs29017495 AAFC02121627
rs29010212
AAFC02031043
AAFC02031043 AAFC02140235
AAFC02065030
AAFC02065030 rs29024029
AAFC02053880
rs29015863
rs29015862 rs29015860
rs29025952 rs29025957
rs29020949 AAFC02094939
AAFC02094939 AAFC02006385
AAFC02026552 AAFC02026552
AAFC02045626
AAFC02099525 AAFC02088766
rs29025559 AAFC02105141
rs29014001 rs29014000
AAFC02064717
AAFC02116297
rs29027923 rs29015078
rs29011469 rs29011468
rs29011466 rs29011465
rs29021721
rs29020754 rs29020753
rs29014391 rs29014393
AAFC02036690 AAFC02036690
rs29018881 rs29012113
rs29012918 rs29017347
rs29022017 AAFC02101230
rs29009935 rs29009934
rs29017490
AAFC02093862 AAFC02072510
AAFC02035657 rs29015299
AAFC02109132
rs29015828 rs29015829
AAFC02116803 AAFC02019914
rs29018841
AAFC02040083 rs29010385
rs29013470 AAFC02071471
AAFC02071471 AAFC02051282
AAFC02110390 rs29025245
rs29016244 rs29019495
rs29023449 rs29023447
NW_930943
AAFC02134693
rs29009838 rs29014082
rs29014081 rs29011463
rs29014395
AAFC02037667 rs29023387
rs29020867 rs29024263
rs29024264 rs29010017
rs29012261
rs29019900 rs29010056
rs29010055
rs29016786 rs29010029
rs29020665 rs29018308
rs29010496 rs29012274
rs29012281 rs29012282
rs29012478 rs29013733
rs29014706 rs29022267
rs29013679 rs29016197
AAFC02106091
rs29022958
SCAFFOLD25500_5689
rs29014020 rs29014021
rs29014019
rs29019465 rs29024930
rs29020939 rs29020941
rs29025431 rs29014448
rs29015278
AAFC02051678
rs29016354 rs29012608
rs29013708 rs29012461
rs29013773
2
2
2
2
2
2
2
2

BTA3

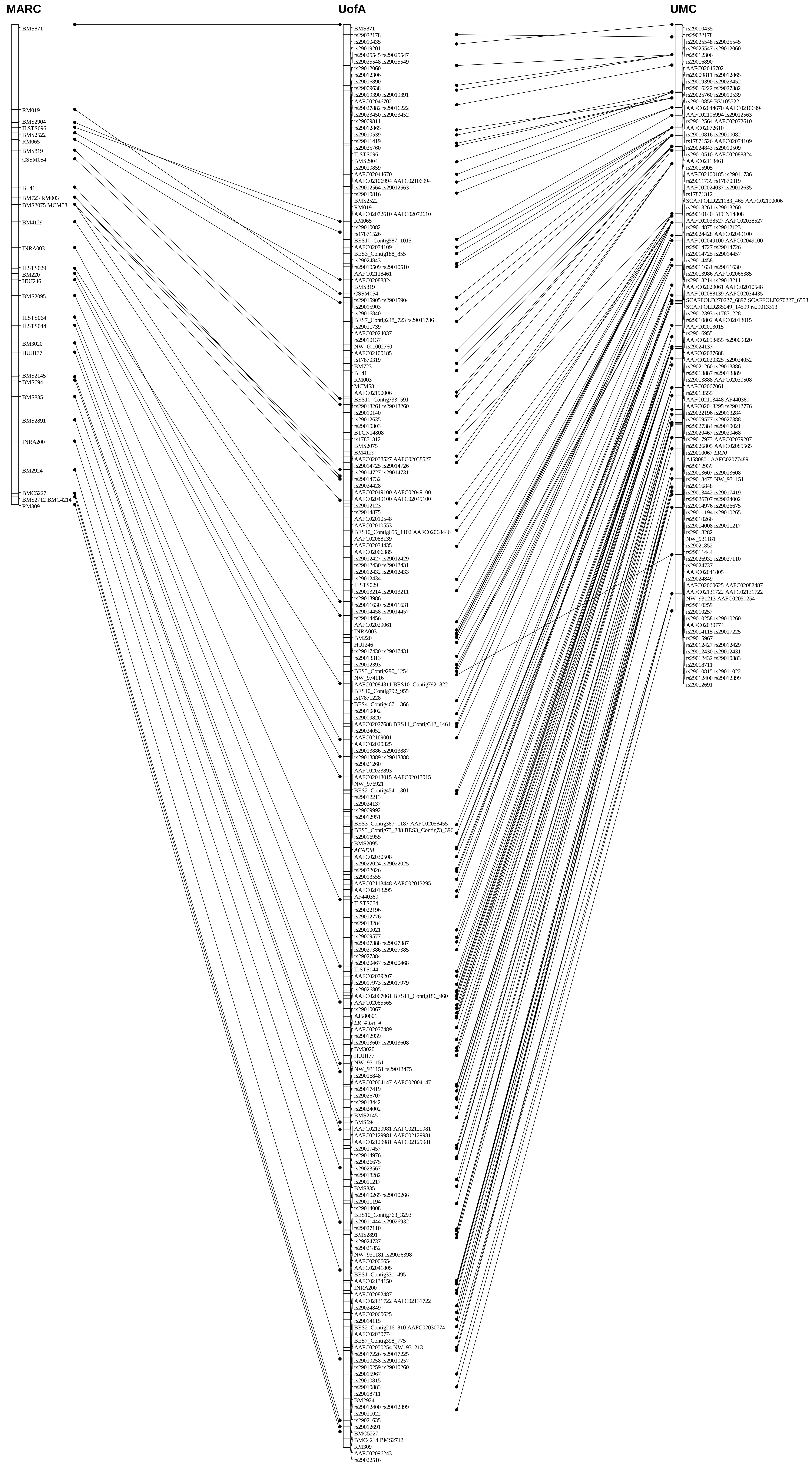
MARC
UofA
UMC
BMS871
RM019
BMS2904
ILSTS096
BMS2522
RM065
BMS819
CSSM054
BL41
BM723 RM003
BMS2075 MCM58
BM4129
INRA003
ILSTS029
BM220
HUJ246
BMS2095
ILSTS064
ILSTS044
BM3020
HUJI177
BMS2145
BMS694
BMS835
BMS2891
INRA200
BM2924
BMC5227
BMS2712 BMC4214
RM309
BMS871
rs29022178
rs29010435
rs29019201
rs29025545 rs29025547
rs29025548 rs29025549
rs29012060
rs29012306
rs29016890
rs29009638
rs29019390 rs29019391
AAFC02046702
rs29027882 rs29016222
rs29023450 rs29023452
rs29009811
rs29012865
rs29010539
rs29011419
rs29025760
ILSTS096
BMS2904
rs29010859
AAFC02044670
AAFC02106994 AAFC02106994
rs29012564 rs29012563
rs29010816
BMS2522
RM019
AAFC02072610 AAFC02072610
RM065
rs29010082
rs17871526
BES10_Contig587_1015
AAFC02074109
BES3_Contig188_855
rs29024843
rs29010509 rs29010510
AAFC02118461
AAFC02088824
BMS819
CSSM054
rs29015905 rs29015904
rs29015903
rs29016840
BES7_Contig248_723 rs29011736
rs29011739
AAFC02024037
rs29010137
NW_001002760
AAFC02100185
rs17870319
BM723
BL41
RM003
MCM58
AAFC02190006
BES10_Contig733_591
rs29013261 rs29013260
rs29010140
rs29012635
rs29010303
BTCN14808
rs17871312
BMS2075
BM4129
AAFC02038527 AAFC02038527
rs29014725 rs29014726
rs29014727 rs29014731
rs29014732
rs29024428
AAFC02049100 AAFC02049100
AAFC02049100 AAFC02049100
rs29012123
rs29014875
AAFC02010548
AAFC02010553
BES10_Contig655_1102 AAFC02068446
AAFC02088139
AAFC02034435
AAFC02066385
rs29012427 rs29012429
rs29012430 rs29012431
rs29012432 rs29012433
rs29012434
ILSTS029
rs29013214 rs29013211
rs29013986
rs29011630 rs29011631
rs29014458 rs29014457
rs29014456
AAFC02029061
INRA003
BM220
HUJ246
rs29017430 rs29017431
rs29013313
rs29012393
BES3_Contig290_1254
NW_974116
AAFC02084311 BES10_Contig792_822
BES10_Contig792_955
rs17871228
BES4_Contig467_1366
rs29010802
rs29009820
AAFC02027688 BES11_Contig312_1461
rs29024052
AAFC02169001
AAFC02020325
rs29013886 rs29013887
rs29013889 rs29013888
rs29021260
AAFC02023893
AAFC02013015 AAFC02013015
NW_976921
BES2_Contig454_1301
rs29012213
rs29024137
rs29009992
rs29012951
BES3_Contig387_1187 AAFC02058455
BES3_Contig73_288 BES3_Contig73_396
rs29016955
BMS2095
ACADM
AAFC02030508
rs29022024 rs29022025
rs29022026
rs29013555
AAFC02113448 AAFC02013295
AAFC02013295
AF440380
ILSTS064
rs29022196
rs29012776
rs29013284
rs29010021
rs29009577
rs29027388 rs29027387
rs29027386 rs29027385
rs29027384
rs29020467 rs29020468
ILSTS044
AAFC02079207
rs29017973 rs29017979
rs29026805
AAFC02067061 BES11_Contig186_960
AAFC02085565
rs29010067
AJ580801
LR_4 LR_4
AAFC02077489
rs29012939
rs29013607 rs29013608
BM3020
HUJI177
NW_931151
NW_931151 rs29013475
rs29016848
AAFC02004147 AAFC02004147
rs29017419
rs29026707
rs29013442
rs29024002
BMS2145
BMS694
AAFC02129981 AAFC02129981
AAFC02129981 AAFC02129981
AAFC02129981 AAFC02129981
rs29017457
rs29014976
rs29026675
rs29023567
rs29018282
rs29011217
BMS835
rs29010265 rs29010266
rs29011194
rs29014008
BES10_Contig763_3293
rs29011444 rs29026932
rs29027110
BMS2891
rs29024737
rs29021852
NW_931181 rs29026398
AAFC02006654
AAFC02041805
BES1_Contig331_495
AAFC02134150
INRA200
AAFC02082487
AAFC02131722 AAFC02131722
rs29024849
AAFC02060625
rs29014115
BES2_Contig216_810 AAFC02030774
AAFC02030774
BES7_Contig398_775
AAFC02050254 NW_931213
rs29017226 rs29017225
rs29010258 rs29010257
rs29010259 rs29010260
rs29015967
rs29010815
rs29010883
rs29018711
BM2924
rs29012400 rs29012399
rs29011022
rs29021635
rs29012691
BMC5227
BMC4214 BMS2712
RM309
AAFC02096243
rs29022516
rs29010435
rs29022178
rs29025548 rs29025545
rs29025547 rs29012060
rs29012306
rs29016890
AAFC02046702
rs29009811 rs29012865
rs29019390 rs29023452
rs29016222 rs29027882
rs29025760 rs29010539
rs29010859 BV105522
AAFC02044670 AAFC02106994
AAFC02106994 rs29012563
rs29012564 AAFC02072610
AAFC02072610
rs29010816 rs29010082
rs17871526 AAFC02074109
rs29024843 rs29010509
rs29010510 AAFC02088824
AAFC02118461
rs29015905
AAFC02100185 rs29011736
rs29011739 rs17870319
AAFC02024037 rs29012635
rs17871312
SCAFFOLD221183_465 AAFC02190006
rs29013261 rs29013260
rs29010140 BTCN14808
AAFC02038527 AAFC02038527
rs29014875 rs29012123
rs29024428 AAFC02049100
AAFC02049100 AAFC02049100
rs29014727 rs29014726
rs29014725 rs29014457
rs29014458
rs29011631 rs29011630
rs29013986 AAFC02066385
rs29013214 rs29013211
AAFC02029061 AAFC02010548
AAFC02088139 AAFC02034435
SCAFFOLD270227_6897 SCAFFOLD270227_6558
SCAFFOLD285049_14599 rs29013313
rs29012393 rs17871228
rs29010802 AAFC02013015
AAFC02013015
rs29016955
AAFC02058455 rs29009820
rs29024137
AAFC02027688
AAFC02020325 rs29024052
rs29021260 rs29013886
rs29013887 rs29013889
rs29013888 AAFC02030508
AAFC02067061
rs29013555
AAFC02113448 AF440380
AAFC02013295 rs29012776
rs29022196 rs29013284
rs29009577 rs29027388
rs29027384 rs29010021
rs29020467 rs29020468
rs29017973 AAFC02079207
rs29026805 AAFC02085565
rs29010067 LR20
AJ580801 AAFC02077489
rs29012939
rs29013607 rs29013608
rs29016848
rs29013475 NW_931151
rs29013442 rs29017419
rs29026707 rs29024002
rs29014976 rs29026675
rs29011194 rs29010265
rs29010266
rs29014008 rs29011217
rs29018282
NW_931181
rs29021852
rs29011444
rs29026932 rs29027110
rs29024737
AAFC02041805
rs29024849
AAFC02060625 AAFC02082487
AAFC02131722 AAFC02131722
NW_931213 AAFC02050254
rs29010259
rs29010257
rs29010258 rs29010260
AAFC02030774
rs29014115 rs29017225
rs29015967
rs29012427 rs29012429
rs29012430 rs29012431
rs29012432 rs29010883
rs29018711
rs29010815 rs29011022
rs29012400 rs29012399
rs29012691

BTA3

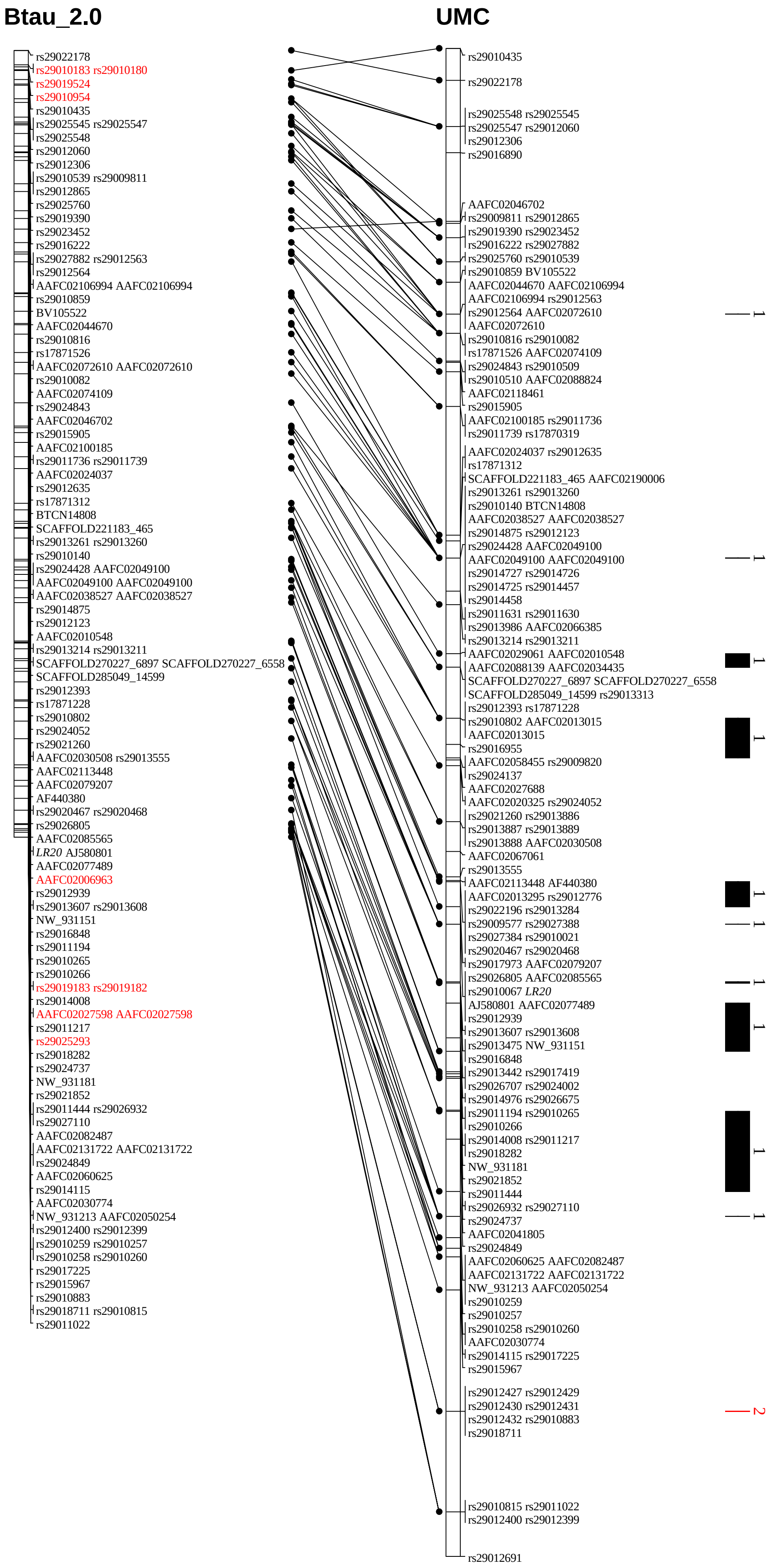
Btau_2.0
UMC
rs29022178
rs29010183 rs29010180
rs29019524
rs29010954
rs29010435
rs29025545 rs29025547
rs29025548
rs29012060
rs29012306
rs29010539 rs29009811
rs29012865
rs29025760
rs29019390
rs29023452
rs29016222
rs29027882 rs29012563
rs29012564
AAFC02106994 AAFC02106994
rs29010859
BV105522
AAFC02044670
rs29010816
rs17871526
AAFC02072610 AAFC02072610
rs29010082
AAFC02074109
rs29024843
AAFC02046702
rs29015905
AAFC02100185
rs29011736 rs29011739
AAFC02024037
rs29012635
rs17871312
BTCN14808
SCAFFOLD221183_465
rs29013261 rs29013260
rs29010140
rs29024428 AAFC02049100
AAFC02049100 AAFC02049100
AAFC02038527 AAFC02038527
rs29014875
rs29012123
AAFC02010548
rs29013214 rs29013211
SCAFFOLD270227_6897 SCAFFOLD270227_6558
SCAFFOLD285049_14599
rs29012393
rs17871228
rs29010802
rs29024052
rs29021260
AAFC02030508 rs29013555
AAFC02113448
AAFC02079207
AF440380
rs29020467 rs29020468
rs29026805
AAFC02085565
LR20 AJ580801
AAFC02077489
AAFC02006963
rs29012939
rs29013607 rs29013608
NW_931151
rs29016848
rs29011194
rs29010265
rs29010266
rs29019183 rs29019182
rs29014008
AAFC02027598 AAFC02027598
rs29011217
rs29025293
rs29018282
rs29024737
NW_931181
rs29021852
rs29011444 rs29026932
rs29027110
AAFC02082487
AAFC02131722 AAFC02131722
rs29024849
AAFC02060625
rs29014115
AAFC02030774
NW_931213 AAFC02050254
rs29012400 rs29012399
rs29010259 rs29010257
rs29010258 rs29010260
rs29017225
rs29015967
rs29010883
rs29018711 rs29010815
rs29011022
rs29010435
rs29022178
rs29025548 rs29025545
rs29025547 rs29012060
rs29012306
rs29016890
AAFC02046702
rs29009811 rs29012865
rs29019390 rs29023452
rs29016222 rs29027882
rs29025760 rs29010539
rs29010859 BV105522
AAFC02044670 AAFC02106994
AAFC02106994 rs29012563
rs29012564 AAFC02072610
AAFC02072610
rs29010816 rs29010082
rs17871526 AAFC02074109
rs29024843 rs29010509
rs29010510 AAFC02088824
AAFC02118461
rs29015905
AAFC02100185 rs29011736
rs29011739 rs17870319
AAFC02024037 rs29012635
rs17871312
SCAFFOLD221183_465 AAFC02190006
rs29013261 rs29013260
rs29010140 BTCN14808
AAFC02038527 AAFC02038527
rs29014875 rs29012123
rs29024428 AAFC02049100
AAFC02049100 AAFC02049100
rs29014727 rs29014726
rs29014725 rs29014457
rs29014458
rs29011631 rs29011630
rs29013986 AAFC02066385
rs29013214 rs29013211
AAFC02029061 AAFC02010548
AAFC02088139 AAFC02034435
SCAFFOLD270227_6897 SCAFFOLD270227_6558
SCAFFOLD285049_14599 rs29013313
rs29012393 rs17871228
rs29010802 AAFC02013015
AAFC02013015
rs29016955
AAFC02058455 rs29009820
rs29024137
AAFC02027688
AAFC02020325 rs29024052
rs29021260 rs29013886
rs29013887 rs29013889
rs29013888 AAFC02030508
AAFC02067061
rs29013555
AAFC02113448 AF440380
AAFC02013295 rs29012776
rs29022196 rs29013284
rs29009577 rs29027388
rs29027384 rs29010021
rs29020467 rs29020468
rs29017973 AAFC02079207
rs29026805 AAFC02085565
rs29010067 *LR20*
AJ580801 AAFC02077489
rs29012939
rs29013607 rs29013608
rs29013475 NW_931151
rs29016848
rs29013442 rs29017419
rs29026707 rs29024002
rs29014976 rs29026675
rs29011194 rs29010265
rs29010266
rs29014008 rs29011217
rs29018282
NW_931181
rs29021852
rs29011444
rs29026932 rs29027110
rs29024737
AAFC02041805
rs29024849
AAFC02060625 AAFC02082487
AAFC02131722 AAFC02131722
NW_931213 AAFC02050254
rs29010259
rs29010257
rs29010258 rs29010260
AAFC02030774
rs29014115 rs29017225
rs29015967
rs29012427 rs29012429
rs29012430 rs29012431
rs29012432 rs29010883
rs29018711
rs29010815 rs29011022
rs29012400 rs29012399
rs29012691
1
1
1
1
1
1
1
1
1
1
2

BTA4

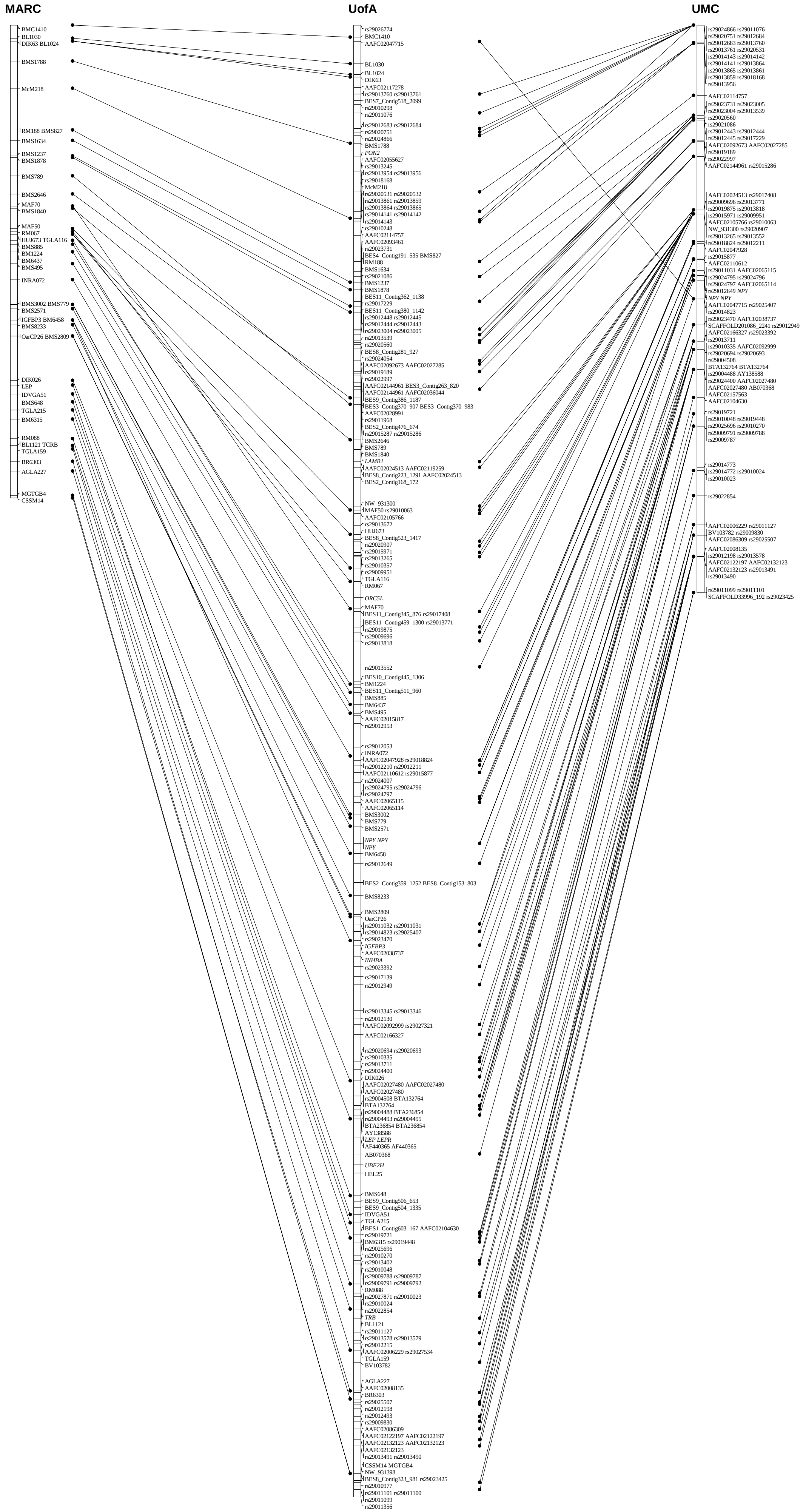
MARC
UofA
UMC
BMC1410
BL1030
DIK63 BL1024
BMS1788
McM218
RM188 BMS827
BMS1634
BMS1237
BMS1878
BMS789
BMS2646
MAF70
BMS1840
MAF50
RM067
HUJ673 TGLA116
BMS885
BM1224
BM6437
BMS495
INRA072
BMS3002 BMS779
BMS2571
IGFBP3 BM6458
BMS8233
OarCP26 BMS2809
DIK026
LEP
IDVGA51
BMS648
TGLA215
BM6315
RM088
BL1121 TCRB
TGLA159
BR6303
AGLA227
MGTGB4
CSSM14
rs29026774
BMC1410
AAFC02047715
BL1030
BL1024
DIK63
AAFC02117278
rs29013760 rs29013761
BES7_Contig518_2099
rs29010298
rs29011076
rs29012683 rs29012684
rs29020751
rs29024866
BMS1788
PON2
AAFC02055627
rs29013245
rs29013954 rs29013956
rs29018168
McM218
rs29020531 rs29020532
rs29013861 rs29013859
rs29013864 rs29013865
rs29014141 rs29014142
rs29014143
rs29010248
AAFC02114757
AAFC02093461
rs29023731
BES4_Contig191_535 BMS827
RM188
BMS1634
rs29021086
BMS1237
BMS1878
BES11_Contig362_1138
rs29017229
BES11_Contig380_1142
rs29012448 rs29012445
rs29012444 rs29012443
rs29023004 rs29023005
rs29013539
rs29020560
BES8_Contig281_927
rs29024054
AAFC02092673 AAFC02027285
rs29019189
rs29022997
AAFC02144961 BES3_Contig263_820
AAFC02144961 AAFC02036044
BES9_Contig386_1187
BES3_Contig370_907 BES3_Contig370_983
AAFC02028991
rs29011968
BES2_Contig476_674
rs29015287 rs29015286
BMS2646
BMS789
BMS1840
LAMB1
AAFC02024513 AAFC02119259
BES8_Contig223_1291 AAFC02024513
BES2_Contig168_172
NW_931300
MAF50 rs29010063
AAFC02105766
rs29013672
HUJ673
BES8_Contig523_1417
rs29020907
rs29015971
rs29013265
rs29010357
rs29009951
TGLA116
RM067
ORC5L
MAF70
BES11_Contig345_876 rs29017408
BES11_Contig459_1300 rs29013771
rs29019875
rs29009696
rs29013818
rs29013552
BES10_Contig445_1306
BM1224
BES11_Contig511_960
BMS885
BM6437
BMS495
AAFC02015817
rs29012953
rs29012053
INRA072
AAFC02047928 rs29018824
rs29012210 rs29012211
AAFC02110612 rs29015877
rs29024007
rs29024795 rs29024796
rs29024797
AAFC02065115
AAFC02065114
BMS3002
BMS779
BMS2571
NPY NPY
NPY
BM6458
rs29012649
BES2_Contig359_1252 BES8_Contig153_803
BMS8233
BMS2809
OarCP26
rs29011032 rs29011031
rs29014823 rs29025407
rs29023470
IGFBP3
AAFC02038737
INHBA
rs29023392
rs29017139
rs29012949
rs29013345 rs29013346
rs29012130
AAFC02092999 rs29027321
AAFC02166327
rs29020694 rs29020693
rs29010335
rs29013711
rs29024400
DIK026
AAFC02027480 AAFC02027480
AAFC02027480
rs29004508 BTA132764
BTA132764
rs29004488 BTA236854
rs29004493 rs29004495
BTA236854 BTA236854
AY138588
LEP LEPR
AF440365 AF440365
AB070368
UBE2H
HEL25
BMS648
BES9_Contig506_653
BES9_Contig504_1335
IDVGA51
TGLA215
BES1_Contig603_167 AAFC02104630
rs29019721
BM6315 rs29019448
rs29025696
rs29010270
rs29013402
rs29010048
rs29009788 rs29009787
rs29009791 rs29009792
RM088
rs29027871 rs29010023
rs29010024
rs29022854
TRB
BL1121
rs29011127
rs29013578 rs29013579
rs29012215
AAFC02006229 rs29027534
TGLA159
BV103782
AGLA227
AAFC02008135
BR6303
rs29025507
rs29012198
rs29012493
rs29009830
AAFC02086309
AAFC02122197 AAFC02122197
AAFC02132123 AAFC02132123
AAFC02132123
rs29013491 rs29013490
CSSM14 MGTGB4
NW_931398
BES8_Contig323_981 rs29023425
rs29010977
rs29011101 rs29011100
rs29011099
rs29011356
rs29024866 rs29011076
rs29020751 rs29012684
rs29012683 rs29013760
rs29013761 rs29020531
rs29014143 rs29014142
rs29014141 rs29013864
rs29013865 rs29013861
rs29013859 rs29018168
rs29013956
AAFC02114757
rs29023731 rs29023005
rs29023004 rs29013539
rs29020560
rs29021086
rs29012443 rs29012444
rs29012445 rs29017229
AAFC02092673 AAFC02027285
rs29019189
rs29022997
AAFC02144961 rs29015286
AAFC02024513 rs29017408
rs29009696 rs29013771
rs29019875 rs29013818
rs29015971 rs29009951
AAFC02105766 rs29010063
NW_931300 rs29020907
rs29013265 rs29013552
rs29018824 rs29012211
AAFC02047928
rs29015877
AAFC02110612
rs29011031 AAFC02065115
rs29024795 rs29024796
rs29024797 AAFC02065114
rs29012649 NPY
NPY NPY
AAFC02047715 rs29025407
rs29014823
rs29023470 AAFC02038737
SCAFFOLD201086_2241 rs29012949
AAFC02166327 rs29023392
rs29013711
rs29010335 AAFC02092999
rs29020694 rs29020693
rs29004508
BTA132764 BTA132764
rs29004488 AY138588
rs29024400 AAFC02027480
AAFC02027480 AB070368
AAFC02157563
AAFC02104630
rs29019721
rs29010048 rs29019448
rs29025696 rs29010270
rs29009791 rs29009788
rs29009787
rs29014773
rs29014772 rs29010024
rs29010023
rs29022854
AAFC02006229 rs29011127
BV103782 rs29009830
AAFC02086309 rs29025507
AAFC02008135
rs29012198 rs29013578
AAFC02122197 AAFC02132123
AAFC02132123 rs29013491
rs29013490
rs29011099 rs29011101
SCAFFOLD33996_192 rs29023425

BTA4

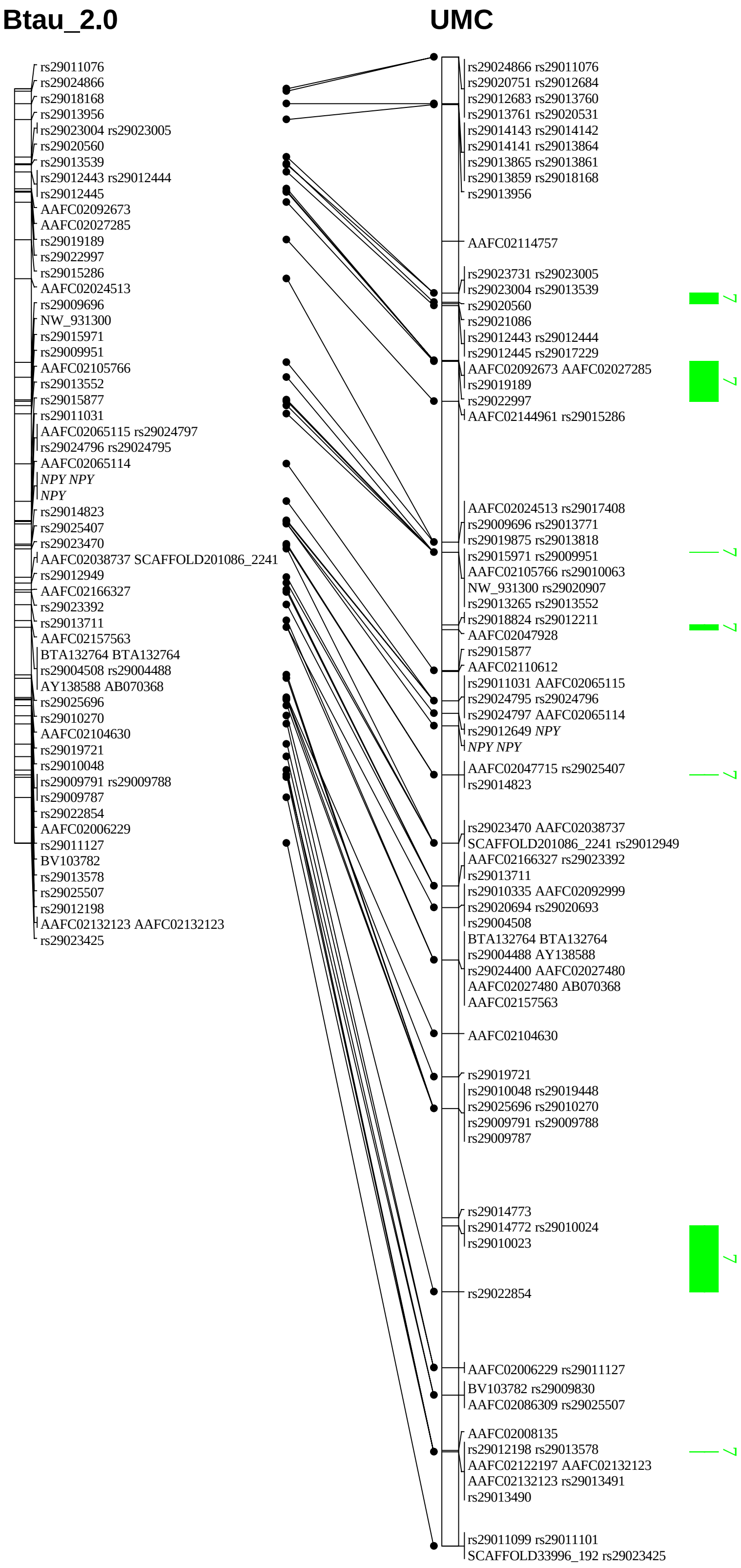
Btau_2.0
UMC
rs29011076
rs29024866
rs29018168
rs29013956
rs29023004 rs29023005
rs29020560
rs29013539
rs29012443 rs29012444
rs29012445
AAFC02092673
AAFC02027285
rs29019189
rs29022997
rs29015286
AAFC02024513
rs29009696
NW_931300
rs29015971
rs29009951
AAFC02105766
rs29013552
rs29015877
rs29011031
AAFC02065115 rs29024797
rs29024796 rs29024795
AAFC02065114
NPY NPY
NPY
rs29014823
rs29025407
rs29023470
AAFC02038737 SCAFFOLD201086_2241
rs29012949
AAFC02166327
rs29023392
rs29013711
AAFC02157563
BTA132764 BTA132764
rs29004508 rs29004488
AY138588 AB070368
rs29025696
rs29010270
AAFC02104630
rs29019721
rs29010048
rs29009791 rs29009788
rs29009787
rs29022854
AAFC02006229
rs29011127
BV103782
rs29013578
rs29025507
rs29012198
AAFC02132123 AAFC02132123
rs29023425
rs29024866 rs29011076
rs29020751 rs29012684
rs29012683 rs29013760
rs29013761 rs29020531
rs29014143 rs29014142
rs29014141 rs29013864
rs29013865 rs29013861
rs29013859 rs29018168
rs29013956
AAFC02114757
rs29023731 rs29023005
rs29023004 rs29013539
rs29020560
rs29021086
rs29012443 rs29012444
rs29012445 rs29017229
AAFC02092673 AAFC02027285
rs29019189
rs29022997
AAFC02144961 rs29015286
AAFC02024513 rs29017408
rs29009696 rs29013771
rs29019875 rs29013818
rs29015971 rs29009951
AAFC02105766 rs29010063
NW_931300 rs29020907
rs29013265 rs29013552
rs29018824 rs29012211
AAFC02047928
rs29015877
AAFC02110612
rs29011031 AAFC02065115
rs29024795 rs29024796
rs29024797 AAFC02065114
rs29012649 NPY
NPY NPY
AAFC02047715 rs29025407
rs29014823
rs29023470 AAFC02038737
SCAFFOLD201086_2241 rs29012949
AAFC02166327 rs29023392
rs29013711
rs29010335 AAFC02092999
rs29020694 rs29020693
rs29004508
BTA132764 BTA132764
rs29004488 AY138588
rs29024400 AAFC02027480
AAFC02027480 AB070368
AAFC02157563
AAFC02104630
rs29019721
rs29010048 rs29019448
rs29025696 rs29010270
rs29009791 rs29009788
rs29009787
rs29014773
rs29014772 rs29010024
rs29010023
rs29022854
AAFC02006229 rs29011127
BV103782 rs29009830
AAFC02086309 rs29025507
AAFC02008135
rs29012198 rs29013578
AAFC02122197 AAFC02132123
AAFC02132123 rs29013491
rs29013490
rs29011099 rs29011101
SCAFFOLD33996_192 rs29023425

BTA5

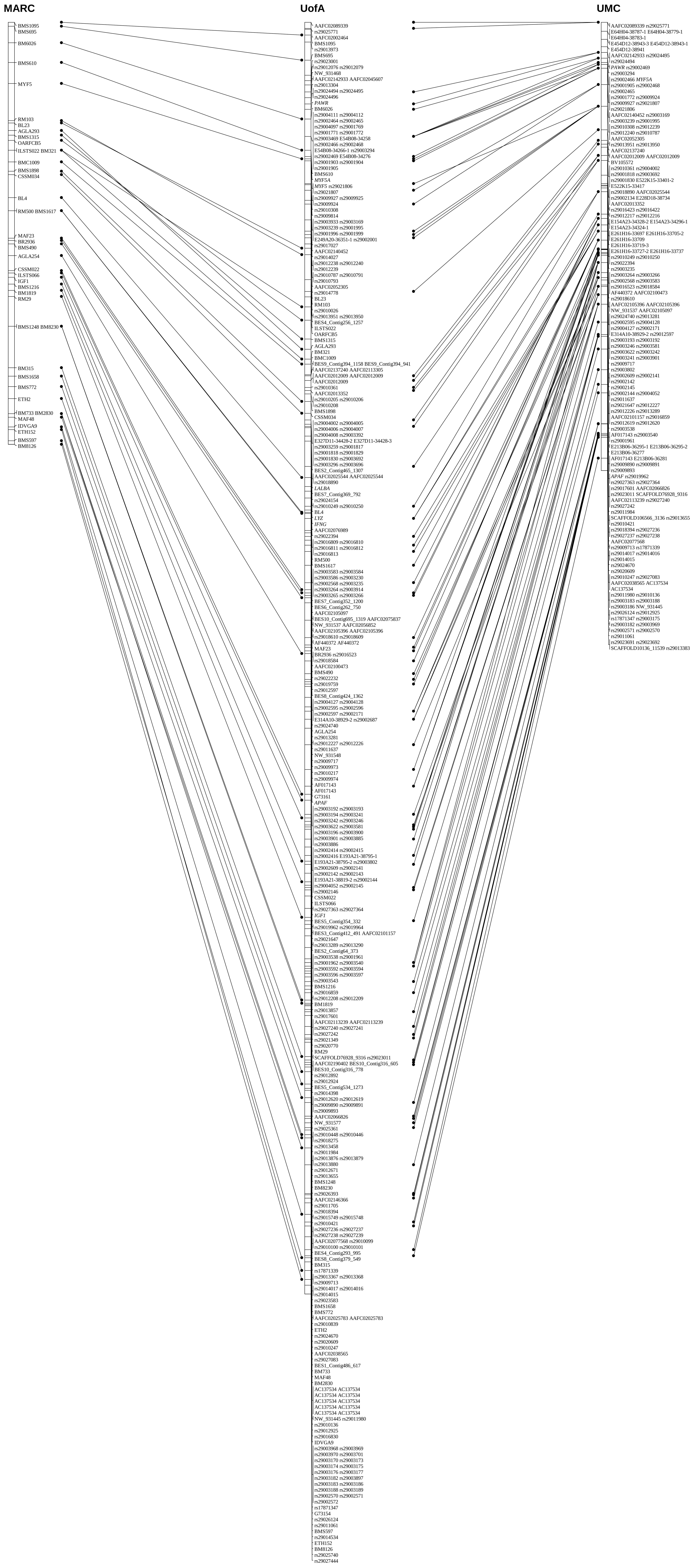
MARC
UofA
UMC
BMS1095
BMS695
BM6026
BMS610
MYF5
RM103
BL23
AGLA293
BMS1315
OARFCB5
ILSTS022 BM321
BMC1009
BMS1898
CSSM034
BL4
RM500 BMS1617
MAF23
BR2936
BMS490
AGLA254
CSSM022
ILSTS066
IGF1
BMS1216
BM1819
RM29
BMS1248 BM8230
BM315
BMS1658
BMS772
ETH2
BM733 BM2830
MAF48
IDVGA9
ETH152
BMS597
BM8126

BTA5

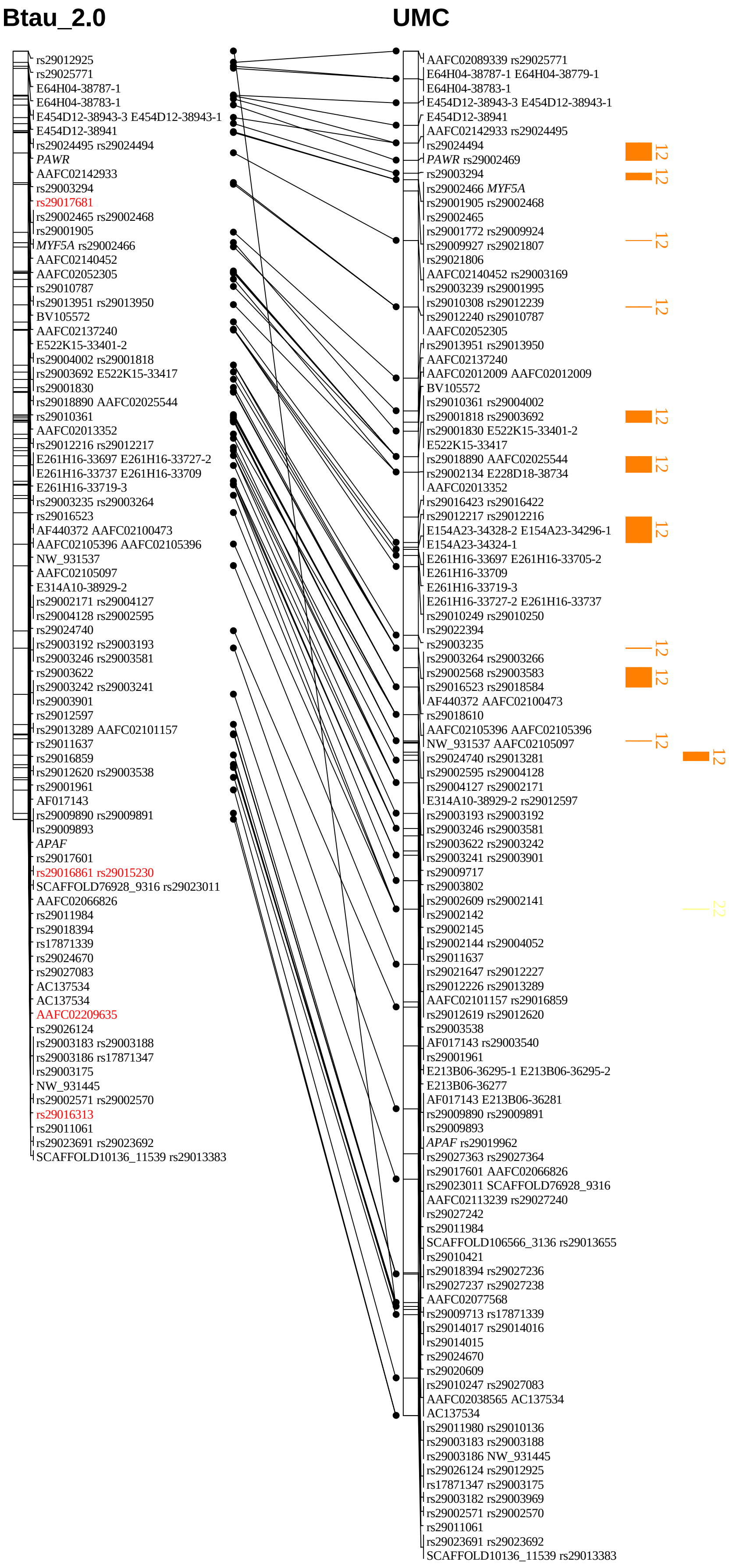

Btau_2.0
UMC
rs29012925
rs29025771
E64H04-38787-1
E64H04-38783-1
E454D12-38943-3 E454D12-38943-1
E454D12-38941
rs29024495 rs29024494
PAWR
AAFC02142933
rs29003294
rs29017681
rs29002465 rs29002468
rs29001905
MYF5A rs29002466
AAFC02140452
AAFC02052305
rs29010787
rs29013951 rs29013950
BV105572
AAFC02137240
E522K15-33401-2
rs29004002 rs29001818
rs29003692 E522K15-33417
rs29001830
rs29018890 AAFC02025544
rs29010361
AAFC02013352
rs29012216 rs29012217
E261H16-33697 E261H16-33727-2
E261H16-33737 E261H16-33709
E261H16-33719-3
rs29003235 rs29003264
rs29016523
AF440372 AAFC02100473
AAFC02105396 AAFC02105396
NW_931537
AAFC02105097
E314A10-38929-2
rs29002171 rs29004127
rs29004128 rs29002595
rs29024740
rs29003192 rs29003193
rs29003246 rs29003581
rs29003622
rs29003242 rs29003241
rs29003901
rs29012597
rs29013289 AAFC02101157
rs29011637
rs29016859
rs29012620 rs29003538
rs29001961
AF017143
rs29009890 rs29009891
rs29009893
APAF
rs29017601
rs29016861 rs29015230
SCAFFOLD76928_9316 rs29023011
AAFC02066826
rs29011984
rs29018394
rs17871339
rs29024670
rs29027083
AC137534
AC137534
AAFC02209635
rs29026124
rs29003183 rs29003188
rs29003186 rs17871347
rs29003175
NW_931445
rs29002571 rs29002570
rs29016313
rs29011061
rs29023691 rs29023692
SCAFFOLD10136_11539 rs29013383
AAFC02089339 rs29025771
E64H04-38787-1 E64H04-38779-1
E64H04-38783-1
E454D12-38943-3 E454D12-38943-1
E454D12-38941
AAFC02142933 rs29024495
rs29024494
PAWR rs29002469
rs29003294
rs29002466 MYF5A
rs29001905 rs29002468
rs29002465
rs29001772 rs29009924
rs29009927 rs29021807
rs29021806
AAFC02140452 rs29003169
rs29003239 rs29001995
rs29010308 rs29012239
rs29012240 rs29010787
AAFC02052305
rs29013951 rs29013950
AAFC02137240
AAFC02012009 AAFC02012009
BV105572
rs29010361 rs29004002
rs29001818 rs29003692
rs29001830 E522K15-33401-2
E522K15-33417
rs29018890 AAFC02025544
rs29002134 E228D18-38734
AAFC02013352
rs29016423 rs29016422
rs29012217 rs29012216
E154A23-34328-2 E154A23-34296-1
E154A23-34324-1
E261H16-33697 E261H16-33705-2
E261H16-33709
E261H16-33719-3
E261H16-33727-2 E261H16-33737
rs29010249 rs29010250
rs29022394
rs29003235
rs29003264 rs29003266
rs29002568 rs29003583
rs29016523 rs29018584
AF440372 AAFC02100473
rs29018610
AAFC02105396 AAFC02105396
NW_931537 AAFC02105097
rs29024740 rs29013281
rs29002595 rs29004128
rs29004127 rs29002171
E314A10-38929-2 rs29012597
rs29003193 rs29003192
rs29003246 rs29003581
rs29003622 rs29003242
rs29003241 rs29003901
rs29009717
rs29003802
rs29002609 rs29002141
rs29002142
rs29002145
rs29002144 rs29004052
rs29011637
rs29021647 rs29012227
rs29012226 rs29013289
AAFC02101157 rs29016859
rs29012619 rs29012620
rs29003538
AF017143 rs29003540
rs29001961
E213B06-36295-1 E213B06-36295-2
E213B06-36277
AF017143 E213B06-36281
rs29009890 rs29009891
rs29009893
APAF rs29019962
rs29027363 rs29027364
rs29017601 AAFC02066826
rs29023011 SCAFFOLD76928_9316
AAFC02113239 rs29027240
rs29027242
rs29011984
SCAFFOLD106566_3136 rs29013655
rs29010421
rs29018394 rs29027236
rs29027237 rs29027238
AAFC02077568
rs29009713 rs17871339
rs29014017 rs29014016
rs29014015
rs29024670
rs29020609
rs29010247 rs29027083
AAFC02038565 AC137534
AC137534
rs29011980 rs29010136
rs29003183 rs29003188
rs29003186 NW_931445
rs29026124 rs29012925
rs17871347 rs29003175
rs29003182 rs29003969
rs29002571 rs29002570
rs29011061
rs29023691 rs29023692
SCAFFOLD10136_11539 rs29013383
12
12
12
12
12
12
12
12
12
12
12

BTA6

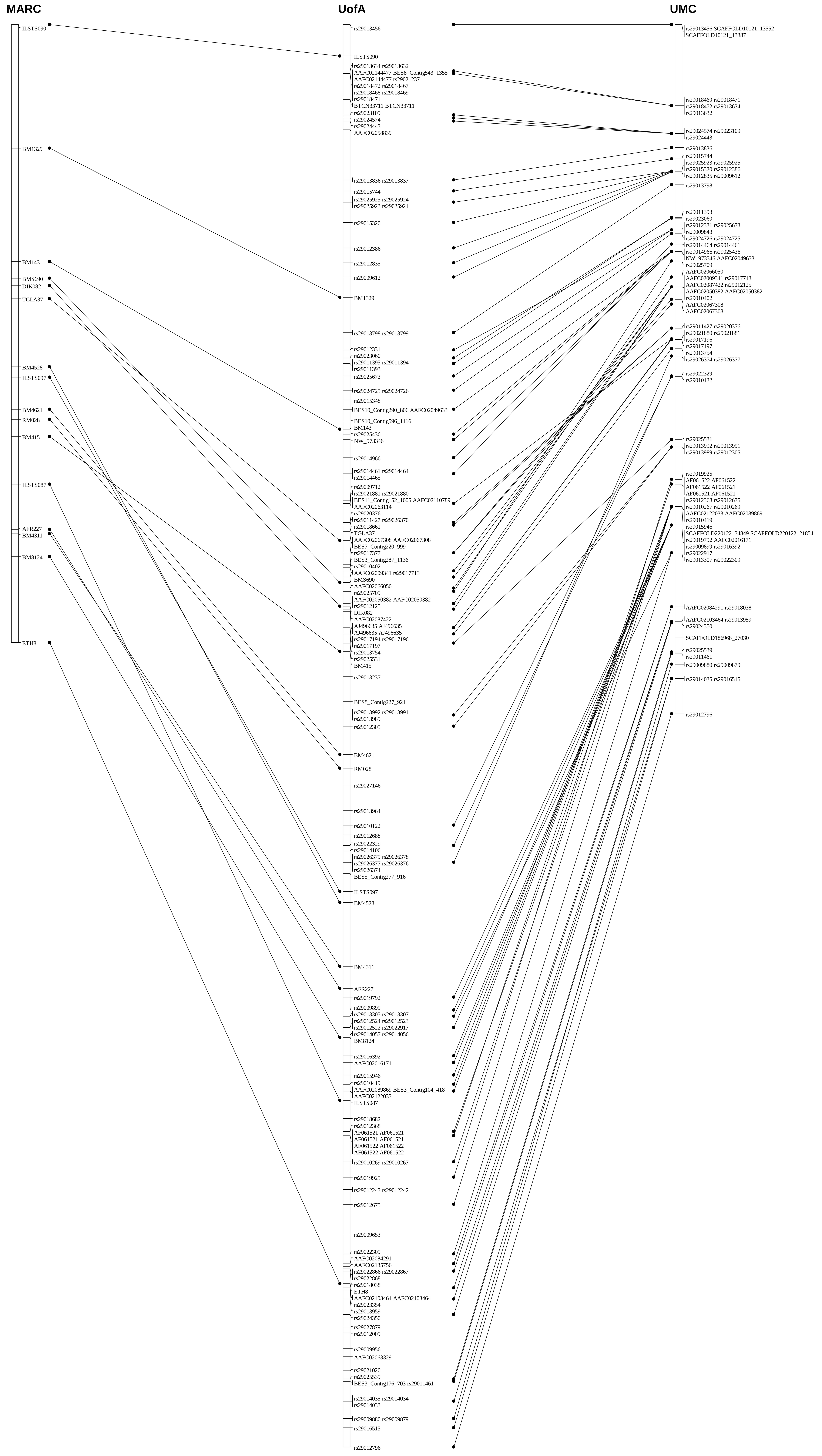
MARC
UofA
UMC
ILSTS090
BM1329
BM143
BMS690
DIK082
TGLA37
BM4528
ILSTS097
BM4621
RM028
BM415
ILSTS087
AFR227
BM4311
BM8124
ETH8
rs29013456
ILSTS090
rs29013634 rs29013632
AAFC02144477 BES8_Contig543_1355
AAFC02144477 rs29021237
rs29018472 rs29018467
rs29018468 rs29018469
rs29018471
BTCN33711 BTCN33711
rs29023109
rs29024574
rs29024443
AAFC02058839
rs29013836 rs29013837
rs29015744
rs29025925 rs29025924
rs29025923 rs29025921
rs29015320
rs29012386
rs29012835
rs29009612
BM1329
rs29013798 rs29013799
rs29012331
rs29023060
rs29011395 rs29011394
rs29011393
rs29025673
rs29024725 rs29024726
rs29015348
BES10_Contig290_806 AAFC02049633
BES10_Contig596_1116
BM143
rs29025436
NW_973346
rs29014966
rs29014461 rs29014464
rs29014465
rs29009712
rs29021881 rs29021880
BES11_Contig152_1005 AAFC02110789
AAFC02063114
rs29020376
rs29011427 rs29026370
rs29018661
TGLA37
AAFC02067308 AAFC02067308
BES7_Contig220_999
rs29017377
BES3_Contig287_1136
rs29010402
AAFC02009341 rs29017713
BMS690
AAFC02066050
rs29025709
AAFC02050382 AAFC02050382
rs29012125
DIK082
AAFC02087422
AJ496635 AJ496635
AJ496635 AJ496635
rs29017194 rs29017196
rs29017197
rs29013754
rs29025531
BM415
rs29013237
BES8_Contig227_921
rs29013992 rs29013991
rs29013989
rs29012305
BM4621
RM028
rs29027146
rs29013964
rs29010122
rs29012688
rs29022329
rs29014106
rs29026379 rs29026378
rs29026377 rs29026376
rs29026374
BES5_Contig277_916
ILSTS097
BM4528
BM4311
AFR227
rs29019792
rs29009899
rs29013305 rs29013307
rs29012524 rs29012523
rs29012522 rs29022917
rs29014057 rs29014056
BM8124
rs29016392
AAFC02016171
rs29015946
rs29010419
AAFC02089869 BES3_Contig104_418
AAFC02122033
ILSTS087
rs29018682
rs29012368
AF061521 AF061521
AF061521 AF061521
AF061522 AF061522
AF061522 AF061522
rs29010269 rs29010267
rs29019925
rs29012243 rs29012242
rs29012675
rs29009653
rs29022309
AAFC02084291
AAFC02135756
rs29022866 rs29022867
rs29022868
rs29018038
ETH8
AAFC02103464 AAFC02103464
rs29023354
rs29013959
rs29024350
rs29027879
rs29012009
rs29009956
AAFC02063329
rs29021020
rs29025539
BES3_Contig176_703 rs29011461
rs29014035 rs29014034
rs29014033
rs29009880 rs29009879
rs29016515
rs29012796
rs29013456 SCAFFOLD10121_13552
SCAFFOLD10121_13387
rs29018469 rs29018471
rs29018472 rs29013634
rs29013632
rs29024574 rs29023109
rs29024443
rs29013836
rs29015744
rs29025923 rs29025925
rs29015320 rs29012386
rs29012835 rs29009612
rs29013798
rs29011393
rs29023060
rs29012331 rs29025673
rs29009843
rs29024726 rs29024725
rs29014464 rs29014461
rs29014966 rs29025436
NW_973346 AAFC02049633
rs29025709
AAFC02066050
AAFC02009341 rs29017713
AAFC02087422 rs29012125
AAFC02050382 AAFC02050382
rs29010402
AAFC02067308
AAFC02067308
rs29011427 rs29020376
rs29021880 rs29021881
rs29017196
rs29017197
rs29013754
rs29026374 rs29026377
rs29022329
rs29010122
rs29025531
rs29013992 rs29013991
rs29013989 rs29012305
rs29019925
AF061522 AF061522
AF061522 AF061521
AF061521 AF061521
rs29012368 rs29012675
rs29010267 rs29010269
AAFC02122033 AAFC02089869
rs29010419
rs29015946
SCAFFOLD220122_34849 SCAFFOLD220122_21854
rs29019792 AAFC02016171
rs29009899 rs29016392
rs29022917
rs29013307 rs29022309
AAFC02084291 rs29018038
AAFC02103464 rs29013959
rs29024350
SCAFFOLD186968_27030
rs29025539
rs29011461
rs29009880 rs29009879
rs29014035 rs29016515
rs29012796

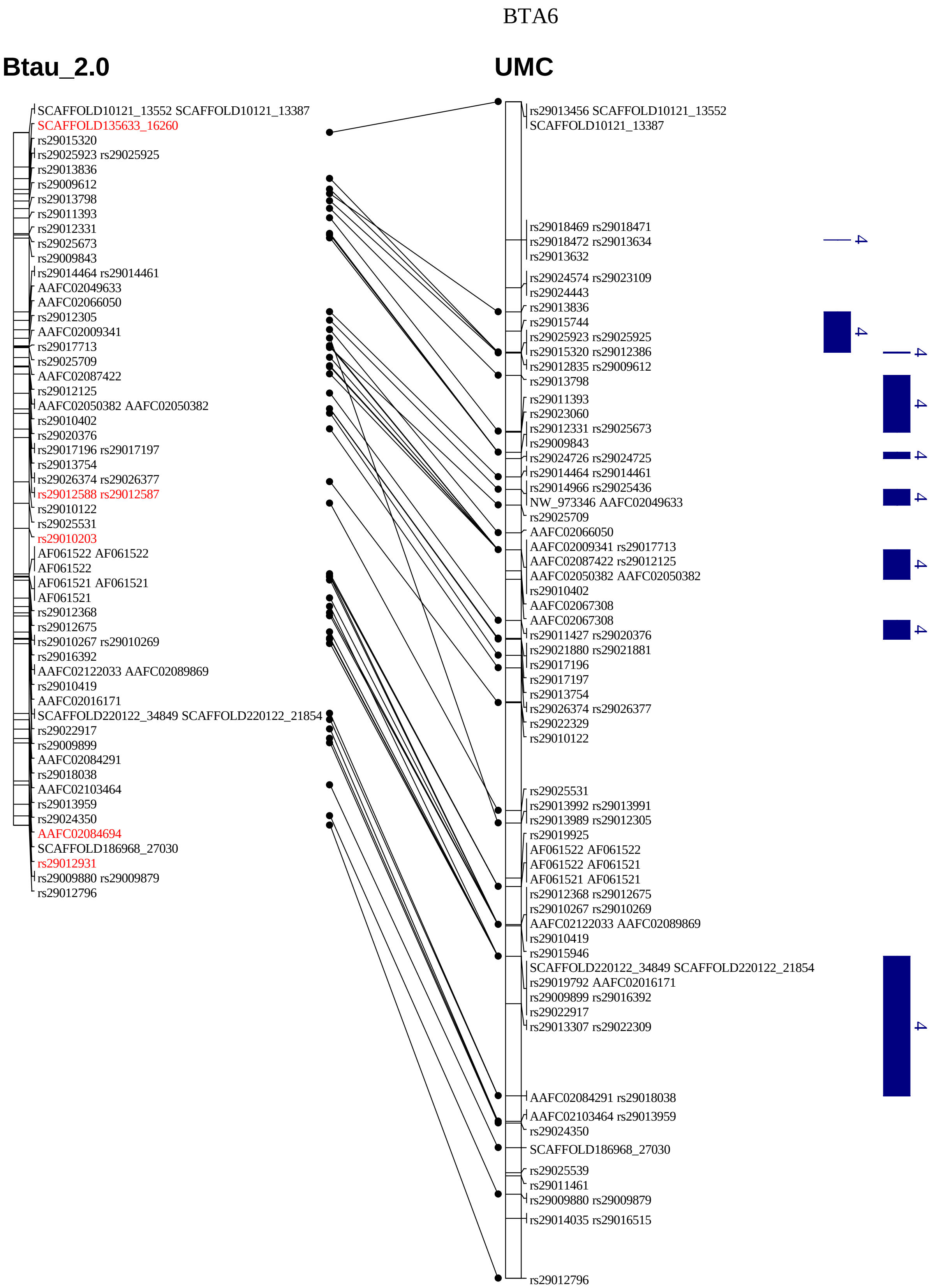

BTA6
Btau_2.0
UMC
SCAFFOLD10121_13552 SCAFFOLD10121_13387
SCAFFOLD135633_16260
rs29015320
rs29025923 rs29025925
rs29013836
rs29009612
rs29013798
rs29011393
rs29012331
rs29025673
rs29009843
rs29014464 rs29014461
AAFC02049633
AAFC02066050
rs29012305
AAFC02009341
rs29017713
rs29025709
AAFC02087422
rs29012125
AAFC02050382 AAFC02050382
rs29010402
rs29020376
rs29017196 rs29017197
rs29013754
rs29026374 rs29026377
rs29012588 rs29012587
rs29010122
rs29025531
rs29010203
AF061522 AF061522
AF061522
AF061521 AF061521
AF061521
rs29012368
rs29012675
rs29010267 rs29010269
rs29016392
AAFC02122033 AAFC02089869
rs29010419
AAFC02016171
SCAFFOLD220122_34849 SCAFFOLD220122_21854
rs29022917
rs29009899
AAFC02084291
rs29018038
AAFC02103464
rs29013959
rs29024350
AAFC02084694
SCAFFOLD186968_27030
rs29012931
rs29009880 rs29009879
rs29012796
rs29013456 SCAFFOLD10121_13552
SCAFFOLD10121_13387
rs29018469 rs29018471
rs29018472 rs29013634
rs29013632
rs29024574 rs29023109
rs29024443
rs29013836
rs29015744
rs29025923 rs29025925
rs29015320 rs29012386
rs29012835 rs29009612
rs29013798
rs29011393
rs29023060
rs29012331 rs29025673
rs29009843
rs29024726 rs29024725
rs29014464 rs29014461
rs29014966 rs29025436
NW_973346 AAFC02049633
rs29025709
AAFC02066050
AAFC02009341 rs29017713
AAFC02087422 rs29012125
AAFC02050382 AAFC02050382
rs29010402
AAFC02067308
AAFC02067308
rs29011427 rs29020376
rs29021880 rs29021881
rs29017196
rs29017197
rs29013754
rs29026374 rs29026377
rs29022329
rs29010122
rs29025531
rs29013992 rs29013991
rs29013989 rs29012305
rs29019925
AF061522 AF061522
AF061522 AF061521
AF061521 AF061521
rs29012368 rs29012675
rs29010267 rs29010269
AAFC02122033 AAFC02089869
rs29010419
rs29015946
SCAFFOLD220122_34849 SCAFFOLD220122_21854
rs29019792 AAFC02016171
rs29009899 rs29016392
rs29022917
rs29013307 rs29022309
AAFC02084291 rs29018038
AAFC02103464 rs29013959
rs29024350
SCAFFOLD186968_27030
rs29025539
rs29011461
rs29009880 rs29009879
rs29014035 rs29016515
rs29012796

BTA7

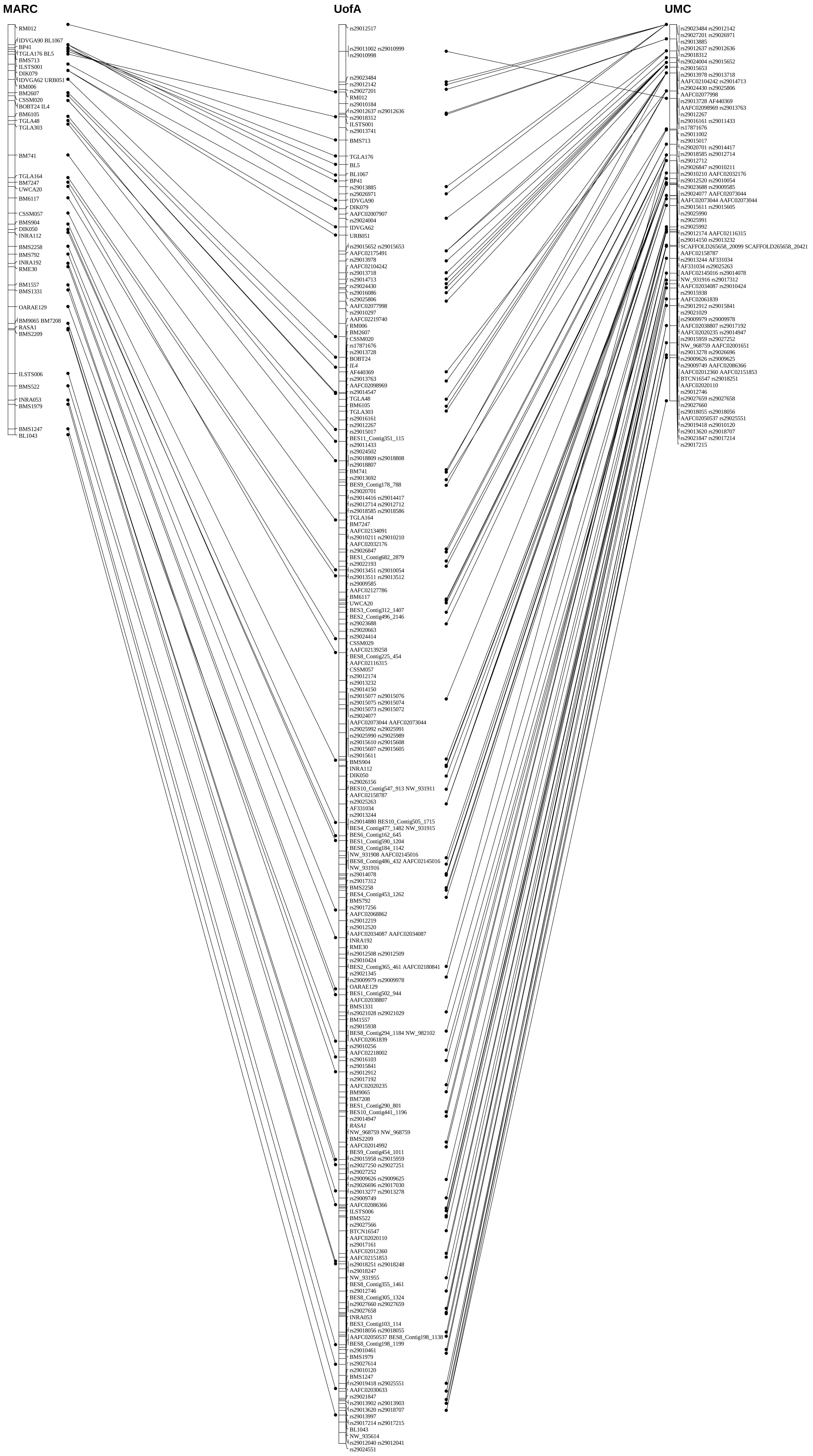
MARC
UofA
UMC
RM012
IDVGA90 BL1067
BP41
TGLA176 BL5
BMS713
ILSTS001
DIK079
IDVGA62 URB051
RM006
BM2607
CSSM020
BOBT24 IL4
BM6105
TGLA48
TGLA303
BM741
TGLA164
BM7247
UWCA20
BM6117
CSSM057
BMS904
DIK050
INRA112
BMS2258
BMS792
INRA192
RME30
BM1557
BMS1331
OARAE129
BM9065 BM7208
RASA1
BMS2209
ILSTS006
BMS522
INRA053
BMS1979
BMS1247
BL1043
rs29012517
rs29011002 rs29010999
rs29010998
rs29023484
rs29012142
rs29027201
RM012
rs29010184
rs29012637 rs29012636
rs29018312
ILSTS001
rs29013741
BMS713
TGLA176
BL5
BL1067
BP41
rs29013885
rs29026971
IDVGA90
DIK079
AAFC02007907
rs29024004
IDVGA62
URB051
rs29015652 rs29015653
AAFC02175491
rs29013978
AAFC02104242
rs29013718
rs29014713
rs29024430
rs29016086
rs29025806
AAFC02077998
rs29010297
AAFC02219740
RM006
BM2607
CSSM020
rs17871676
rs29013728
BOBT24
IL4
AF440369
rs29013763
AAFC02098969
rs29014547
TGLA48
BM6105
TGLA303
rs29016161
rs29012267
rs29015017
BES11_Contig351_115
rs29011433
rs29024502
rs29018809 rs29018808
rs29018807
BM741
rs29013692
BES9_Contig178_788
rs29020701
rs29014416 rs29014417
rs29012714 rs29012712
rs29018585 rs29018586
TGLA164
BM7247
AAFC02134091
rs29010211 rs29010210
AAFC02032176
rs29026847
BES1_Contig682_2879
rs29022193
rs29013451 rs29010054
rs29013511 rs29013512
rs29009585
AAFC02127786
BM6117
UWCA20
BES3_Contig312_1407
BES2_Contig496_2146
rs29023688
rs29020663
rs29024414
CSSM029
AAFC02139258
BES8_Contig225_454
AAFC02116315
CSSM057
rs29012174
rs29013232
rs29014150
rs29015077 rs29015076
rs29015075 rs29015074
rs29015073 rs29015072
rs29024077
AAFC02073044 AAFC02073044
rs29025992 rs29025991
rs29025990 rs29025989
rs29015610 rs29015608
rs29015607 rs29015605
rs29015611
BMS904
INRA112
DIK050
rs29026156
BES10_Contig547_913 NW_931911
AAFC02158787
rs29025263
AF331034
rs29013244
rs29014880 BES10_Contig505_1715
BES4_Contig477_1482 NW_931915
BES6_Contig162_645
BES1_Contig590_1204
BES8_Contig184_1142
NW_931908 AAFC02145016
BES8_Contig486_432 AAFC02145016
NW_931916
rs29014078
rs29017312
BMS2258
BES4_Contig453_1262
BMS792
rs29017256
AAFC02068862
rs29012219
rs29012520
AAFC02034087 AAFC02034087
INRA192
RME30
rs29012508 rs29012509
rs29010424
BES2_Contig365_461 AAFC02180841
rs29021345
rs29009979 rs29009978
OARAE129
BES1_Contig502_944
AAFC02038807
BMS1331
rs29021028 rs29021029
BM1557
rs29015938
BES8_Contig294_1184 NW_982102
AAFC02061839
rs29010256
AAFC02218002
rs29016103
rs29015841
rs29012912
rs29017192
AAFC02020235
BM9065
BM7208
BES1_Contig290_801
BES10_Contig441_1196
rs29014947
RASA1
NW_968759 NW_968759
BMS2209
AAFC02014992
BES9_Contig454_1011
rs29015958 rs29015959
rs29027250 rs29027251
rs29027252
rs29009626 rs29009625
rs29026696 rs29017030
rs29013277 rs29013278
rs29009749
AAFC02086366
ILSTS006
BMS522
rs29027566
BTCN16547
AAFC02020110
rs29017161
AAFC02012360
AAFC02151853
rs29018251 rs29018248
rs29018247
NW_931955
BES8_Contig355_1461
rs29012746
BES8_Contig305_1324
rs29027660 rs29027659
rs29027658
INRA053
BES3_Contig103_114
rs29018056 rs29018055
AAFC02050537 BES8_Contig198_1138
BES8_Contig198_1199
rs29010461
BMS1979
rs29027614
rs29010120
BMS1247
rs29019418 rs29025551
AAFC02030633
rs29021847
rs29013902 rs29013903
rs29013620 rs29018707
rs29013997
rs29017214 rs29017215
BL1043
NW_935614
rs29012040 rs29012041
rs29024551
rs29023484 rs29012142
rs29027201 rs29026971
rs29013885
rs29012637 rs29012636
rs29018312
rs29024004 rs29015652
rs29015653
rs29013978 rs29013718
AAFC02104242 rs29014713
rs29024430 rs29025806
AAFC02077998
rs29013728 AF440369
AAFC02098969 rs29013763
rs29012267
rs29016161 rs29011433
rs17871676
rs29011002
rs29015017
rs29020701 rs29014417
rs29018585 rs29012714
rs29012712
rs29026847 rs29010211
rs29010210 AAFC02032176
rs29012520 rs29010054
rs29023688 rs29009585
rs29024077 AAFC02073044
AAFC02073044 AAFC02073044
rs29015611 rs29015605
rs29025990
rs29025991
rs29025992
rs29012174 AAFC02116315
rs29014150 rs29013232
SCAFFOLD265658_20099 SCAFFOLD265658_20421
AAFC02158787
rs29013244 AF331034
AF331034 rs29025263
AAFC02145016 rs29014078
NW_931916 rs29017312
AAFC02034087 rs29010424
rs29015938
AAFC02061839
rs29012912 rs29015841
rs29021029
rs29009979 rs29009978
AAFC02038807 rs29017192
AAFC02020235 rs29014947
rs29015959 rs29027252
NW_968759 AAFC02001651
rs29013278 rs29026696
rs29009626 rs29009625
rs29009749 AAFC02086366
AAFC02012360 AAFC02151853
BTCN16547 rs29018251
AAFC02020110
rs29012746
rs29027659 rs29027658
rs29027660
rs29018055 rs29018056
AAFC02050537 rs29025551
rs29019418 rs29010120
rs29013620 rs29018707
rs29021847 rs29017214
rs29017215

BTA7

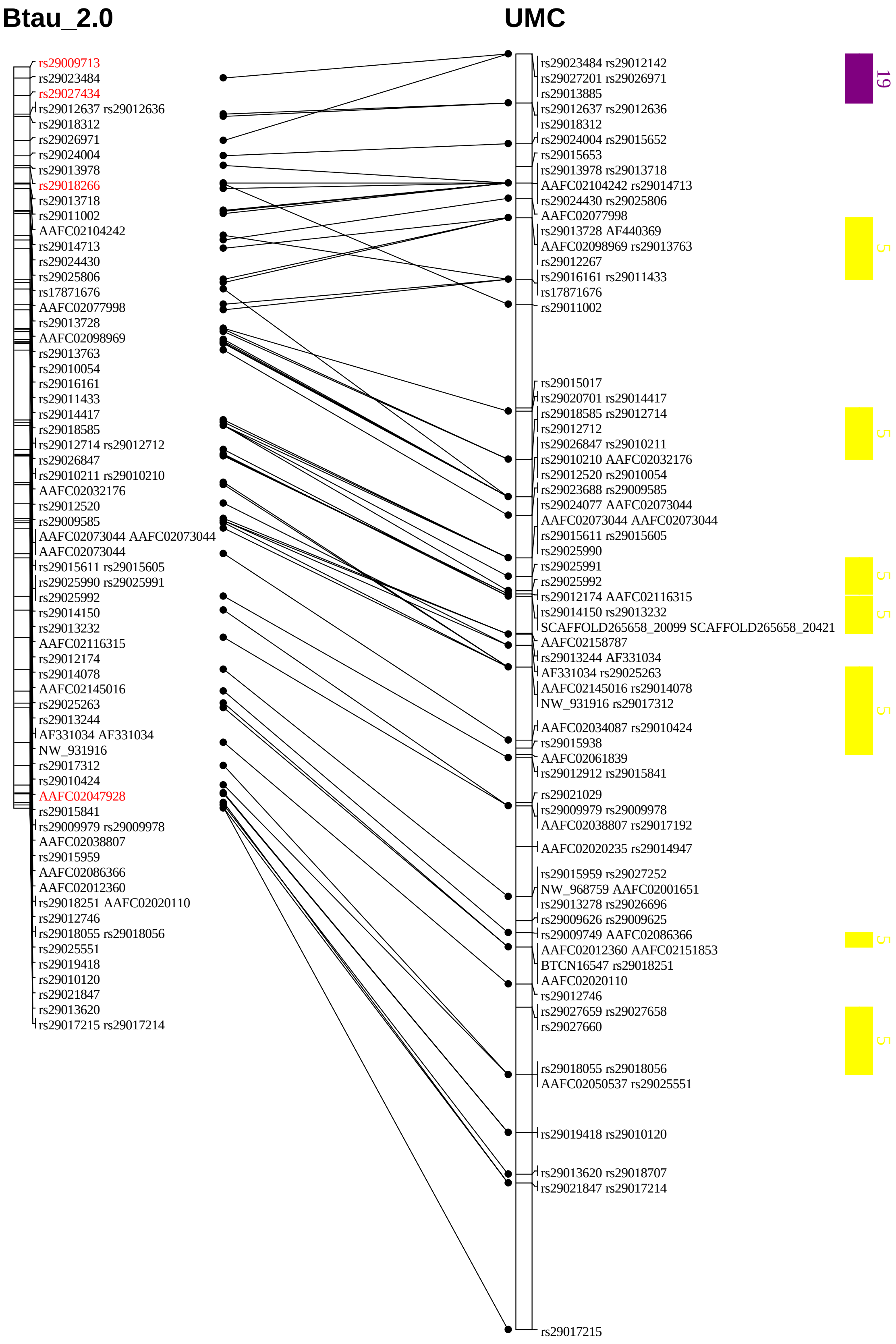
Btau_2.0
UMC
rs29009713
rs29023484
rs29027434
rs29012637 rs29012636
rs29018312
rs29026971
rs29024004
rs29013978
rs29018266
rs29013718
rs29011002
AAFC02104242
rs29014713
rs29024430
rs29025806
rs17871676
AAFC02077998
rs29013728
AAFC02098969
rs29013763
rs29010054
rs29016161
rs29011433
rs29014417
rs29018585
rs29012714 rs29012712
rs29026847
rs29010211 rs29010210
AAFC02032176
rs29012520
rs29009585
AAFC02073044 AAFC02073044
AAFC02073044
rs29015611 rs29015605
rs29025990 rs29025991
rs29025992
rs29014150
rs29013232
AAFC02116315
rs29012174
rs29014078
AAFC02145016
rs29025263
rs29013244
AF331034 AF331034
NW_931916
rs29017312
rs29010424
AAFC02047928
rs29015841
rs29009979 rs29009978
AAFC02038807
rs29015959
AAFC02086366
AAFC02012360
rs29018251 AAFC02020110
rs29012746
rs29018055 rs29018056
rs29025551
rs29019418
rs29010120
rs29021847
rs29013620
rs29017215 rs29017214
rs29023484 rs29012142
rs29027201 rs29026971
rs29013885
rs29012637 rs29012636
rs29018312
rs29024004 rs29015652
rs29015653
rs29013978 rs29013718
AAFC02104242 rs29014713
rs29024430 rs29025806
AAFC02077998
rs29013728 AF440369
AAFC02098969 rs29013763
rs29012267
rs29016161 rs29011433
rs17871676
rs29011002
rs29015017
rs29020701 rs29014417
rs29018585 rs29012714
rs29012712
rs29026847 rs29010211
rs29010210 AAFC02032176
rs29012520 rs29010054
rs29023688 rs29009585
rs29024077 AAFC02073044
AAFC02073044 AAFC02073044
rs29015611 rs29015605
rs29025990
rs29025991
rs29025992
rs29012174 AAFC02116315
rs29014150 rs29013232
SCAFFOLD265658_20099 SCAFFOLD265658_20421
AAFC02158787
rs29013244 AF331034
AF331034 rs29025263
AAFC02145016 rs29014078
NW_931916 rs29017312
AAFC02034087 rs29010424
rs29015938
AAFC02061839
rs29012912 rs29015841
rs29021029
rs29009979 rs29009978
AAFC02038807 rs29017192
AAFC02020235 rs29014947
rs29015959 rs29027252
NW_968759 AAFC02001651
rs29013278 rs29026696
rs29009626 rs29009625
rs29009749 AAFC02086366
AAFC02012360 AAFC02151853
BTCN16547 rs29018251
AAFC02020110
rs29012746
rs29027659 rs29027658
rs29027660
rs29018055 rs29018056
AAFC02050537 rs29025551
rs29019418 rs29010120
rs29013620 rs29018707
rs29021847 rs29017214
rs29017215
19
5
5
5
5
5
5
5

BTA8

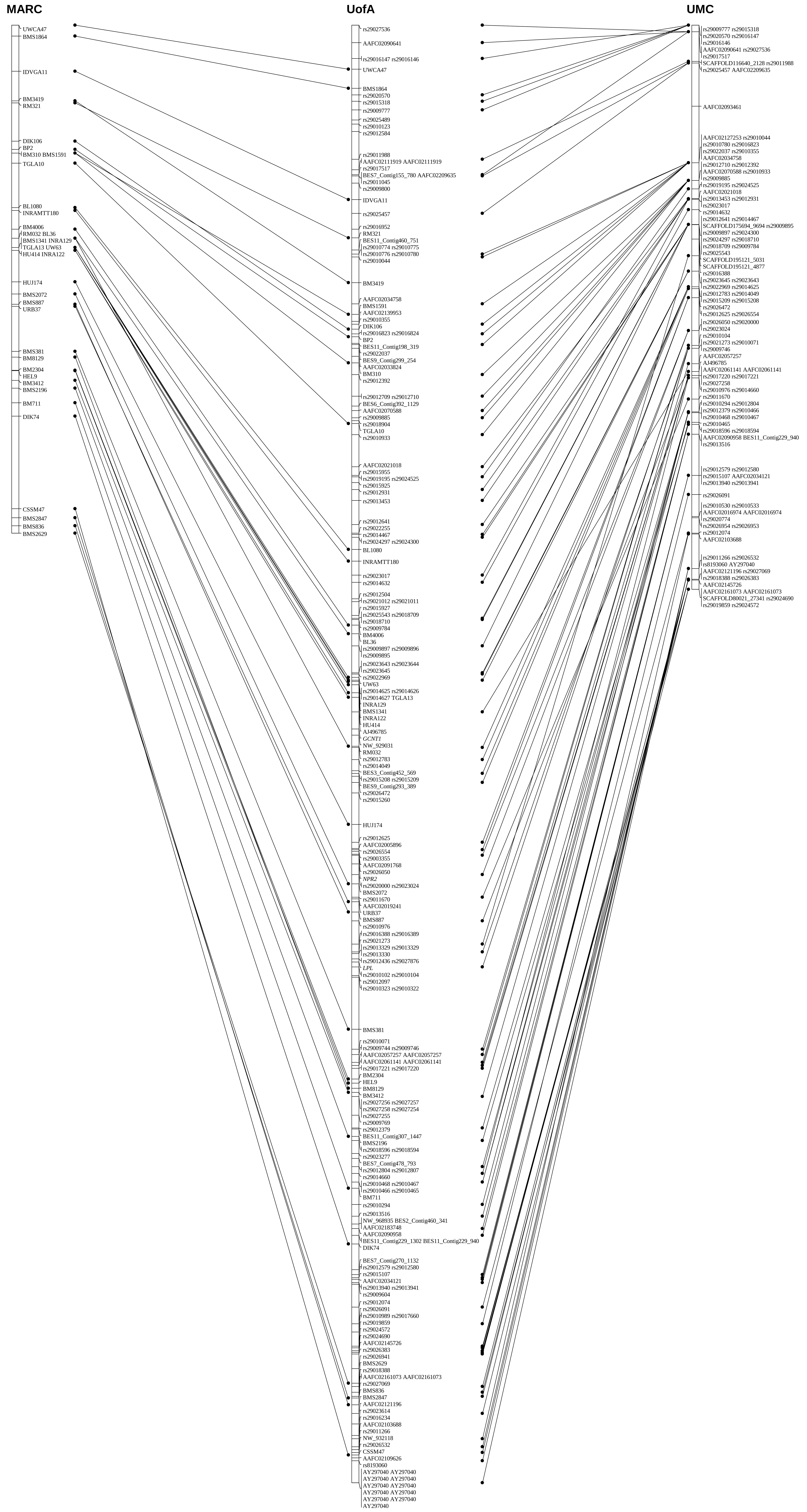
MARC
UofA
UMC
UWCA47
BMS1864
IDVGA11
BM3419
RM321
DIK106
BP2
BM310 BMS1591
TGLA10
BL1080
INRAMTT180
BM4006
RM032 BL36
BMS1341 INRA129
TGLA13 UW63
HU414 INRA122
HUJ174
BMS2072
BMS887
URB37
BMS381
BM8129
BM2304
HEL9
BM3412
BMS2196
BM711
DIK74
CSSM47
BMS2847
BMS836
BMS2629
rs29027536
AAFC02090641
rs29016147 rs29016146
UWCA47
BMS1864
rs29020570
rs29015318
rs29009777
rs29025489
rs29010123
rs29012584
rs29011988
AAFC02111919 AAFC02111919
rs29017517
BES7_Contig155_780 AAFC02209635
rs29011045
rs29009800
IDVGA11
rs29025457
rs29016952
RM321
BES11_Contig460_751
rs29010774 rs29010775
rs29010776 rs29010780
rs29010044
BM3419
AAFC02034758
BMS1591
AAFC02139953
rs29010355
DIK106
rs29016823 rs29016824
BP2
BES11_Contig198_319
rs29022037
BES9_Contig299_254
AAFC02033824
BM310
rs29012392
rs29012709 rs29012710
BES6_Contig392_1129
AAFC02070588
rs29009885
rs29018904
TGLA10
rs29010933
AAFC02021018
rs29015955
rs29019195 rs29024525
rs29015925
rs29012931
rs29013453
rs29012641
rs29022255
rs29014467
rs29024297 rs29024300
BL1080
INRAMTT180
rs29023017
rs29014632
rs29012504
rs29021012 rs29021011
rs29015927
rs29025543 rs29018709
rs29018710
rs29009784
BM4006
BL36
rs29009897 rs29009896
rs29009895
rs29023643 rs29023644
rs29023645
rs29022969
UW63
rs29014625 rs29014626
rs29014627 TGLA13
INRA129
BMS1341
INRA122
HU414
AJ496785
GCNT1
NW_929031
RM032
rs29012783
rs29014049
BES3_Contig452_569
rs29015208 rs29015209
BES9_Contig293_389
rs29026472
rs29015260
HUJ174
rs29012625
AAFC02005896
rs29026554
rs29003355
AAFC02091768
rs29026050
NPR2
rs29020000 rs29023024
BMS2072
rs29011670
AAFC02019241
URB37
BMS887
rs29010976
rs29016388 rs29016389
rs29021273
rs29013329 rs29013329
rs29013330
rs29012436 rs29027876
LPL
rs29010102 rs29010104
rs29012097
rs29010323 rs29010322
BMS381
rs29010071
rs29009744 rs29009746
AAFC02057257 AAFC02057257
AAFC02061141 AAFC02061141
rs29017221 rs29017220
BM2304
HEL9
BM8129
BM3412
rs29027256 rs29027257
rs29027258 rs29027254
rs29027255
rs29009769
rs29012379
BES11_Contig307_1447
BMS2196
rs29018596 rs29018594
rs29023277
BES7_Contig478_793
rs29012804 rs29012807
rs29014660
rs29010468 rs29010467
rs29010466 rs29010465
BM711
rs29010294
rs29013516
NW_968935 BES2_Contig460_341
AAFC02183748
AAFC02090958
BES11_Contig229_1302 BES11_Contig229_940
DIK74
BES7_Contig270_1132
rs29012579 rs29012580
rs29015107
AAFC02034121
rs29013940 rs29013941
rs29009604
rs29012074
rs29026091
rs29010989 rs29017660
rs29019859
rs29024572
rs29024690
AAFC02145726
rs29026383
rs29026941
BMS2629
rs29018388
AAFC02161073 AAFC02161073
rs29027069
BMS836
BMS2847
AAFC02121196
rs29023614
rs29016234
AAFC02103688
rs29011266
NW_932118
rs29026532
CSSM47
AAFC02109626
rs8193060
AY297040 AY297040
AY297040 AY297040
AY297040 AY297040
AY297040 AY297040
AY297040 AY297040
AY297040
rs29009777 rs29015318
rs29020570 rs29016147
rs29016146
AAFC02090641 rs29027536
rs29017517
SCAFFOLD116640_2128 rs29011988
rs29025457 AAFC02209635
AAFC02093461
AAFC02127253 rs29010044
rs29010780 rs29016823
rs29022037 rs29010355
AAFC02034758
rs29012710 rs29012392
AAFC02070588 rs29010933
rs29009885
rs29019195 rs29024525
AAFC02021018
rs29013453 rs29012931
rs29023017
rs29014632
rs29012641 rs29014467
SCAFFOLD175694_9694 rs29009895
rs29009897 rs29024300
rs29024297 rs29018710
rs29018709 rs29009784
rs29025543
SCAFFOLD195121_5031
SCAFFOLD195121_4877
rs29016388
rs29023645 rs29023643
rs29022969 rs29014625
rs29012783 rs29014049
rs29015209 rs29015208
rs29026472
rs29012625 rs29026554
rs29026050 rs29020000
rs29023024
rs29010104
rs29021273 rs29010071
rs29009746
AAFC02057257
AJ496785
AAFC02061141 AAFC02061141
rs29017220 rs29017221
rs29027258
rs29010976 rs29014660
rs29011670
rs29010294 rs29012804
rs29012379 rs29010466
rs29010468 rs29010467
rs29010465
rs29018596 rs29018594
AAFC02090958 BES11_Contig229_940
rs29013516
rs29012579 rs29012580
rs29015107 AAFC02034121
rs29013940 rs29013941
rs29026091
rs29010530 rs29010533
AAFC02016974 AAFC02016974
rs29020774
rs29026954 rs29026953
rs29012074
AAFC02103688
rs29011266 rs29026532
rs8193060 AY297040
AAFC02121196 rs29027069
rs29018388 rs29026383
AAFC02145726
AAFC02161073 AAFC02161073
SCAFFOLD80021_27341 rs29024690
rs29019859 rs29024572

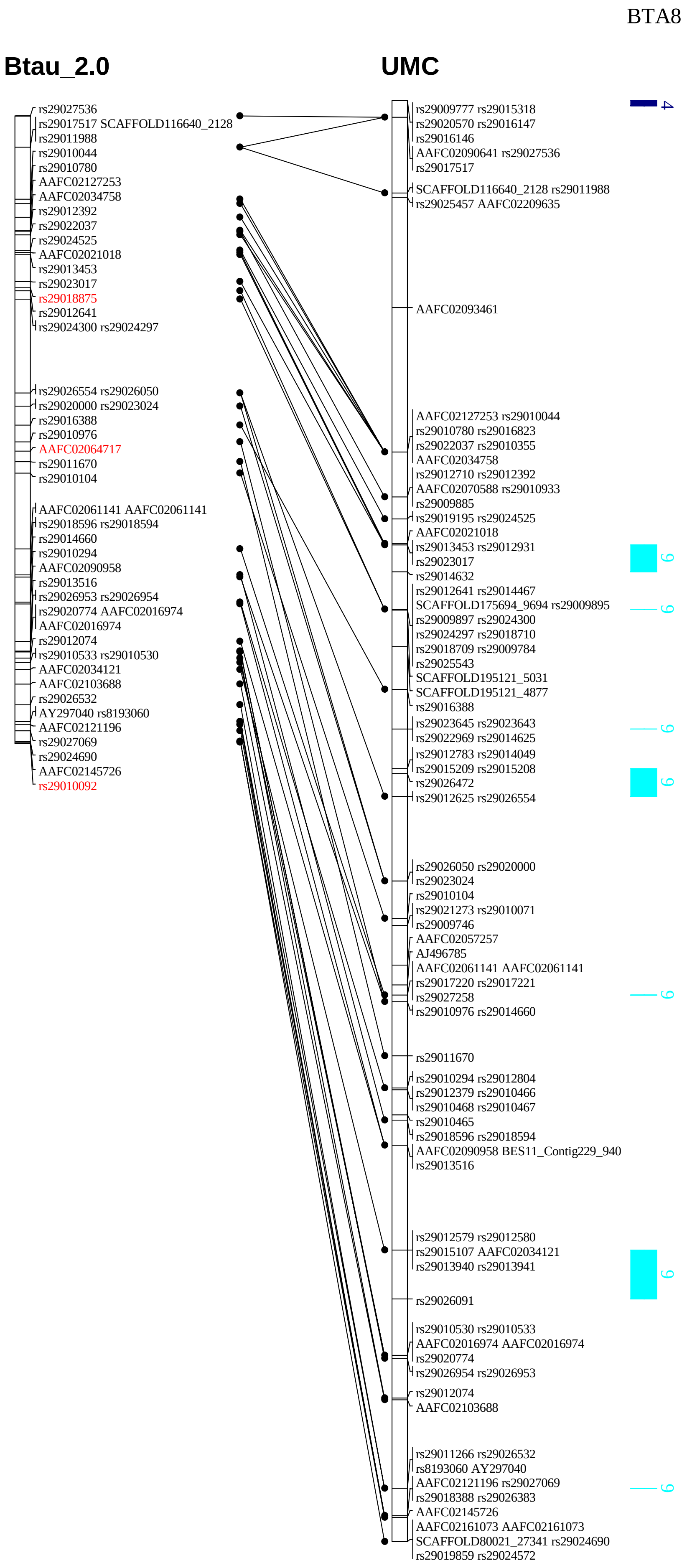

BTA8
Btau_2.0
UMC
rs29027536
rs29017517 SCAFFOLD116640_2128
rs29011988
rs29010044
rs29010780
AAFC02127253
AAFC02034758
rs29012392
rs29022037
rs29024525
AAFC02021018
rs29013453
rs29023017
rs29018875
rs29012641
rs29024300 rs29024297
rs29026554 rs29026050
rs29020000 rs29023024
rs29016388
rs29010976
AAFC02064717
rs29011670
rs29010104
AAFC02061141 AAFC02061141
rs29018596 rs29018594
rs29014660
rs29010294
AAFC02090958
rs29013516
rs29026953 rs29026954
rs29020774 AAFC02016974
AAFC02016974
rs29012074
rs29010533 rs29010530
AAFC02034121
AAFC02103688
rs29026532
AY297040 rs8193060
AAFC02121196
rs29027069
rs29024690
AAFC02145726
rs29010092
rs29009777 rs29015318
rs29020570 rs29016147
rs29016146
AAFC02090641 rs29027536
rs29017517
SCAFFOLD116640_2128 rs29011988
rs29025457 AAFC02209635
AAFC02093461
AAFC02127253 rs29010044
rs29010780 rs29016823
rs29022037 rs29010355
AAFC02034758
rs29012710 rs29012392
AAFC02070588 rs29010933
rs29009885
rs29019195 rs29024525
AAFC02021018
rs29013453 rs29012931
rs29023017
rs29014632
rs29012641 rs29014467
SCAFFOLD175694_9694 rs29009895
rs29009897 rs29024300
rs29024297 rs29018710
rs29018709 rs29009784
rs29025543
SCAFFOLD195121_5031
SCAFFOLD195121_4877
rs29016388
rs29023645 rs29023643
rs29022969 rs29014625
rs29012783 rs29014049
rs29015209 rs29015208
rs29026472
rs29012625 rs29026554
rs29026050 rs29020000
rs29023024
rs29010104
rs29021273 rs29010071
rs29009746
AAFC02057257
AJ496785
AAFC02061141 AAFC02061141
rs29017220 rs29017221
rs29027258
rs29010976 rs29014660
rs29011670
rs29010294 rs29012804
rs29012379 rs29010466
rs29010468 rs29010467
rs29010465
rs29018596 rs29018594
AAFC02090958 BES11_Contig229_940
rs29013516
rs29012579 rs29012580
rs29015107 AAFC02034121
rs29013940 rs29013941
rs29026091
rs29010530 rs29010533
AAFC02016974 AAFC02016974
rs29020774
rs29026954 rs29026953
rs29012074
AAFC02103688
rs29011266 rs29026532
rs8193060 AY297040
AAFC02121196 rs29027069
rs29018388 rs29026383
AAFC02145726
AAFC02161073 AAFC02161073
SCAFFOLD80021_27341 rs29024690
rs29019859 rs29024572
4
6
6
6
6
6
6

BTA9

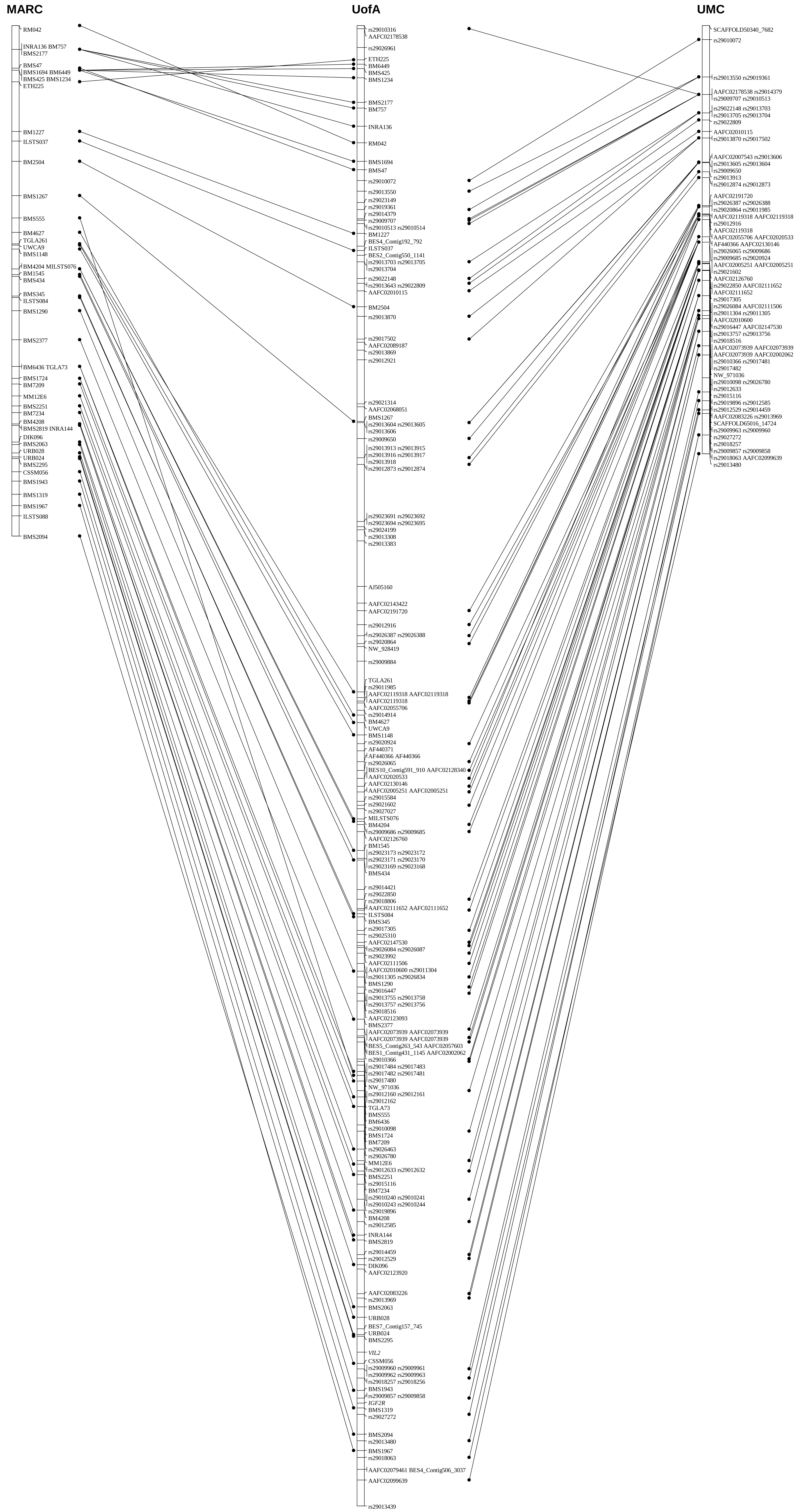
MARC
UofA
UMC
RM042
INRA136 BM757
BMS2177
BMS47
BMS1694 BM6449
BMS425 BMS1234
ETH225
BM1227
ILSTS037
BM2504
BMS1267
BMS555
BM4627
TGLA261
UWCA9
BMS1148
BM4204 MILSTS076
BM1545
BMS434
BMS345
ILSTS084
BMS1290
BMS2377
BM6436 TGLA73
BMS1724
BM7209
MM12E6
BMS2251
BM7234
BM4208
BMS2819 INRA144
DIK096
BMS2063
URB028
URB024
BMS2295
CSSM056
BMS1943
BMS1319
BMS1967
ILSTS088
BMS2094
rs29010316
AAFC02178538
rs29026961
ETH225
BM6449
BMS425
BMS1234
BMS2177
BM757
INRA136
RM042
BMS1694
BMS47
rs29010072
rs29013550
rs29023149
rs29019361
rs29014379
rs29009707
rs29010513 rs29010514
BM1227
BES4_Contig192_792
ILSTS037
BES2_Contig550_1141
rs29013703 rs29013705
rs29013704
rs29022148
rs29013643 rs29022809
AAFC02010115
BM2504
rs29013870
rs29017502
AAFC02089187
rs29013869
rs29012921
rs29021314
AAFC02068051
BMS1267
rs29013604 rs29013605
rs29013606
rs29009650
rs29013913 rs29013915
rs29013916 rs29013917
rs29013918
rs29012873 rs29012874
rs29023691 rs29023692
rs29023694 rs29023695
rs29024199
rs29013308
rs29013383
AJ505160
AAFC02143422
AAFC02191720
rs29012916
rs29026387 rs29026388
rs29020864
NW_928419
rs29009884
TGLA261
rs29011985
AAFC02119318 AAFC02119318
AAFC02119318
AAFC02055706
rs29014914
BM4627
UWCA9
BMS1148
rs29020924
AF440371
AF440366 AF440366
rs29026065
BES10_Contig591_910 AAFC02128340
AAFC02020533
AAFC02130146
AAFC02005251 AAFC02005251
rs29015584
rs29021602
rs29027027
MILSTS076
BM4204
rs29009686 rs29009685
AAFC02126760
BM1545
rs29023173 rs29023172
rs29023171 rs29023170
rs29023169 rs29023168
BMS434
rs29014421
rs29022850
rs29018806
AAFC02111652 AAFC02111652
ILSTS084
BMS345
rs29017305
rs29025310
AAFC02147530
rs29026084 rs29026087
rs29023992
AAFC02111506
AAFC02010600 rs29011304
rs29011305 rs29026834
BMS1290
rs29016447
rs29013755 rs29013758
rs29013757 rs29013756
rs29018516
AAFC02123093
BMS2377
AAFC02073939 AAFC02073939
AAFC02073939 AAFC02073939
BES5_Contig263_543 AAFC02057603
BES1_Contig431_1145 AAFC02002062
rs29010366
rs29017484 rs29017483
rs29017482 rs29017481
rs29017480
NW_971036
rs29012160 rs29012161
rs29012162
TGLA73
BMS555
BM6436
rs29010098
BMS1724
BM7209
rs29026463
rs29026780
MM12E6
rs29012633 rs29012632
BMS2251
rs29015116
BM7234
rs29010240 rs29010241
rs29010243 rs29010244
rs29019896
BM4208
rs29012585
INRA144
BMS2819
rs29014459
rs29012529
DIK096
AAFC02123920
AAFC02083226
rs29013969
BMS2063
URB028
BES7_Contig157_745
URB024
BMS2295
VIL2
CSSM056
rs29009960 rs29009961
rs29009962 rs29009963
rs29018257 rs29018256
BMS1943
rs29009857 rs29009858
IGF2R
BMS1319
rs29027272
BMS2094
rs29013480
BMS1967
rs29018063
AAFC02079461 BES4_Contig506_3037
AAFC02099639
rs29013439
SCAFFOLD50340_7682
rs29010072
rs29013550 rs29019361
AAFC02178538 rs29014379
rs29009707 rs29010513
rs29022148 rs29013703
rs29013705 rs29013704
rs29022809
AAFC02010115
rs29013870 rs29017502
AAFC02007543 rs29013606
rs29013605 rs29013604
rs29009650
rs29013913
rs29012874 rs29012873
AAFC02191720
rs29026387 rs29026388
rs29020864 rs29011985
AAFC02119318 AAFC02119318
rs29012916
AAFC02119318
AAFC02055706 AAFC02020533
AF440366 AAFC02130146
rs29026065 rs29009686
rs29009685 rs29020924
AAFC02005251 AAFC02005251
rs29021602
AAFC02126760
rs29022850 AAFC02111652
AAFC02111652
rs29017305
rs29026084 AAFC02111506
rs29011304 rs29011305
AAFC02010600
rs29016447 AAFC02147530
rs29013757 rs29013756
rs29018516
AAFC02073939 AAFC02073939
AAFC02073939 AAFC02002062
rs29010366 rs29017481
rs29017482
NW_971036
rs29010098 rs29026780
rs29012633
rs29015116
rs29019896 rs29012585
rs29012529 rs29014459
AAFC02083226 rs29013969
SCAFFOLD65016_14724
rs29009963 rs29009960
rs29027272
rs29018257
rs29009857 rs29009858
rs29018063 AAFC02099639
rs29013480

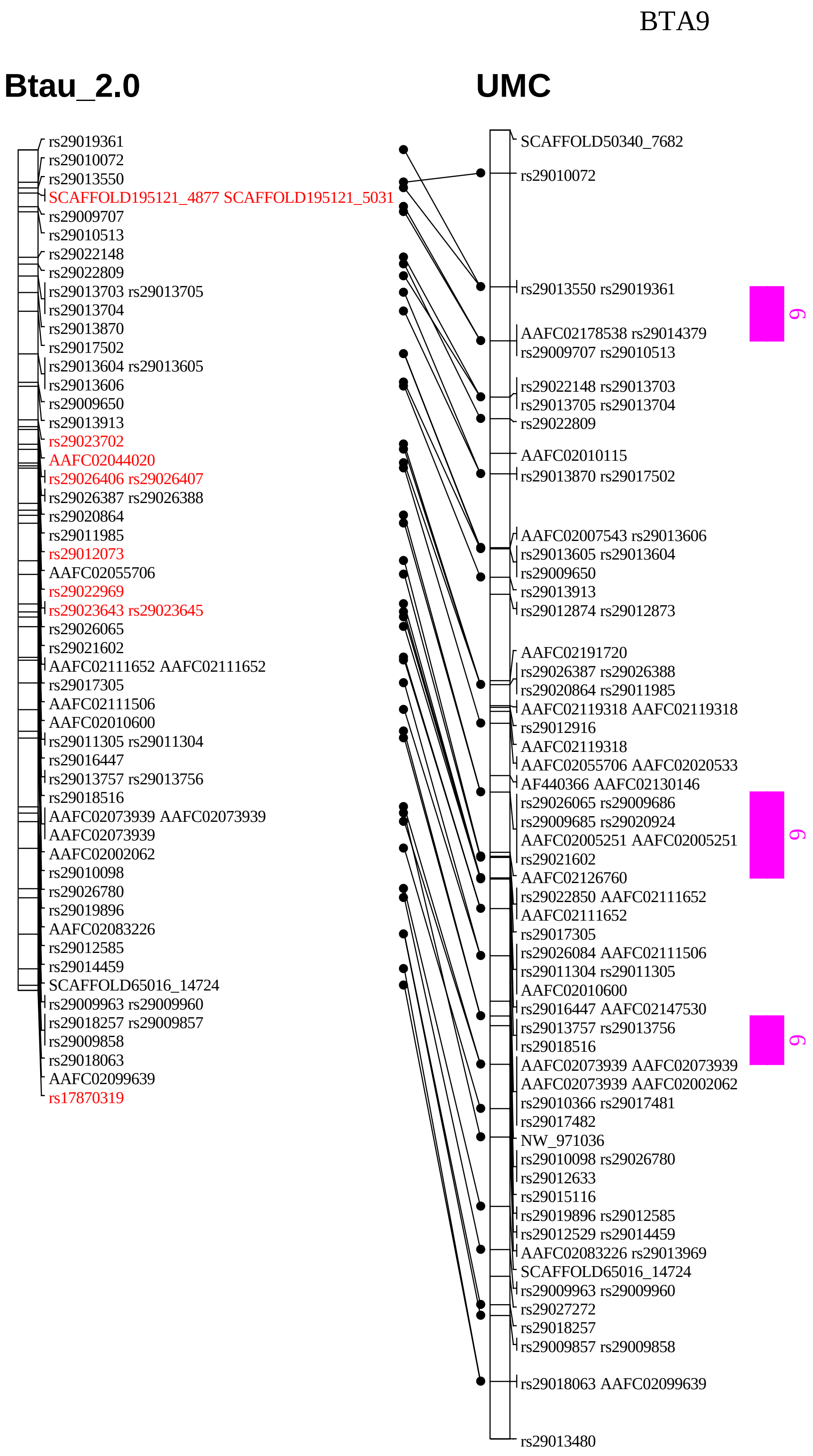
BTA9
Btau_2.0
UMC
rs29019361
rs29010072
rs29013550
SCAFFOLD195121_4877 SCAFFOLD195121_5031
rs29009707
rs29010513
rs29022148
rs29022809
rs29013703 rs29013705
rs29013704
rs29013870
rs29017502
rs29013604 rs29013605
rs29013606
rs29009650
rs29013913
rs29023702
AAFC02044020
rs29026406 rs29026407
rs29026387 rs29026388
rs29020864
rs29011985
rs29012073
AAFC02055706
rs29022969
rs29023643 rs29023645
rs29026065
rs29021602
AAFC02111652 AAFC02111652
rs29017305
AAFC02111506
AAFC02010600
rs29011305 rs29011304
rs29016447
rs29013757 rs29013756
rs29018516
AAFC02073939 AAFC02073939
AAFC02073939
AAFC02002062
rs29010098
rs29026780
rs29019896
AAFC02083226
rs29012585
rs29014459
SCAFFOLD65016_14724
rs29009963 rs29009960
rs29018257 rs29009857
rs29009858
rs29018063
AAFC02099639
rs17870319
SCAFFOLD50340_7682
rs29010072
rs29013550 rs29019361
AAFC02178538 rs29014379
rs29009707 rs29010513
rs29022148 rs29013703
rs29013705 rs29013704
rs29022809
AAFC02010115
rs29013870 rs29017502
AAFC02007543 rs29013606
rs29013605 rs29013604
rs29009650
rs29013913
rs29012874 rs29012873
AAFC02191720
rs29026387 rs29026388
rs29020864 rs29011985
AAFC02119318 AAFC02119318
rs29012916
AAFC02119318
AAFC02055706 AAFC02020533
AF440366 AAFC02130146
rs29026065 rs29009686
rs29009685 rs29020924
AAFC02005251 AAFC02005251
rs29021602
AAFC02126760
rs29022850 AAFC02111652
AAFC02111652
rs29017305
rs29026084 AAFC02111506
rs29011304 rs29011305
AAFC02010600
rs29016447 AAFC02147530
rs29013757 rs29013756
rs29018516
AAFC02073939 AAFC02073939
AAFC02073939 AAFC02002062
rs29010366 rs29017481
rs29017482
NW_971036
rs29010098 rs29026780
rs29012633
rs29015116
rs29019896 rs29012585
rs29012529 rs29014459
AAFC02083226 rs29013969
SCAFFOLD65016_14724
rs29009963 rs29009960
rs29027272
rs29018257
rs29009857 rs29009858
rs29018063 AAFC02099639
rs29013480
9
9
9

BTA10

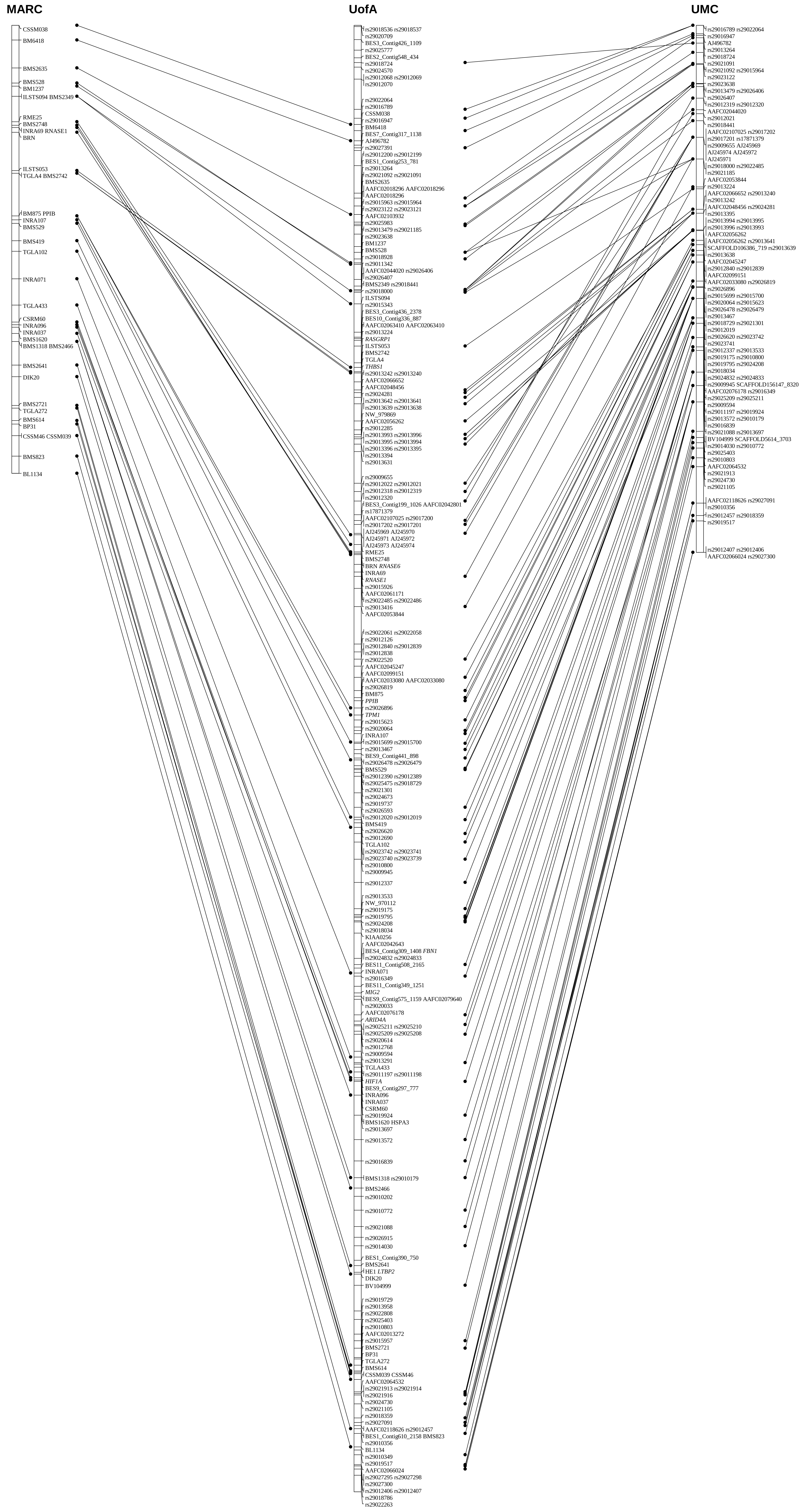
MARC
CSSM038
BM6418
BMS2635
BMS528
BM1237
ILSTS094 BMS2349
RME25
BMS2748
INRA69 RNASE1
BRN
ILSTS053
TGLA4 BMS2742
BM875 PPIB
INRA107
BMS529
BMS419
TGLA102
INRA071
TGLA433
CSRM60
INRA096
INRA037
BMS1620
BMS1318 BMS2466
BMS2641
DIK20
BMS2721
TGLA272
BMS614
BP31
CSSM46 CSSM039
BMS823
BL1134
UofA
rs29018536 rs29018537
rs29020709
BES3_Contig426_1109
rs29025777
BES2_Contig548_434
rs29018724
rs29024570
rs29012068 rs29012069
rs29012070
rs29022064
rs29016789
CSSM038
rs29016947
BM6418
BES7_Contig317_1138
AJ496782
rs29027391
rs29012200 rs29012199
BES1_Contig253_781
rs29013264
rs29021092 rs29021091
BMS2635
AAFC02018296 AAFC02018296
AAFC02018296
rs29015963 rs29015964
rs29023122 rs29023121
AAFC02103932
rs29025983
rs29013479 rs29021185
rs29023638
BM1237
BMS528
rs29018928
rs29011342
AAFC02044020 rs29026406
rs29026407
BMS2349 rs29018441
rs29018000
ILSTS094
rs29015343
BES3_Contig436_2378
BES10_Contig336_887
AAFC02063410 AAFC02063410
rs29013224
RASGRP1
ILSTS053
BMS2742
TGLA4
THBS1
rs29013242 rs29013240
AAFC02066652
AAFC02048456
rs29024281
rs29013642 rs29013641
rs29013639 rs29013638
NW_979869
AAFC02056262
rs29012285
rs29013993 rs29013996
rs29013995 rs29013994
rs29013396 rs29013395
rs29013394
rs29013631
rs29009655
rs29012022 rs29012021
rs29012318 rs29012319
rs29012320
BES3_Contig199_1026 AAFC02042801
rs17871379
AAFC02107025 rs29017200
rs29017202 rs29017201
AJ245969 AJ245970
AJ245971 AJ245972
AJ245973 AJ245974
RME25
BMS2748
BRN RNASE6
INRA69
RNASE1
rs29015926
AAFC02061171
rs29022485 rs29022486
rs29013416
AAFC02053844
rs29022061 rs29022058
rs29012126
rs29012840 rs29012839
rs29012838
rs29022520
AAFC02045247
AAFC02099151
AAFC02033080 AAFC02033080
rs29026819
BM875
PPIB
rs29026896
TPM1
rs29015623
rs29020064
INRA107
rs29015699 rs29015700
rs29013467
BES9_Contig441_898
rs29026478 rs29026479
BMS529
rs29012390 rs29012389
rs29025475 rs29018729
rs29021301
rs29024673
rs29019737
rs29026593
rs29012020 rs29012019
BMS419
rs29026620
rs29012690
TGLA102
rs29023742 rs29023741
rs29023740 rs29023739
rs29010800
rs29009945
rs29012337
rs29013533
NW_970112
rs29019175
rs29019795
rs29024208
rs29018034
KIAA0256
AAFC02042643
BES4_Contig309_1408 FBN1
rs29024832 rs29024833
BES11_Contig508_2165
INRA071
rs29016349
BES11_Contig349_1251
MIG2
BES9_Contig575_1159 AAFC02079640
rs29020033
AAFC02076178
ARID4A
rs29025211 rs29025210
rs29025209 rs29025208
rs29020614
rs29012768
rs29009594
rs29013291
TGLA433
rs29011197 rs29011198
HIF1A
BES9_Contig297_777
INRA096
INRA037
CSRM60
rs29019924
BMS1620 HSPA3
rs29013697
rs29013572
rs29016839
BMS1318 rs29010179
BMS2466
rs29010202
rs29010772
rs29021088
rs29026915
rs29014030
BES1_Contig390_750
BMS2641
HE1 LTBP2
DIK20
BV104999
rs29019729
rs29013958
rs29022808
rs29025403
rs29010803
AAFC02013272
rs29015957
BMS2721
BP31
TGLA272
BMS614
CSSM039 CSSM46
AAFC02064532
rs29021913 rs29021914
rs29021916
rs29024730
rs29021105
rs29018359
rs29027091
AAFC02118626 rs29012457
BES1_Contig610_2158 BMS823
rs29010356
BL1134
rs29010349
rs29019517
AAFC02066024
rs29027295 rs29027298
rs29027300
rs29012406 rs29012407
rs29018786
rs29022263
UMC
rs29016789 rs29022064
rs29016947
AJ496782
rs29013264
rs29018724
rs29021091
rs29021092 rs29015964
rs29023122
rs29023638
rs29013479 rs29026406
rs29026407
rs29012319 rs29012320
AAFC02044020
rs29012021
rs29018441
AAFC02107025 rs29017202
rs29017201 rs17871379
rs29009655 AJ245969
AJ245974 AJ245972
AJ245971
rs29018000 rs29022485
rs29021185
AAFC02053844
rs29013224
AAFC02066652 rs29013240
rs29013242
AAFC02048456 rs29024281
rs29013395
rs29013994 rs29013995
rs29013996 rs29013993
AAFC02056262
AAFC02056262 rs29013641
SCAFFOLD106386_719 rs29013639
rs29013638
AAFC02045247
rs29012840 rs29012839
AAFC02099151
AAFC02033080 rs29026819
rs29026896
rs29015699 rs29015700
rs29020064 rs29015623
rs29026478 rs29026479
rs29013467
rs29018729 rs29021301
rs29012019
rs29026620 rs29023742
rs29023741
rs29012337 rs29013533
rs29019175 rs29010800
rs29019795 rs29024208
rs29018034
rs29024832 rs29024833
rs29009945 SCAFFOLD156147_8320
AAFC02076178 rs29016349
rs29025209 rs29025211
rs29009594
rs29011197 rs29019924
rs29013572 rs29010179
rs29016839
rs29021088 rs29013697
BV104999 SCAFFOLD5614_3703
rs29014030 rs29010772
rs29025403
rs29010803
AAFC02064532
rs29021913
rs29024730
rs29021105
AAFC02118626 rs29027091
rs29010356
rs29012457 rs29018359
rs29019517
rs29012407 rs29012406
AAFC02066024 rs29027300

BTA10

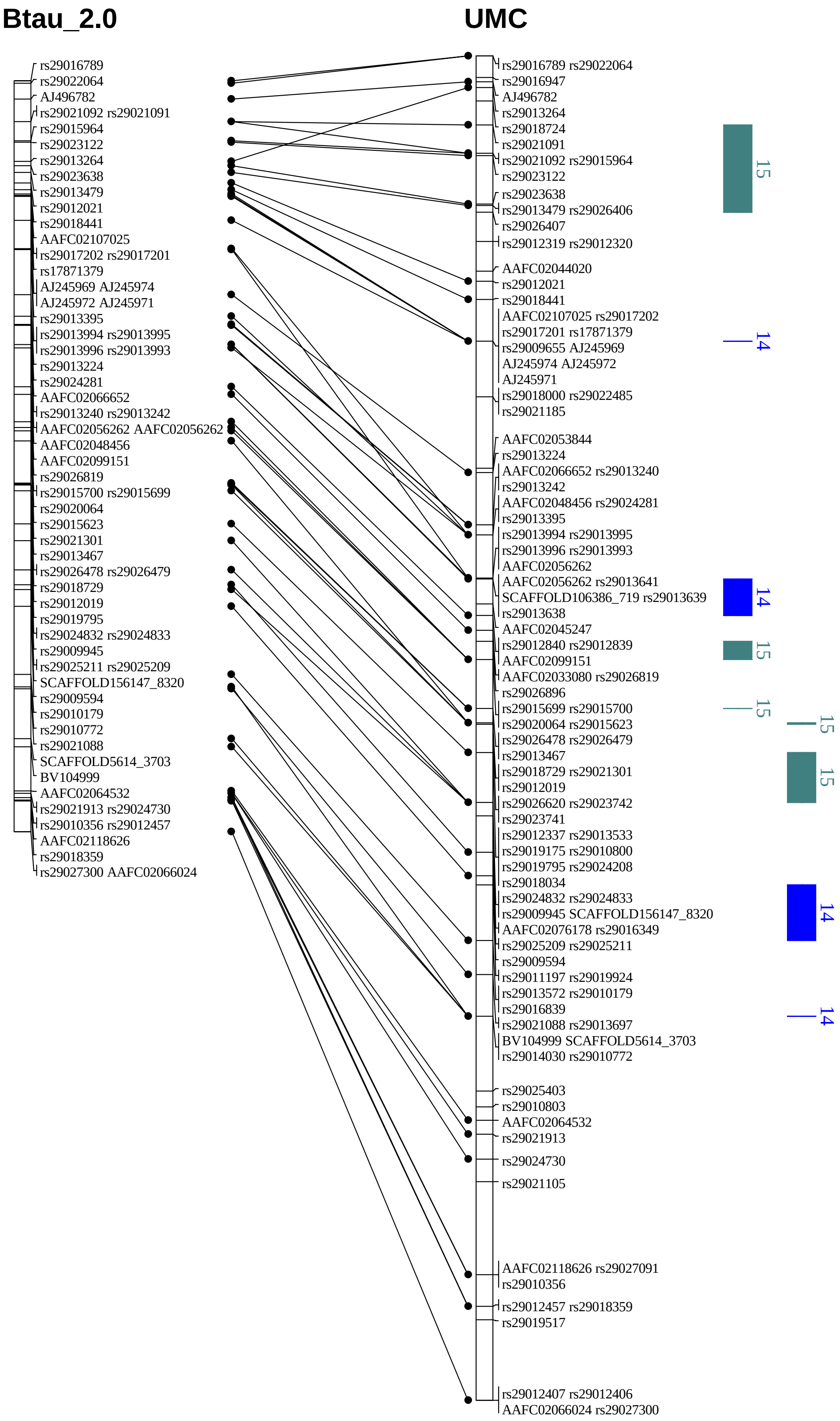
Btau_2.0
UMC
rs29016789
rs29022064
AJ496782
rs29021092 rs29021091
rs29015964
rs29023122
rs29013264
rs29023638
rs29013479
rs29012021
rs29018441
AAFC02107025
rs29017202 rs29017201
rs17871379
AJ245969 AJ245974
AJ245972 AJ245971
rs29013395
rs29013994 rs29013995
rs29013996 rs29013993
rs29013224
rs29024281
AAFC02066652
rs29013240 rs29013242
AAFC02056262 AAFC02056262
AAFC02048456
AAFC02099151
rs29026819
rs29015700 rs29015699
rs29020064
rs29015623
rs29021301
rs29013467
rs29026478 rs29026479
rs29018729
rs29012019
rs29019795
rs29024832 rs29024833
rs29009945
rs29025211 rs29025209
SCAFFOLD156147_8320
rs29009594
rs29010179
rs29010772
rs29021088
SCAFFOLD5614_3703
BV104999
AAFC02064532
rs29021913 rs29024730
rs29010356 rs29012457
AAFC02118626
rs29018359
rs29027300 AAFC02066024
rs29016789 rs29022064
rs29016947
AJ496782
rs29013264
rs29018724
rs29021091
rs29021092 rs29015964
rs29023122
rs29023638
rs29013479 rs29026406
rs29026407
rs29012319 rs29012320
AAFC02044020
rs29012021
rs29018441
AAFC02107025 rs29017202
rs29017201 rs17871379
rs29009655 AJ245969
AJ245974 AJ245972
AJ245971
rs29018000 rs29022485
rs29021185
AAFC02053844
rs29013224
AAFC02066652 rs29013240
rs29013242
AAFC02048456 rs29024281
rs29013395
rs29013994 rs29013995
rs29013996 rs29013993
AAFC02056262
AAFC02056262 rs29013641
SCAFFOLD106386_719 rs29013639
rs29013638
AAFC02045247
rs29012840 rs29012839
AAFC02099151
AAFC02033080 rs29026819
rs29026896
rs29015699 rs29015700
rs29020064 rs29015623
rs29026478 rs29026479
rs29013467
rs29018729 rs29021301
rs29012019
rs29026620 rs29023742
rs29023741
rs29012337 rs29013533
rs29019175 rs29010800
rs29019795 rs29024208
rs29018034
rs29024832 rs29024833
rs29009945 SCAFFOLD156147_8320
AAFC02076178 rs29016349
rs29025209 rs29025211
rs29009594
rs29011197 rs29019924
rs29013572 rs29010179
rs29016839
rs29021088 rs29013697
BV104999 SCAFFOLD5614_3703
rs29014030 rs29010772
rs29025403
rs29010803
AAFC02064532
rs29021913
rs29024730
rs29021105
AAFC02118626 rs29027091
rs29010356
rs29012457 rs29018359
rs29019517
rs29012407 rs29012406
AAFC02066024 rs29027300
15
14
14
15
15
15
15
14
14

BTA11

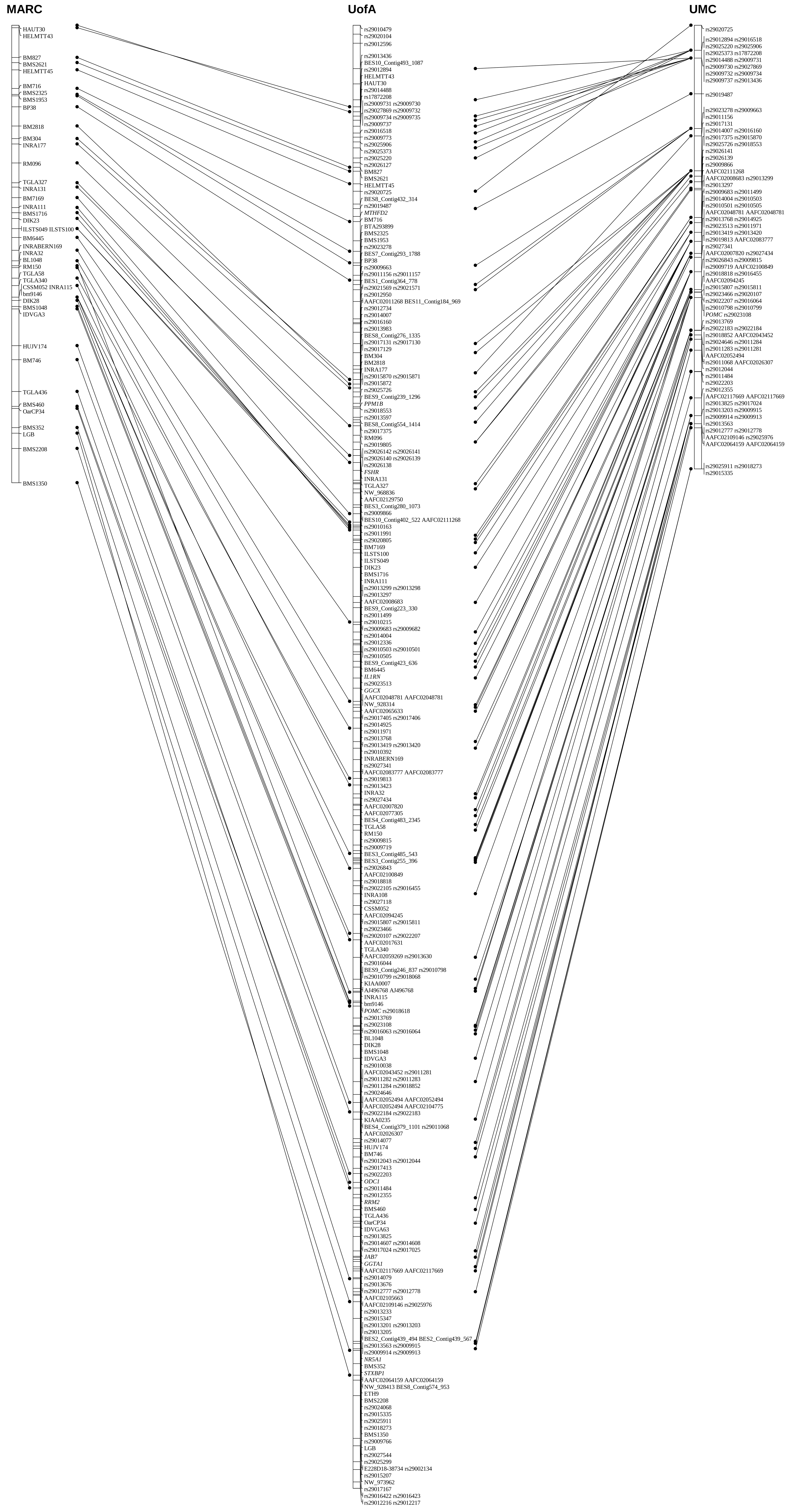
MARC
HAUT30
HELMTT43
BM827
BMS2621
HELMTT45
BM716
BMS2325
BMS1953
BP38
BM2818
BM304
INRA177
RM096
TGLA327
INRA131
BM7169
INRA111
BMS1716
DIK23
ILSTS049 ILSTS100
BM6445
INRABERN169
INRA32
BL1048
RM150
TGLA58
TGLA340
CSSM052 INRA115
bm9146
DIK28
BMS1048
IDVGA3
HUJV174
BM746
TGLA436
BMS460
OarCP34
BMS352
LGB
BMS2208
BMS1350
UofA
rs29010479
rs29020104
rs29012596
rs29013436
BES10_Contig493_1087
rs29012894
HELMTT43
HAUT30
rs29014488
rs17872208
rs29009731 rs29009730
rs29027869 rs29009732
rs29009734 rs29009735
rs29009737
rs29016518
rs29009773
rs29025906
rs29025373
rs29025220
rs29026127
BM827
BMS2621
HELMTT45
rs29020725
BES8_Contig432_314
rs29019487
MTHFD2
BM716
BTA293899
BMS2325
BMS1953
rs29023278
BES7_Contig293_1788
BP38
rs29009663
rs29011156 rs29011157
BES1_Contig364_778
rs29021569 rs29021571
rs29012950
AAFC02011268 BES11_Contig184_969
rs29012734
rs29014007
rs29016160
rs29013983
BES8_Contig276_1335
rs29017131 rs29017130
rs29017129
BM304
BM2818
INRA177
rs29015870 rs29015871
rs29015872
rs29025726
BES9_Contig239_1296
PPM1B
rs29018553
rs29013597
BES8_Contig554_1414
rs29017375
RM096
rs29019805
rs29026142 rs29026141
rs29026140 rs29026139
rs29026138
FSHR
INRA131
TGLA327
NW_968836
AAFC02129750
BES3_Contig280_1073
rs29009866
BES10_Contig402_522 AAFC02111268
rs29010163
rs29011991
rs29020805
BM7169
ILSTS100
ILSTS049
DIK23
BMS1716
INRA111
rs29013299 rs29013298
rs29013297
AAFC02008683
BES9_Contig223_330
rs29011499
rs29010215
rs29009683 rs29009682
rs29014004
rs29012336
rs29010503 rs29010501
rs29010505
BES9_Contig423_636
BM6445
IL1RN
rs29023513
GGCX
AAFC02048781 AAFC02048781
NW_928314
AAFC02065633
rs29017405 rs29017406
rs29014925
rs29011971
rs29013768
rs29013419 rs29013420
rs29010392
INRABERN169
rs29027341
AAFC02083777 AAFC02083777
rs29019813
rs29013423
INRA32
rs29027434
AAFC02007820
AAFC02077305
BES4_Contig483_2345
TGLA58
RM150
rs29009815
rs29009719
BES3_Contig485_543
BES3_Contig255_396
rs29026843
AAFC02100849
rs29018818
rs29022105 rs29016455
INRA108
rs29027118
CSSM052
AAFC02094245
rs29015807 rs29015811
rs29023466
rs29020107 rs29022207
AAFC02017631
TGLA340
AAFC02059269 rs29013630
rs29016044
BES9_Contig246_837 rs29010798
rs29010799 rs29018068
KIAA0007
AJ496768 AJ496768
INRA115
bm9146
POMC rs29018618
rs29013769
rs29023108
rs29016063 rs29016064
BL1048
DIK28
BMS1048
IDVGA3
rs29010038
AAFC02043452 rs29011281
rs29011282 rs29011283
rs29011284 rs29018852
rs29024646
AAFC02052494 AAFC02052494
AAFC02052494 AAFC02104775
rs29022184 rs29022183
KIAA0235
BES4_Contig379_1101 rs29011068
AAFC02026307
rs29014077
HUJV174
BM746
rs29012043 rs29012044
rs29017413
rs29022203
ODC1
rs29011484
rs29012355
RRM2
BMS460
TGLA436
OarCP34
IDVGA63
rs29013825
rs29014607 rs29014608
rs29017024 rs29017025
JAB7
GGTA1
AAFC02117669 AAFC02117669
rs29014079
rs29013676
rs29012777 rs29012778
AAFC02105663
AAFC02109146 rs29025976
rs29013233
rs29015347
rs29013201 rs29013203
rs29013205
BES2_Contig439_494 BES2_Contig439_567
rs29013563 rs29009915
rs29009914 rs29009913
NR5A1
BMS352
STXBP1
AAFC02064159 AAFC02064159
NW_928413 BES8_Contig574_953
ETH9
BMS2208
rs29024068
rs29015335
rs29025911
rs29018273
BMS1350
rs29009766
LGB
rs29027544
rs29025299
E228D18-38734 rs29002134
rs29015207
NW_973962
rs29017167
rs29016422 rs29016423
rs29012216 rs29012217
UMC
rs29020725
rs29012894 rs29016518
rs29025220 rs29025906
rs29025373 rs17872208
rs29014488 rs29009731
rs29009730 rs29027869
rs29009732 rs29009734
rs29009737 rs29013436
rs29019487
rs29023278 rs29009663
rs29011156
rs29017131
rs29014007 rs29016160
rs29017375 rs29015870
rs29025726 rs29018553
rs29026141
rs29026139
rs29009866
AAFC02111268
AAFC02008683 rs29013299
rs29013297
rs29009683 rs29011499
rs29014004 rs29010503
rs29010501 rs29010505
AAFC02048781 AAFC02048781
rs29013768 rs29014925
rs29023513 rs29011971
rs29013419 rs29013420
rs29019813 AAFC02083777
rs29027341
AAFC02007820 rs29027434
rs29026843 rs29009815
rs29009719 AAFC02100849
rs29018818 rs29016455
AAFC02094245
rs29015807 rs29015811
rs29023466 rs29020107
rs29022207 rs29016064
rs29010798 rs29010799
POMC rs29023108
rs29013769
rs29022183 rs29022184
rs29018852 AAFC02043452
rs29024646 rs29011284
rs29011283 rs29011281
AAFC02052494
rs29011068 AAFC02026307
rs29012044
rs29011484
rs29022203
rs29012355
AAFC02117669 AAFC02117669
rs29013825 rs29017024
rs29013203 rs29009915
rs29009914 rs29009913
rs29013563
rs29012777 rs29012778
AAFC02109146 rs29025976
AAFC02064159 AAFC02064159
rs29025911 rs29018273
rs29015335

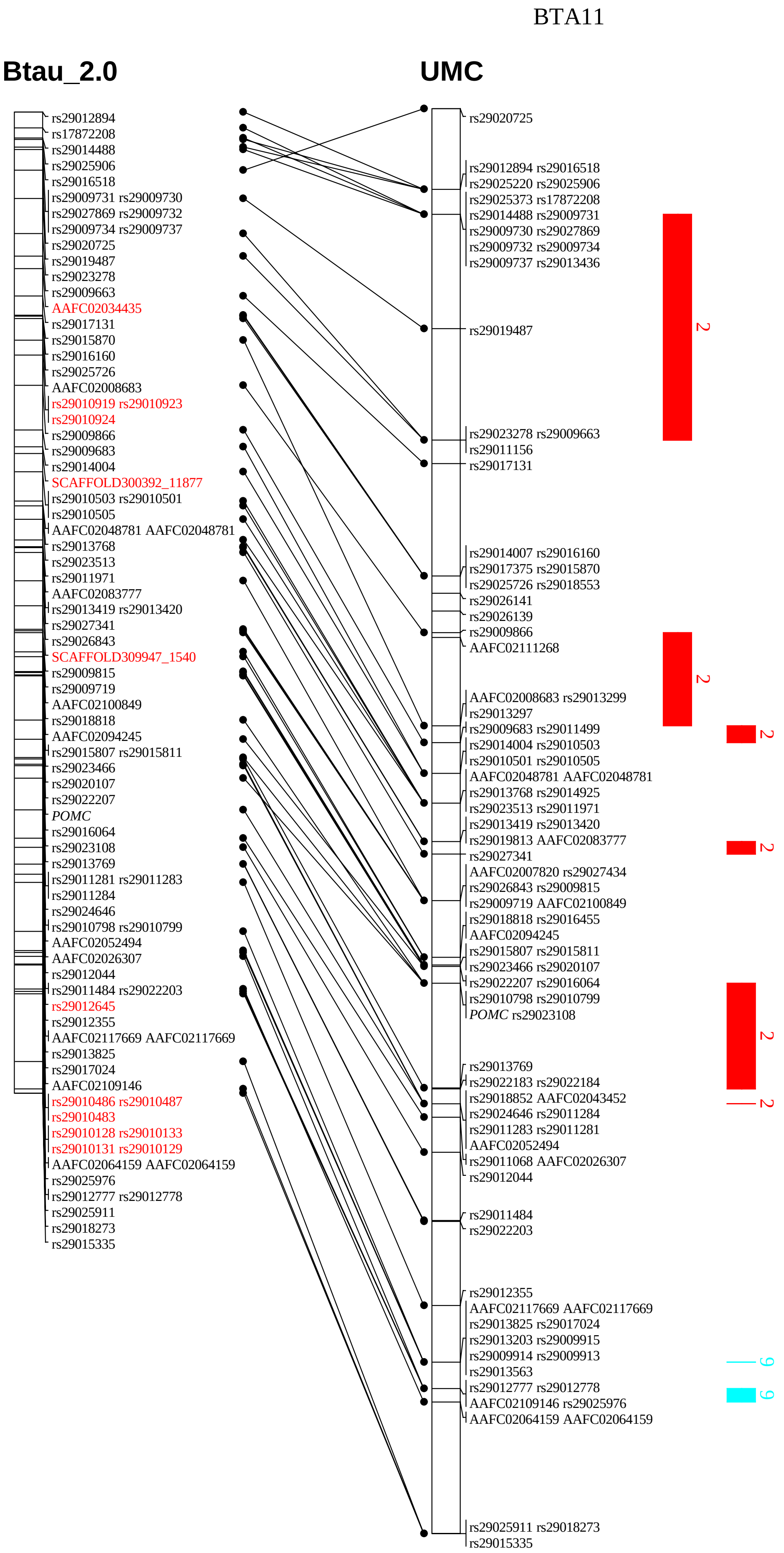
BTA11
Btau_2.0
UMC
rs29012894
rs17872208
rs29014488
rs29025906
rs29016518
rs29009731 rs29009730
rs29027869 rs29009732
rs29009734 rs29009737
rs29020725
rs29019487
rs29023278
rs29009663
AAFC02034435
rs29017131
rs29015870
rs29016160
rs29025726
AAFC02008683
rs29010919 rs29010923
rs29010924
rs29009866
rs29009683
rs29014004
SCAFFOLD300392_11877
rs29010503 rs29010501
rs29010505
AAFC02048781 AAFC02048781
rs29013768
rs29023513
rs29011971
AAFC02083777
rs29013419 rs29013420
rs29027341
rs29026843
SCAFFOLD309947_1540
rs29009815
rs29009719
AAFC02100849
rs29018818
AAFC02094245
rs29015807 rs29015811
rs29023466
rs29020107
rs29022207
POMC
rs29016064
rs29023108
rs29013769
rs29011281 rs29011283
rs29011284
rs29024646
rs29010798 rs29010799
AAFC02052494
AAFC02026307
rs29012044
rs29011484 rs29022203
rs29012645
rs29012355
AAFC02117669 AAFC02117669
rs29013825
rs29017024
AAFC02109146
rs29010486 rs29010487
rs29010483
rs29010128 rs29010133
rs29010131 rs29010129
AAFC02064159 AAFC02064159
rs29025976
rs29012777 rs29012778
rs29025911
rs29018273
rs29015335
rs29020725
rs29012894 rs29016518
rs29025220 rs29025906
rs29025373 rs17872208
rs29014488 rs29009731
rs29009730 rs29027869
rs29009732 rs29009734
rs29009737 rs29013436
rs29019487
rs29023278 rs29009663
rs29011156
rs29017131
rs29014007 rs29016160
rs29017375 rs29015870
rs29025726 rs29018553
rs29026141
rs29026139
rs29009866
AAFC02111268
AAFC02008683 rs29013299
rs29013297
rs29009683 rs29011499
rs29014004 rs29010503
rs29010501 rs29010505
AAFC02048781 AAFC02048781
rs29013768 rs29014925
rs29023513 rs29011971
rs29013419 rs29013420
rs29019813 AAFC02083777
rs29027341
AAFC02007820 rs29027434
rs29026843 rs29009815
rs29009719 AAFC02100849
rs29018818 rs29016455
AAFC02094245
rs29015807 rs29015811
rs29023466 rs29020107
rs29022207 rs29016064
rs29010798 rs29010799
POMC rs29023108
rs29013769
rs29022183 rs29022184
rs29018852 AAFC02043452
rs29024646 rs29011284
rs29011283 rs29011281
AAFC02052494
rs29011068 AAFC02026307
rs29012044
rs29011484
rs29022203
rs29012355
AAFC02117669 AAFC02117669
rs29013825 rs29017024
rs29013203 rs29009915
rs29009914 rs29009913
rs29013563
rs29012777 rs29012778
AAFC02109146 rs29025976
AAFC02064159 AAFC02064159
rs29025911 rs29018273
rs29015335
2
2
2
2
2
2
9
9

BTA12

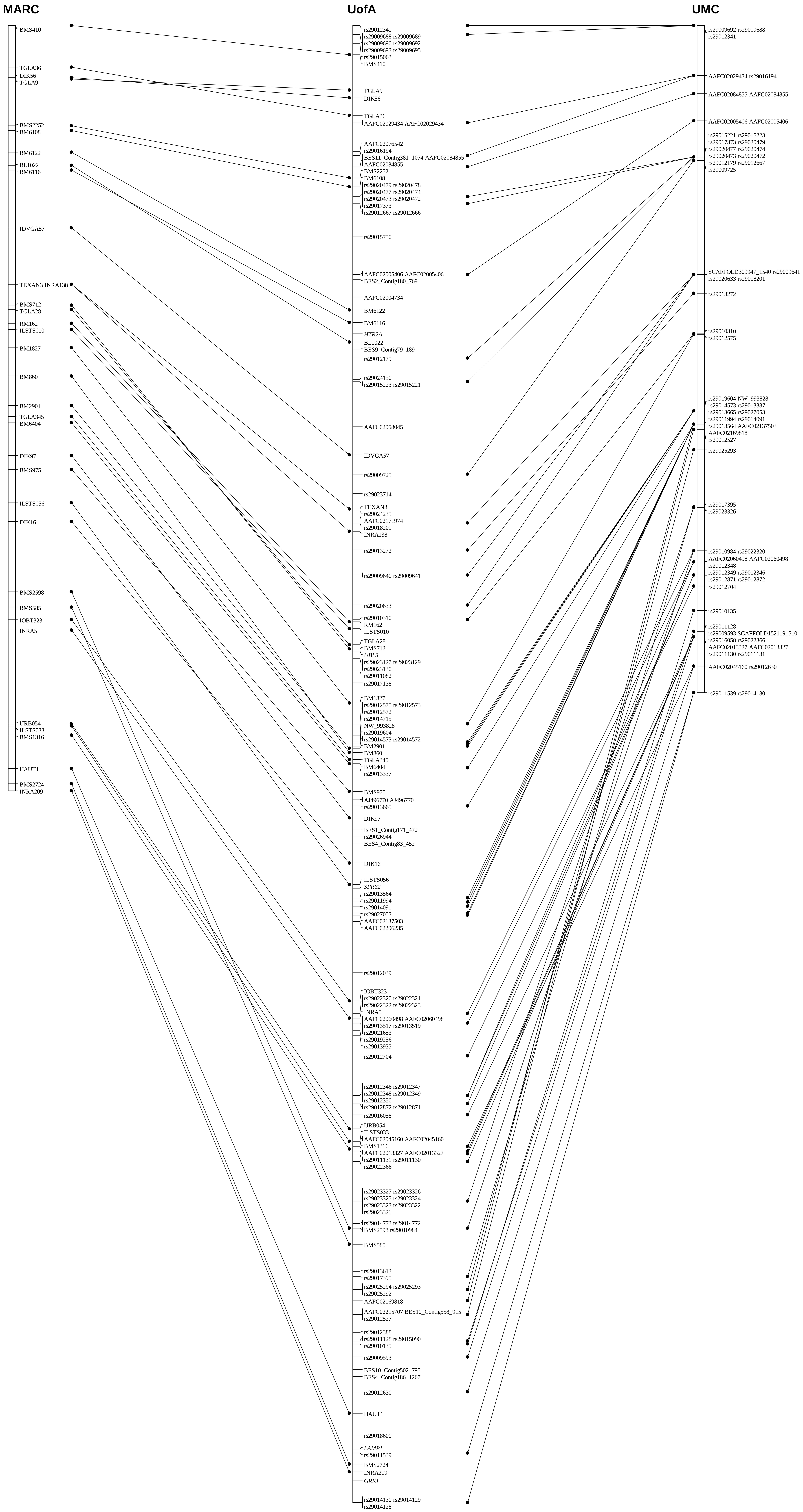
MARC
BMS410
TGLA36
DIK56
TGLA9
BMS2252
BM6108
BM6122
BL1022
BM6116
IDVGA57
TEXAN3 INRA138
BMS712
TGLA28
RM162
ILSTS010
BM1827
BM860
BM2901
TGLA345
BM6404
DIK97
BMS975
ILSTS056
DIK16
BMS2598
BMS585
IOBT323
INRA5
URB054
ILSTS033
BMS1316
HAUT1
BMS2724
INRA209
UofA
rs29012341
rs29009688 rs29009689
rs29009690 rs29009692
rs29009693 rs29009695
rs29015063
BMS410
TGLA9
DIK56
TGLA36
AAFC02029434 AAFC02029434
AAFC02076542
rs29016194
BES11_Contig381_1074 AAFC02084855
AAFC02084855
BMS2252
BM6108
rs29020479 rs29020478
rs29020477 rs29020474
rs29020473 rs29020472
rs29017373
rs29012667 rs29012666
rs29015750
AAFC02005406 AAFC02005406
BES2_Contig180_769
AAFC02004734
BM6122
BM6116
HTR2A
BL1022
BES9_Contig79_189
rs29012179
rs29024150
rs29015223 rs29015221
AAFC02058045
IDVGA57
rs29009725
rs29023714
TEXAN3
rs29024235
AAFC02171974
rs29018201
INRA138
rs29013272
rs29009640 rs29009641
rs29020633
rs29010310
RM162
ILSTS010
TGLA28
BMS712
UBL3
rs29023127 rs29023129
rs29023130
rs29011082
rs29017138
BM1827
rs29012575 rs29012573
rs29012572
rs29014715
NW_993828
rs29019604
rs29014573 rs29014572
BM2901
BM860
TGLA345
BM6404
rs29013337
BMS975
AJ496770 AJ496770
rs29013665
DIK97
BES1_Contig171_472
rs29026944
BES4_Contig83_452
DIK16
ILSTS056
SPRY2
rs29013564
rs29011994
rs29014091
rs29027053
AAFC02137503
AAFC02206235
rs29012039
IOBT323
rs29022320 rs29022321
rs29022322 rs29022323
INRA5
AAFC02060498 AAFC02060498
rs29013517 rs29013519
rs29021653
rs29019256
rs29013935
rs29012704
rs29012346 rs29012347
rs29012348 rs29012349
rs29012350
rs29012872 rs29012871
rs29016058
URB054
ILSTS033
AAFC02045160 AAFC02045160
BMS1316
AAFC02013327 AAFC02013327
rs29011131 rs29011130
rs29022366
rs29023327 rs29023326
rs29023325 rs29023324
rs29023323 rs29023322
rs29023321
rs29014773 rs29014772
BMS2598 rs29010984
BMS585
rs29013612
rs29017395
rs29025294 rs29025293
rs29025292
AAFC02169818
AAFC02215707 BES10_Contig558_915
rs29012527
rs29012388
rs29011128 rs29015090
rs29010135
rs29009593
BES10_Contig502_795
BES4_Contig186_1267
rs29012630
HAUT1
rs29018600
LAMP1
rs29011539
BMS2724
INRA209
GRK1
rs29014130 rs29014129
rs29014128
UMC
rs29009692 rs29009688
rs29012341
AAFC02029434 rs29016194
AAFC02084855 AAFC02084855
AAFC02005406 AAFC02005406
rs29015221 rs29015223
rs29017373 rs29020479
rs29020477 rs29020474
rs29020473 rs29020472
rs29012179 rs29012667
rs29009725
SCAFFOLD309947_1540 rs29009641
rs29020633 rs29018201
rs29013272
rs29010310
rs29012575
rs29019604 NW_993828
rs29014573 rs29013337
rs29013665 rs29027053
rs29011994 rs29014091
rs29013564 AAFC02137503
AAFC02169818
rs29012527
rs29025293
rs29017395
rs29023326
rs29010984 rs29022320
AAFC02060498 AAFC02060498
rs29012348
rs29012349 rs29012346
rs29012871 rs29012872
rs29012704
rs29010135
rs29011128
rs29009593 SCAFFOLD152119_510
rs29016058 rs29022366
AAFC02013327 AAFC02013327
rs29011130 rs29011131
AAFC02045160 rs29012630
rs29011539 rs29014130

BTA12

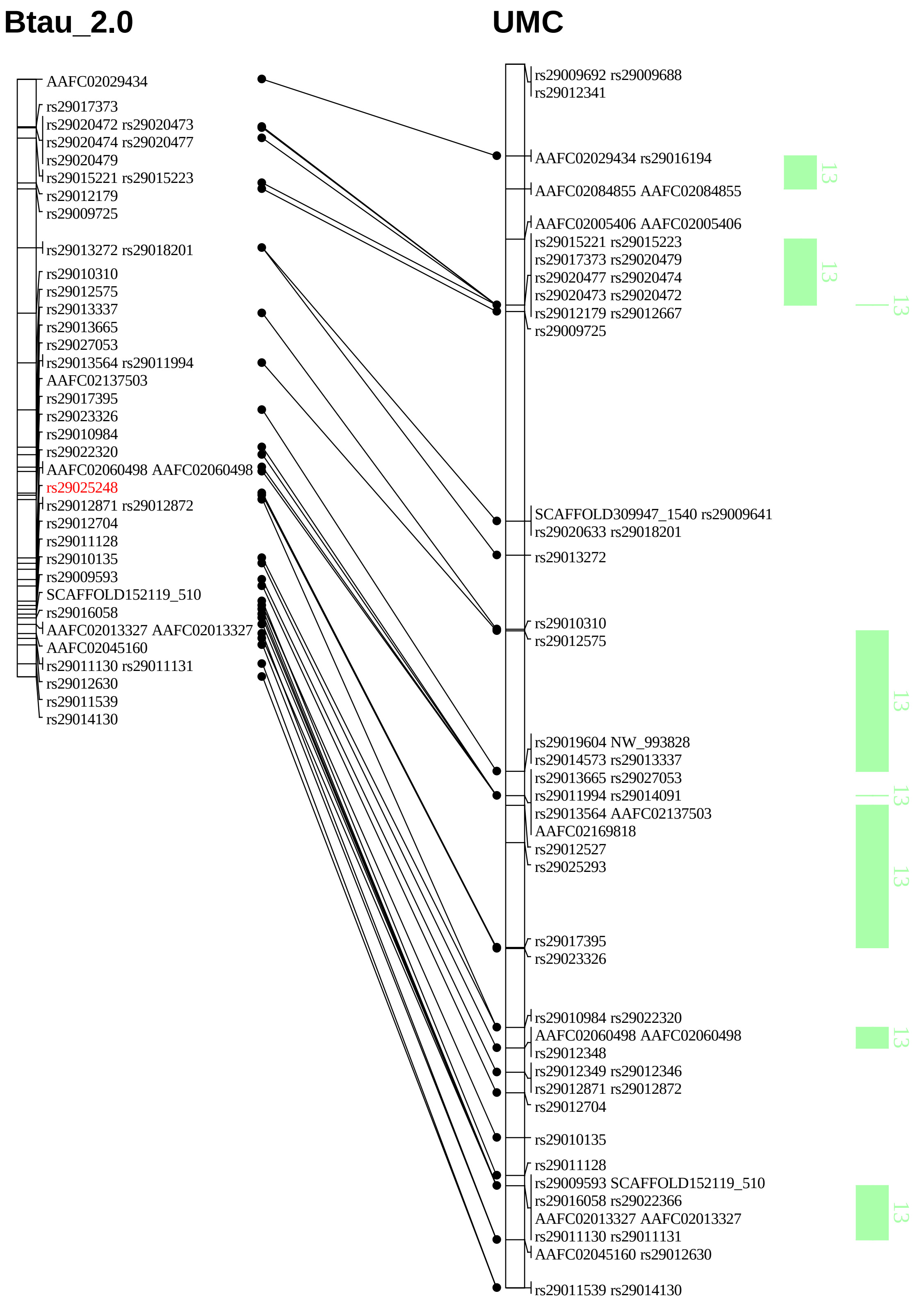

Btau_2.0
UMC
AAFC02029434
rs29017373
rs29020472 rs29020473
rs29020474 rs29020477
rs29020479
rs29015221 rs29015223
rs29012179
rs29009725
rs29013272 rs29018201
rs29010310
rs29012575
rs29013337
rs29013665
rs29027053
rs29013564 rs29011994
AAFC02137503
rs29017395
rs29023326
rs29010984
rs29022320
AAFC02060498 AAFC02060498
rs29025248
rs29012871 rs29012872
rs29012704
rs29011128
rs29010135
rs29009593
SCAFFOLD152119_510
rs29016058
AAFC02013327 AAFC02013327
AAFC02045160
rs29011130 rs29011131
rs29012630
rs29011539
rs29014130
rs29009692 rs29009688
rs29012341
AAFC02029434 rs29016194
AAFC02084855 AAFC02084855
AAFC02005406 AAFC02005406
rs29015221 rs29015223
rs29017373 rs29020479
rs29020477 rs29020474
rs29020473 rs29020472
rs29012179 rs29012667
rs29009725
SCAFFOLD309947_1540 rs29009641
rs29020633 rs29018201
rs29013272
rs29010310
rs29012575
rs29019604 NW_993828
rs29014573 rs29013337
rs29013665 rs29027053
rs29011994 rs29014091
rs29013564 AAFC02137503
AAFC02169818
rs29012527
rs29025293
rs29017395
rs29023326
rs29010984 rs29022320
AAFC02060498 AAFC02060498
rs29012348
rs29012349 rs29012346
rs29012871 rs29012872
rs29012704
rs29010135
rs29011128
rs29009593 SCAFFOLD152119_510
rs29016058 rs29022366
AAFC02013327 AAFC02013327
rs29011130 rs29011131
AAFC02045160 rs29012630
rs29011539 rs29014130
13
13
13
13
13
13
13
13

BTA13

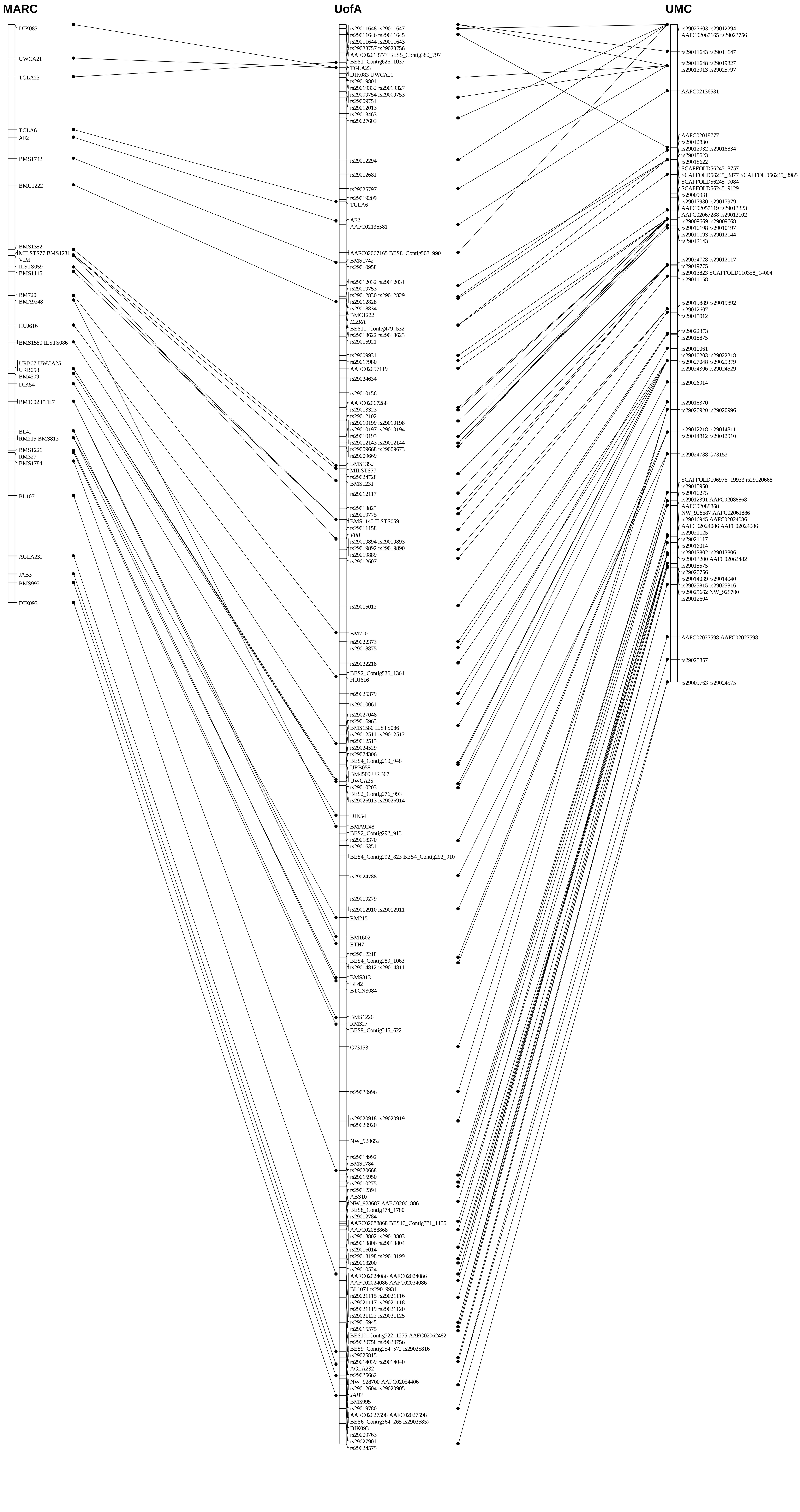

MARC
DIK083
UWCA21
TGLA23
TGLA6
AF2
BMS1742
BMC1222
BMS1352
MILSTS77 BMS1231
VIM
ILSTS059
BMS1145
BM720
BMA9248
HUJ616
BMS1580 ILSTS086
URB07 UWCA25
URB058
BM4509
DIK54
BM1602 ETH7
BL42
RM215 BMS813
BMS1226
RM327
BMS1784
BL1071
AGLA232
JAB3
BMS995
DIK093
UofA
rs29011648 rs29011647
rs29011646 rs29011645
rs29011644 rs29011643
rs29023757 rs29023756
AAFC02018777 BES5_Contig380_797
BES1_Contig626_1037
TGLA23
DIK083 UWCA21
rs29019801
rs29019332 rs29019327
rs29009754 rs29009753
rs29009751
rs29012013
rs29013463
rs29027603
rs29012294
rs29012681
rs29025797
rs29019209
TGLA6
AF2
AAFC02136581
AAFC02067165 BES8_Contig508_990
BMS1742
rs29010958
rs29012032 rs29012031
rs29019753
rs29012830 rs29012829
rs29012828
rs29018834
BMC1222
IL2RA
BES11_Contig479_532
rs29018622 rs29018623
rs29015921
rs29009931
rs29017980
AAFC02057119
rs29024634
rs29010156
AAFC02067288
rs29013323
rs29012102
rs29010199 rs29010198
rs29010197 rs29010194
rs29010193
rs29012143 rs29012144
rs29009668 rs29009673
rs29009669
BMS1352
MILSTS77
rs29024728
BMS1231
rs29012117
rs29013823
rs29019775
BMS1145 ILSTS059
rs29011158
VIM
rs29019894 rs29019893
rs29019892 rs29019890
rs29019889
rs29012607
rs29015012
BM720
rs29022373
rs29018875
rs29022218
BES2_Contig526_1364
HUJ616
rs29025379
rs29010061
rs29027048
rs29016963
BMS1580 ILSTS086
rs29012511 rs29012512
rs29012513
rs29024529
rs29024306
BES4_Contig210_948
URB058
BM4509 URB07
UWCA25
rs29010203
BES2_Contig276_993
rs29026913 rs29026914
DIK54
BMA9248
BES2_Contig292_913
rs29018370
rs29016351
BES4_Contig292_823 BES4_Contig292_910
rs29024788
rs29019279
rs29012910 rs29012911
RM215
BM1602
ETH7
rs29012218
BES4_Contig289_1063
rs29014812 rs29014811
BMS813
BL42
BTCN3084
BMS1226
RM327
BES9_Contig345_622
G73153
rs29020996
rs29020918 rs29020919
rs29020920
NW_928652
rs29014992
BMS1784
rs29020668
rs29015950
rs29010275
rs29012391
ABS10
NW_928687 AAFC02061886
BES8_Contig474_1780
rs29012784
AAFC02088868 BES10_Contig781_1135
AAFC02088868
rs29013802 rs29013803
rs29013806 rs29013804
rs29016014
rs29013198 rs29013199
rs29013200
rs29010524
AAFC02024086 AAFC02024086
AAFC02024086 AAFC02024086
BL1071 rs29019931
rs29021115 rs29021116
rs29021117 rs29021118
rs29021119 rs29021120
rs29021122 rs29021125
rs29016945
rs29015575
BES10_Contig722_1275 AAFC02062482
rs29020758 rs29020756
BES9_Contig254_572 rs29025816
rs29025815
rs29014039 rs29014040
AGLA232
rs29025662
NW_928700 AAFC02054406
rs29012604 rs29020905
JAB3
BMS995
rs29019780
AAFC02027598 AAFC02027598
BES6_Contig364_265 rs29025857
DIK093
rs29009763
rs29027901
rs29024575
UMC
rs29027603 rs29012294
AAFC02067165 rs29023756
rs29011643 rs29011647
rs29011648 rs29019327
rs29012013 rs29025797
AAFC02136581
AAFC02018777
rs29012830
rs29012032 rs29018834
rs29018623
rs29018622
SCAFFOLD56245_8757
SCAFFOLD56245_8877 SCAFFOLD56245_8985
SCAFFOLD56245_9084
SCAFFOLD56245_9129
rs29009931
rs29017980 rs29017979
AAFC02057119 rs29013323
AAFC02067288 rs29012102
rs29009669 rs29009668
rs29010198 rs29010197
rs29010193 rs29012144
rs29012143
rs29024728 rs29012117
rs29019775
rs29013823 SCAFFOLD110358_14004
rs29011158
rs29019889 rs29019892
rs29012607
rs29015012
rs29022373
rs29018875
rs29010061
rs29010203 rs29022218
rs29027048 rs29025379
rs29024306 rs29024529
rs29026914
rs29018370
rs29020920 rs29020996
rs29012218 rs29014811
rs29014812 rs29012910
rs29024788 G73153
SCAFFOLD106976_19933 rs29020668
rs29015950
rs29010275
rs29012391 AAFC02088868
AAFC02088868
NW_928687 AAFC02061886
rs29016945 AAFC02024086
AAFC02024086 AAFC02024086
rs29021125
rs29021117
rs29016014
rs29013802 rs29013806
rs29013200 AAFC02062482
rs29015575
rs29020756
rs29014039 rs29014040
rs29025815 rs29025816
rs29025662 NW_928700
rs29012604
AAFC02027598 AAFC02027598
rs29025857
rs29009763 rs29024575

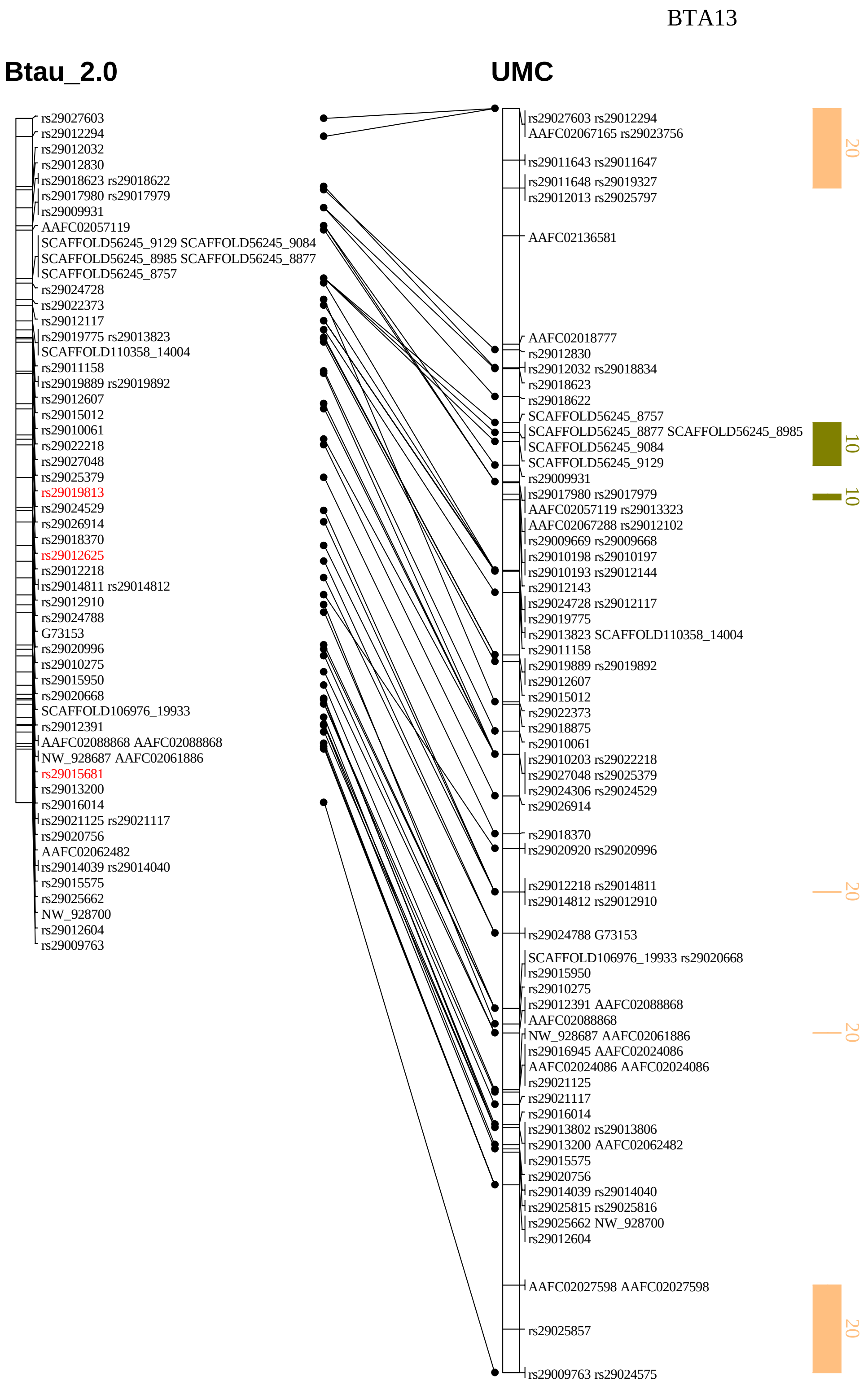
BTA13
Btau_2.0
UMC
rs29027603
rs29012294
rs29012032
rs29012830
rs29018623 rs29018622
rs29017980 rs29017979
rs29009931
AAFC02057119
SCAFFOLD56245_9129 SCAFFOLD56245_9084
SCAFFOLD56245_8985 SCAFFOLD56245_8877
SCAFFOLD56245_8757
rs29024728
rs29022373
rs29012117
rs29019775 rs29013823
SCAFFOLD110358_14004
rs29011158
rs29019889 rs29019892
rs29012607
rs29015012
rs29010061
rs29022218
rs29027048
rs29025379
rs29019813
rs29024529
rs29026914
rs29018370
rs29012625
rs29012218
rs29014811 rs29014812
rs29012910
rs29024788
G73153
rs29020996
rs29010275
rs29015950
rs29020668
SCAFFOLD106976_19933
rs29012391
AAFC02088868 AAFC02088868
NW_928687 AAFC02061886
rs29015681
rs29013200
rs29016014
rs29021125 rs29021117
rs29020756
AAFC02062482
rs29014039 rs29014040
rs29015575
rs29025662
NW_928700
rs29012604
rs29009763
rs29027603 rs29012294
AAFC02067165 rs29023756
rs29011643 rs29011647
rs29011648 rs29019327
rs29012013 rs29025797
AAFC02136581
AAFC02018777
rs29012830
rs29012032 rs29018834
rs29018623
rs29018622
SCAFFOLD56245_8757
SCAFFOLD56245_8877 SCAFFOLD56245_8985
SCAFFOLD56245_9084
SCAFFOLD56245_9129
rs29009931
rs29017980 rs29017979
AAFC02057119 rs29013323
AAFC02067288 rs29012102
rs29009669 rs29009668
rs29010198 rs29010197
rs29010193 rs29012144
rs29012143
rs29024728 rs29012117
rs29019775
rs29013823 SCAFFOLD110358_14004
rs29011158
rs29019889 rs29019892
rs29012607
rs29015012
rs29022373
rs29018875
rs29010061
rs29010203 rs29022218
rs29027048 rs29025379
rs29024306 rs29024529
rs29026914
rs29018370
rs29020920 rs29020996
rs29012218 rs29014811
rs29014812 rs29012910
rs29024788 G73153
SCAFFOLD106976_19933 rs29020668
rs29015950
rs29010275
rs29012391 AAFC02088868
AAFC02088868
NW_928687 AAFC02061886
rs29016945 AAFC02024086
AAFC02024086 AAFC02024086
rs29021125
rs29021117
rs29016014
rs29013802 rs29013806
rs29013200 AAFC02062482
rs29015575
rs29020756
rs29014039 rs29014040
rs29025815 rs29025816
rs29025662 NW_928700
rs29012604
AAFC02027598 AAFC02027598
rs29025857
rs29009763 rs29024575
20
10
10
20
20
20

BTA14

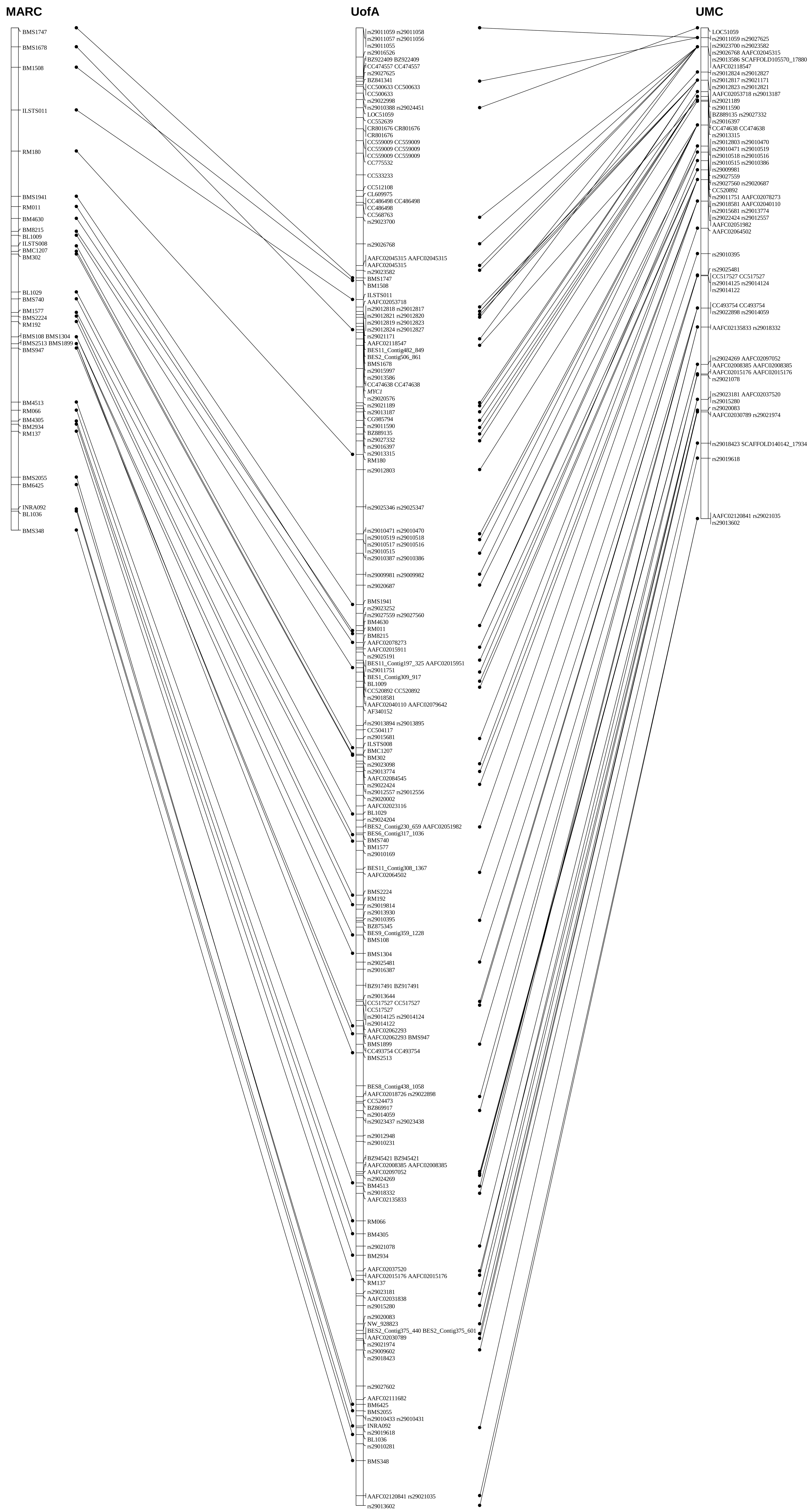
MARC
BMS1747
BMS1678
BM1508
ILSTS011
RM180
BMS1941
RM011
BM4630
BM8215
BL1009
ILSTS008
BMC1207
BM302
BL1029
BMS740
BM1577
BMS2224
RM192
BMS108 BMS1304
BMS2513 BMS1899
BMS947
BM4513
RM066
BM4305
BM2934
RM137
BMS2055
BM6425
INRA092
BL1036
BMS348
UofA
rs29011059 rs29011058
rs29011057 rs29011056
rs29011055
rs29016526
BZ922409 BZ922409
CC474557 CC474557
rs29027625
BZ841341
CC500633 CC500633
CC500633
rs29022998
rs29010388 rs29024451
LOC51059
CC552639
CR801676 CR801676
CR801676
CC559009 CC559009
CC559009 CC559009
CC559009 CC559009
CC775532
CC533233
CC512108
CL609975
CC486498 CC486498
CC486498
CC568763
rs29023700
rs29026768
AAFC02045315 AAFC02045315
AAFC02045315
rs29023582
BMS1747
BM1508
ILSTS011
AAFC02053718
rs29012818 rs29012817
rs29012821 rs29012820
rs29012819 rs29012823
rs29012824 rs29012827
rs29021171
AAFC02118547
BES11_Contig482_849
BES2_Contig506_861
BMS1678
rs29015997
rs29013586
CC474638 CC474638
MYC1
rs29020576
rs29021189
rs29013187
CG985794
rs29011590
BZ889135
rs29027332
rs29016397
rs29013315
RM180
rs29012803
rs29025346 rs29025347
rs29010471 rs29010470
rs29010519 rs29010518
rs29010517 rs29010516
rs29010515
rs29010387 rs29010386
rs29009981 rs29009982
rs29020687
BMS1941
rs29023252
rs29027559 rs29027560
BM4630
RM011
BM8215
AAFC02078273
AAFC02015911
rs29025191
BES11_Contig197_325 AAFC02015951
rs29011751
BES1_Contig309_917
BL1009
CC520892 CC520892
rs29018581
AAFC02040110 AAFC02079642
AF340152
rs29013894 rs29013895
CC504117
rs29015681
ILSTS008
BMC1207
BM302
rs29023098
rs29013774
AAFC02084545
rs29022424
rs29012557 rs29012556
rs29020002
AAFC02023116
BL1029
rs29024204
BES2_Contig230_659 AAFC02051982
BES6_Contig317_1036
BMS740
BM1577
rs29010169
BES11_Contig308_1367
AAFC02064502
BMS2224
RM192
rs29019814
rs29013930
rs29010395
BZ875345
BES9_Contig359_1228
BMS108
BMS1304
rs29025481
rs29016387
BZ917491 BZ917491
rs29013644
CC517527 CC517527
CC517527
rs29014125 rs29014124
rs29014122
AAFC02062293
AAFC02062293 BMS947
BMS1899
CC493754 CC493754
BMS2513
BES8_Contig438_1058
AAFC02018726 rs29022898
CC524473
BZ869917
rs29014059
rs29023437 rs29023438
rs29012948
rs29010231
BZ945421 BZ945421
AAFC02008385 AAFC02008385
AAFC02097052
rs29024269
BM4513
rs29018332
AAFC02135833
RM066
BM4305
rs29021078
BM2934
AAFC02037520
AAFC02015176 AAFC02015176
RM137
rs29023181
AAFC02031838
rs29015280
rs29020083
NW_928823
BES2_Contig375_440 BES2_Contig375_601
AAFC02030789
rs29021974
rs29009602
rs29018423
rs29027602
AAFC02111682
BM6425
BMS2055
rs29010433 rs29010431
INRA092
rs29019618
BL1036
rs29010281
BMS348
AAFC02120841 rs29021035
rs29013602
UMC
LOC51059
rs29011059 rs29027625
rs29023700 rs29023582
rs29026768 AAFC02045315
rs29013586 SCAFFOLD105570_17880
AAFC02118547
rs29012824 rs29012827
rs29012817 rs29021171
rs29012823 rs29012821
AAFC02053718 rs29013187
rs29021189
rs29011590
BZ889135 rs29027332
rs29016397
CC474638 CC474638
rs29013315
rs29012803 rs29010470
rs29010471 rs29010519
rs29010518 rs29010516
rs29010515 rs29010386
rs29009981
rs29027559
rs29027560 rs29020687
CC520892
rs29011751 AAFC02078273
rs29018581 AAFC02040110
rs29015681 rs29013774
rs29022424 rs29012557
AAFC02051982
AAFC02064502
rs29010395
rs29025481
CC517527 CC517527
rs29014125 rs29014124
rs29014122
CC493754 CC493754
rs29022898 rs29014059
AAFC02135833 rs29018332
rs29024269 AAFC02097052
AAFC02008385 AAFC02008385
AAFC02015176 AAFC02015176
rs29021078
rs29023181 AAFC02037520
rs29015280
rs29020083
AAFC02030789 rs29021974
rs29018423 SCAFFOLD140142_17934
rs29019618
AAFC02120841 rs29021035
rs29013602

BTA14

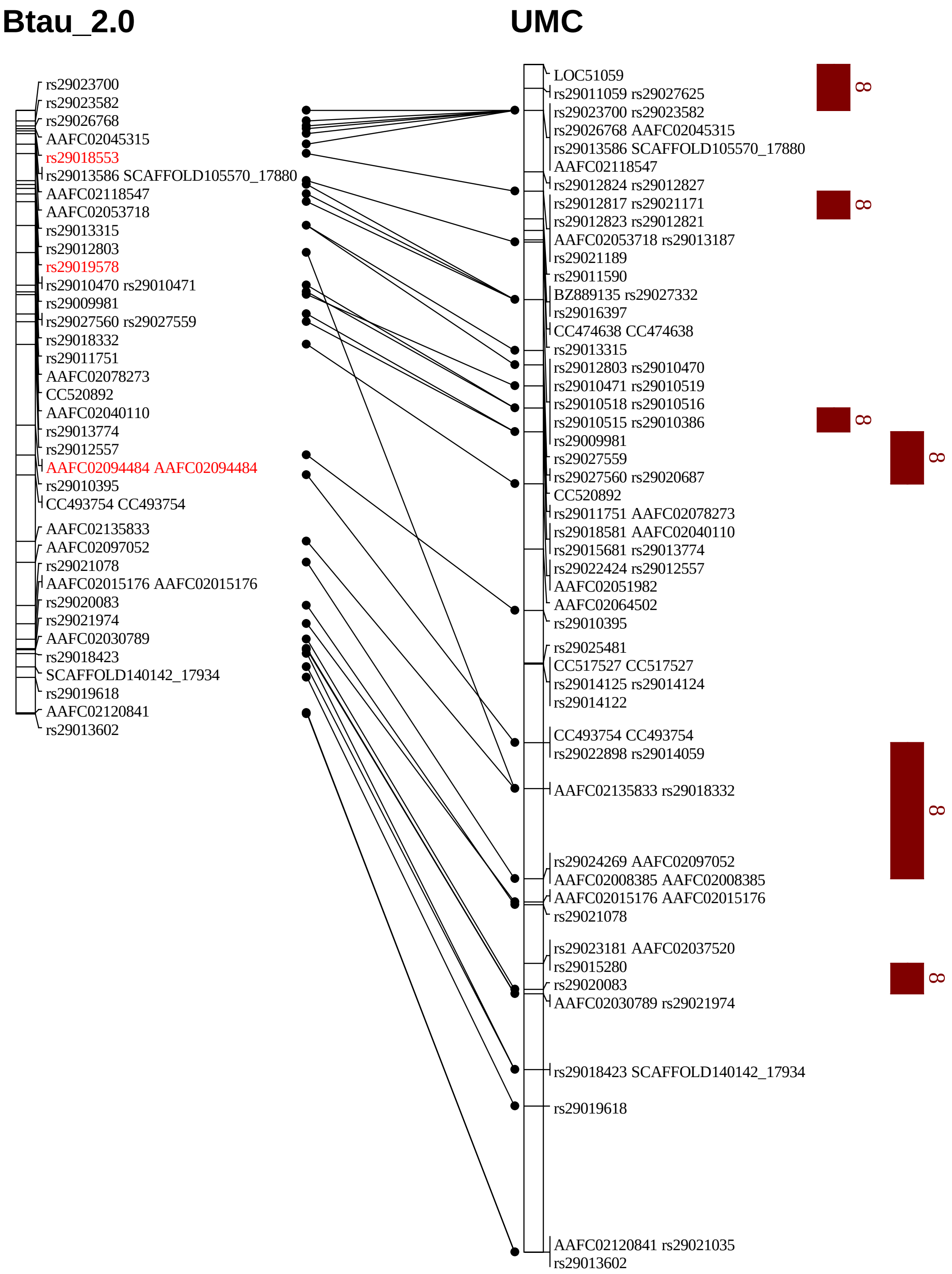

Btau_2.0
UMC
rs29023700
rs29023582
rs29026768
AAFC02045315
rs29018553
rs29013586 SCAFFOLD105570_17880
AAFC02118547
AAFC02053718
rs29013315
rs29012803
rs29019578
rs29010470 rs29010471
rs29009981
rs29027560 rs29027559
rs29018332
rs29011751
AAFC02078273
CC520892
AAFC02040110
rs29013774
rs29012557
AAFC02094484 AAFC02094484
rs29010395
CC493754 CC493754
AAFC02135833
AAFC02097052
rs29021078
AAFC02015176 AAFC02015176
rs29020083
rs29021974
AAFC02030789
rs29018423
SCAFFOLD140142_17934
rs29019618
AAFC02120841
rs29013602
LOC51059
rs29011059 rs29027625
rs29023700 rs29023582
rs29026768 AAFC02045315
rs29013586 SCAFFOLD105570_17880
AAFC02118547
rs29012824 rs29012827
rs29012817 rs29021171
rs29012823 rs29012821
AAFC02053718 rs29013187
rs29021189
rs29011590
BZ889135 rs29027332
rs29016397
CC474638 CC474638
rs29013315
rs29012803 rs29010470
rs29010471 rs29010519
rs29010518 rs29010516
rs29010515 rs29010386
rs29009981
rs29027559
rs29027560 rs29020687
CC520892
rs29011751 AAFC02078273
rs29018581 AAFC02040110
rs29015681 rs29013774
rs29022424 rs29012557
AAFC02051982
AAFC02064502
rs29010395
rs29025481
CC517527 CC517527
rs29014125 rs29014124
rs29014122
CC493754 CC493754
rs29022898 rs29014059
AAFC02135833 rs29018332
rs29024269 AAFC02097052
AAFC02008385 AAFC02008385
AAFC02015176 AAFC02015176
rs29021078
rs29023181 AAFC02037520
rs29015280
rs29020083
AAFC02030789 rs29021974
rs29018423 SCAFFOLD140142_17934
rs29019618
AAFC02120841 rs29021035
rs29013602
8
8
8
8
8
8

BTA15

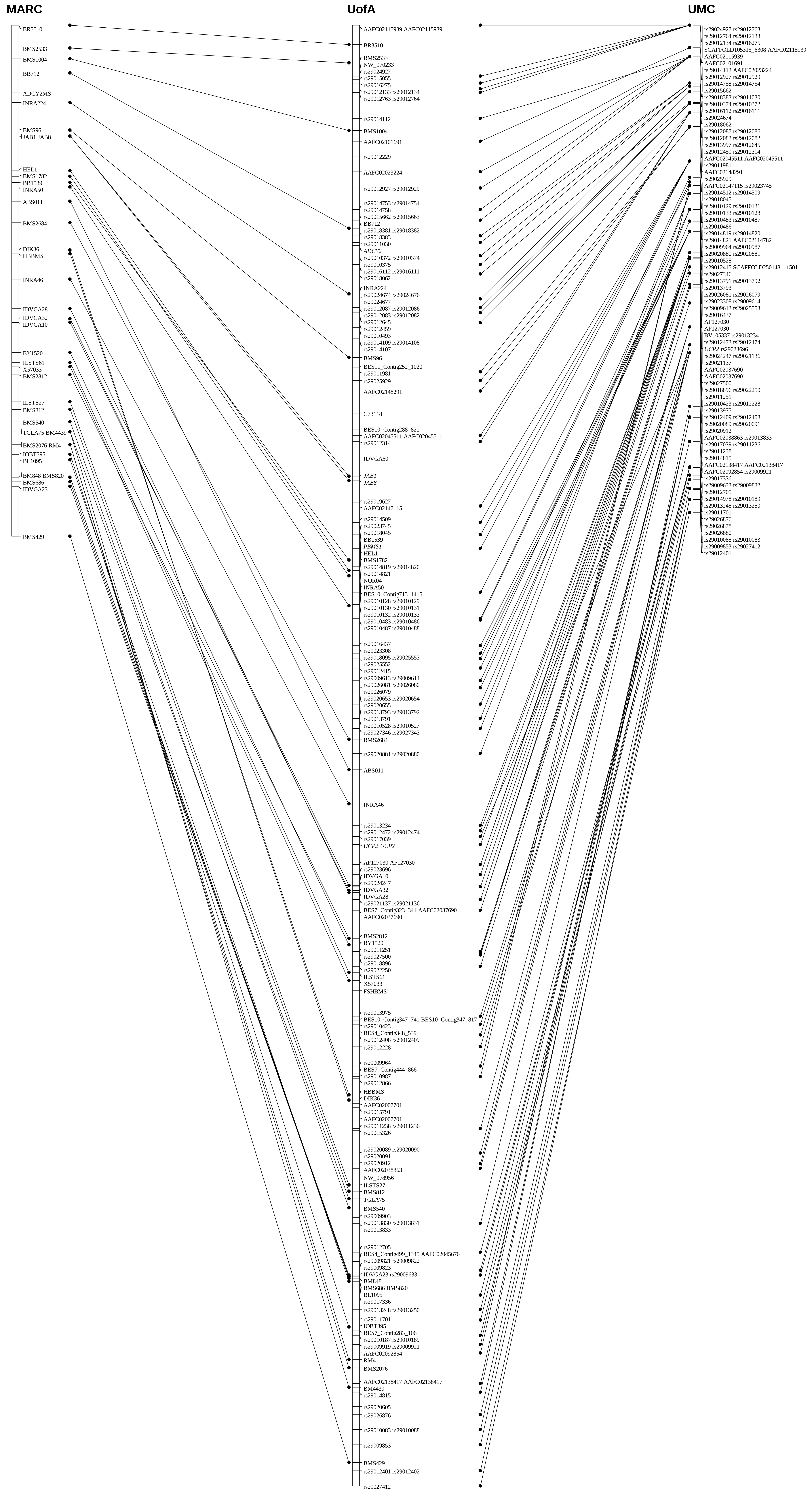
MARC
UofA
UMC
BR3510
BMS2533
BMS1004
BB712
ADCY2MS
INRA224
BMS96
JAB1 JAB8
HEL1
BMS1782
BB1539
INRA50
ABS011
BMS2684
DIK36
HBBMS
INRA46
IDVGA28
IDVGA32
IDVGA10
BY1520
ILSTS61
X57033
BMS2812
ILSTS27
BMS812
BMS540
TGLA75 BM4439
BMS2076 RM4
IOBT395
BL1095
BM848 BMS820
BMS686
IDVGA23
BMS429
AAFC02115939 AAFC02115939
BR3510
BMS2533
NW_970233
rs29024927
rs29015055
rs29016275
rs29012133 rs29012134
rs29012763 rs29012764
rs29014112
BMS1004
AAFC02101691
rs29012229
AAFC02023224
rs29012927 rs29012929
rs29014753 rs29014754
rs29014758
rs29015662 rs29015663
BB712
rs29018381 rs29018382
rs29018383
rs29011030
ADCY2
rs29010372 rs29010374
rs29010375
rs29016112 rs29016111
rs29018062
INRA224
rs29024674 rs29024676
rs29024677
rs29012087 rs29012086
rs29012083 rs29012082
rs29012645
rs29012459
rs29010493
rs29014109 rs29014108
rs29014107
BMS96
BES11_Contig252_1020
rs29011981
rs29025929
AAFC02148291
G73118
BES10_Contig288_821
AAFC02045511 AAFC02045511
rs29012314
IDVGA60
JAB1
JAB8
rs29019627
AAFC02147115
rs29014509
rs29023745
rs29018045
BB1539
PBMS1
HEL1
BMS1782
rs29014819 rs29014820
rs29014821
NOR04
INRA50
BES10_Contig713_1415
rs29010128 rs29010129
rs29010130 rs29010131
rs29010132 rs29010133
rs29010483 rs29010486
rs29010487 rs29010488
rs29016437
rs29023308
rs29018095 rs29025553
rs29025552
rs29012415
rs29009613 rs29009614
rs29026081 rs29026080
rs29026079
rs29020653 rs29020654
rs29020655
rs29013793 rs29013792
rs29013791
rs29010528 rs29010527
rs29027346 rs29027343
BMS2684
rs29020881 rs29020880
ABS011
INRA46
rs29013234
rs29012472 rs29012474
rs29017039
UCP2 UCP2
AF127030 AF127030
rs29023696
IDVGA10
rs29024247
IDVGA32
IDVGA28
rs29021137 rs29021136
BES7_Contig323_341 AAFC02037690
AAFC02037690
BMS2812
BY1520
rs29011251
rs29027500
rs29018896
rs29022250
ILSTS61
X57033
FSHBMS
rs29013975
BES10_Contig347_741 BES10_Contig347_817
rs29010423
BES4_Contig348_539
rs29012408 rs29012409
rs29012228
rs29009964
BES7_Contig444_866
rs29010987
rs29012866
HBBMS
DIK36
AAFC02007701
rs29015791
AAFC02007701
rs29011238 rs29011236
rs29015326
rs29020089 rs29020090
rs29020091
rs29020912
AAFC02038863
NW_978956
ILSTS27
BMS812
TGLA75
BMS540
rs29009903
rs29013830 rs29013831
rs29013833
rs29012705
BES4_Contig499_1345 AAFC02045676
rs29009821 rs29009822
rs29009823
IDVGA23 rs29009633
BM848
BMS686 BMS820
BL1095
rs29017336
rs29013248 rs29013250
rs29011701
IOBT395
BES7_Contig283_106
rs29010187 rs29010189
rs29009919 rs29009921
AAFC02092854
RM4
BMS2076
AAFC02138417 AAFC02138417
BM4439
rs29014815
rs29020605
rs29026876
rs29010083 rs29010088
rs29009853
BMS429
rs29012401 rs29012402
rs29027412
rs29024927 rs29012763
rs29012764 rs29012133
rs29012134 rs29016275
SCAFFOLD105315_6308 AAFC02115939
AAFC02115939
AAFC02101691
rs29014112 AAFC02023224
rs29012927 rs29012929
rs29014758 rs29014754
rs29015662
rs29018383 rs29011030
rs29010374 rs29010372
rs29016112 rs29016111
rs29024674
rs29018062
rs29012087 rs29012086
rs29012083 rs29012082
rs29013997 rs29012645
rs29012459 rs29012314
AAFC02045511 AAFC02045511
rs29011981
AAFC02148291
rs29025929
AAFC02147115 rs29023745
rs29014512 rs29014509
rs29018045
rs29010129 rs29010131
rs29010133 rs29010128
rs29010483 rs29010487
rs29010486
rs29014819 rs29014820
rs29014821 AAFC02114782
rs29009964 rs29010987
rs29020880 rs29020881
rs29010528
rs29012415 SCAFFOLD250148_11501
rs29027346
rs29013791 rs29013792
rs29013793
rs29026081 rs29026079
rs29023308 rs29009614
rs29009613 rs29025553
rs29016437
AF127030
AF127030
BV105337 rs29013234
rs29012472 rs29012474
UCP2 rs29023696
rs29024247 rs29021136
rs29021137
AAFC02037690
AAFC02037690
rs29027500
rs29018896 rs29022250
rs29011251
rs29010423 rs29012228
rs29013975
rs29012409 rs29012408
rs29020089 rs29020091
rs29020912
AAFC02038863 rs29013833
rs29017039 rs29011236
rs29011238
rs29014815
AAFC02138417 AAFC02138417
AAFC02092854 rs29009921
rs29017336
rs29009633 rs29009822
rs29012705
rs29014978 rs29010189
rs29013248 rs29013250
rs29011701
rs29026876
rs29026878
rs29026880
rs29010088 rs29010083
rs29009853 rs29027412
rs29012401

BTA15

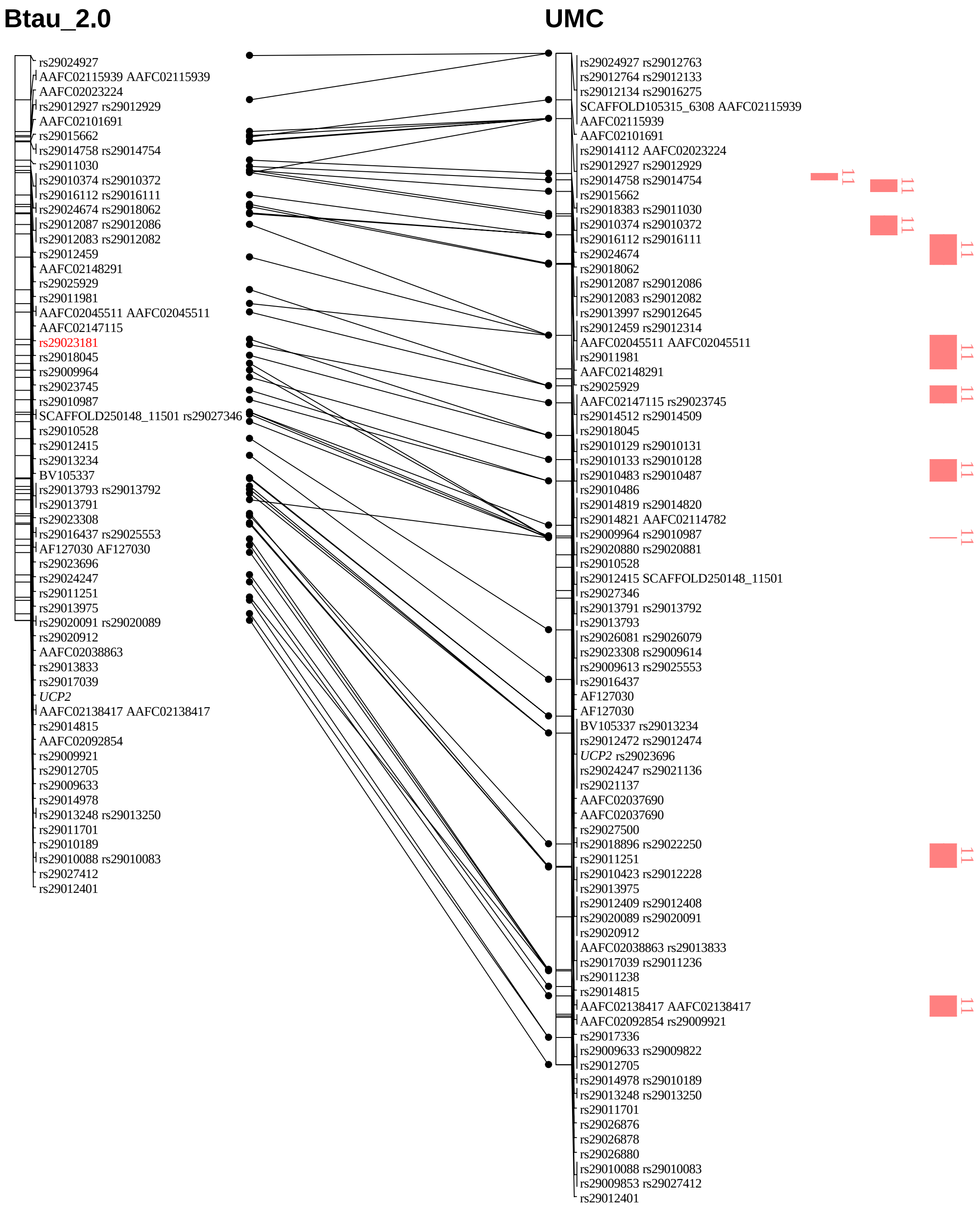
Btau_2.0
UMC
rs29024927
AAFC02115939 AAFC02115939
AAFC02023224
rs29012927 rs29012929
AAFC02101691
rs29015662
rs29014758 rs29014754
rs29011030
rs29010374 rs29010372
rs29016112 rs29016111
rs29024674 rs29018062
rs29012087 rs29012086
rs29012083 rs29012082
rs29012459
AAFC02148291
rs29025929
rs29011981
AAFC02045511 AAFC02045511
AAFC02147115
rs29023181
rs29018045
rs29009964
rs29023745
rs29010987
SCAFFOLD250148_11501 rs29027346
rs29010528
rs29012415
rs29013234
BV105337
rs29013793 rs29013792
rs29013791
rs29023308
rs29016437 rs29025553
AF127030 AF127030
rs29023696
rs29024247
rs29011251
rs29013975
rs29020091 rs29020089
rs29020912
AAFC02038863
rs29013833
rs29017039
UCP2
AAFC02138417 AAFC02138417
rs29014815
AAFC02092854
rs29009921
rs29012705
rs29009633
rs29014978
rs29013248 rs29013250
rs29011701
rs29010189
rs29010088 rs29010083
rs29027412
rs29012401
rs29024927 rs29012763
rs29012764 rs29012133
rs29012134 rs29016275
SCAFFOLD105315_6308 AAFC02115939
AAFC02115939
AAFC02101691
rs29014112 AAFC02023224
rs29012927 rs29012929
rs29014758 rs29014754
rs29015662
rs29018383 rs29011030
rs29010374 rs29010372
rs29016112 rs29016111
rs29024674
rs29018062
rs29012087 rs29012086
rs29012083 rs29012082
rs29013997 rs29012645
rs29012459 rs29012314
AAFC02045511 AAFC02045511
rs29011981
AAFC02148291
rs29025929
AAFC02147115 rs29023745
rs29014512 rs29014509
rs29018045
rs29010129 rs29010131
rs29010133 rs29010128
rs29010483 rs29010487
rs29010486
rs29014819 rs29014820
rs29014821 AAFC02114782
rs29009964 rs29010987
rs29020880 rs29020881
rs29010528
rs29012415 SCAFFOLD250148_11501
rs29027346
rs29013791 rs29013792
rs29013793
rs29026081 rs29026079
rs29023308 rs29009614
rs29009613 rs29025553
rs29016437
AF127030
AF127030
BV105337 rs29013234
rs29012472 rs29012474
UCP2 rs29023696
rs29024247 rs29021136
rs29021137
AAFC02037690
AAFC02037690
rs29027500
rs29018896 rs29022250
rs29011251
rs29010423 rs29012228
rs29013975
rs29012409 rs29012408
rs29020089 rs29020091
rs29020912
AAFC02038863 rs29013833
rs29017039 rs29011236
rs29011238
rs29014815
AAFC02138417 AAFC02138417
AAFC02092854 rs29009921
rs29017336
rs29009633 rs29009822
rs29012705
rs29014978 rs29010189
rs29013248 rs29013250
rs29011701
rs29026876
rs29026878
rs29026880
rs29010088 rs29010083
rs29009853 rs29027412
rs29012401
11
11
11
11
11
11
11
11
11
11

BTA16

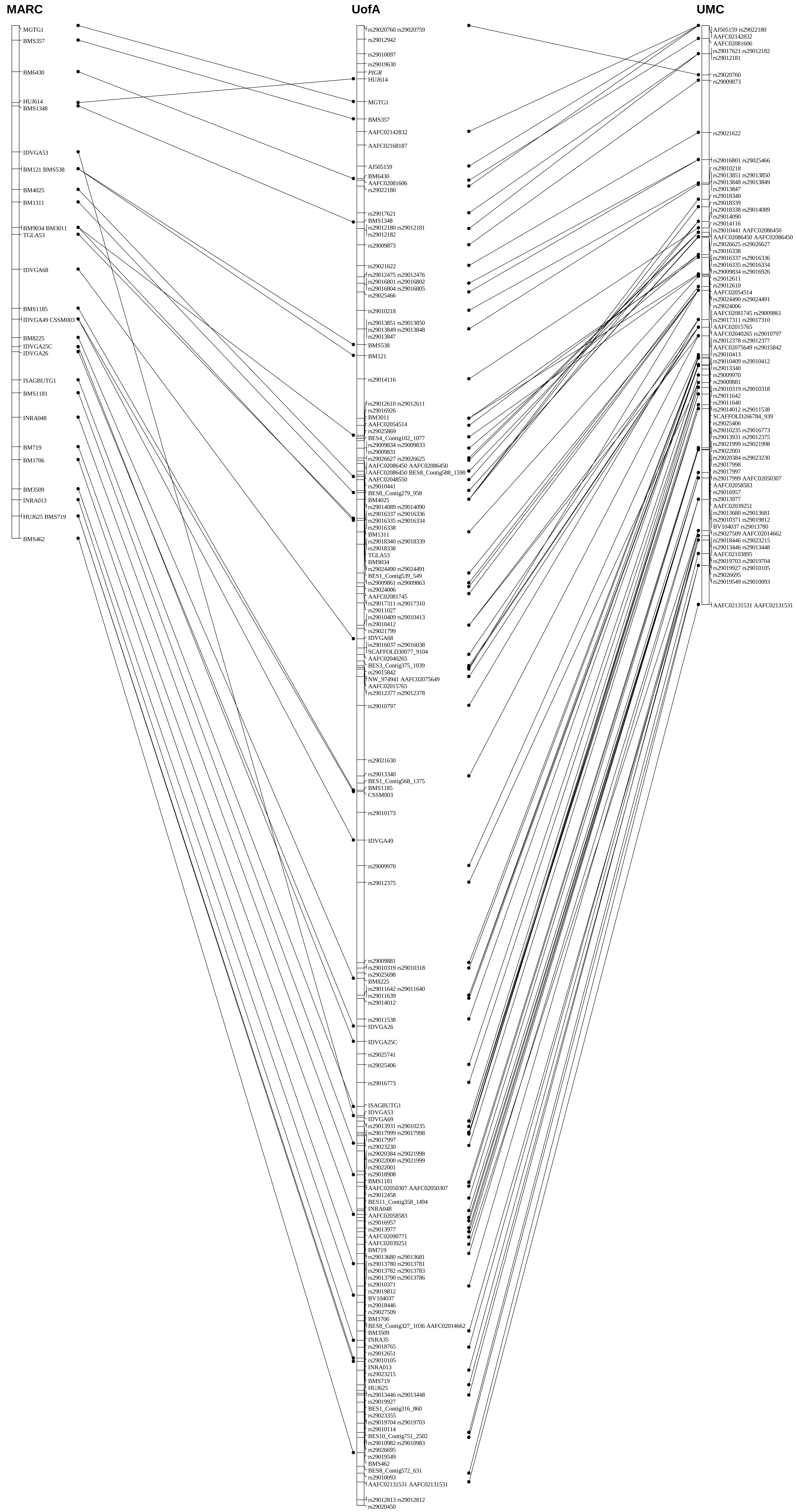
MARC
MGTG1
BMS357
BM6430
HUJ614
BMS1348
IDVGA53
BM121 BMS538
BM4025
BM1311
BM9034 BM3011
TGLA53
IDVGA68
BMS1185
IDVGA49 CSSM003
BM8225
IDVGA25C
IDVGA26
ISAGBUTG1
BMS1181
INRA048
BM719
BM1706
BM3509
INRA013
HUJ625 BMS719
BMS462
UofA
rs29020760 rs29020759
rs29012942
rs29010097
rs29019630
PIGR
HUJ614
MGTG1
BMS357
AAFC02142832
AAFC02168187
AJ505159
BM6430
AAFC02081606
rs29022180
rs29017621
BMS1348
rs29012180 rs29012181
rs29012182
rs29009873
rs29021622
rs29012475 rs29012476
rs29016801 rs29016802
rs29016804 rs29016805
rs29025466
rs29010218
rs29013851 rs29013850
rs29013849 rs29013848
rs29013847
BMS538
BM121
rs29014116
rs29012610 rs29012611
rs29016926
BM3011
AAFC02054514
rs29025869
BES4_Contig102_1077
rs29009834 rs29009833
rs29009831
rs29026627 rs29026625
AAFC02086450 AAFC02086450
AAFC02086450 BES8_Contig588_1590
AAFC02048550
rs29010441
BES8_Contig279_958
BM4025
rs29014089 rs29014090
rs29016337 rs29016336
rs29016335 rs29016334
rs29016338
BM1311
rs29018340 rs29018339
rs29018338
TGLA53
BM9034
rs29024490 rs29024491
BES1_Contig539_549
rs29009861 rs29009863
rs29024006
AAFC02081745
rs29017311 rs29017310
rs29011027
rs29010409 rs29010413
rs29010412
rs29021799
IDVGA68
rs29016037 rs29016038
SCAFFOLD30077_9104
AAFC02040265
BES3_Contig375_1039
rs29015842
NW_974941 AAFC02075649
AAFC02015765
rs29012377 rs29012378
rs29010797
rs29021630
rs29013340
BES1_Contig568_1375
BMS1185
CSSM003
rs29010173
IDVGA49
rs29009970
rs29012375
rs29009881
rs29010319 rs29010318
rs29025698
BM8225
rs29011642 rs29011640
rs29011639
rs29014012
rs29011538
IDVGA26
IDVGA25C
rs29025741
rs29025406
rs29016773
ISAGBUTG1
IDVGA53
IDVGA69
rs29013931 rs29010235
rs29017999 rs29017998
rs29017997
rs29023230
rs29020384 rs29021998
rs29022000 rs29021999
rs29022001
rs29018908
BMS1181
AAFC02050307 AAFC02050307
rs29012458
BES11_Contig358_1494
INRA048
AAFC02058583
rs29016957
rs29013977
AAFC02090771
AAFC02039251
BM719
rs29013680 rs29013681
rs29013780 rs29013781
rs29013782 rs29013783
rs29013790 rs29013786
rs29010371
rs29019812
BV104037
rs29018446
rs29027509
BM1706
BES8_Contig327_1036 AAFC02014662
BM3509
INRA35
rs29018765
rs29012651
rs29010105
INRA013
rs29023215
BMS719
HUJ625
rs29013446 rs29013448
rs29019927
BES1_Contig316_860
rs29023355
rs29019704 rs29019703
rs29010114
BES10_Contig751_2502
rs29010982 rs29010983
rs29026695
rs29019549
BMS462
BES8_Contig572_631
rs29010093
AAFC02131531 AAFC02131531
rs29012813 rs29012812
rs29020450
UMC
AJ505159 rs29022180
AAFC02142832
AAFC02081606
rs29017621 rs29012182
rs29012181
rs29020760
rs29009873
rs29021622
rs29016801 rs29025466
rs29010218
rs29013851 rs29013850
rs29013848 rs29013849
rs29013847
rs29018340
rs29018339
rs29018338 rs29014089
rs29014090
rs29014116
rs29010441 AAFC02086450
AAFC02086450 AAFC02086450
rs29026625 rs29026627
rs29016338
rs29016337 rs29016336
rs29016335 rs29016334
rs29009834 rs29016926
rs29012611
rs29012610
AAFC02054514
rs29024490 rs29024491
rs29024006
AAFC02081745 rs29009861
rs29017311 rs29017310
AAFC02015765
AAFC02040265 rs29010797
rs29012378 rs29012377
AAFC02075649 rs29015842
rs29010413
rs29010409 rs29010412
rs29013340
rs29009970
rs29009881
rs29010319 rs29010318
rs29011642
rs29011640
rs29014012 rs29011538
SCAFFOLD266784_939
rs29025406
rs29010235 rs29016773
rs29013931 rs29012375
rs29021999 rs29021998
rs29022001
rs29020384 rs29023230
rs29017998
rs29017997
rs29017999 AAFC02050307
AAFC02058583
rs29016957
rs29013977
AAFC02039251
rs29013680 rs29013681
rs29010371 rs29019812
BV104037 rs29013780
rs29027509 AAFC02014662
rs29018446 rs29023215
rs29013446 rs29013448
AAFC02103895
rs29019703 rs29019704
rs29019927 rs29010105
rs29026695
rs29019549 rs29010093
AAFC02131531 AAFC02131531

BTA16

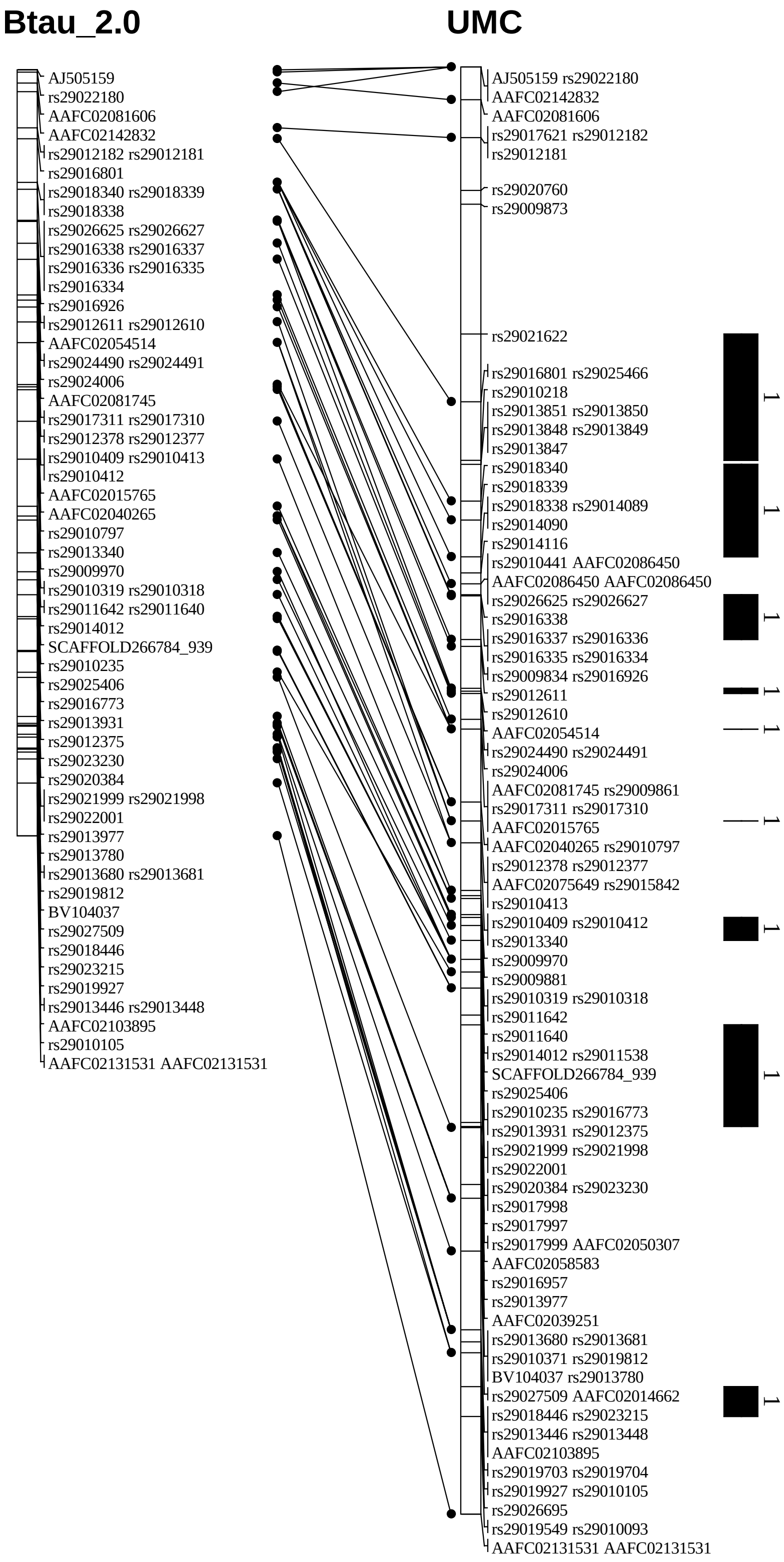
Btau_2.0
UMC
AJ505159
rs29022180
AAFC02081606
AAFC02142832
rs29012182 rs29012181
rs29016801
rs29018340 rs29018339
rs29018338
rs29026625 rs29026627
rs29016338 rs29016337
rs29016336 rs29016335
rs29016334
rs29016926
rs29012611 rs29012610
AAFC02054514
rs29024490 rs29024491
rs29024006
AAFC02081745
rs29017311 rs29017310
rs29012378 rs29012377
rs29010409 rs29010413
rs29010412
AAFC02015765
AAFC02040265
rs29010797
rs29013340
rs29009970
rs29010319 rs29010318
rs29011642 rs29011640
rs29014012
SCAFFOLD266784_939
rs29010235
rs29025406
rs29016773
rs29013931
rs29012375
rs29023230
rs29020384
rs29021999 rs29021998
rs29022001
rs29013977
rs29013780
rs29013680 rs29013681
rs29019812
BV104037
rs29027509
rs29018446
rs29023215
rs29019927
rs29013446 rs29013448
AAFC02103895
rs29010105
AAFC02131531 AAFC02131531
AJ505159 rs29022180
AAFC02142832
AAFC02081606
rs29017621 rs29012182
rs29012181
rs29020760
rs29009873
rs29021622
rs29016801 rs29025466
rs29010218
rs29013851 rs29013850
rs29013848 rs29013849
rs29013847
rs29018340
rs29018339
rs29018338 rs29014089
rs29014090
rs29014116
rs29010441 AAFC02086450
AAFC02086450 AAFC02086450
rs29026625 rs29026627
rs29016338
rs29016337 rs29016336
rs29016335 rs29016334
rs29009834 rs29016926
rs29012611
rs29012610
AAFC02054514
rs29024490 rs29024491
rs29024006
AAFC02081745 rs29009861
rs29017311 rs29017310
AAFC02015765
AAFC02040265 rs29010797
rs29012378 rs29012377
AAFC02075649 rs29015842
rs29010413
rs29010409 rs29010412
rs29013340
rs29009970
rs29009881
rs29010319 rs29010318
rs29011642
rs29011640
rs29014012 rs29011538
SCAFFOLD266784_939
rs29025406
rs29010235 rs29016773
rs29013931 rs29012375
rs29021999 rs29021998
rs29022001
rs29020384 rs29023230
rs29017998
rs29017997
rs29017999 AAFC02050307
AAFC02058583
rs29016957
rs29013977
AAFC02039251
rs29013680 rs29013681
rs29010371 rs29019812
BV104037 rs29013780
rs29027509 AAFC02014662
rs29018446 rs29023215
rs29013446 rs29013448
AAFC02103895
rs29019703 rs29019704
rs29019927 rs29010105
rs29026695
rs29019549 rs29010093
AAFC02131531 AAFC02131531

BTA17

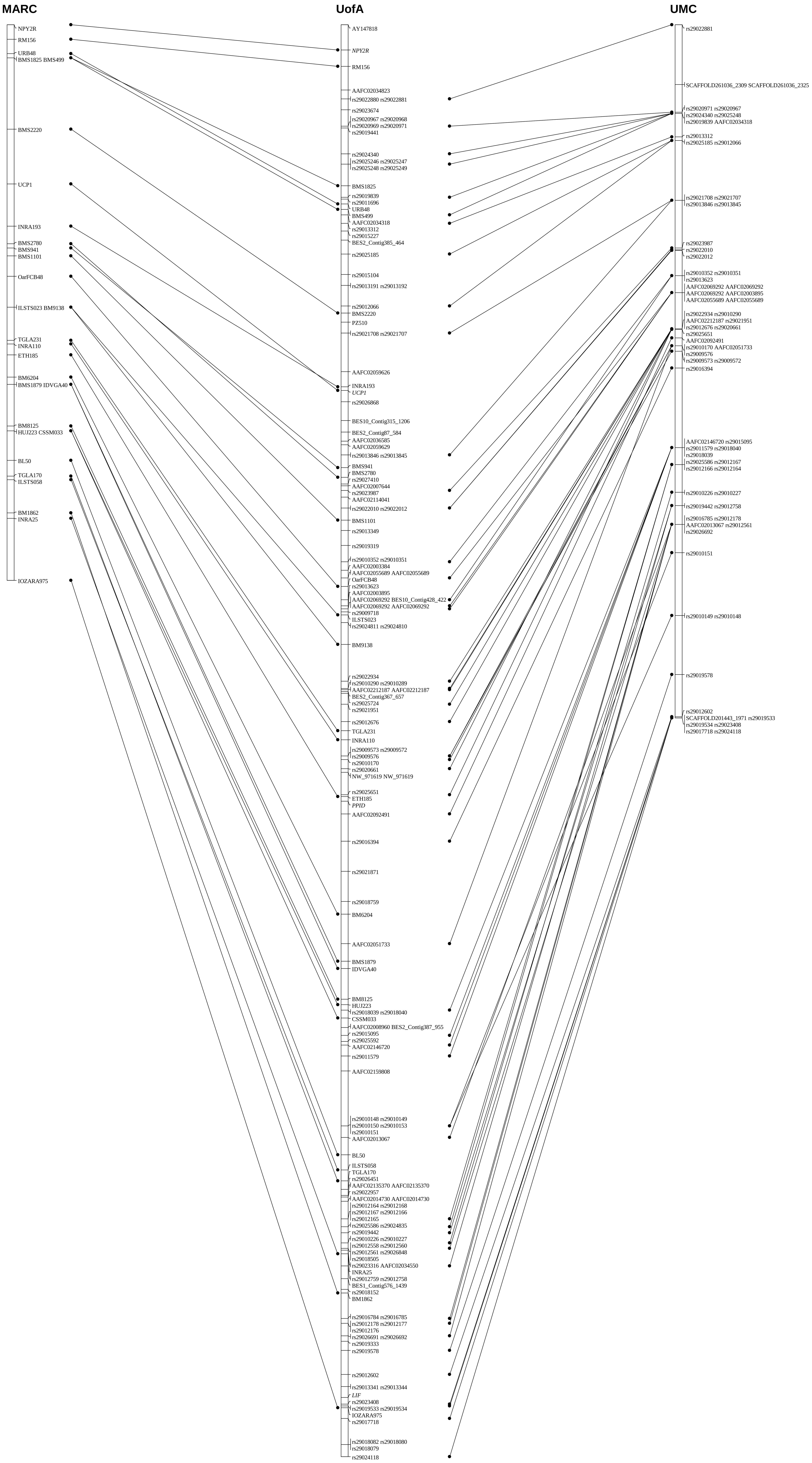
MARC
NPY2R
RM156
URB48
BMS1825 BMS499
BMS2220
UCP1
INRA193
BMS2780
BMS941
BMS1101
OarFCB48
ILSTS023 BM9138
TGLA231
INRA110
ETH185
BM6204
BMS1879 IDVGA40
BM8125
HUJ223 CSSM033
BL50
TGLA170
ILSTS058
BM1862
INRA25
IOZARA975
UofA
AY147818
NPY2R
RM156
AAFC02034823
rs29022880 rs29022881
rs29023674
rs29020967 rs29020968
rs29020969 rs29020971
rs29019441
rs29024340
rs29025246 rs29025247
rs29025248 rs29025249
BMS1825
rs29019839
rs29011696
URB48
BMS499
AAFC02034318
rs29013312
rs29015227
BES2_Contig385_464
rs29025185
rs29015104
rs29013191 rs29013192
rs29012066
BMS2220
PZ510
rs29021708 rs29021707
AAFC02059626
INRA193
UCP1
rs29026868
BES10_Contig315_1206
BES2_Contig87_584
AAFC02036585
AAFC02059629
rs29013846 rs29013845
BMS941
BMS2780
rs29027410
AAFC02007644
rs29023987
AAFC02114041
rs29022010 rs29022012
BMS1101
rs29013349
rs29019319
rs29010352 rs29010351
AAFC02003384
AAFC02055689 AAFC02055689
OarFCB48
rs29013623
AAFC02003895
AAFC02069292 BES10_Contig428_422
AAFC02069292 AAFC02069292
rs29009718
ILSTS023
rs29024811 rs29024810
BM9138
rs29022934
rs29010290 rs29010289
AAFC02212187 AAFC02212187
BES2_Contig367_657
rs29025724
rs29021951
rs29012676
TGLA231
INRA110
rs29009573 rs29009572
rs29009576
rs29010170
rs29020661
NW_971619 NW_971619
rs29025651
ETH185
PPID
AAFC02092491
rs29016394
rs29021871
rs29018759
BM6204
AAFC02051733
BMS1879
IDVGA40
BM8125
HUJ223
rs29018039 rs29018040
CSSM033
AAFC02008960 BES2_Contig387_955
rs29015095
rs29025592
AAFC02146720
rs29011579
AAFC02159808
rs29010148 rs29010149
rs29010150 rs29010153
rs29010151
AAFC02013067
BL50
ILSTS058
TGLA170
rs29026451
AAFC02135370 AAFC02135370
rs29022957
AAFC02014730 AAFC02014730
rs29012164 rs29012168
rs29012167 rs29012166
rs29012165
rs29025586 rs29024835
rs29019442
rs29010226 rs29010227
rs29012558 rs29012560
rs29012561 rs29026848
rs29018505
rs29023316 AAFC02034550
INRA25
rs29012759 rs29012758
BES1_Contig576_1439
rs29018152
BM1862
rs29016784 rs29016785
rs29012178 rs29012177
rs29012176
rs29026691 rs29026692
rs29019333
rs29019578
rs29012602
rs29013341 rs29013344
LIF
rs29023408
rs29019533 rs29019534
IOZARA975
rs29017718
rs29018082 rs29018080
rs29018079
rs29024118
UMC
rs29022881
SCAFFOLD261036_2309 SCAFFOLD261036_2325
rs29020971 rs29020967
rs29024340 rs29025248
rs29019839 AAFC02034318
rs29013312
rs29025185 rs29012066
rs29021708 rs29021707
rs29013846 rs29013845
rs29023987
rs29022010
rs29022012
rs29010352 rs29010351
rs29013623
AAFC02069292 AAFC02069292
AAFC02069292 AAFC02003895
AAFC02055689 AAFC02055689
rs29022934 rs29010290
AAFC02212187 rs29021951
rs29012676 rs29020661
rs29025651
AAFC02092491
rs29010170 AAFC02051733
rs29009576
rs29009573 rs29009572
rs29016394
AAFC02146720 rs29015095
rs29011579 rs29018040
rs29018039
rs29025586 rs29012167
rs29012166 rs29012164
rs29010226 rs29010227
rs29019442 rs29012758
rs29016785 rs29012178
AAFC02013067 rs29012561
rs29026692
rs29010151
rs29010149 rs29010148
rs29019578
rs29012602
SCAFFOLD201443_1971 rs29019533
rs29019534 rs29023408
rs29017718 rs29024118

BTA17

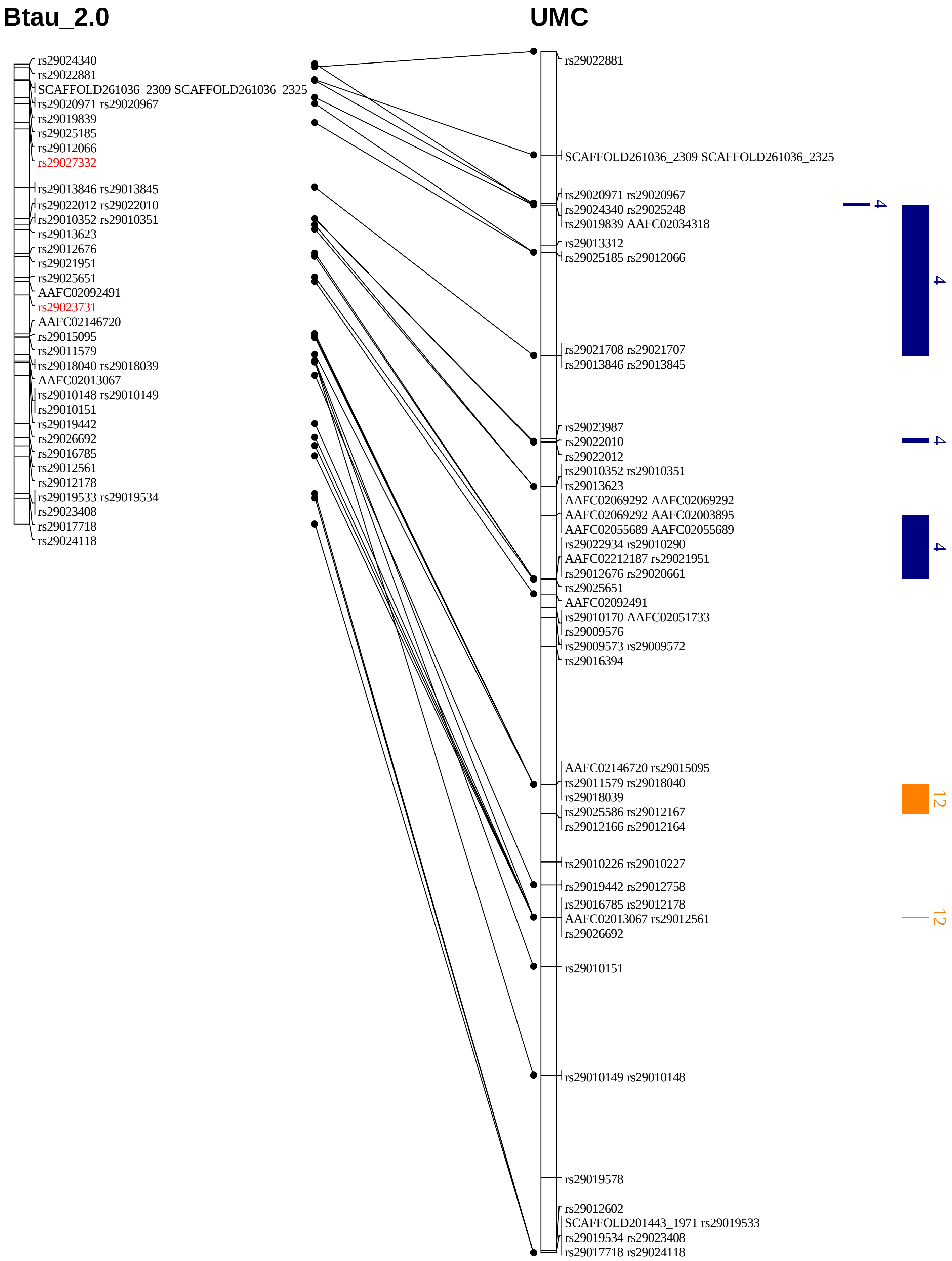

Btau_2.0
UMC
rs29024340
rs29022881
SCAFFOLD261036_2309 SCAFFOLD261036_2325
rs29020971 rs29020967
rs29019839
rs29025185
rs29012066
rs29027332
rs29013846 rs29013845
rs29022012 rs29022010
rs29010352 rs29010351
rs29013623
rs29012676
rs29021951
rs29025651
AAFC02092491
rs29023731
AAFC02146720
rs29015095
rs29011579
rs29018040 rs29018039
AAFC02013067
rs29010148 rs29010149
rs29010151
rs29019442
rs29026692
rs29016785
rs29012561
rs29012178
rs29019533 rs29019534
rs29023408
rs29017718
rs29024118
rs29022881
SCAFFOLD261036_2309 SCAFFOLD261036_2325
rs29020971 rs29020967
rs29024340 rs29025248
rs29019839 AAFC02034318
rs29013312
rs29025185 rs29012066
rs29021708 rs29021707
rs29013846 rs29013845
rs29023987
rs29022010
rs29022012
rs29010352 rs29010351
rs29013623
AAFC02069292 AAFC02069292
AAFC02069292 AAFC02003895
AAFC02055689 AAFC02055689
rs29022934 rs29010290
AAFC02212187 rs29021951
rs29012676 rs29020661
rs29025651
AAFC02092491
rs29010170 AAFC02051733
rs29009576
rs29009573 rs29009572
rs29016394
AAFC02146720 rs29015095
rs29011579 rs29018040
rs29018039
rs29025586 rs29012167
rs29012166 rs29012164
rs29010226 rs29010227
rs29019442 rs29012758
rs29016785 rs29012178
AAFC02013067 rs29012561
rs29026692
rs29010151
rs29010149 rs29010148
rs29019578
rs29012602
SCAFFOLD201443_1971 rs29019533
rs29019534 rs29023408
rs29017718 rs29024118
4
4
4
4
12
12

BTA18

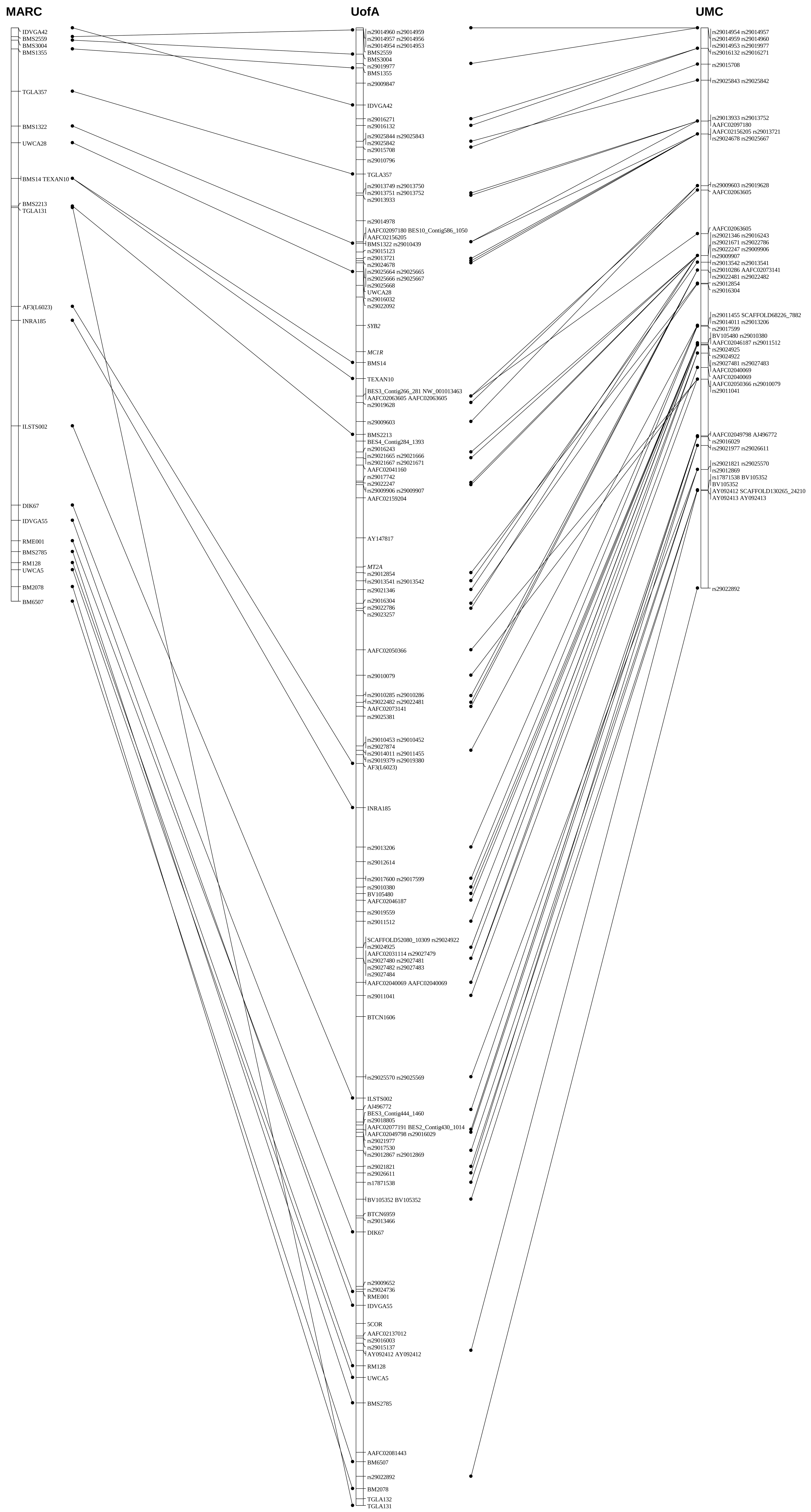
MARC
UofA
UMC
IDVGA42
BMS2559
BMS3004
BMS1355
TGLA357
BMS1322
UWCA28
BMS14 TEXAN10
BMS2213
TGLA131
AF3(L6023)
INRA185
ILSTS002
DIK67
IDVGA55
RME001
BMS2785
RM128
UWCA5
BM2078
BM6507
rs29014960 rs29014959
rs29014957 rs29014956
rs29014954 rs29014953
BMS2559
BMS3004
rs29019977
BMS1355
rs29009847
IDVGA42
rs29016271
rs29016132
rs29025844 rs29025843
rs29025842
rs29015708
rs29010796
TGLA357
rs29013749 rs29013750
rs29013751 rs29013752
rs29013933
rs29014978
AAFC02097180 BES10_Contig586_1050
AAFC02156205
BMS1322 rs29010439
rs29015123
rs29013721
rs29024678
rs29025664 rs29025665
rs29025666 rs29025667
rs29025668
UWCA28
rs29016032
rs29022092
SYB2
MC1R
BMS14
TEXAN10
BES3_Contig266_281 NW_001013463
AAFC02063605 AAFC02063605
rs29019628
rs29009603
BMS2213
BES4_Contig284_1393
rs29016243
rs29021665 rs29021666
rs29021667 rs29021671
AAFC02041160
rs29017742
rs29022247
rs29009906 rs29009907
AAFC02159204
AY147817
MT2A
rs29012854
rs29013541 rs29013542
rs29021346
rs29016304
rs29022786
rs29023257
AAFC02050366
rs29010079
rs29010285 rs29010286
rs29022482 rs29022481
AAFC02073141
rs29025381
rs29010453 rs29010452
rs29027874
rs29014011 rs29011455
rs29019379 rs29019380
AF3(L6023)
INRA185
rs29013206
rs29012614
rs29017600 rs29017599
rs29010380
BV105480
AAFC02046187
rs29019559
rs29011512
SCAFFOLD52080_10309 rs29024922
rs29024925
AAFC02031114 rs29027479
rs29027480 rs29027481
rs29027482 rs29027483
rs29027484
AAFC02040069 AAFC02040069
rs29011041
BTCN1606
rs29025570 rs29025569
ILSTS002
AJ496772
BES3_Contig444_1460
rs29018805
AAFC02077191 BES2_Contig430_1014
AAFC02049798 rs29016029
rs29021977
rs29017530
rs29012867 rs29012869
rs29021821
rs29026611
rs17871538
BV105352 BV105352
BTCN6959
rs29013466
DIK67
rs29009652
rs29024736
RME001
IDVGA55
5COR
AAFC02137012
rs29016003
rs29015137
AY092412 AY092412
RM128
UWCA5
BMS2785
AAFC02081443
BM6507
rs29022892
BM2078
TGLA132
TGLA131
rs29014954 rs29014957
rs29014959 rs29014960
rs29014953 rs29019977
rs29016132 rs29016271
rs29015708
rs29025843 rs29025842
rs29013933 rs29013752
AAFC02097180
AAFC02156205 rs29013721
rs29024678 rs29025667
rs29009603 rs29019628
AAFC02063605
AAFC02063605
rs29021346 rs29016243
rs29021671 rs29022786
rs29022247 rs29009906
rs29009907
rs29013542 rs29013541
rs29010286 AAFC02073141
rs29022481 rs29022482
rs29012854
rs29016304
rs29011455 SCAFFOLD68226_7882
rs29014011 rs29013206
rs29017599
BV105480 rs29010380
AAFC02046187 rs29011512
rs29024925
rs29024922
rs29027481 rs29027483
AAFC02040069
AAFC02040069
AAFC02050366 rs29010079
rs29011041
AAFC02049798 AJ496772
rs29016029
rs29021977 rs29026611
rs29021821 rs29025570
rs29012869
rs17871538 BV105352
BV105352
AY092412 SCAFFOLD130265_24210
AY092413 AY092413
rs29022892

BTA18

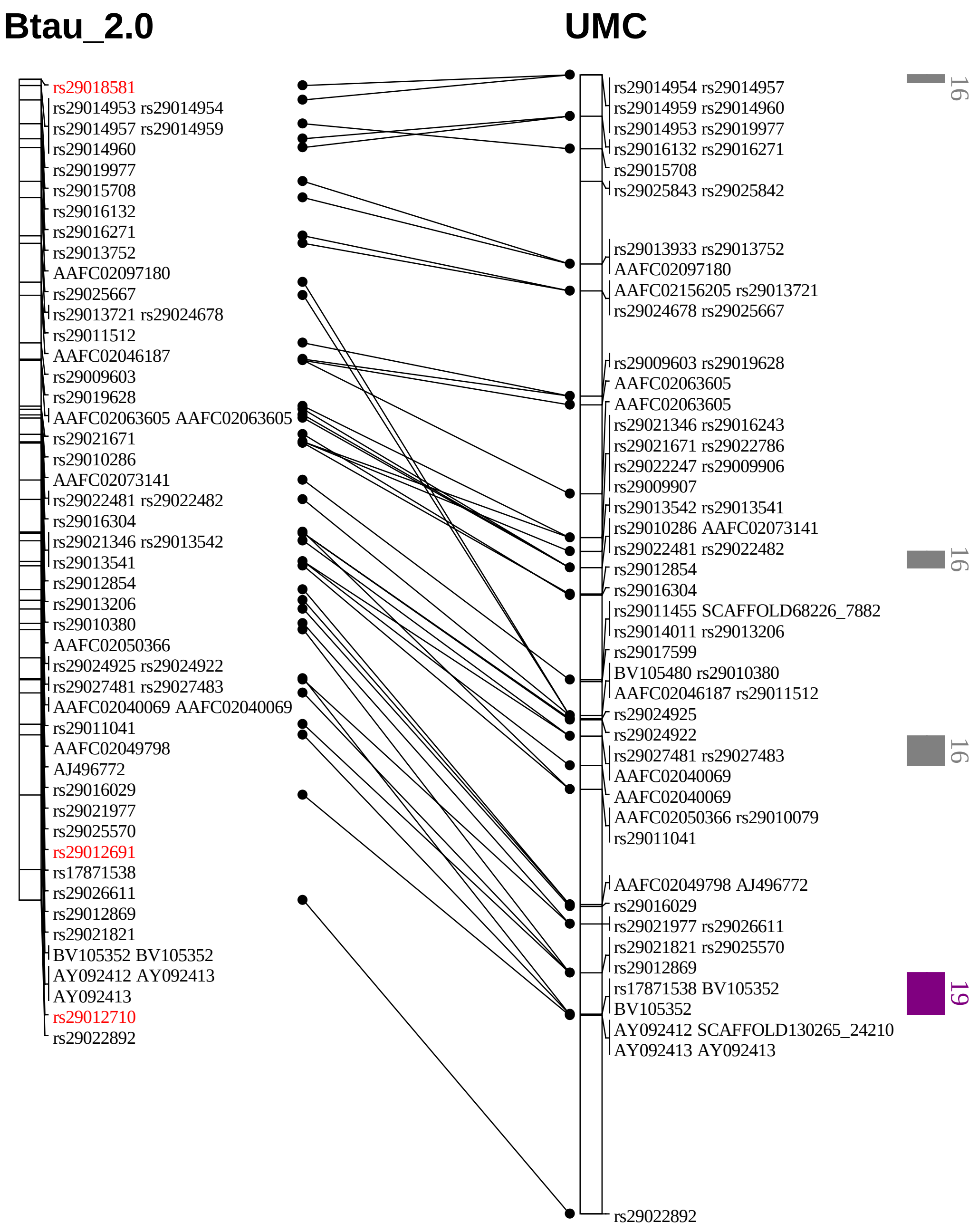
Btau_2.0
UMC
rs29018581
rs29014953 rs29014954
rs29014957 rs29014959
rs29014960
rs29019977
rs29015708
rs29016132
rs29016271
rs29013752
AAFC02097180
rs29025667
rs29013721 rs29024678
rs29011512
AAFC02046187
rs29009603
rs29019628
AAFC02063605 AAFC02063605
rs29021671
rs29010286
AAFC02073141
rs29022481 rs29022482
rs29016304
rs29021346 rs29013542
rs29013541
rs29012854
rs29013206
rs29010380
AAFC02050366
rs29024925 rs29024922
rs29027481 rs29027483
AAFC02040069 AAFC02040069
rs29011041
AAFC02049798
AJ496772
rs29016029
rs29021977
rs29025570
rs29012691
rs17871538
rs29026611
rs29012869
rs29021821
BV105352 BV105352
AY092412 AY092413
AY092413
rs29012710
rs29022892
rs29014954 rs29014957
rs29014959 rs29014960
rs29014953 rs29019977
rs29016132 rs29016271
rs29015708
rs29025843 rs29025842
rs29013933 rs29013752
AAFC02097180
AAFC02156205 rs29013721
rs29024678 rs29025667
rs29009603 rs29019628
AAFC02063605
AAFC02063605
rs29021346 rs29016243
rs29021671 rs29022786
rs29022247 rs29009906
rs29009907
rs29013542 rs29013541
rs29010286 AAFC02073141
rs29022481 rs29022482
rs29012854
rs29016304
rs29011455 SCAFFOLD68226_7882
rs29014011 rs29013206
rs29017599
BV105480 rs29010380
AAFC02046187 rs29011512
rs29024925
rs29024922
rs29027481 rs29027483
AAFC02040069
AAFC02040069
AAFC02050366 rs29010079
rs29011041
AAFC02049798 AJ496772
rs29016029
rs29021977 rs29026611
rs29021821 rs29025570
rs29012869
rs17871538 BV105352
BV105352
AY092412 SCAFFOLD130265_24210
AY092413 AY092413
rs29022892
16
16
16
19

BTA19

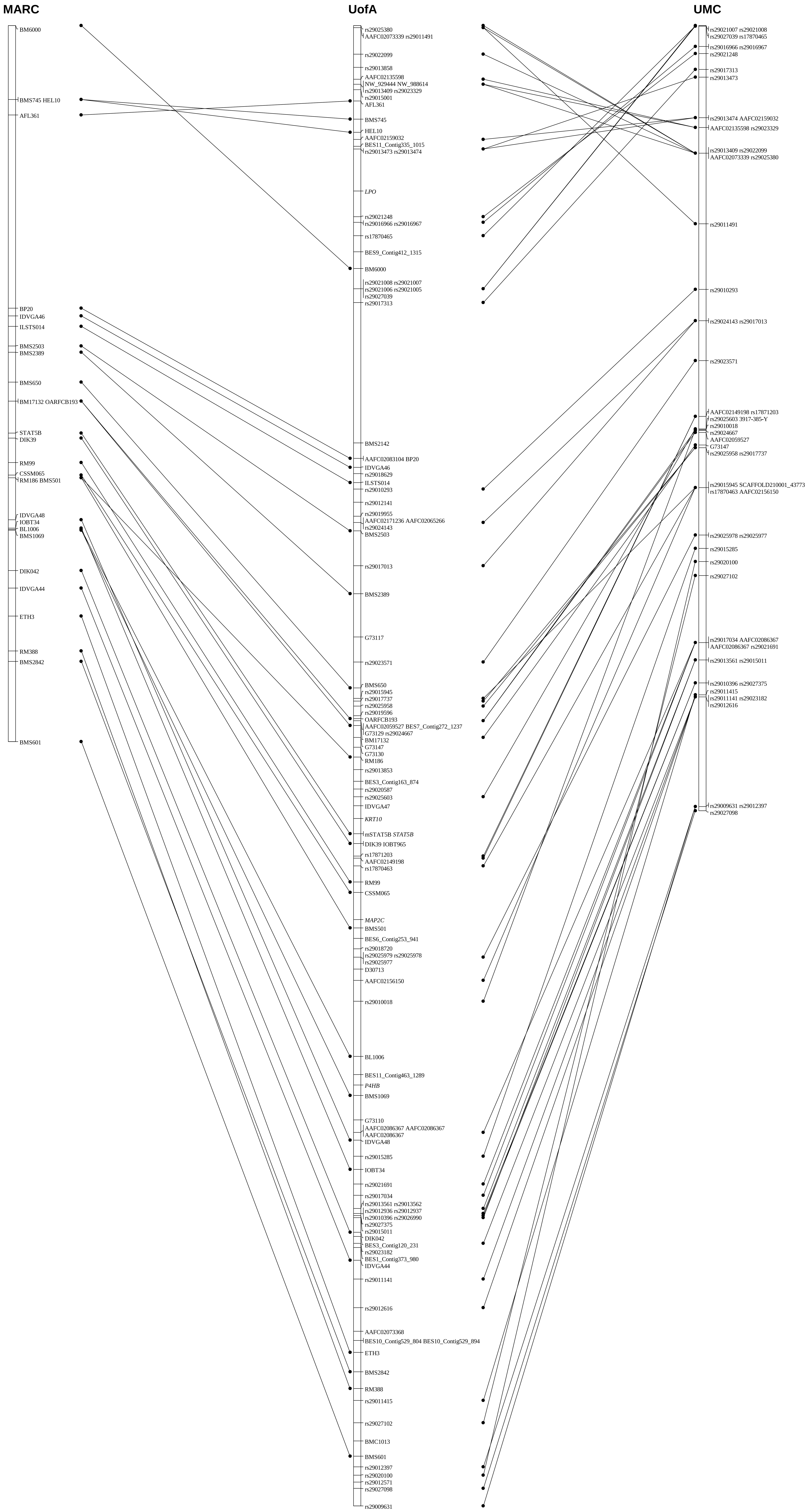
MARC
BM6000
BMS745 HEL10
AFL361
BP20
IDVGA46
ILSTS014
BMS2503
BMS2389
BMS650
BM17132 OARFCB193
STAT5B
DIK39
RM99
CSSM065
RM186 BMS501
IDVGA48
IOBT34
BL1006
BMS1069
DIK042
IDVGA44
ETH3
RM388
BMS2842
BMS601
UofA
rs29025380
AAFC02073339 rs29011491
rs29022099
rs29013858
AAFC02135598
NW_929444 NW_988614
rs29013409 rs29023329
rs29015001
AFL361
BMS745
HEL10
AAFC02159032
BES11_Contig335_1015
rs29013473 rs29013474
LPO
rs29021248
rs29016966 rs29016967
rs17870465
BES9_Contig412_1315
BM6000
rs29021008 rs29021007
rs29021006 rs29021005
rs29027039
rs29017313
BMS2142
AAFC02083104 BP20
IDVGA46
rs29018629
ILSTS014
rs29010293
rs29012141
rs29019955
AAFC02171236 AAFC02065266
rs29024143
BMS2503
rs29017013
BMS2389
G73117
rs29023571
BMS650
rs29015945
rs29017737
rs29025958
rs29019596
OARFCB193
AAFC02059527 BES7_Contig272_1237
G73129 rs29024667
BM17132
G73147
G73130
RM186
rs29013853
BES3_Contig163_874
rs29020587
rs29025603
IDVGA47
KRT10
mSTAT5B STAT5B
DIK39 IOBT965
rs17871203
AAFC02149198
rs17870463
RM99
CSSM065
MAP2C
BMS501
BES6_Contig253_941
rs29018720
rs29025979 rs29025978
rs29025977
D30713
AAFC02156150
rs29010018
BL1006
BES11_Contig463_1289
P4HB
BMS1069
G73110
AAFC02086367 AAFC02086367
AAFC02086367
IDVGA48
rs29015285
IOBT34
rs29021691
rs29017034
rs29013561 rs29013562
rs29012936 rs29012937
rs29010396 rs29026990
rs29027375
rs29015011
DIK042
BES3_Contig120_231
rs29023182
BES1_Contig373_980
IDVGA44
rs29011141
rs29012616
AAFC02073368
BES10_Contig529_804 BES10_Contig529_894
ETH3
BMS2842
RM388
rs29011415
rs29027102
BMC1013
BMS601
rs29012397
rs29020100
rs29012571
rs29027098
rs29009631
UMC
rs29021007 rs29021008
rs29027039 rs17870465
rs29016966 rs29016967
rs29021248
rs29017313
rs29013473
rs29013474 AAFC02159032
AAFC02135598 rs29023329
rs29013409 rs29022099
AAFC02073339 rs29025380
rs29011491
rs29010293
rs29024143 rs29017013
rs29023571
AAFC02149198 rs17871203
rs29025603 3917-385-Y
rs29010018
rs29024667
AAFC02059527
G73147
rs29025958 rs29017737
rs29015945 SCAFFOLD210001_43773
rs17870463 AAFC02156150
rs29025978 rs29025977
rs29015285
rs29020100
rs29027102
rs29017034 AAFC02086367
AAFC02086367 rs29021691
rs29013561 rs29015011
rs29010396 rs29027375
rs29011415
rs29011141 rs29023182
rs29012616
rs29009631 rs29012397
rs29027098

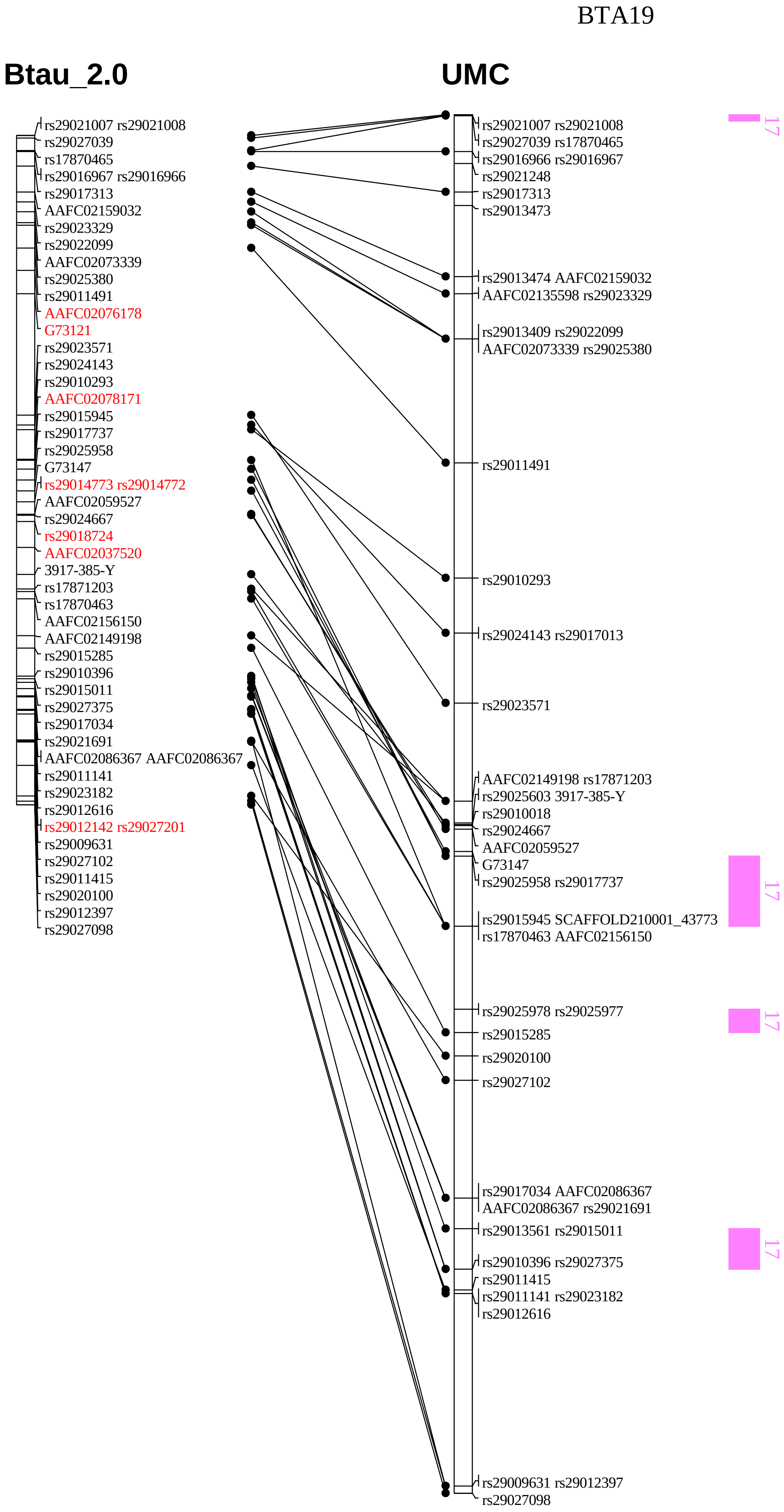

BTA19
Btau_2.0
UMC
rs29021007 rs29021008
rs29027039
rs17870465
rs29016967 rs29016966
rs29017313
AAFC02159032
rs29023329
rs29022099
AAFC02073339
rs29025380
rs29011491
AAFC02076178
G73121
rs29023571
rs29024143
rs29010293
AAFC02078171
rs29015945
rs29017737
rs29025958
G73147
rs29014773 rs29014772
AAFC02059527
rs29024667
rs29018724
AAFC02037520
3917-385-Y
rs17871203
rs17870463
AAFC02156150
AAFC02149198
rs29015285
rs29010396
rs29015011
rs29027375
rs29017034
rs29021691
AAFC02086367 AAFC02086367
rs29011141
rs29023182
rs29012616
rs29012142 rs29027201
rs29009631
rs29027102
rs29011415
rs29020100
rs29012397
rs29027098
rs29021007 rs29021008
rs29027039 rs17870465
rs29016966 rs29016967
rs29021248
rs29017313
rs29013473
rs29013474 AAFC02159032
AAFC02135598 rs29023329
rs29013409 rs29022099
AAFC02073339 rs29025380
rs29011491
rs29010293
rs29024143 rs29017013
rs29023571
AAFC02149198 rs17871203
rs29025603 3917-385-Y
rs29010018
rs29024667
AAFC02059527
G73147
rs29025958 rs29017737
rs29015945 SCAFFOLD210001_43773
rs17870463 AAFC02156150
rs29025978 rs29025977
rs29015285
rs29020100
rs29027102
rs29017034 AAFC02086367
AAFC02086367 rs29021691
rs29013561 rs29015011
rs29010396 rs29027375
rs29011415
rs29011141 rs29023182
rs29012616
rs29009631 rs29012397
rs29027098
17
17
17
17

BTA20

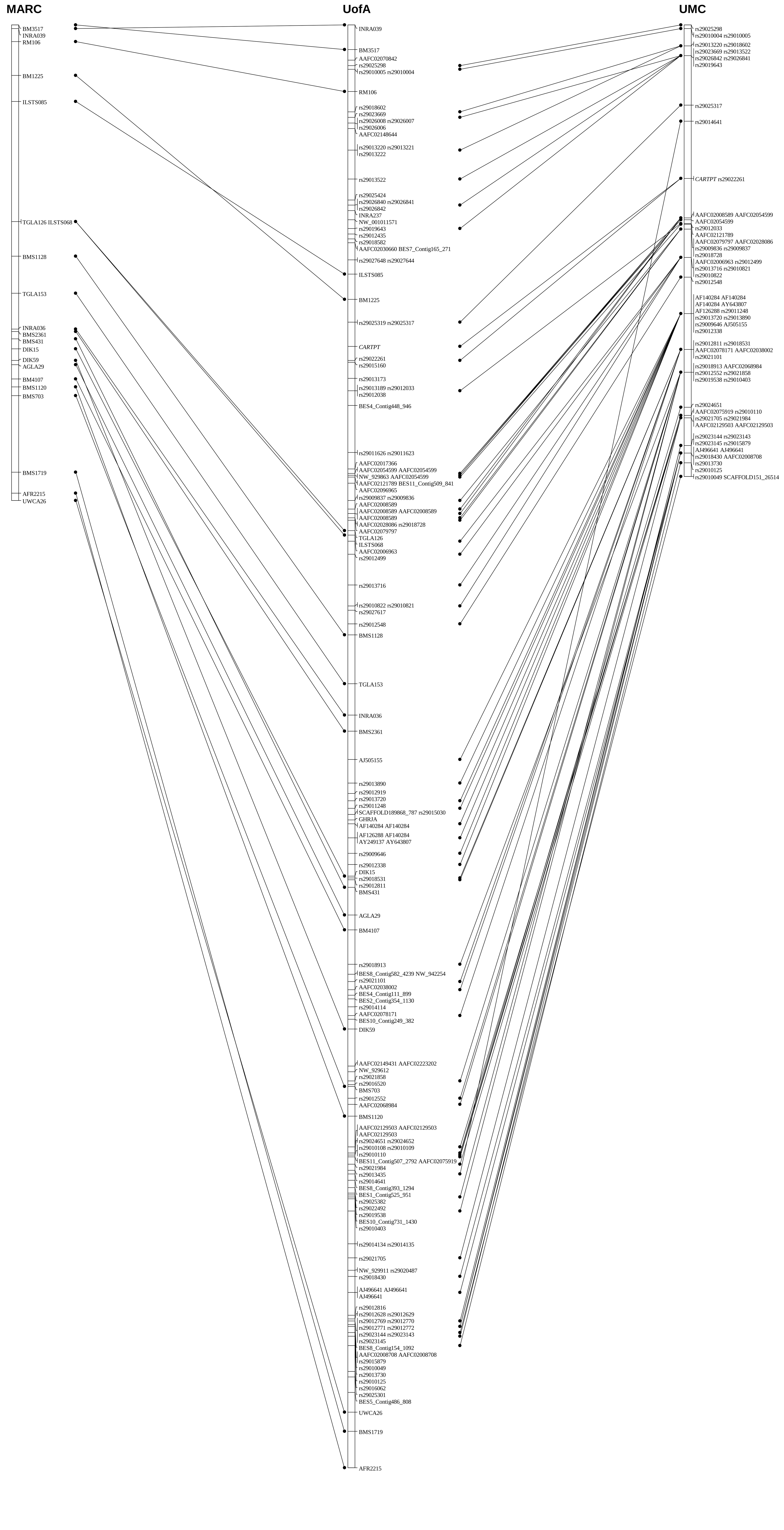

MARC
UofA
UMC
BM3517
INRA039
RM106
BM1225
ILSTS085
TGLA126 ILSTS068
BMS1128
TGLA153
INRA036
BMS2361
BMS431
DIK15
DIK59
AGLA29
BM4107
BMS1120
BMS703
BMS1719
AFR2215
UWCA26
INRA039
BM3517
AAFC02070842
rs29025298
rs29010005 rs29010004
RM106
rs29018602
rs29023669
rs29026008 rs29026007
rs29026006
AAFC02148644
rs29013220 rs29013221
rs29013222
rs29013522
rs29025424
rs29026840 rs29026841
rs29026842
INRA237
NW_001011571
rs29019643
rs29012435
rs29018582
AAFC02030660 BES7_Contig165_271
rs29027648 rs29027644
ILSTS085
BM1225
rs29025319 rs29025317
CARTPT
rs29022261
rs29015160
rs29013173
rs29013189 rs29012033
rs29012038
BES4_Contig448_946
rs29011626 rs29011623
AAFC02017366
AAFC02054599 AAFC02054599
NW_929863 AAFC02054599
AAFC02121789 BES11_Contig509_841
AAFC02096965
rs29009837 rs29009836
AAFC02008589
AAFC02008589 AAFC02008589
AAFC02008589
AAFC02028086 rs29018728
AAFC02079797
TGLA126
ILSTS068
AAFC02006963
rs29012499
rs29013716
rs29010822 rs29010821
rs29027617
rs29012548
BMS1128
TGLA153
INRA036
BMS2361
AJ505155
rs29013890
rs29012919
rs29013720
rs29011248
SCAFFOLD189868_787 rs29015030
GHRJA
AF140284 AF140284
AF126288 AF140284
AY249137 AY643807
rs29009646
rs29012338
DIK15
rs29018531
rs29012811
BMS431
AGLA29
BM4107
rs29018913
BES8_Contig582_4239 NW_942254
rs29021101
AAFC02038002
BES4_Contig111_899
BES2_Contig354_1130
rs29014114
AAFC02078171
BES10_Contig249_382
DIK59
AAFC02149431 AAFC02223202
NW_929612
rs29021858
rs29016520
BMS703
rs29012552
AAFC02068984
BMS1120
AAFC02129503 AAFC02129503
AAFC02129503
rs29024651 rs29024652
rs29010108 rs29010109
rs29010110
BES11_Contig507_2792 AAFC02075919
rs29021984
rs29013435
rs29014641
BES8_Contig393_1294
BES1_Contig525_951
rs29025382
rs29022492
rs29019538
BES10_Contig731_1430
rs29010403
rs29014134 rs29014135
rs29021705
NW_929911 rs29020487
rs29018430
AJ496641 AJ496641
AJ496641
rs29012816
rs29012628 rs29012629
rs29012769 rs29012770
rs29012771 rs29012772
rs29023144 rs29023143
rs29023145
BES8_Contig154_1092
AAFC02008708 AAFC02008708
rs29015879
rs29010049
rs29013730
rs29010125
rs29016062
rs29025301
BES5_Contig486_808
UWCA26
BMS1719
AFR2215
rs29025298
rs29010004 rs29010005
rs29013220 rs29018602
rs29023669 rs29013522
rs29026842 rs29026841
rs29019643
rs29025317
rs29014641
CARTPT rs29022261
AAFC02008589 AAFC02054599
AAFC02054599
rs29012033
AAFC02121789
AAFC02079797 AAFC02028086
rs29009836 rs29009837
rs29018728
AAFC02006963 rs29012499
rs29013716 rs29010821
rs29010822
rs29012548
AF140284 AF140284
AF140284 AY643807
AF126288 rs29011248
rs29013720 rs29013890
rs29009646 AJ505155
rs29012338
rs29012811 rs29018531
AAFC02078171 AAFC02038002
rs29021101
rs29018913 AAFC02068984
rs29012552 rs29021858
rs29019538 rs29010403
rs29024651
AAFC02075919 rs29010110
rs29021705 rs29021984
AAFC02129503 AAFC02129503
rs29023144 rs29023143
rs29023145 rs29015879
AJ496641 AJ496641
rs29018430 AAFC02008708
rs29013730
rs29010125
rs29010049 SCAFFOLD151_26514

BTA20

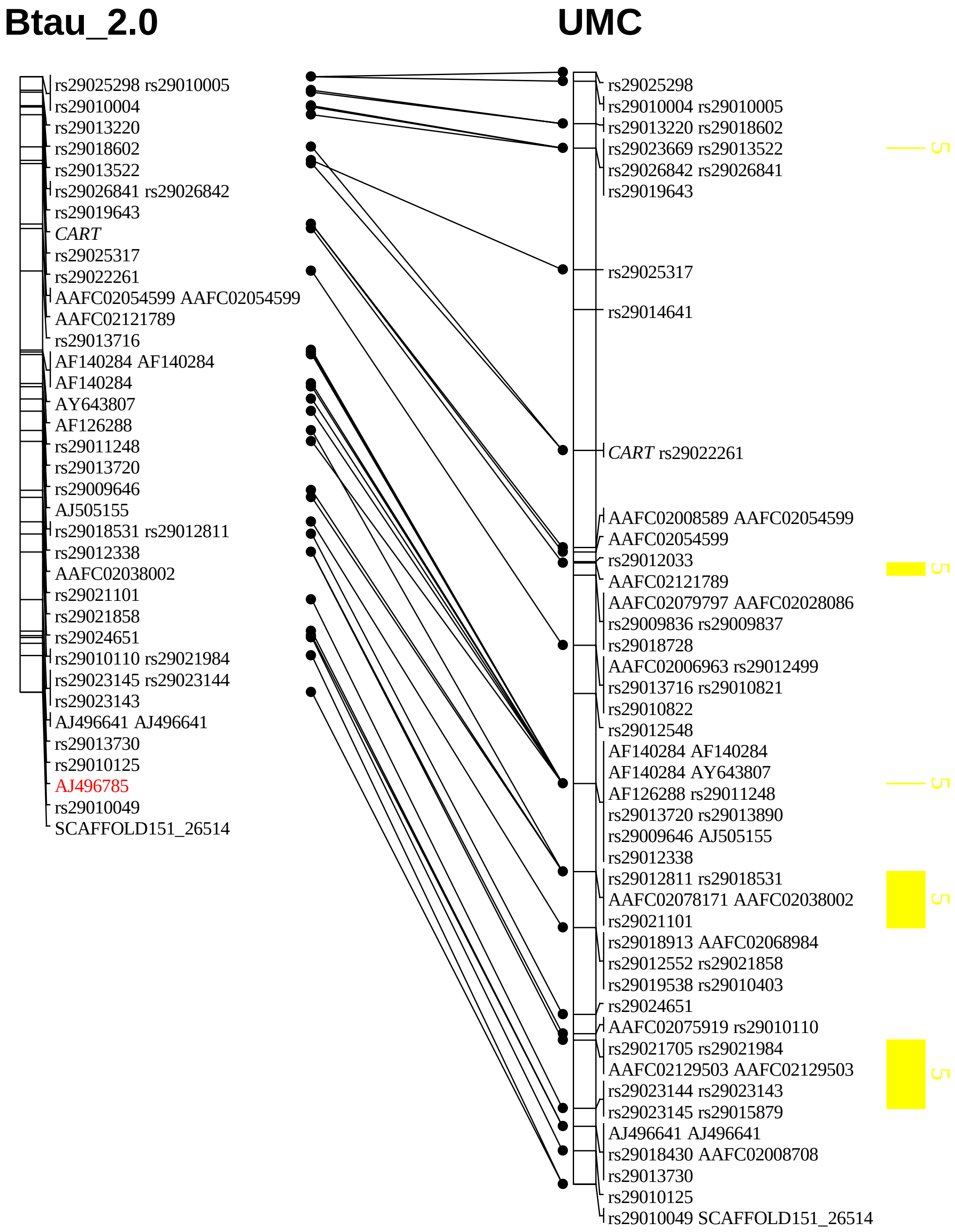
Btau_2.0
UMC
rs29025298 rs29010005
rs29010004
rs29013220
rs29018602
rs29013522
rs29026841 rs29026842
rs29019643
CART
rs29025317
rs29022261
AAFC02054599 AAFC02054599
AAFC02121789
rs29013716
AF140284 AF140284
AF140284
AY643807
AF126288
rs29011248
rs29013720
rs29009646
AJ505155
rs29018531 rs29012811
rs29012338
AAFC02038002
rs29021101
rs29021858
rs29024651
rs29010110 rs29021984
rs29023145 rs29023144
rs29023143
AJ496641 AJ496641
rs29013730
rs29010125
AJ496785
rs29010049
SCAFFOLD151_26514
rs29025298
rs29010004 rs29010005
rs29013220 rs29018602
rs29023669 rs29013522
rs29026842 rs29026841
rs29019643
rs29025317
rs29014641
CART rs29022261
AAFC02008589 AAFC02054599
AAFC02054599
rs29012033
AAFC02121789
AAFC02079797 AAFC02028086
rs29009836 rs29009837
rs29018728
AAFC02006963 rs29012499
rs29013716 rs29010821
rs29010822
rs29012548
AF140284 AF140284
AF140284 AY643807
AF126288 rs29011248
rs29013720 rs29013890
rs29009646 AJ505155
rs29012338
rs29012811 rs29018531
AAFC02078171 AAFC02038002
rs29021101
rs29018913 AAFC02068984
rs29012552 rs29021858
rs29019538 rs29010403
rs29024651
AAFC02075919 rs29010110
rs29021705 rs29021984
AAFC02129503 AAFC02129503
rs29023144 rs29023143
rs29023145 rs29015879
AJ496641 AJ496641
rs29018430 AAFC02008708
rs29013730
rs29010125
rs29010049 SCAFFOLD151_26514
5
5
5
5
5

BTA21

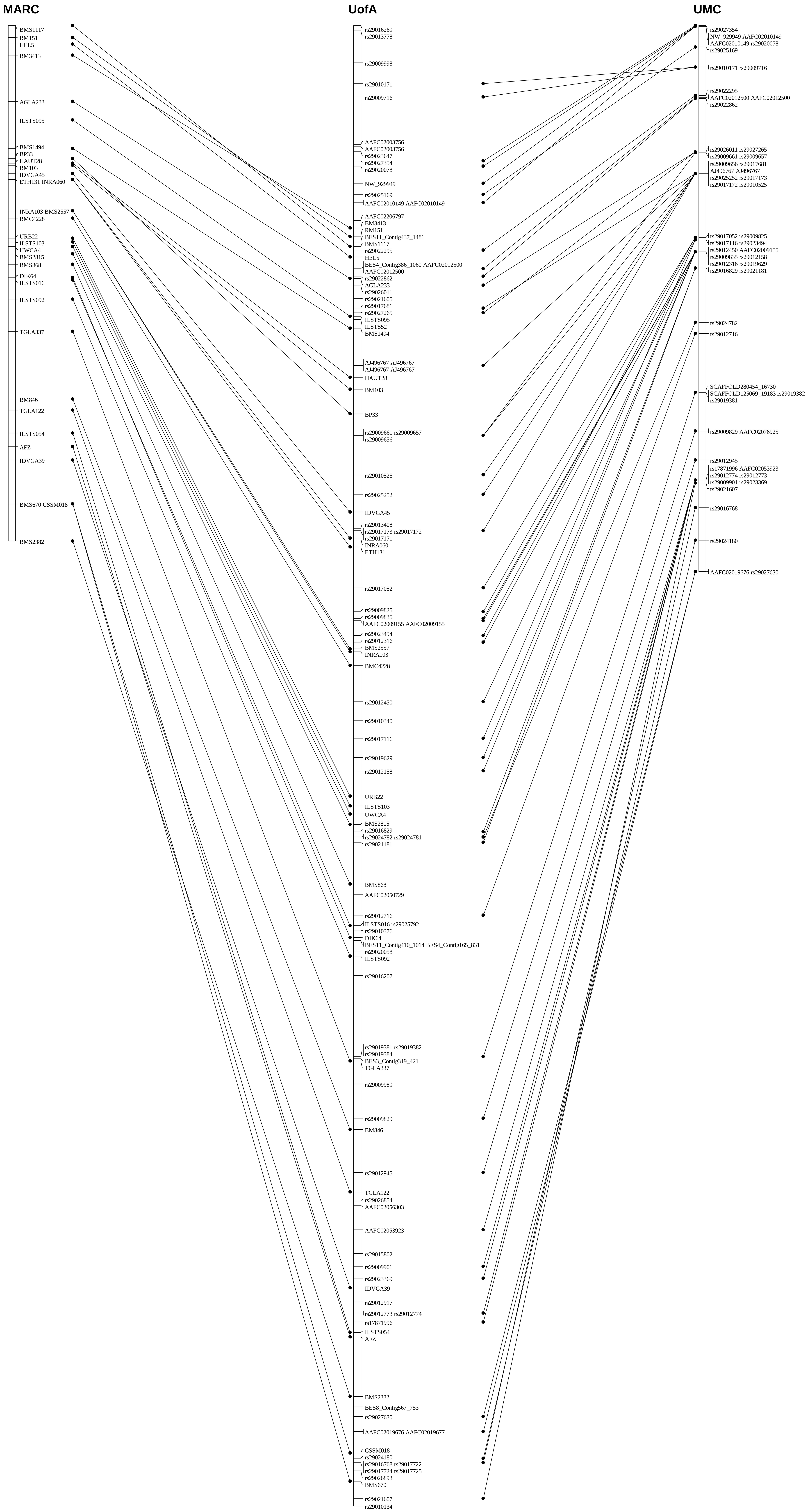
MARC
BMS1117
RM151
HEL5
BM3413
AGLA233
ILSTS095
BMS1494
BP33
HAUT28
BM103
IDVGA45
ETH131 INRA060
INRA103 BMS2557
BMC4228
URB22
ILSTS103
UWCA4
BMS2815
BMS868
DIK64
ILSTS016
ILSTS092
TGLA337
BM846
TGLA122
ILSTS054
AFZ
IDVGA39
BMS670 CSSM018
BMS2382
UofA
rs29016269
rs29013778
rs29009998
rs29010171
rs29009716
AAFC02003756
AAFC02003756
rs29023647
rs29027354
rs29020078
NW_929949
rs29025169
AAFC02010149 AAFC02010149
AAFC02206797
BM3413
RM151
BES11_Contig437_1481
BMS1117
rs29022295
HEL5
BES4_Contig386_1060 AAFC02012500
AAFC02012500
rs29022862
AGLA233
rs29026011
rs29021605
rs29017681
rs29027265
ILSTS095
ILSTS52
BMS1494
AJ496767 AJ496767
AJ496767 AJ496767
HAUT28
BM103
BP33
rs29009661 rs29009657
rs29009656
rs29010525
rs29025252
IDVGA45
rs29013408
rs29017173 rs29017172
rs29017171
INRA060
ETH131
rs29017052
rs29009825
rs29009835
AAFC02009155 AAFC02009155
rs29023494
rs29012316
BMS2557
INRA103
BMC4228
rs29012450
rs29010340
rs29017116
rs29019629
rs29012158
URB22
ILSTS103
UWCA4
BMS2815
rs29016829
rs29024782 rs29024781
rs29021181
BMS868
AAFC02050729
rs29012716
ILSTS016 rs29025792
rs29010376
DIK64
BES11_Contig410_1014 BES4_Contig165_831
rs29020058
ILSTS092
rs29016207
rs29019381 rs29019382
rs29019384
BES3_Contig319_421
TGLA337
rs29009989
rs29009829
BM846
rs29012945
TGLA122
rs29026854
AAFC02056303
AAFC02053923
rs29015802
rs29009901
rs29023369
IDVGA39
rs29012917
rs29012773 rs29012774
rs17871996
ILSTS054
AFZ
BMS2382
BES8_Contig567_753
rs29027630
AAFC02019676 AAFC02019677
CSSM018
rs29024180
rs29016768 rs29017722
rs29017724 rs29017725
rs29026893
BMS670
rs29021607
rs29010134
UMC
rs29027354
NW_929949 AAFC02010149
AAFC02010149 rs29020078
rs29025169
rs29010171 rs29009716
rs29022295
AAFC02012500 AAFC02012500
rs29022862
rs29026011 rs29027265
rs29009661 rs29009657
rs29009656 rs29017681
AJ496767 AJ496767
rs29025252 rs29017173
rs29017172 rs29010525
rs29017052 rs29009825
rs29017116 rs29023494
rs29012450 AAFC02009155
rs29009835 rs29012158
rs29012316 rs29019629
rs29016829 rs29021181
rs29024782
rs29012716
SCAFFOLD280454_16730
SCAFFOLD125069_19183 rs29019382
rs29019381
rs29009829 AAFC02076925
rs29012945
rs17871996 AAFC02053923
rs29012774 rs29012773
rs29009901 rs29023369
rs29021607
rs29016768
rs29024180
AAFC02019676 rs29027630

BTA21

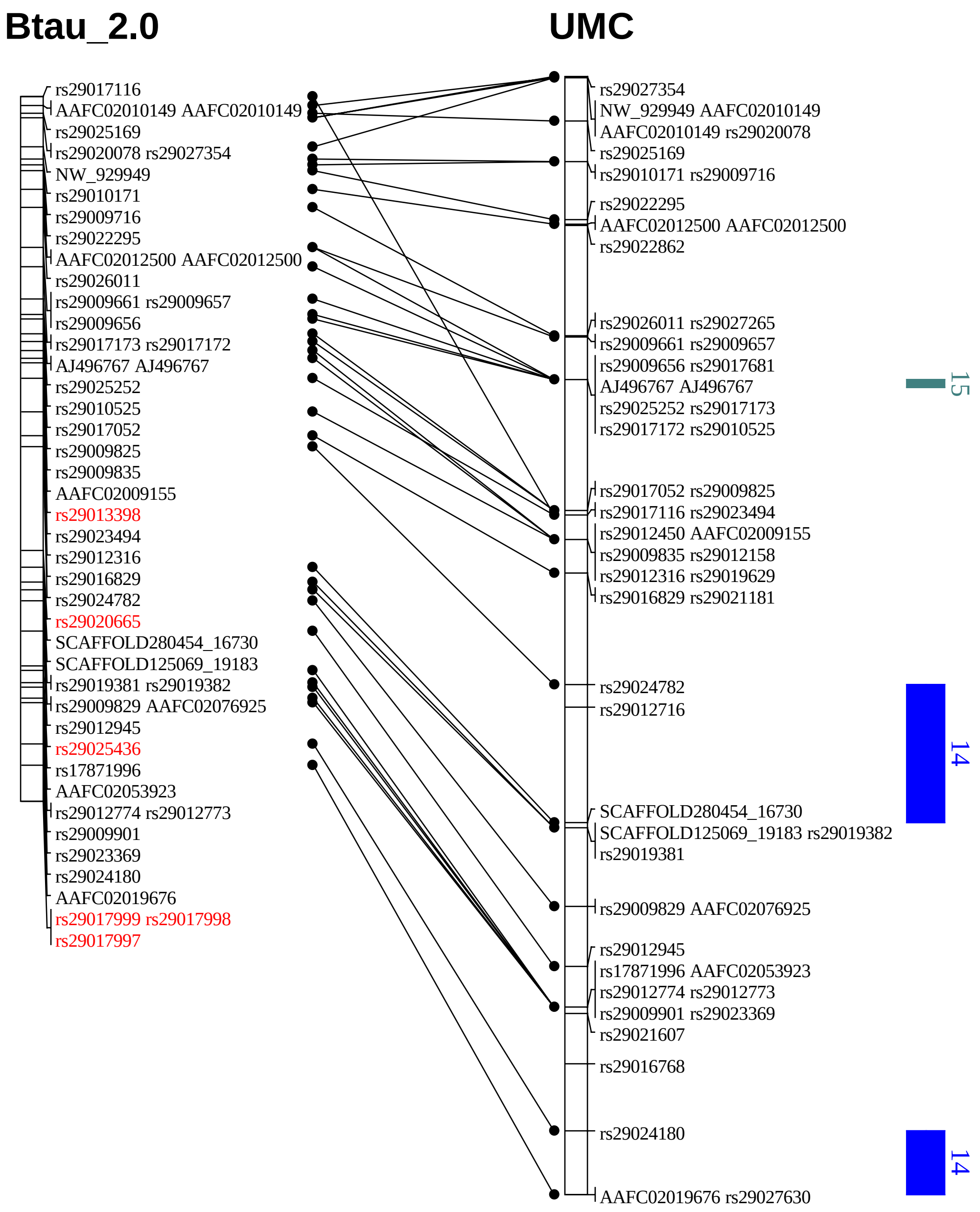
Btau_2.0
UMC
rs29017116
AAFC02010149 AAFC02010149
rs29025169
rs29020078 rs29027354
NW_929949
rs29010171
rs29009716
rs29022295
AAFC02012500 AAFC02012500
rs29026011
rs29009661 rs29009657
rs29009656
rs29017173 rs29017172
AJ496767 AJ496767
rs29025252
rs29010525
rs29017052
rs29009825
rs29009835
AAFC02009155
rs29013398
rs29023494
rs29012316
rs29016829
rs29024782
rs29020665
SCAFFOLD280454_16730
SCAFFOLD125069_19183
rs29019381 rs29019382
rs29009829 AAFC02076925
rs29012945
rs29025436
rs17871996
AAFC02053923
rs29012774 rs29012773
rs29009901
rs29023369
rs29024180
AAFC02019676
rs29017999 rs29017998
rs29017997
rs29027354
NW_929949 AAFC02010149
AAFC02010149 rs29020078
rs29025169
rs29010171 rs29009716
rs29022295
AAFC02012500 AAFC02012500
rs29022862
rs29026011 rs29027265
rs29009661 rs29009657
rs29009656 rs29017681
AJ496767 AJ496767
rs29025252 rs29017173
rs29017172 rs29010525
rs29017052 rs29009825
rs29017116 rs29023494
rs29012450 AAFC02009155
rs29009835 rs29012158
rs29012316 rs29019629
rs29016829 rs29021181
rs29024782
rs29012716
SCAFFOLD280454_16730
SCAFFOLD125069_19183 rs29019382
rs29019381
rs29009829 AAFC02076925
rs29012945
rs17871996 AAFC02053923
rs29012774 rs29012773
rs29009901 rs29023369
rs29021607
rs29016768
rs29024180
AAFC02019676 rs29027630
15
14
14

BTA22

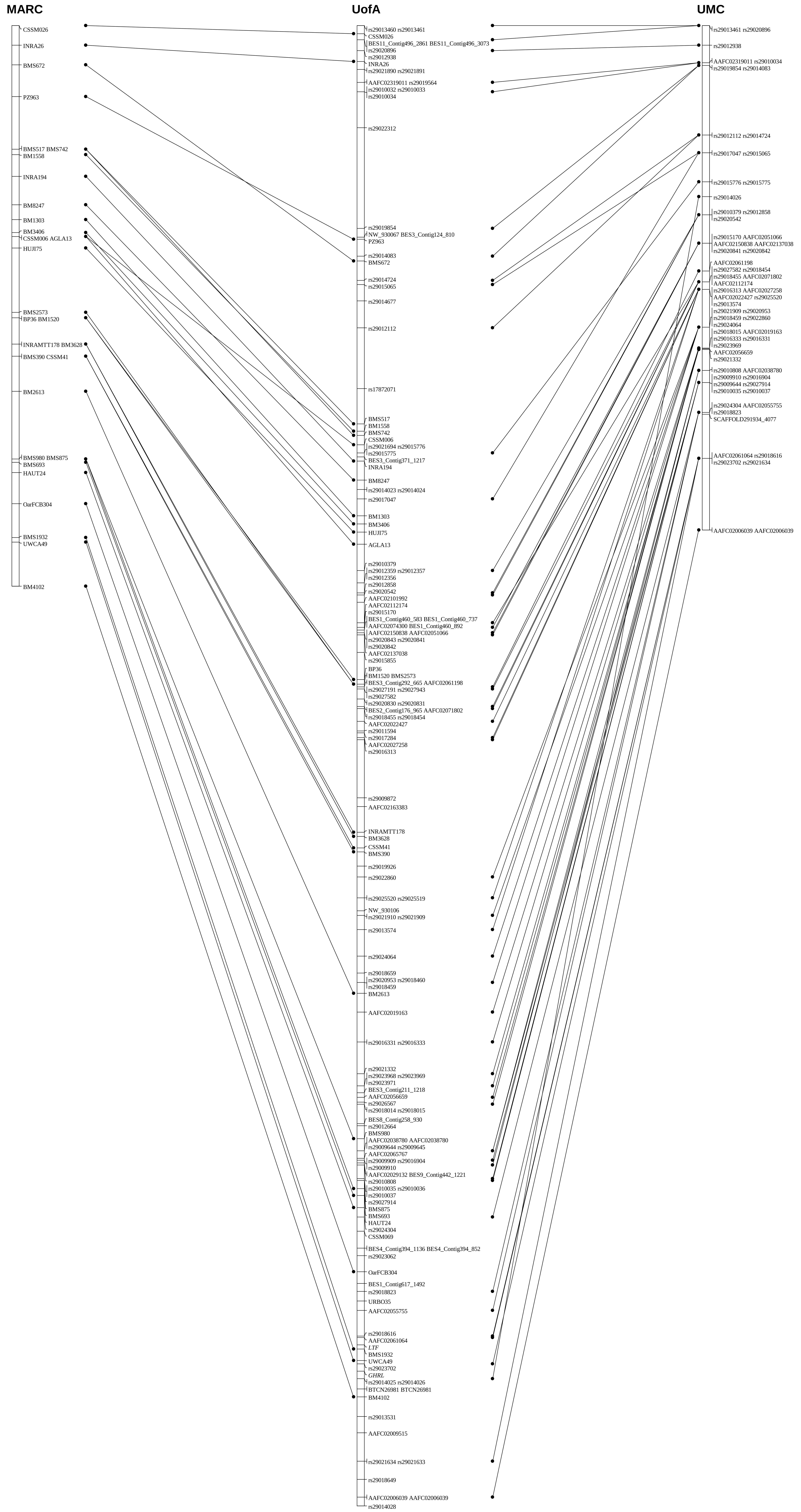

MARC
UofA
UMC
CSSM026
INRA26
BMS672
PZ963
BMS517 BMS742
BM1558
INRA194
BM8247
BM1303
BM3406
CSSM006 AGLA13
HUJI75
BMS2573
BP36 BM1520
INRAMTT178 BM3628
BMS390 CSSM41
BM2613
BMS980 BMS875
BMS693
HAUT24
OarFCB304
BMS1932
UWCA49
BM4102
rs29013460 rs29013461
CSSM026
BES11_Contig496_2861 BES11_Contig496_3073
rs29020896
rs29012938
INRA26
rs29021890 rs29021891
AAFC02319011 rs29019564
rs29010032 rs29010033
rs29010034
rs29022312
rs29019854
NW_930067 BES3_Contig124_810
PZ963
rs29014083
BMS672
rs29014724
rs29015065
rs29014677
rs29012112
rs17872071
BMS517
BM1558
BMS742
CSSM006
rs29021694 rs29015776
rs29015775
BES3_Contig371_1217
INRA194
BM8247
rs29014023 rs29014024
rs29017047
BM1303
BM3406
HUJI75
AGLA13
rs29010379
rs29012359 rs29012357
rs29012356
rs29012858
rs29020542
AAFC02101992
AAFC02112174
rs29015170
BES1_Contig460_583 BES1_Contig460_737
AAFC02074300 BES1_Contig460_892
AAFC02150838 AAFC02051066
rs29020843 rs29020841
rs29020842
AAFC02137038
rs29015855
BP36
BM1520 BMS2573
BES3_Contig292_665 AAFC02061198
rs29027191 rs29027943
rs29027582
rs29020830 rs29020831
BES2_Contig176_965 AAFC02071802
rs29018455 rs29018454
AAFC02022427
rs29011594
rs29017284
AAFC02027258
rs29016313
rs29009872
AAFC02163383
INRAMTT178
BM3628
CSSM41
BMS390
rs29019926
rs29022860
rs29025520 rs29025519
NW_930106
rs29021910 rs29021909
rs29013574
rs29024064
rs29018659
rs29020953 rs29018460
rs29018459
BM2613
AAFC02019163
rs29016331 rs29016333
rs29021332
rs29023968 rs29023969
rs29023971
BES3_Contig211_1218
AAFC02056659
rs29026567
rs29018014 rs29018015
BES8_Contig258_930
rs29012664
BMS980
AAFC02038780 AAFC02038780
rs29009644 rs29009645
AAFC02065767
rs29009909 rs29016904
rs29009910
AAFC02029132 BES9_Contig442_1221
rs29010808
rs29010035 rs29010036
rs29010037
rs29027914
BMS875
BMS693
HAUT24
rs29024304
CSSM069
BES4_Contig394_1136 BES4_Contig394_852
rs29023062
OarFCB304
BES1_Contig617_1492
rs29018823
URBO35
AAFC02055755
rs29018616
AAFC02061064
LTF
BMS1932
UWCA49
rs29023702
GHRL
rs29014025 rs29014026
BTCN26981 BTCN26981
BM4102
rs29013531
AAFC02009515
rs29021634 rs29021633
rs29018649
AAFC02006039 AAFC02006039
rs29014028
rs29013461 rs29020896
rs29012938
AAFC02319011 rs29010034
rs29019854 rs29014083
rs29012112 rs29014724
rs29017047 rs29015065
rs29015776 rs29015775
rs29014026
rs29010379 rs29012858
rs29020542
rs29015170 AAFC02051066
AAFC02150838 AAFC02137038
rs29020841 rs29020842
AAFC02061198
rs29027582 rs29018454
rs29018455 AAFC02071802
AAFC02112174
rs29016313 AAFC02027258
AAFC02022427 rs29025520
rs29013574
rs29021909 rs29020953
rs29018459 rs29022860
rs29024064
rs29018015 AAFC02019163
rs29016333 rs29016331
rs29023969
AAFC02056659
rs29021332
rs29010808 AAFC02038780
rs29009910 rs29016904
rs29009644 rs29027914
rs29010035 rs29010037
rs29024304 AAFC02055755
rs29018823
SCAFFOLD291934_4077
AAFC02061064 rs29018616
rs29023702 rs29021634
AAFC02006039 AAFC02006039

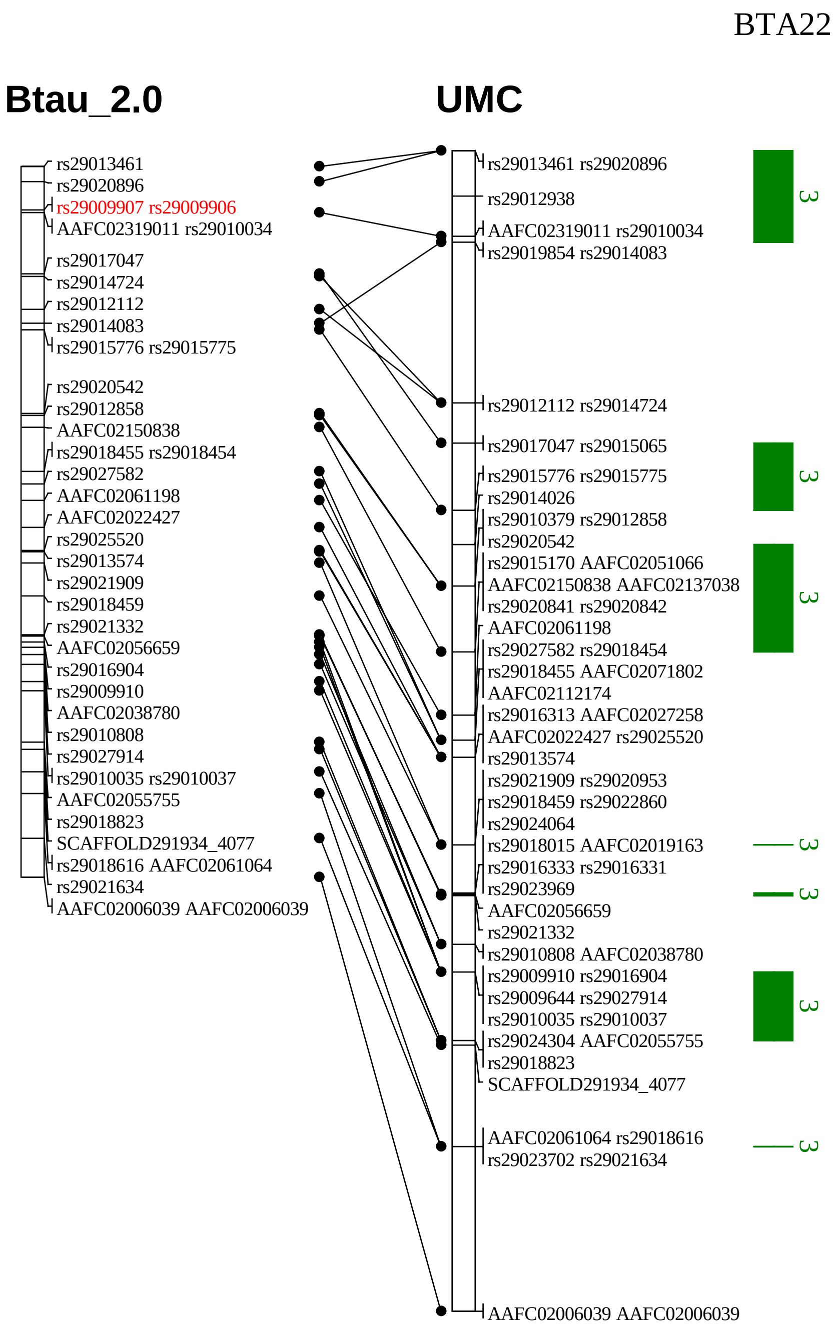

BTA22
Btau_2.0
UMC
rs29013461
rs29020896
rs29009907 rs29009906
AAFC02319011 rs29010034
rs29017047
rs29014724
rs29012112
rs29014083
rs29015776 rs29015775
rs29020542
rs29012858
AAFC02150838
rs29018455 rs29018454
rs29027582
AAFC02061198
AAFC02022427
rs29025520
rs29013574
rs29021909
rs29018459
rs29021332
AAFC02056659
rs29016904
rs29009910
AAFC02038780
rs29010808
rs29027914
rs29010035 rs29010037
AAFC02055755
rs29018823
SCAFFOLD291934_4077
rs29018616 AAFC02061064
rs29021634
AAFC02006039 AAFC02006039
rs29013461 rs29020896
rs29012938
AAFC02319011 rs29010034
rs29019854 rs29014083
rs29012112 rs29014724
rs29017047 rs29015065
rs29015776 rs29015775
rs29014026
rs29010379 rs29012858
rs29020542
rs29015170 AAFC02051066
AAFC02150838 AAFC02137038
rs29020841 rs29020842
AAFC02061198
rs29027582 rs29018454
rs29018455 AAFC02071802
AAFC02112174
rs29016313 AAFC02027258
AAFC02022427 rs29025520
rs29013574
rs29021909 rs29020953
rs29018459 rs29022860
rs29024064
rs29018015 AAFC02019163
rs29016333 rs29016331
rs29023969
AAFC02056659
rs29021332
rs29010808 AAFC02038780
rs29009910 rs29016904
rs29009644 rs29027914
rs29010035 rs29010037
rs29024304 AAFC02055755
rs29018823
SCAFFOLD291934_4077
AAFC02061064 rs29018616
rs29023702 rs29021634
AAFC02006039 AAFC02006039
3
3
3
3
3
3
3

BTA23

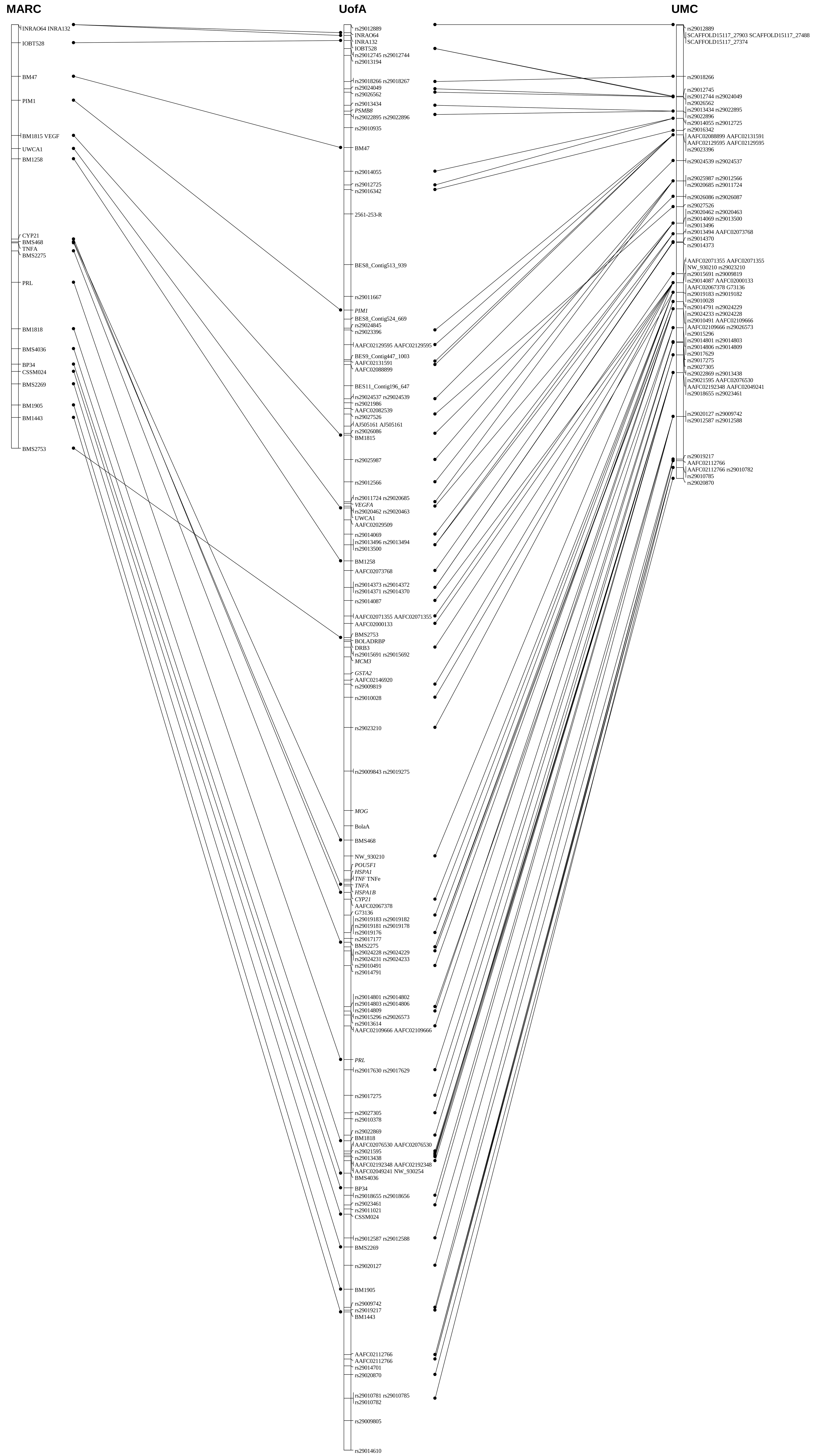
MARC
INRAO64 INRA132
IOBT528
BM47
PIM1
BM1815 VEGF
UWCA1
BM1258
CYP21
BMS468
TNFA
BMS2275
PRL
BM1818
BMS4036
BP34
CSSM024
BMS2269
BM1905
BM1443
BMS2753
UofA
rs29012889
INRAO64
INRA132
IOBT528
rs29012745 rs29012744
rs29013194
rs29018266 rs29018267
rs29024049
rs29026562
rs29013434
PSMB8
rs29022895 rs29022896
rs29010935
BM47
rs29014055
rs29012725
rs29016342
2561-253-R
BES8_Contig513_939
rs29011667
PIM1
BES8_Contig524_669
rs29024845
rs29023396
AAFC02129595 AAFC02129595
BES9_Contig447_1003
AAFC02131591
AAFC02088899
BES11_Contig196_647
rs29024537 rs29024539
rs29021986
AAFC02082539
rs29027526
AJ505161 AJ505161
rs29026086
BM1815
rs29025987
rs29012566
rs29011724 rs29020685
VEGFA
rs29020462 rs29020463
UWCA1
AAFC02029509
rs29014069
rs29013496 rs29013494
rs29013500
BM1258
AAFC02073768
rs29014373 rs29014372
rs29014371 rs29014370
rs29014087
AAFC02071355 AAFC02071355
AAFC02000133
BMS2753
BOLADRBP
DRB3
rs29015691 rs29015692
MCM3
GSTA2
AAFC02146920
rs29009819
rs29010028
rs29023210
rs29009843 rs29019275
MOG
BolaA
BMS468
NW_930210
POU5F1
HSPA1
TNF TNFe
TNFA
HSPA1B
CYP21
AAFC02067378
G73136
rs29019183 rs29019182
rs29019181 rs29019178
rs29019176
rs29017177
BMS2275
rs29024228 rs29024229
rs29024231 rs29024233
rs29010491
rs29014791
rs29014801 rs29014802
rs29014803 rs29014806
rs29014809
rs29015296 rs29026573
rs29013614
AAFC02109666 AAFC02109666
PRL
rs29017630 rs29017629
rs29017275
rs29027305
rs29010378
rs29022869
BM1818
AAFC02076530 AAFC02076530
rs29021595
rs29013438
AAFC02192348 AAFC02192348
AAFC02049241 NW_930254
BMS4036
BP34
rs29018655 rs29018656
rs29023461
rs29011021
CSSM024
rs29012587 rs29012588
BMS2269
rs29020127
BM1905
rs29009742
rs29019217
BM1443
AAFC02112766
AAFC02112766
rs29014701
rs29020870
rs29010781 rs29010785
rs29010782
rs29009805
rs29014610
UMC
rs29012889
SCAFFOLD15117_27903 SCAFFOLD15117_27488
SCAFFOLD15117_27374
rs29018266
rs29012745
rs29012744 rs29024049
rs29026562
rs29013434 rs29022895
rs29022896
rs29014055 rs29012725
rs29016342
AAFC02088899 AAFC02131591
AAFC02129595 AAFC02129595
rs29023396
rs29024539 rs29024537
rs29025987 rs29012566
rs29020685 rs29011724
rs29026086 rs29026087
rs29027526
rs29020462 rs29020463
rs29014069 rs29013500
rs29013496
rs29013494 AAFC02073768
rs29014370
rs29014373
AAFC02071355 AAFC02071355
NW_930210 rs29023210
rs29015691 rs29009819
rs29014087 AAFC02000133
AAFC02067378 G73136
rs29019183 rs29019182
rs29010028
rs29014791 rs29024229
rs29024233 rs29024228
rs29010491 AAFC02109666
AAFC02109666 rs29026573
rs29015296
rs29014801 rs29014803
rs29014806 rs29014809
rs29017629
rs29017275
rs29027305
rs29022869 rs29013438
rs29021595 AAFC02076530
AAFC02192348 AAFC02049241
rs29018655 rs29023461
rs29020127 rs29009742
rs29012587 rs29012588
rs29019217
AAFC02112766
AAFC02112766 rs29010782
rs29010785
rs29020870

BTA23

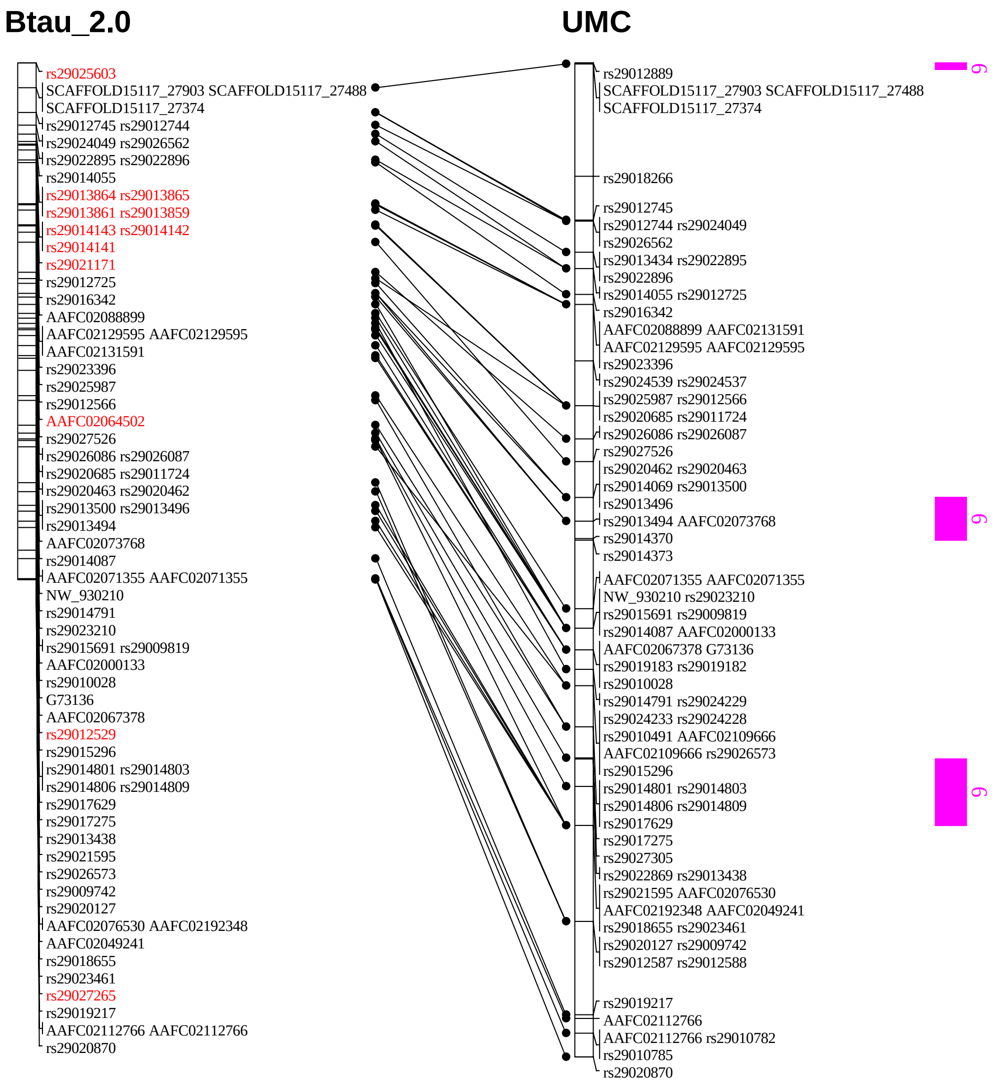

Btau_2.0
UMC
rs29025603
SCAFFOLD15117_27903 SCAFFOLD15117_27488
SCAFFOLD15117_27374
rs29012745 rs29012744
rs29024049 rs29026562
rs29022895 rs29022896
rs29014055
rs29013864 rs29013865
rs29013861 rs29013859
rs29014143 rs29014142
rs29014141
rs29021171
rs29012725
rs29016342
AAFC02088899
AAFC02129595 AAFC02129595
AAFC02131591
rs29023396
rs29025987
rs29012566
AAFC02064502
rs29027526
rs29026086 rs29026087
rs29020685 rs29011724
rs29020463 rs29020462
rs29013500 rs29013496
rs29013494
AAFC02073768
rs29014087
AAFC02071355 AAFC02071355
NW_930210
rs29014791
rs29023210
rs29015691 rs29009819
AAFC02000133
rs29010028
G73136
AAFC02067378
rs29012529
rs29015296
rs29014801 rs29014803
rs29014806 rs29014809
rs29017629
rs29017275
rs29013438
rs29021595
rs29026573
rs29009742
rs29020127
AAFC02076530 AAFC02192348
AAFC02049241
rs29018655
rs29023461
rs29027265
rs29019217
AAFC02112766 AAFC02112766
rs29020870
rs29012889
SCAFFOLD15117_27903 SCAFFOLD15117_27488
SCAFFOLD15117_27374
rs29018266
rs29012745
rs29012744 rs29024049
rs29026562
rs29013434 rs29022895
rs29022896
rs29014055 rs29012725
rs29016342
AAFC02088899 AAFC02131591
AAFC02129595 AAFC02129595
rs29023396
rs29024539 rs29024537
rs29025987 rs29012566
rs29020685 rs29011724
rs29026086 rs29026087
rs29027526
rs29020462 rs29020463
rs29014069 rs29013500
rs29013496
rs29013494 AAFC02073768
rs29014370
rs29014373
AAFC02071355 AAFC02071355
NW_930210 rs29023210
rs29015691 rs29009819
rs29014087 AAFC02000133
AAFC02067378 G73136
rs29019183 rs29019182
rs29010028
rs29014791 rs29024229
rs29024233 rs29024228
rs29010491 AAFC02109666
AAFC02109666 rs29026573
rs29015296
rs29014801 rs29014803
rs29014806 rs29014809
rs29017629
rs29017275
rs29027305
rs29022869 rs29013438
rs29021595 AAFC02076530
AAFC02192348 AAFC02049241
rs29018655 rs29023461
rs29020127 rs29009742
rs29012587 rs29012588
rs29019217
AAFC02112766
AAFC02112766 rs29010782
rs29010785
rs29020870
6
6
6

BTA24

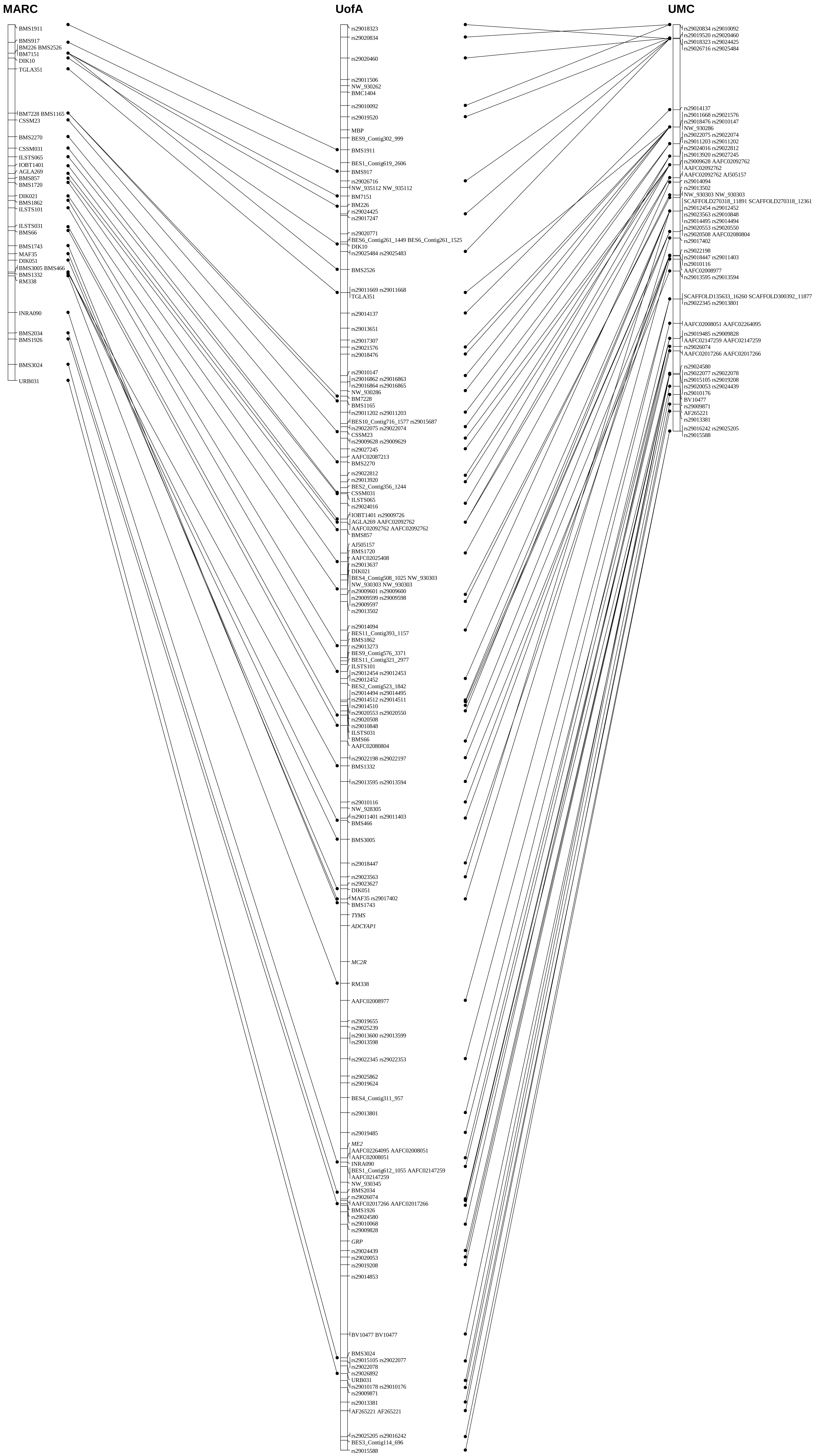
MARC
BMS1911
BMS917
BM226 BMS2526
BM7151
DIK10
TGLA351
BM7228 BMS1165
CSSM23
BMS2270
CSSM031
ILSTS065
IOBT1401
AGLA269
BMS857
BMS1720
DIK021
BMS1862
ILSTS101
ILSTS031
BMS66
BMS1743
MAF35
DIK051
BMS3005 BMS466
BMS1332
RM338
INRA090
BMS2034
BMS1926
BMS3024
URB031
UofA
rs29018323
rs29020834
rs29020460
rs29011506
NW_930262
BMC1404
rs29010092
rs29019520
MBP
BES9_Contig302_999
BMS1911
BES1_Contig619_2606
BMS917
rs29026716
NW_935112 NW_935112
BM7151
BM226
rs29024425
rs29017247
rs29020771
BES6_Contig261_1449 BES6_Contig261_1525
DIK10
rs29025484 rs29025483
BMS2526
rs29011669 rs29011668
TGLA351
rs29014137
rs29013651
rs29017307
rs29021576
rs29018476
rs29010147
rs29016862 rs29016863
rs29016864 rs29016865
NW_930286
BM7228
BMS1165
rs29011202 rs29011203
BES10_Contig716_1577 rs29015687
rs29022075 rs29022074
CSSM23
rs29009628 rs29009629
rs29027245
AAFC02087213
BMS2270
rs29022812
rs29013920
BES2_Contig356_1244
CSSM031
ILSTS065
rs29024016
IOBT1401 rs29009726
AGLA269 AAFC02092762
AAFC02092762 AAFC02092762
BMS857
AJ505157
BMS1720
AAFC02025408
rs29013637
DIK021
BES4_Contig508_1025 NW_930303
NW_930303 NW_930303
rs29009601 rs29009600
rs29009599 rs29009598
rs29009597
rs29013502
rs29014094
BES11_Contig393_1157
BMS1862
rs29013273
BES9_Contig576_3371
BES11_Contig321_2977
ILSTS101
rs29012454 rs29012453
rs29012452
BES2_Contig523_1842
rs29014494 rs29014495
rs29014512 rs29014511
rs29014510
rs29020553 rs29020550
rs29020508
rs29010848
ILSTS031
BMS66
AAFC02080804
rs29022198 rs29022197
BMS1332
rs29013595 rs29013594
rs29010116
NW_928305
rs29011401 rs29011403
BMS466
BMS3005
rs29018447
rs29023563
rs29023627
DIK051
MAF35 rs29017402
BMS1743
TYMS
ADCYAP1
MC2R
RM338
AAFC02008977
rs29019655
rs29025239
rs29013600 rs29013599
rs29013598
rs29022345 rs29022353
rs29025862
rs29019624
BES4_Contig311_957
rs29013801
rs29019485
ME2
AAFC02264095 AAFC02008051
AAFC02008051
INRA090
BES1_Contig612_1055 AAFC02147259
AAFC02147259
NW_930345
BMS2034
rs29026074
AAFC02017266 AAFC02017266
BMS1926
rs29024580
rs29010068
rs29009828
GRP
rs29024439
rs29020053
rs29019208
rs29014853
BV10477 BV10477
BMS3024
rs29015105 rs29022077
rs29022078
rs29026892
URB031
rs29010178 rs29010176
rs29009871
rs29013381
AF265221 AF265221
rs29025205 rs29016242
BES3_Contig114_696
rs29015588
UMC
rs29020834 rs29010092
rs29019520 rs29020460
rs29018323 rs29024425
rs29026716 rs29025484
rs29014137
rs29011668 rs29021576
rs29018476 rs29010147
NW_930286
rs29022075 rs29022074
rs29011203 rs29011202
rs29024016 rs29022812
rs29013920 rs29027245
rs29009628 AAFC02092762
AAFC02092762
AAFC02092762 AJ505157
rs29014094
rs29013502
NW_930303 NW_930303
SCAFFOLD270318_11891 SCAFFOLD270318_12361
rs29012454 rs29012452
rs29023563 rs29010848
rs29014495 rs29014494
rs29020553 rs29020550
rs29020508 AAFC02080804
rs29017402
rs29022198
rs29018447 rs29011403
rs29010116
AAFC02008977
rs29013595 rs29013594
SCAFFOLD135633_16260 SCAFFOLD300392_11877
rs29022345 rs29013801
AAFC02008051 AAFC02264095
rs29019485 rs29009828
AAFC02147259 AAFC02147259
rs29026074
AAFC02017266 AAFC02017266
rs29024580
rs29022077 rs29022078
rs29015105 rs29019208
rs29020053 rs29024439
rs29010176
BV10477
rs29009871
AF265221
rs29013381
rs29016242 rs29025205
rs29015588

BTA24

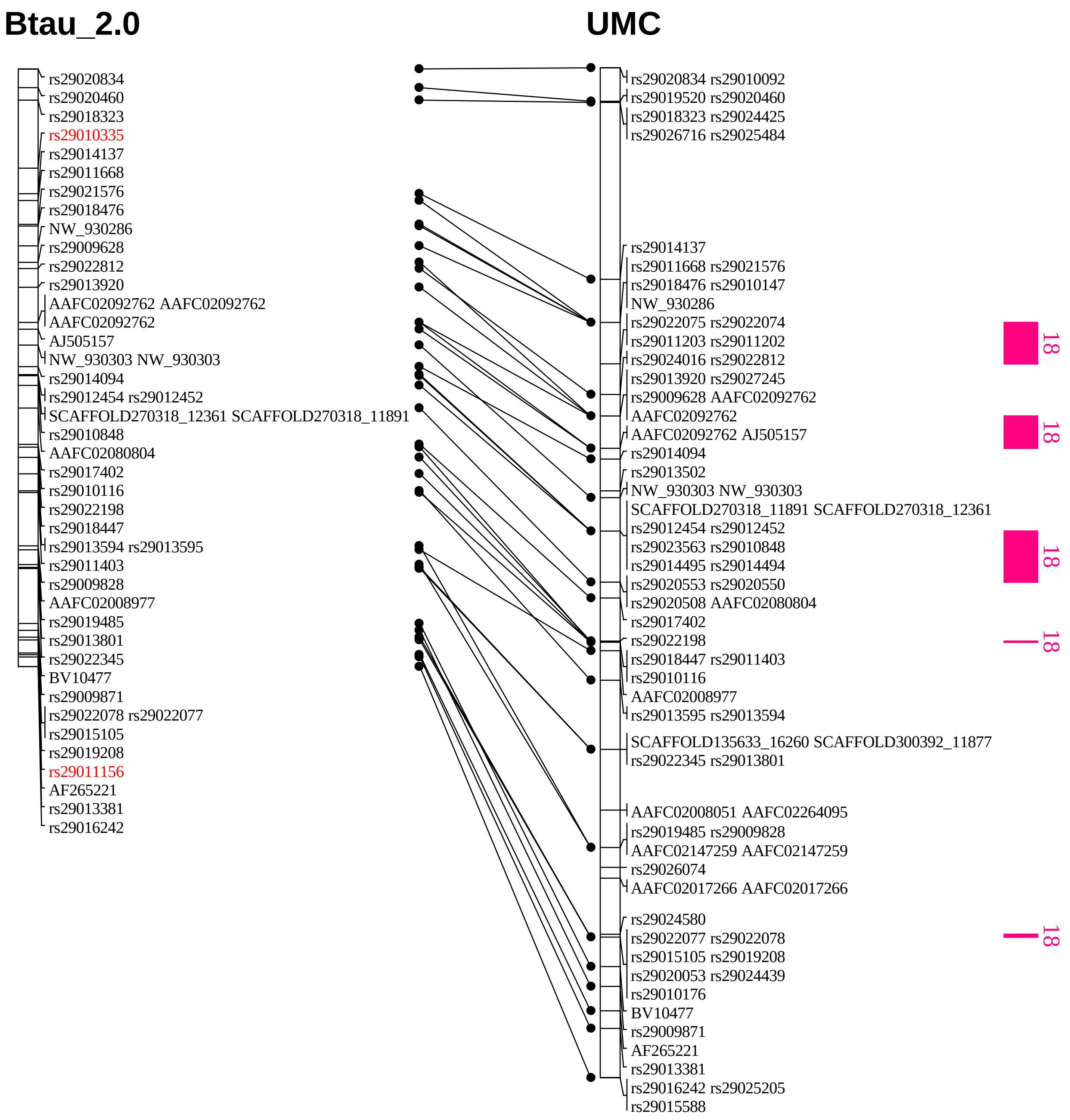

Btau_2.0
UMC
rs29020834
rs29020460
rs29018323
rs29010335
rs29014137
rs29011668
rs29021576
rs29018476
NW_930286
rs29009628
rs29022812
rs29013920
AAFC02092762 AAFC02092762
AAFC02092762
AJ505157
NW_930303 NW_930303
rs29014094
rs29012454 rs29012452
SCAFFOLD270318_12361 SCAFFOLD270318_11891
rs29010848
AAFC02080804
rs29017402
rs29010116
rs29022198
rs29018447
rs29013594 rs29013595
rs29011403
rs29009828
AAFC02008977
rs29019485
rs29013801
rs29022345
BV10477
rs29009871
rs29022078 rs29022077
rs29015105
rs29019208
rs29011156
AF265221
rs29013381
rs29016242
rs29020834 rs29010092
rs29019520 rs29020460
rs29018323 rs29024425
rs29026716 rs29025484
rs29014137
rs29011668 rs29021576
rs29018476 rs29010147
NW_930286
rs29022075 rs29022074
rs29011203 rs29011202
rs29024016 rs29022812
rs29013920 rs29027245
rs29009628 AAFC02092762
AAFC02092762
AAFC02092762 AJ505157
rs29014094
rs29013502
NW_930303 NW_930303
SCAFFOLD270318_11891 SCAFFOLD270318_12361
rs29012454 rs29012452
rs29023563 rs29010848
rs29014495 rs29014494
rs29020553 rs29020550
rs29020508 AAFC02080804
rs29017402
rs29022198
rs29018447 rs29011403
rs29010116
AAFC02008977
rs29013595 rs29013594
SCAFFOLD135633_16260 SCAFFOLD300392_11877
rs29022345 rs29013801
AAFC02008051 AAFC02264095
rs29019485 rs29009828
AAFC02147259 AAFC02147259
rs29026074
AAFC02017266 AAFC02017266
rs29024580
rs29022077 rs29022078
rs29015105 rs29019208
rs29020053 rs29024439
rs29010176
BV10477
rs29009871
AF265221
rs29013381
rs29016242 rs29025205
rs29015588
18
18
18
18
18

BTA25

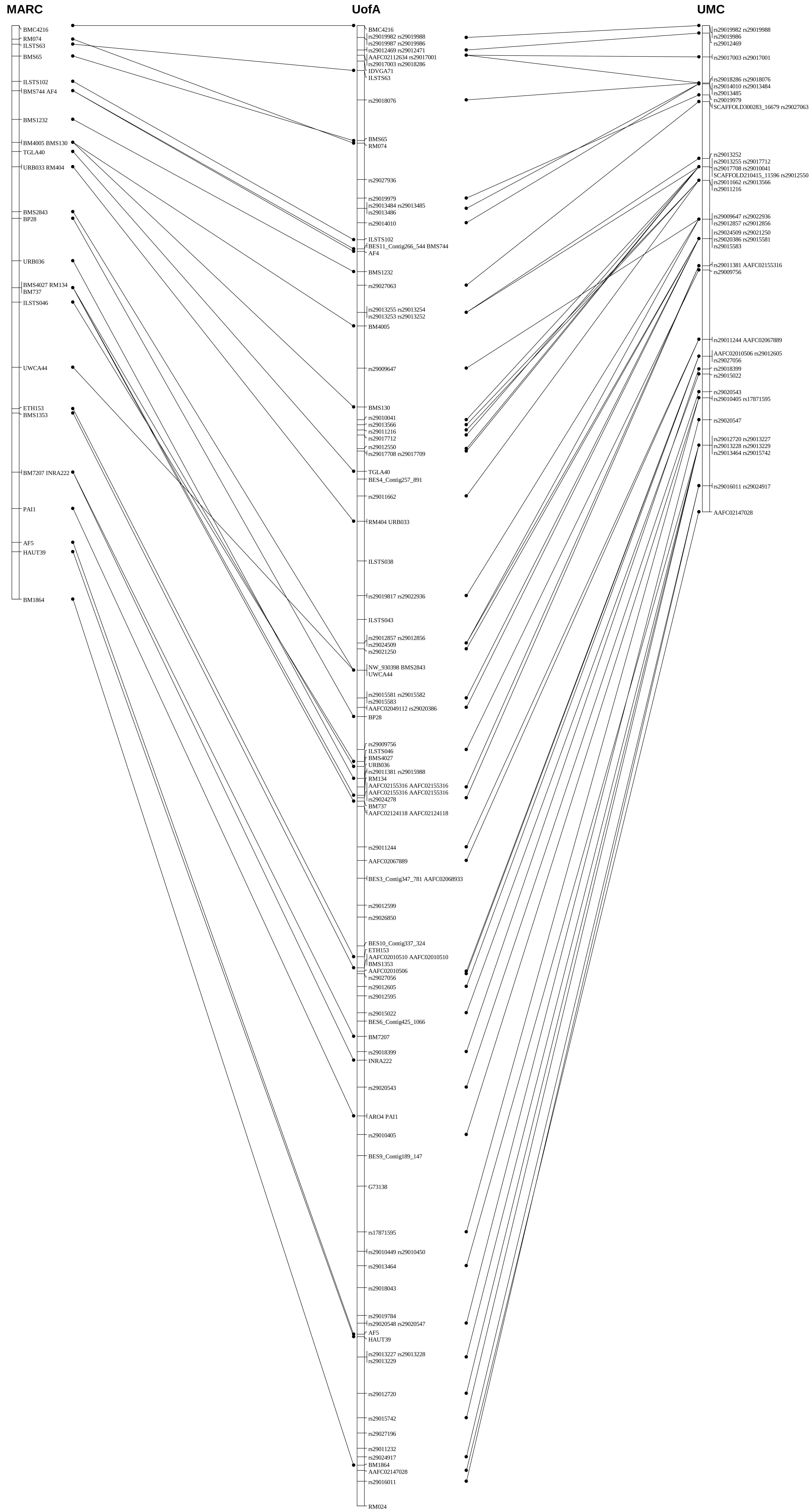
MARC
BMC4216
RM074
ILSTS63
BMS65
ILSTS102
BMS744 AF4
BMS1232
BM4005 BMS130
TGLA40
URB033 RM404
BMS2843
BP28
URB036
BMS4027 RM134
BM737
ILSTS046
UWCA44
ETH153
BMS1353
BM7207 INRA222
PAI1
AF5
HAUT39
BM1864
UofA
BMC4216
rs29019982 rs29019988
rs29019987 rs29019986
rs29012469 rs29012471
AAFC02112634 rs29017001
rs29017003 rs29018286
IDVGA71
ILSTS63
rs29018076
BMS65
RM074
rs29027936
rs29019979
rs29013484 rs29013485
rs29013486
rs29014010
ILSTS102
BES11_Contig266_544 BMS744
AF4
BMS1232
rs29027063
rs29013255 rs29013254
rs29013253 rs29013252
BM4005
rs29009647
BMS130
rs29010041
rs29013566
rs29011216
rs29017712
rs29012550
rs29017708 rs29017709
TGLA40
BES4_Contig257_891
rs29011662
RM404 URB033
ILSTS038
rs29019817 rs29022936
ILSTS043
rs29012857 rs29012856
rs29024509
rs29021250
NW_930398 BMS2843
UWCA44
rs29015581 rs29015582
rs29015583
AAFC02049112 rs29020386
BP28
rs29009756
ILSTS046
BMS4027
URB036
rs29011381 rs29015988
RM134
AAFC02155316 AAFC02155316
AAFC02155316 AAFC02155316
rs29024278
BM737
AAFC02124118 AAFC02124118
rs29011244
AAFC02067889
BES3_Contig347_781 AAFC02068933
rs29012599
rs29026850
BES10_Contig337_324
ETH153
AAFC02010510 AAFC02010510
BMS1353
AAFC02010506
rs29027056
rs29012605
rs29012595
rs29015022
BES6_Contig425_1066
BM7207
rs29018399
INRA222
rs29020543
ARO4 PAI1
rs29010405
BES9_Contig189_147
G73138
rs17871595
rs29010449 rs29010450
rs29013464
rs29018043
rs29019784
rs29020548 rs29020547
AF5
HAUT39
rs29013227 rs29013228
rs29013229
rs29012720
rs29015742
rs29027196
rs29011232
rs29024917
BM1864
AAFC02147028
rs29016011
RM024
UMC
rs29019982 rs29019988
rs29019986
rs29012469
rs29017003 rs29017001
rs29018286 rs29018076
rs29014010 rs29013484
rs29013485
rs29019979
SCAFFOLD300283_16679 rs29027063
rs29013252
rs29013255 rs29017712
rs29017708 rs29010041
SCAFFOLD210415_11596 rs29012550
rs29011662 rs29013566
rs29011216
rs29009647 rs29022936
rs29012857 rs29012856
rs29024509 rs29021250
rs29020386 rs29015581
rs29015583
rs29011381 AAFC02155316
rs29009756
rs29011244 AAFC02067889
AAFC02010506 rs29012605
rs29027056
rs29018399
rs29015022
rs29020543
rs29010405 rs17871595
rs29020547
rs29012720 rs29013227
rs29013228 rs29013229
rs29013464 rs29015742
rs29016011 rs29024917
AAFC02147028

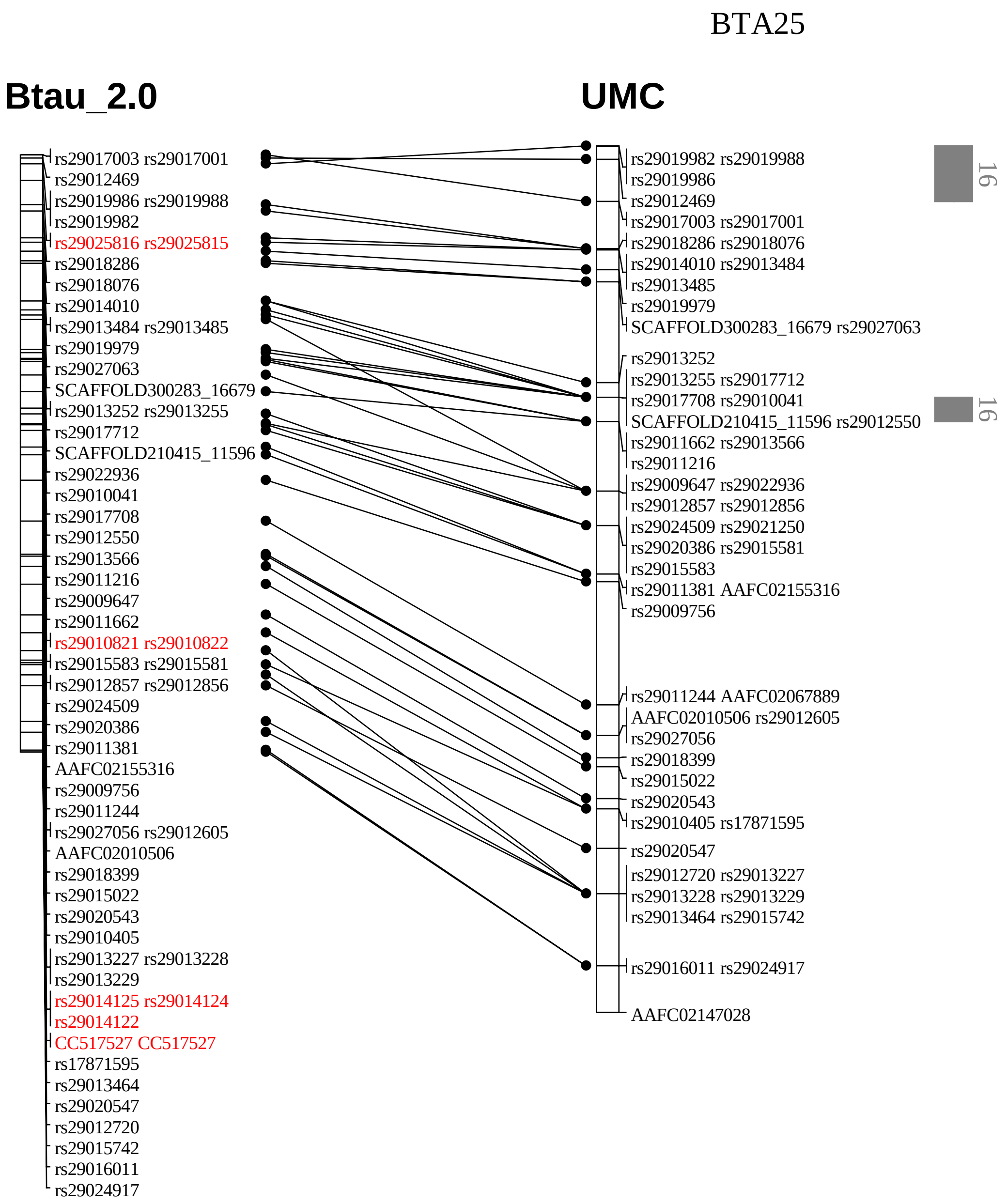
BTA25
Btau_2.0
UMC
rs29017003 rs29017001
rs29012469
rs29019986 rs29019988
rs29019982
rs29025816 rs29025815
rs29018286
rs29018076
rs29014010
rs29013484 rs29013485
rs29019979
rs29027063
SCAFFOLD300283_16679
rs29013252 rs29013255
rs29017712
SCAFFOLD210415_11596
rs29022936
rs29010041
rs29017708
rs29012550
rs29013566
rs29011216
rs29009647
rs29011662
rs29010821 rs29010822
rs29015583 rs29015581
rs29012857 rs29012856
rs29024509
rs29020386
rs29011381
AAFC02155316
rs29009756
rs29011244
rs29027056 rs29012605
AAFC02010506
rs29018399
rs29015022
rs29020543
rs29010405
rs29013227 rs29013228
rs29013229
rs29014125 rs29014124
rs29014122
CC517527 CC517527
rs17871595
rs29013464
rs29020547
rs29012720
rs29015742
rs29016011
rs29024917
rs29019982 rs29019988
rs29019986
rs29012469
rs29017003 rs29017001
rs29018286 rs29018076
rs29014010 rs29013484
rs29013485
rs29019979
SCAFFOLD300283_16679 rs29027063
rs29013252
rs29013255 rs29017712
rs29017708 rs29010041
SCAFFOLD210415_11596 rs29012550
rs29011662 rs29013566
rs29011216
rs29009647 rs29022936
rs29012857 rs29012856
rs29024509 rs29021250
rs29020386 rs29015581
rs29015583
rs29011381 AAFC02155316
rs29009756
rs29011244 AAFC02067889
AAFC02010506 rs29012605
rs29027056
rs29018399
rs29015022
rs29020543
rs29010405 rs17871595
rs29020547
rs29012720 rs29013227
rs29013228 rs29013229
rs29013464 rs29015742
rs29016011 rs29024917
AAFC02147028
16
16

BTA26

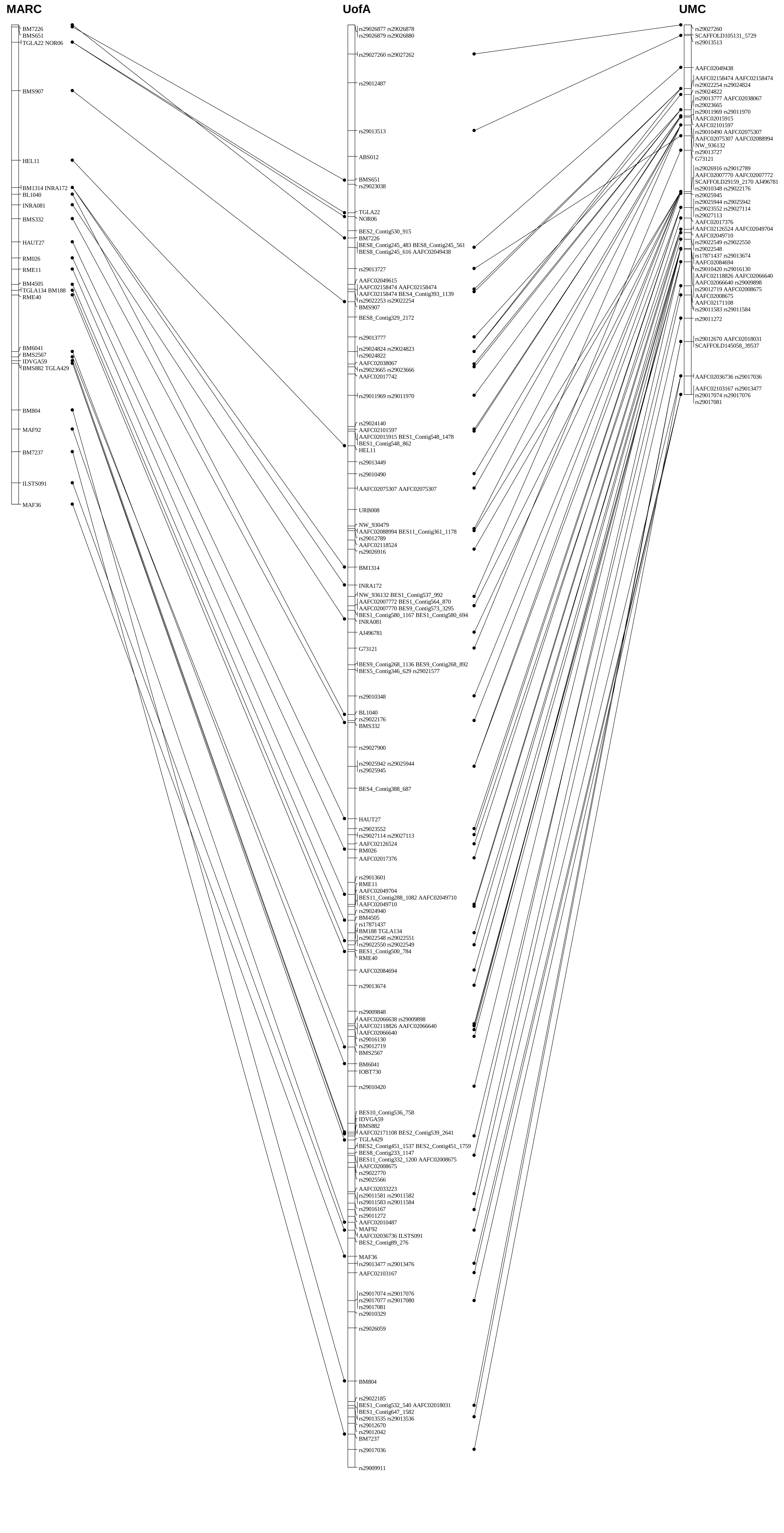
MARC
UofA
UMC
BM7226
BMS651
TGLA22 NOR06
BMS907
HEL11
BM1314 INRA172
BL1040
INRA081
BMS332
HAUT27
RM026
RME11
BM4505
TGLA134 BM188
RME40
BM6041
BMS2567
IDVGA59
BMS882 TGLA429
BM804
MAF92
BM7237
ILSTS091
MAF36
rs29026877 rs29026878
rs29026879 rs29026880
rs29027260 rs29027262
rs29012487
rs29013513
ABS012
BMS651
rs29023038
TGLA22
NOR06
BES2_Contig530_915
BM7226
BES8_Contig245_483 BES8_Contig245_561
BES8_Contig245_616 AAFC02049438
rs29013727
AAFC02049615
AAFC02158474 AAFC02158474
AAFC02158474 BES4_Contig393_1139
rs29022253 rs29022254
BMS907
BES8_Contig329_2172
rs29013777
rs29024824 rs29024823
rs29024822
AAFC02038067
rs29023665 rs29023666
AAFC02017742
rs29011969 rs29011970
rs29024140
AAFC02101597
AAFC02015915 BES1_Contig548_1478
BES1_Contig548_862
HEL11
rs29013449
rs29010490
AAFC02075307 AAFC02075307
URB008
NW_930479
AAFC02088994 BES11_Contig361_1178
rs29012789
AAFC02118524
rs29026916
BM1314
INRA172
NW_936132 BES1_Contig537_992
AAFC02007772 BES1_Contig564_870
AAFC02007770 BES9_Contig573_3295
BES1_Contig580_1167 BES1_Contig580_694
INRA081
AJ496781
G73121
BES9_Contig268_1136 BES9_Contig268_892
BES5_Contig346_629 rs29021577
rs29010348
BL1040
rs29022176
BMS332
rs29027900
rs29025942 rs29025944
rs29025945
BES4_Contig388_687
HAUT27
rs29023552
rs29027114 rs29027113
AAFC02126524
RM026
AAFC02017376
rs29013601
RME11
AAFC02049704
BES11_Contig288_1082 AAFC02049710
AAFC02049710
rs29024940
BM4505
rs17871437
BM188 TGLA134
rs29022548 rs29022551
rs29022550 rs29022549
BES1_Contig500_784
RME40
AAFC02084694
rs29013674
rs29009848
AAFC02066638 rs29009898
AAFC02118826 AAFC02066640
AAFC02066640
rs29016130
rs29012719
BMS2567
BM6041
IOBT730
rs29010420
BES10_Contig536_758
IDVGA59
BMS882
AAFC02171108 BES2_Contig539_2641
TGLA429
BES2_Contig451_1537 BES2_Contig451_1759
BES8_Contig233_1147
BES11_Contig332_1200 AAFC02008675
AAFC02008675
rs29022770
rs29025566
AAFC02033223
rs29011581 rs29011582
rs29011583 rs29011584
rs29016167
rs29011272
AAFC02010487
MAF92
AAFC02036736 ILSTS091
BES2_Contig89_276
MAF36
rs29013477 rs29013476
AAFC02103167
rs29017074 rs29017076
rs29017077 rs29017080
rs29017081
rs29010329
rs29026059
BM804
rs29022185
BES1_Contig532_540 AAFC02018031
BES1_Contig647_1582
rs29013535 rs29013536
rs29012670
rs29012042
BM7237
rs29017036
rs29009911
rs29027260
SCAFFOLD105131_5729
rs29013513
AAFC02049438
AAFC02158474 AAFC02158474
rs29022254 rs29024824
rs29024822
rs29013777 AAFC02038067
rs29023665
rs29011969 rs29011970
AAFC02015915
AAFC02101597
rs29010490 AAFC02075307
AAFC02075307 AAFC02088994
NW_936132
rs29013727
G73121
rs29026916 rs29012789
AAFC02007770 AAFC02007772
SCAFFOLD29159_2170 AJ496781
rs29010348 rs29022176
rs29025945
rs29025944 rs29025942
rs29023552 rs29027114
rs29027113
AAFC02017376
AAFC02126524 AAFC02049704
AAFC02049710
rs29022549 rs29022550
rs29022548
rs17871437 rs29013674
AAFC02084694
rs29010420 rs29016130
AAFC02118826 AAFC02066640
AAFC02066640 rs29009898
rs29012719 AAFC02008675
AAFC02008675
AAFC02171108
rs29011583 rs29011584
rs29011272
rs29012670 AAFC02018031
SCAFFOLD145058_39537
AAFC02036736 rs29017036
AAFC02103167 rs29013477
rs29017074 rs29017076
rs29017081

BTA26

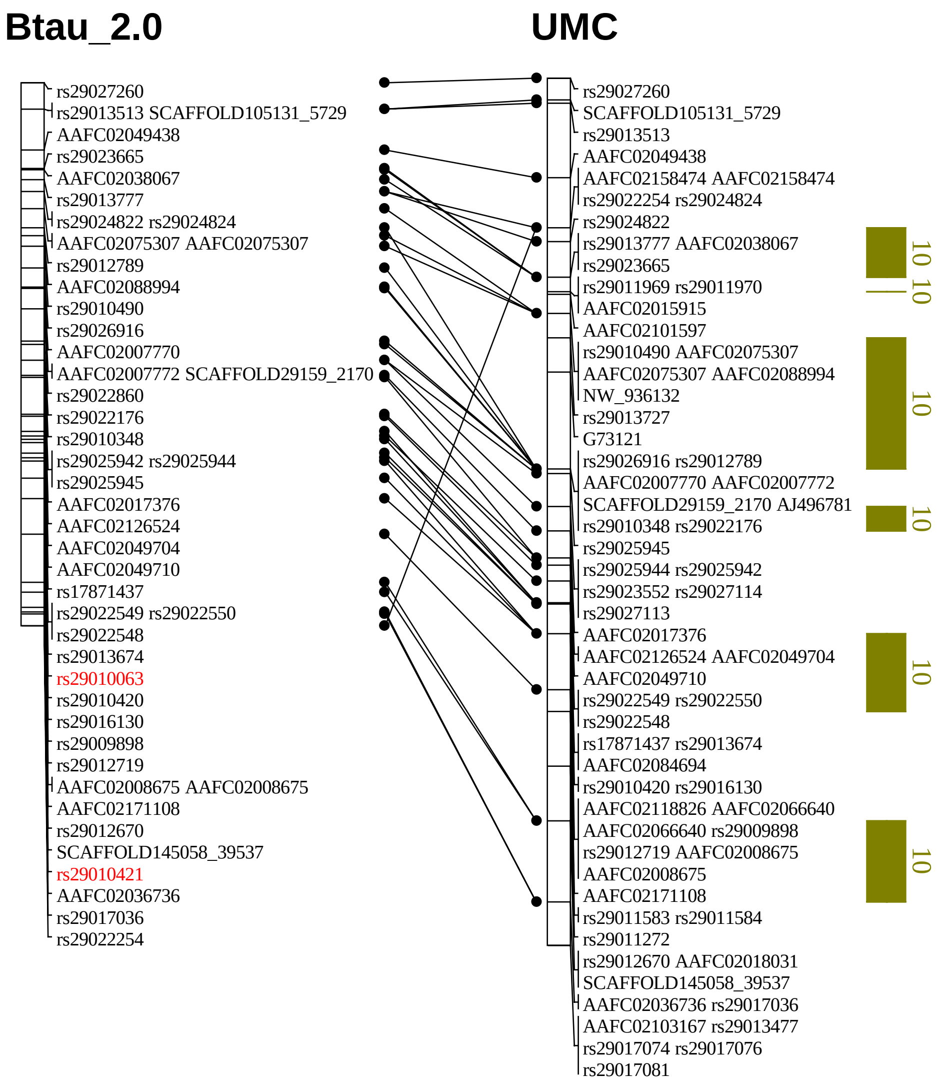
Btau_2.0
UMC
rs29027260
rs29013513 SCAFFOLD105131_5729
AAFC02049438
rs29023665
AAFC02038067
rs29013777
rs29024822 rs29024824
AAFC02075307 AAFC02075307
rs29012789
AAFC02088994
rs29010490
rs29026916
AAFC02007770
AAFC02007772 SCAFFOLD29159_2170
rs29022860
rs29022176
rs29010348
rs29025942 rs29025944
rs29025945
AAFC02017376
AAFC02126524
AAFC02049704
AAFC02049710
rs17871437
rs29022549 rs29022550
rs29022548
rs29013674
rs29010063
rs29010420
rs29016130
rs29009898
rs29012719
AAFC02008675 AAFC02008675
AAFC02171108
rs29012670
SCAFFOLD145058_39537
rs29010421
AAFC02036736
rs29017036
rs29022254
rs29027260
SCAFFOLD105131_5729
rs29013513
AAFC02049438
AAFC02158474 AAFC02158474
rs29022254 rs29024824
rs29024822
rs29013777 AAFC02038067
rs29023665
rs29011969 rs29011970
AAFC02015915
AAFC02101597
rs29010490 AAFC02075307
AAFC02075307 AAFC02088994
NW_936132
rs29013727
G73121
rs29026916 rs29012789
AAFC02007770 AAFC02007772
SCAFFOLD29159_2170 AJ496781
rs29010348 rs29022176
rs29025945
rs29025944 rs29025942
rs29023552 rs29027114
rs29027113
AAFC02017376
AAFC02126524 AAFC02049704
AAFC02049710
rs29022549 rs29022550
rs29022548
rs17871437 rs29013674
AAFC02084694
rs29010420 rs29016130
AAFC02118826 AAFC02066640
AAFC02066640 rs29009898
rs29012719 AAFC02008675
AAFC02008675
AAFC02171108
rs29011583 rs29011584
rs29011272
rs29012670 AAFC02018031
SCAFFOLD145058_39537
AAFC02036736 rs29017036
AAFC02103167 rs29013477
rs29017074 rs29017076
rs29017081
10
10
10
10
10
10

BTA27

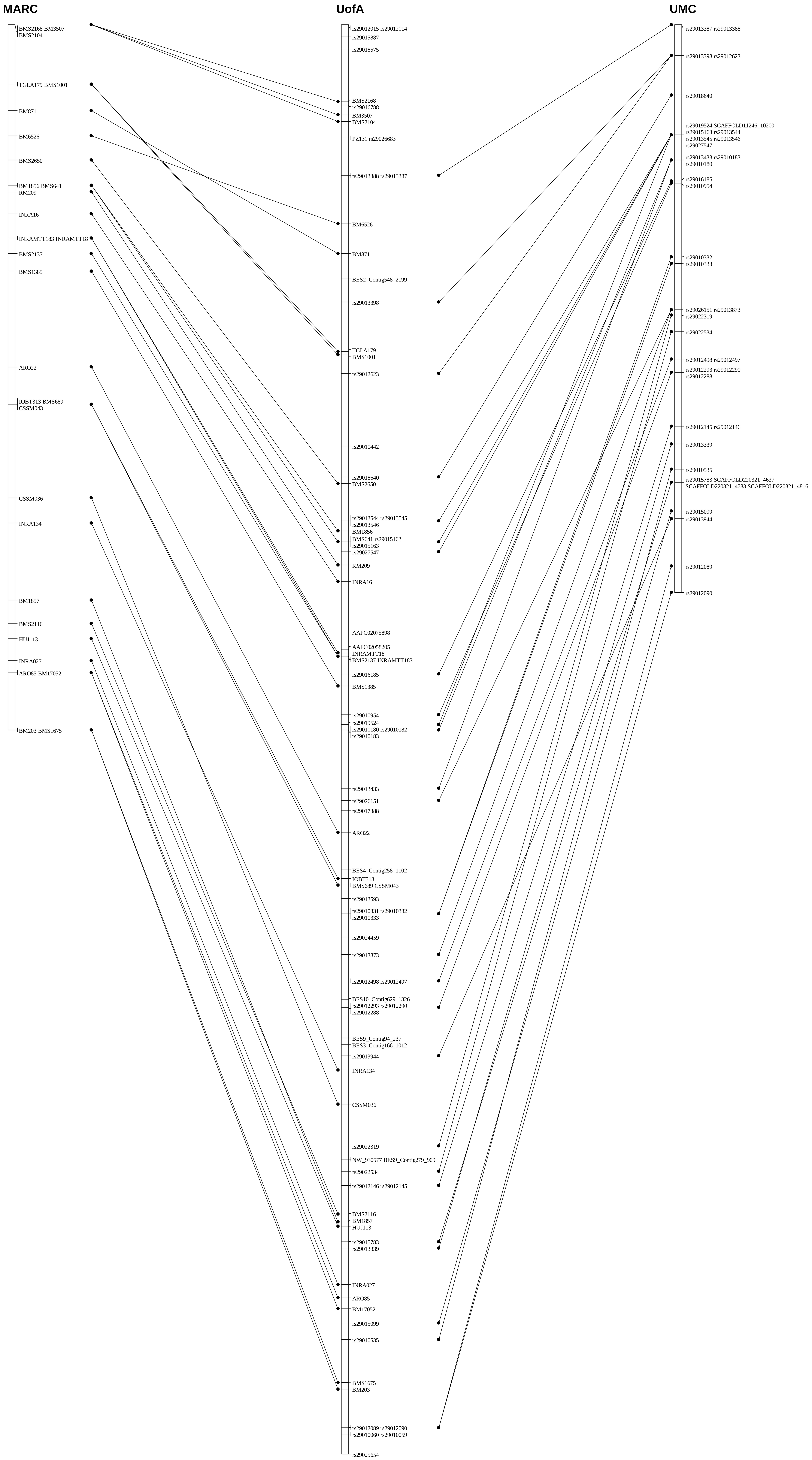
MARC
UofA
UMC
BMS2168 BM3507
BMS2104
TGLA179 BMS1001
BM871
BM6526
BMS2650
BM1856 BMS641
RM209
INRA16
INRAMTT183 INRAMTT18
BMS2137
BMS1385
ARO22
IOBT313 BMS689
CSSM043
CSSM036
INRA134
BM1857
BMS2116
HUJ113
INRA027
ARO85 BM17052
BM203 BMS1675
rs29012015 rs29012014
rs29015887
rs29018575
BMS2168
rs29016788
BM3507
BMS2104
PZ131 rs29026683
rs29013388 rs29013387
BM6526
BM871
BES2_Contig548_2199
rs29013398
TGLA179
BMS1001
rs29012623
rs29010442
rs29018640
BMS2650
rs29013544 rs29013545
rs29013546
BM1856
BMS641 rs29015162
rs29015163
rs29027547
RM209
INRA16
AAFC02075898
AAFC02058205
INRAMTT18
BMS2137 INRAMTT183
rs29016185
BMS1385
rs29010954
rs29019524
rs29010180 rs29010182
rs29010183
rs29013433
rs29026151
rs29017388
ARO22
BES4_Contig258_1102
IOBT313
BMS689 CSSM043
rs29013593
rs29010331 rs29010332
rs29010333
rs29024459
rs29013873
rs29012498 rs29012497
BES10_Contig629_1326
rs29012293 rs29012290
rs29012288
BES9_Contig94_237
BES3_Contig166_1012
rs29013944
INRA134
CSSM036
rs29022319
NW_930577 BES9_Contig279_909
rs29022534
rs29012146 rs29012145
BMS2116
BM1857
HUJ113
rs29015783
rs29013339
INRA027
ARO85
BM17052
rs29015099
rs29010535
BMS1675
BM203
rs29012089 rs29012090
rs29010060 rs29010059
rs29025654
rs29013387 rs29013388
rs29013398 rs29012623
rs29018640
rs29019524 SCAFFOLD11246_10200
rs29015163 rs29013544
rs29013545 rs29013546
rs29027547
rs29013433 rs29010183
rs29010180
rs29016185
rs29010954
rs29010332
rs29010333
rs29026151 rs29013873
rs29022319
rs29022534
rs29012498 rs29012497
rs29012293 rs29012290
rs29012288
rs29012145 rs29012146
rs29013339
rs29010535
rs29015783 SCAFFOLD220321_4637
SCAFFOLD220321_4783 SCAFFOLD220321_4816
rs29015099
rs29013944
rs29012089
rs29012090

BTA27

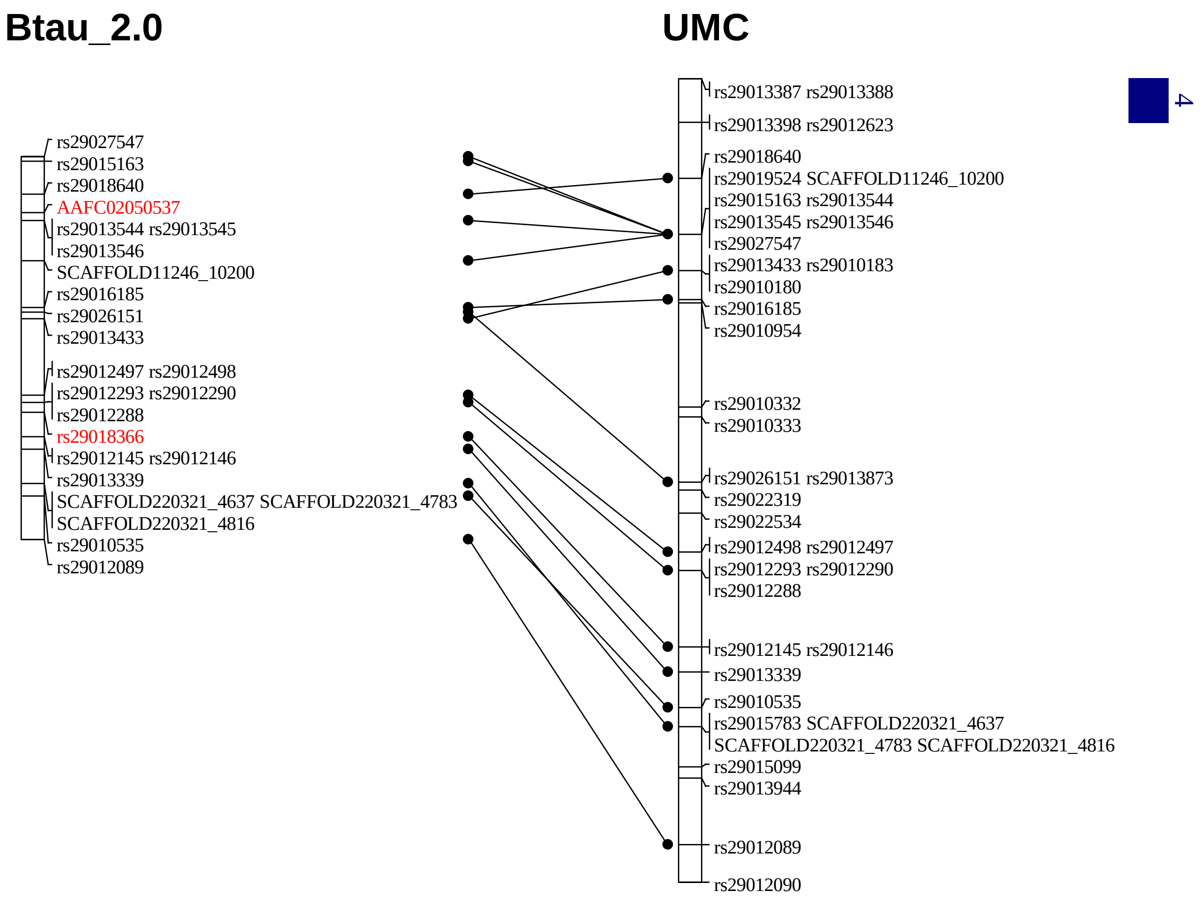

Btau_2.0
UMC
rs29027547
rs29015163
rs29018640
AAFC02050537
rs29013544 rs29013545
rs29013546
SCAFFOLD11246_10200
rs29016185
rs29026151
rs29013433
rs29012497 rs29012498
rs29012293 rs29012290
rs29012288
rs29018366
rs29012145 rs29012146
rs29013339
SCAFFOLD220321_4637 SCAFFOLD220321_4783
SCAFFOLD220321_4816
rs29010535
rs29012089
rs29013387 rs29013388
rs29013398 rs29012623
rs29018640
rs29019524 SCAFFOLD11246_10200
rs29015163 rs29013544
rs29013545 rs29013546
rs29027547
rs29013433 rs29010183
rs29010180
rs29016185
rs29010954
rs29010332
rs29010333
rs29026151 rs29013873
rs29022319
rs29022534
rs29012498 rs29012497
rs29012293 rs29012290
rs29012288
rs29012145 rs29012146
rs29013339
rs29010535
rs29015783 SCAFFOLD220321_4637
SCAFFOLD220321_4783 SCAFFOLD220321_4816
rs29015099
rs29013944
rs29012089
rs29012090
4

BTA28

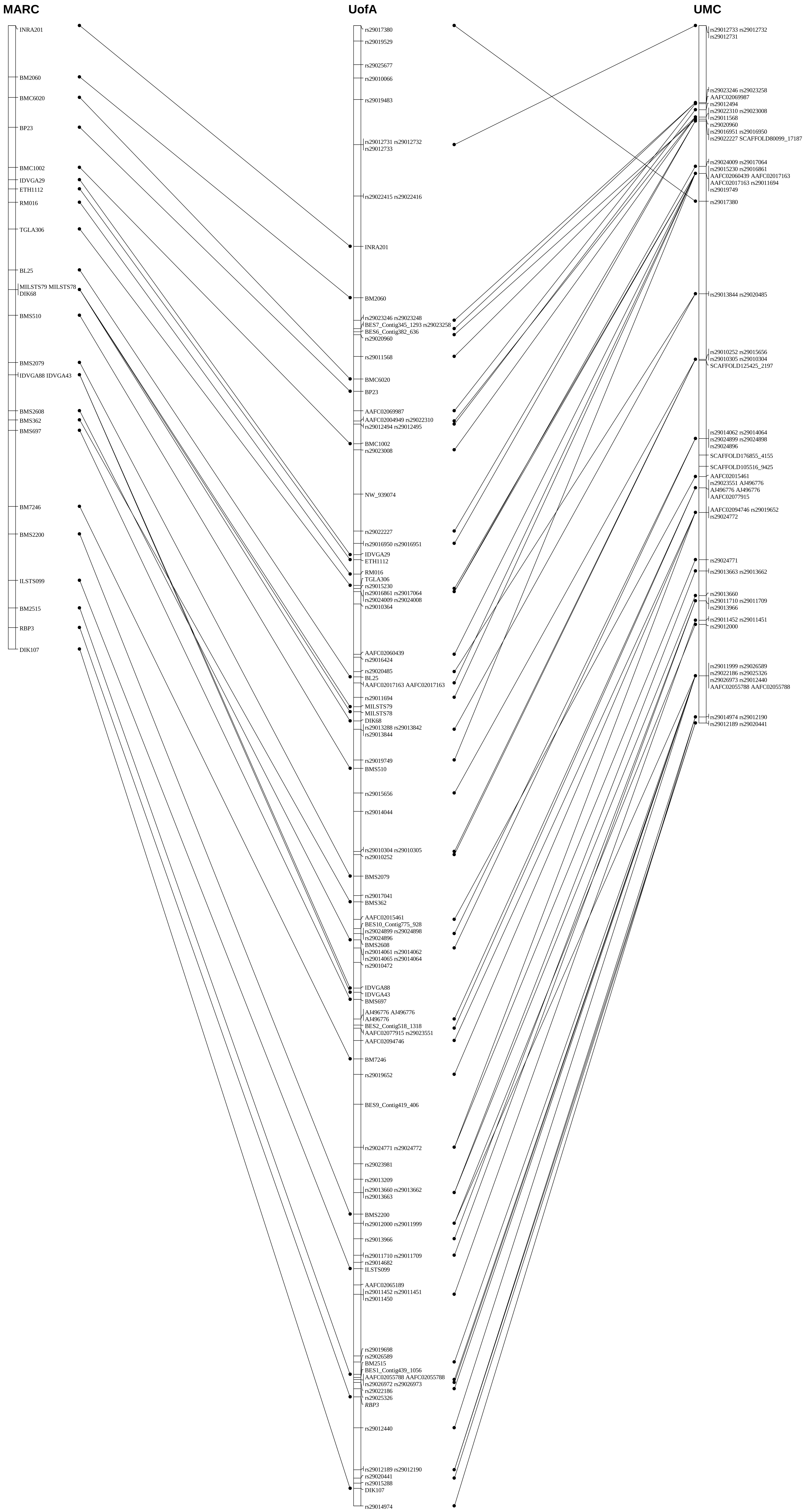
MARC
INRA201
BM2060
BMC6020
BP23
BMC1002
IDVGA29
ETH1112
RM016
TGLA306
BL25
MILSTS79 MILSTS78
DIK68
BMS510
BMS2079
IDVGA88 IDVGA43
BMS2608
BMS362
BMS697
BM7246
BMS2200
ILSTS099
BM2515
RBP3
DIK107
UofA
rs29017380
rs29019529
rs29025677
rs29010066
rs29019483
rs29012731 rs29012732
rs29012733
rs29022415 rs29022416
INRA201
BM2060
rs29023246 rs29023248
BES7_Contig345_1293 rs29023258
BES6_Contig382_636
rs29020960
rs29011568
BMC6020
BP23
AAFC02069987
AAFC02004949 rs29022310
rs29012494 rs29012495
BMC1002
rs29023008
NW_939074
rs29022227
rs29016950 rs29016951
IDVGA29
ETH1112
RM016
TGLA306
rs29015230
rs29016861 rs29017064
rs29024009 rs29024008
rs29010364
AAFC02060439
rs29016424
rs29020485
BL25
AAFC02017163 AAFC02017163
rs29011694
MILSTS79
MILSTS78
DIK68
rs29013288 rs29013842
rs29013844
rs29019749
BMS510
rs29015656
rs29014044
rs29010304 rs29010305
rs29010252
BMS2079
rs29017041
BMS362
AAFC02015461
BES10_Contig775_928
rs29024899 rs29024898
rs29024896
BMS2608
rs29014061 rs29014062
rs29014065 rs29014064
rs29010472
IDVGA88
IDVGA43
BMS697
AJ496776 AJ496776
AJ496776
BES2_Contig518_1318
AAFC02077915 rs29023551
AAFC02094746
BM7246
rs29019652
BES9_Contig419_406
rs29024771 rs29024772
rs29023981
rs29013209
rs29013660 rs29013662
rs29013663
BMS2200
rs29012000 rs29011999
rs29013966
rs29011710 rs29011709
rs29014682
ILSTS099
AAFC02065189
rs29011452 rs29011451
rs29011450
rs29019698
rs29026589
BM2515
BES1_Contig439_1056
AAFC02055788 AAFC02055788
rs29026972 rs29026973
rs29022186
rs29025326
RBP3
rs29012440
rs29012189 rs29012190
rs29020441
rs29015288
DIK107
rs29014974
UMC
rs29012733 rs29012732
rs29012731
rs29023246 rs29023258
AAFC02069987
rs29012494
rs29022310 rs29023008
rs29011568
rs29020960
rs29016951 rs29016950
rs29022227 SCAFFOLD80099_17187
rs29024009 rs29017064
rs29015230 rs29016861
AAFC02060439 AAFC02017163
AAFC02017163 rs29011694
rs29019749
rs29017380
rs29013844 rs29020485
rs29010252 rs29015656
rs29010305 rs29010304
SCAFFOLD125425_2197
rs29014062 rs29014064
rs29024899 rs29024898
rs29024896
SCAFFOLD176855_4155
SCAFFOLD105516_9425
AAFC02015461
rs29023551 AJ496776
AJ496776 AJ496776
AAFC02077915
AAFC02094746 rs29019652
rs29024772
rs29024771
rs29013663 rs29013662
rs29013660
rs29011710 rs29011709
rs29013966
rs29011452 rs29011451
rs29012000
rs29011999 rs29026589
rs29022186 rs29025326
rs29026973 rs29012440
AAFC02055788 AAFC02055788
rs29014974 rs29012190
rs29012189 rs29020441

BTA28

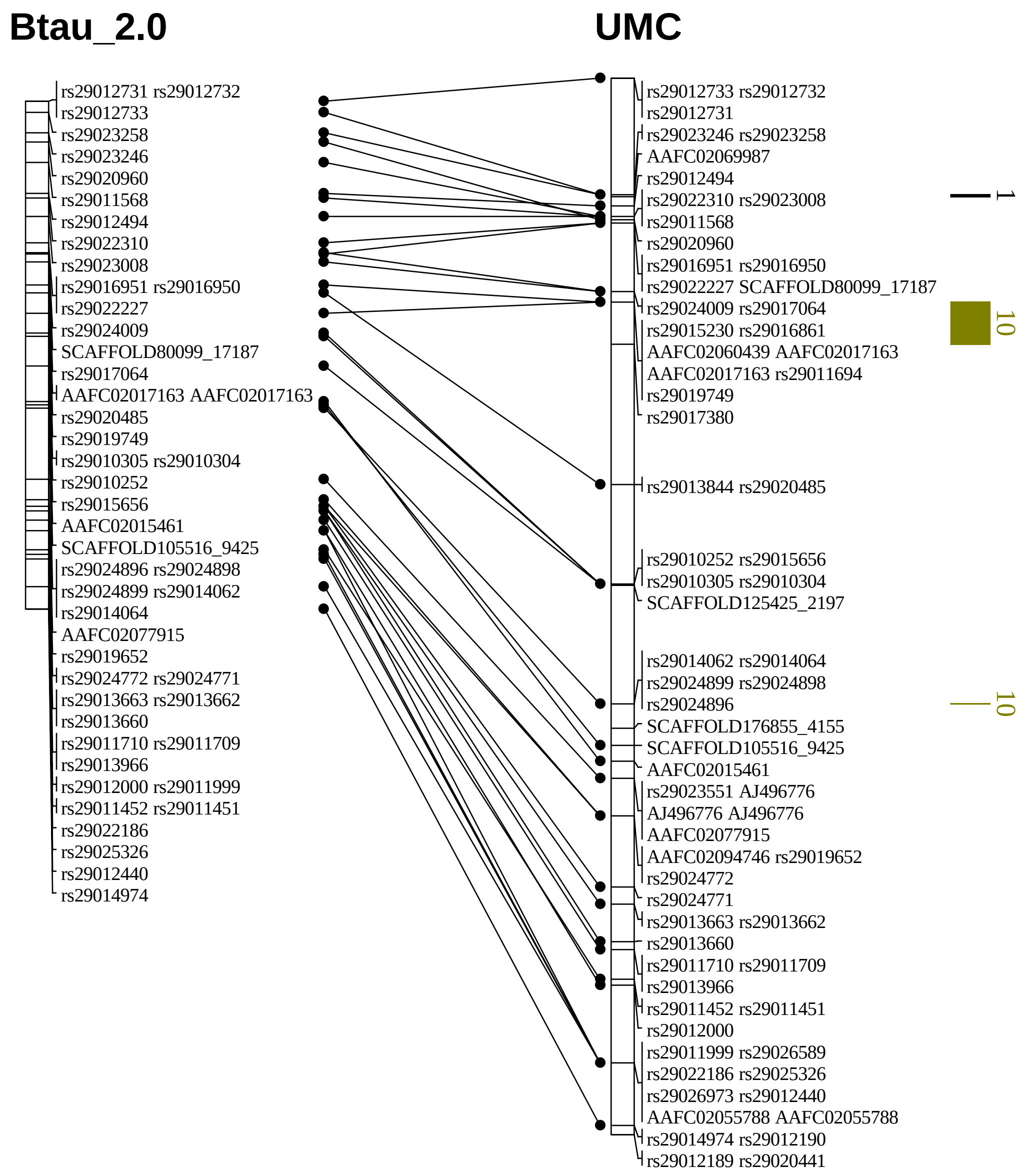
Btau_2.0
UMC
rs29012731 rs29012732
rs29012733
rs29023258
rs29023246
rs29020960
rs29011568
rs29012494
rs29022310
rs29023008
rs29016951 rs29016950
rs29022227
rs29024009
SCAFFOLD80099_17187
rs29017064
AAFC02017163 AAFC02017163
rs29020485
rs29019749
rs29010305 rs29010304
rs29010252
rs29015656
AAFC02015461
SCAFFOLD105516_9425
rs29024896 rs29024898
rs29024899 rs29014062
rs29014064
AAFC02077915
rs29019652
rs29024772 rs29024771
rs29013663 rs29013662
rs29013660
rs29011710 rs29011709
rs29013966
rs29012000 rs29011999
rs29011452 rs29011451
rs29022186
rs29025326
rs29012440
rs29014974
rs29012733 rs29012732
rs29012731
rs29023246 rs29023258
AAFC02069987
rs29012494
rs29022310 rs29023008
rs29011568
rs29020960
rs29016951 rs29016950
rs29022227 SCAFFOLD80099_17187
rs29024009 rs29017064
rs29015230 rs29016861
AAFC02060439 AAFC02017163
AAFC02017163 rs29011694
rs29019749
rs29017380
rs29013844 rs29020485
rs29010252 rs29015656
rs29010305 rs29010304
SCAFFOLD125425_2197
rs29014062 rs29014064
rs29024899 rs29024898
rs29024896
SCAFFOLD176855_4155
SCAFFOLD105516_9425
AAFC02015461
rs29023551 AJ496776
AJ496776 AJ496776
AAFC02077915
AAFC02094746 rs29019652
rs29024772
rs29024771
rs29013663 rs29013662
rs29013660
rs29011710 rs29011709
rs29013966
rs29011452 rs29011451
rs29012000
rs29011999 rs29026589
rs29022186 rs29025326
rs29026973 rs29012440
AAFC02055788 AAFC02055788
rs29014974 rs29012190
rs29012189 rs29020441
1
10
10

BTA29

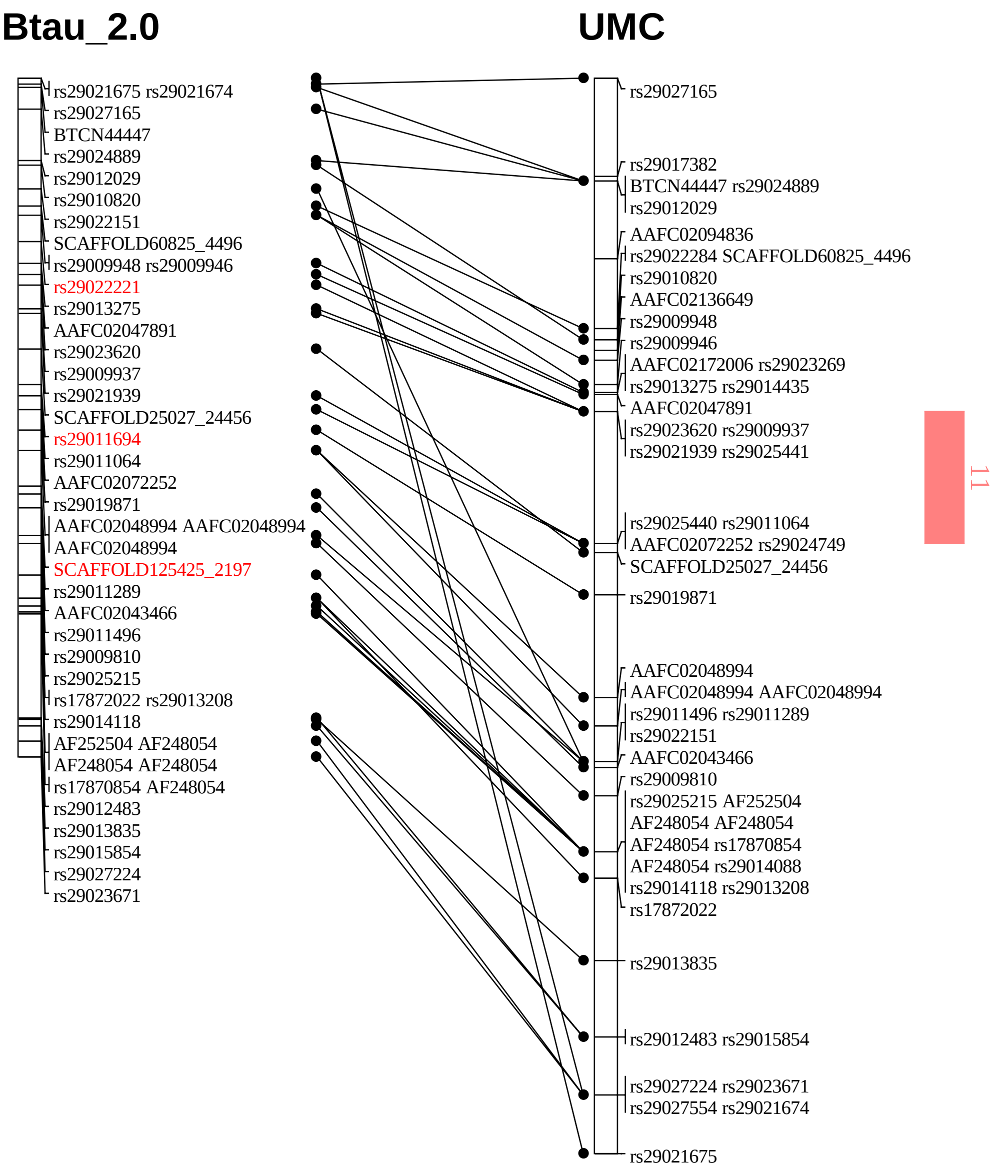
Btau_2.0
UMC
rs29021675 rs29021674
rs29027165
BTCN44447
rs29024889
rs29012029
rs29010820
rs29022151
SCAFFOLD60825_4496
rs29009948 rs29009946
rs29022221
rs29013275
AAFC02047891
rs29023620
rs29009937
rs29021939
SCAFFOLD25027_24456
rs29011694
rs29011064
AAFC02072252
rs29019871
AAFC02048994 AAFC02048994
AAFC02048994
SCAFFOLD125425_2197
rs29011289
AAFC02043466
rs29011496
rs29009810
rs29025215
rs17872022 rs29013208
rs29014118
AF252504 AF248054
AF248054 AF248054
rs17870854 AF248054
rs29012483
rs29013835
rs29015854
rs29027224
rs29023671
rs29027165
rs29017382
BTCN44447 rs29024889
rs29012029
AAFC02094836
rs29022284 SCAFFOLD60825_4496
rs29010820
AAFC02136649
rs29009948
rs29009946
AAFC02172006 rs29023269
rs29013275 rs29014435
AAFC02047891
rs29023620 rs29009937
rs29021939 rs29025441
rs29025440 rs29011064
AAFC02072252 rs29024749
SCAFFOLD25027_24456
rs29019871
AAFC02048994
AAFC02048994 AAFC02048994
rs29011496 rs29011289
rs29022151
AAFC02043466
rs29009810
rs29025215 AF252504
AF248054 AF248054
AF248054 rs17870854
AF248054 rs29014088
rs29014118 rs29013208
rs17872022
rs29013835
rs29012483 rs29015854
rs29027224 rs29023671
rs29027554 rs29021674
rs29021675
11

BTA29

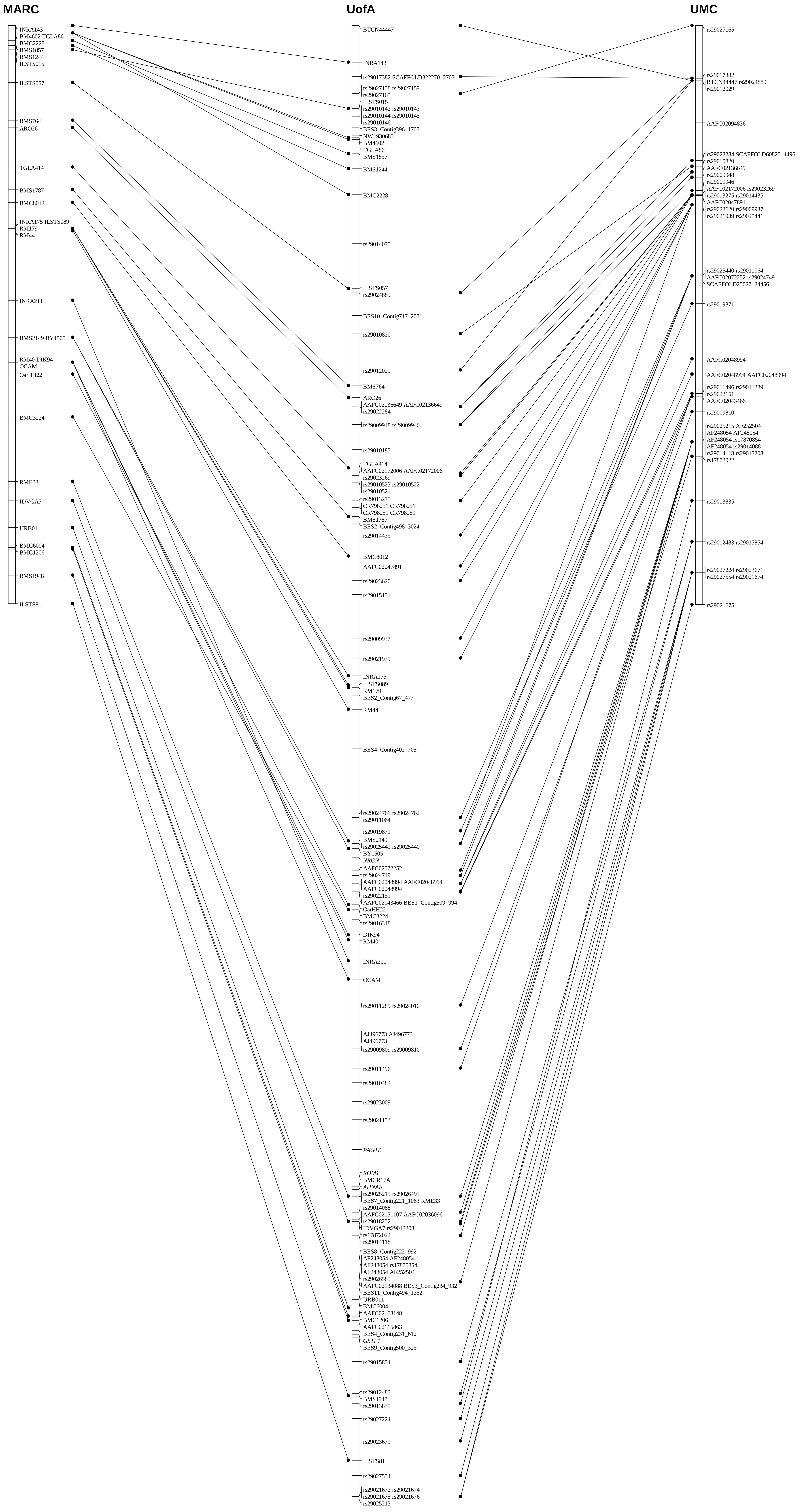

MARC
INRA143
BM4602 TGLA86
BMC2228
BMS1857
BMS1244
ILSTS015
ILSTS057
BMS764
ARO26
TGLA414
BMS1787
BMC8012
INRA175 ILSTS089
RM179
RM44
INRA211
BMS2149 BY1505
RM40 DIK94
OCAM
OarHH22
BMC3224
RME33
IDVGA7
URB011
BMC6004
BMC1206
BMS1948
ILSTS81
UofA
BTCN44447
INRA143
rs29017382 SCAFFOLD322270_2707
rs29027158 rs29027159
rs29027165
ILSTS015
rs29010142 rs29010143
rs29010144 rs29010145
rs29010146
BES3_Contig396_1707
NW_930683
BM4602
TGLA86
BMS1857
BMS1244
BMC2228
rs29014075
ILSTS057
rs29024889
BES10_Contig717_2071
rs29010820
rs29012029
BMS764
ARO26
AAFC02136649 AAFC02136649
rs29022284
rs29009948 rs29009946
rs29010185
TGLA414
AAFC02172006 AAFC02172006
rs29023269
rs29010523 rs29010522
rs29010521
rs29013275
CR798251 CR798251
CR798251 CR798251
BMS1787
BES2_Contig498_3024
rs29014435
BMC8012
AAFC02047891
rs29023620
rs29015151
rs29009937
rs29021939
INRA175
ILSTS089
RM179
BES2_Contig67_477
RM44
BES4_Contig402_705
rs29024761 rs29024762
rs29011064
rs29019871
BMS2149
rs29025441 rs29025440
BY1505
NRGN
AAFC02072252
rs29024749
AAFC02048994 AAFC02048994
AAFC02048994
rs29022151
AAFC02043466 BES1_Contig509_994
OarHH22
BMC3224
rs29016318
DIK94
RM40
INRA211
OCAM
rs29011289 rs29024010
AJ496773 AJ496773
AJ496773
rs29009809 rs29009810
rs29011496
rs29010482
rs29023009
rs29021153
PAG1B
ROM1
BMCR17A
AHNAK
rs29025215 rs29026495
BES7_Contig221_1063 RME33
rs29014088
AAFC02151107 AAFC02036096
rs29018252
IDVGA7 rs29013208
rs17872022
rs29014118
BES8_Contig222_992
AF248054 AF248054
AF248054 rs17870854
AF248054 AF252504
rs29026585
AAFC02134088 BES3_Contig234_932
BES11_Contig494_1352
URB011
BMC6004
AAFC02168148
BMC1206
AAFC02115863
BES4_Contig231_612
GSTP1
BES9_Contig500_325
rs29015854
rs29012483
BMS1948
rs29013835
rs29027224
rs29023671
ILSTS81
rs29027554
rs29021672 rs29021674
rs29021675 rs29021676
rs29025213
UMC
rs29027165
rs29017382
BTCN44447 rs29024889
rs29012029
AAFC02094836
rs29022284 SCAFFOLD60825_4496
rs29010820
AAFC02136649
rs29009948
rs29009946
AAFC02172006 rs29023269
rs29013275 rs29014435
AAFC02047891
rs29023620 rs29009937
rs29021939 rs29025441
rs29025440 rs29011064
AAFC02072252 rs29024749
SCAFFOLD25027_24456
rs29019871
AAFC02048994
AAFC02048994 AAFC02048994
rs29011496 rs29011289
rs29022151
AAFC02043466
rs29009810
rs29025215 AF252504
AF248054 AF248054
AF248054 rs17870854
AF248054 rs29014088
rs29014118 rs29013208
rs17872022
rs29013835
rs29012483 rs29015854
rs29027224 rs29023671
rs29027554 rs29021674
rs29021675
